# Selective Approaches to α‐ and β‐Arylated Vinyl Ethers

**DOI:** 10.1002/anie.202109801

**Published:** 2021-11-23

**Authors:** Anna Domzalska‐Pieczykolan, Ignacio Funes‐Ardoiz, Bartłomiej Furman, Carsten Bolm

**Affiliations:** ^1^ Institut für Organische Chemie RWTH Aachen University Landoltweg 1 52074 Aachen Germany; ^2^ Departamento de Química Centro de Investigación en Síntesis Química (CISQ) University of La Rioja Madre de Dios 53 26006 Logroño Spain; ^3^ Institute of Organic Chemistry Polish Academy of Sciences Kasprzaka 44/52 01-224 Warsaw Poland

**Keywords:** arylation reaction, DFT studies, enol ether, Heck reaction, palladium catalysis

## Abstract

We developed simple and efficient protocols for palladium‐catalyzed regioselective α‐ and β‐arylations of structurally diverse vinyl ethers. Both catalytic methods proceed under relatively mild reactions conditions applying to a broad substrate range including more complex compounds providing arylated glucal or isochromene. Lacking the common requirement of a large reagent excess, the transformations are highly economic and limiting the waste production. Results from computational studies (DFT) provided insight into the key factors determining the pronounced regioselectivities of the investigated reactions.

## Graphical abstract

Benzyl vinyl ethers have selectively been arylated at either α or β position by fine‐tuned Heck‐type palladium catalyses. In contrast to previous protocols, no excess of arylating reagent or substrate chelation is necessary. For achieving good yields and high α/β‐selectivities various factors determining the catalytic system had to be optimized. The results of DFT computational studies correlate selectivity with electron richness on the Pd center.

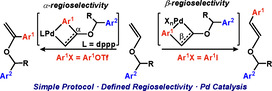

## Introduction

Due to the presence of electron‐enriched double bonds, vinyl ethers are molecules of great importance for organic chemistry. They exhibit unique properties and undergo a variety of transformations leading to structurally diverse, complex molecules.^[1]^ Consequently, they are considered as highly valuable synthetic building blocks.^[2]^ However, stereochemically defined vinyl ether formations through *trans*‐vinylation reactions, double bond isomerizations, eliminations, olefinations, or coupling reactions are commonly limited to the simplest (*n*‐butyl or ethyl) or non‐substituted derivatives.^[3]^ Syntheses of substituted and more complex enol ethers such as α‐ and β‐(benzyloxy)styrene have been described but their preparations require toxic, expensive or hard‐to‐obtain reagents making those methods less attractive.^[4]^

**Scheme 1:**
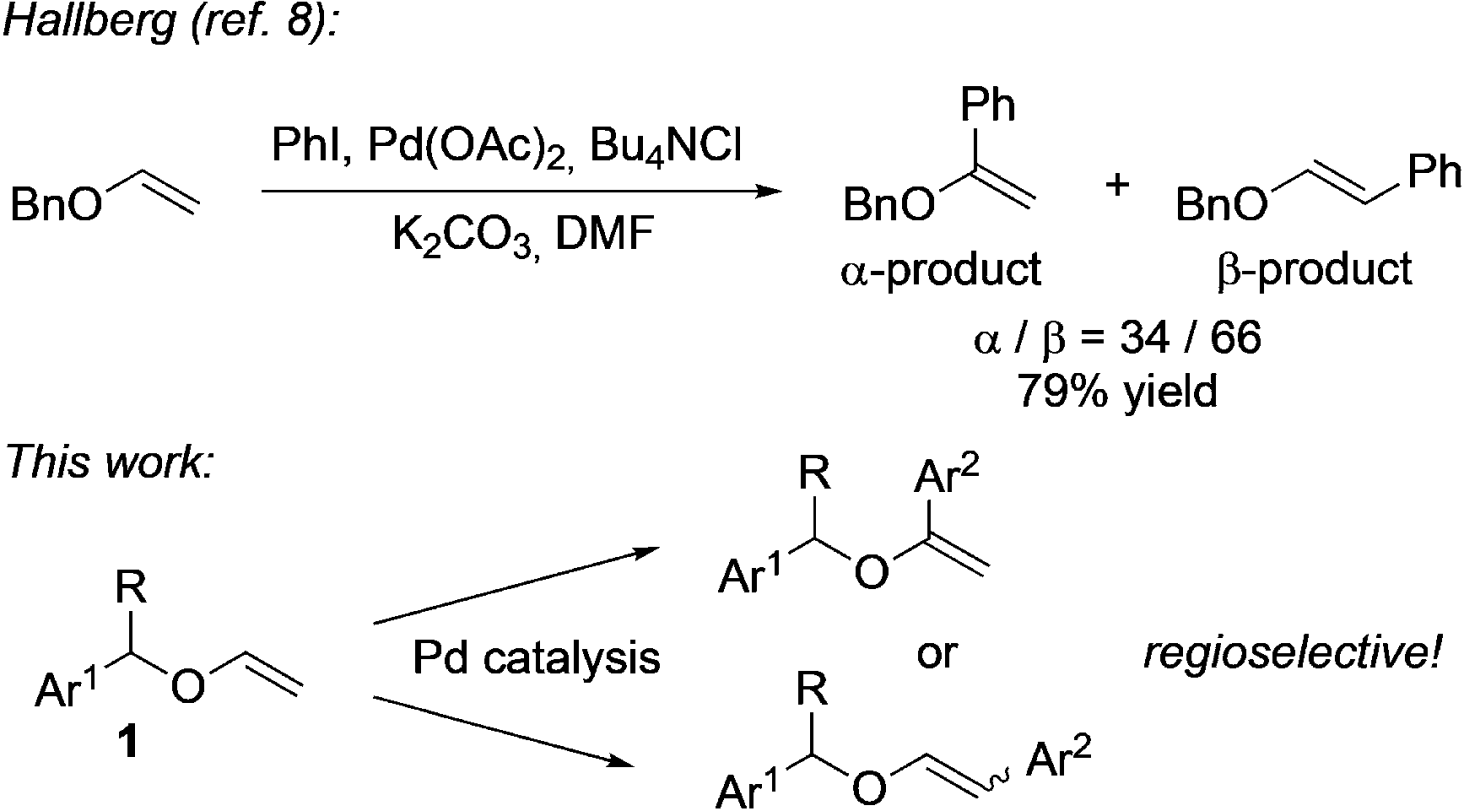
Heck‐type arylations of vinyl ethers as reported by Hallberg^[8]^ and developed here.

Among the more recent methodologies for modifications of vinyl ethers are palladium‐catalyzed Heck reactions.^[5]^ However, these very attractive direct C−H activation methods have only been applied to simple model compounds such as *n*‐butyl vinyl ethers (Scheme 1).^[6]^ Furthermore, they are characterized by low regioselectivities and a poor reagent economy with the requirement of large excesses of starting materials.[^6a^, ^6b^, ^6c^, ^6m^, ^6n^, ^6o^, ^6p^, ^6q^, ^6r^, ^6s^, ^6t^] Studies beyond model reactions[^6f^, ^6g^, ^6h^, ^6i^, ^6j^, ^6k^, ^6l^, ^7^] focussing on conversions of substituted vinyl ethers and their regioselective functionalizations are lacking. In light of the above facts and inspired by the seminal work of Hallberg from 1993,^[8]^ we initiated a study directed towards the development of regioselective Heck‐type arylation reactions of benzyl‐substituted vinyl ethers **1** (Scheme 1).

## Results and Discussion

**Scheme 2:**
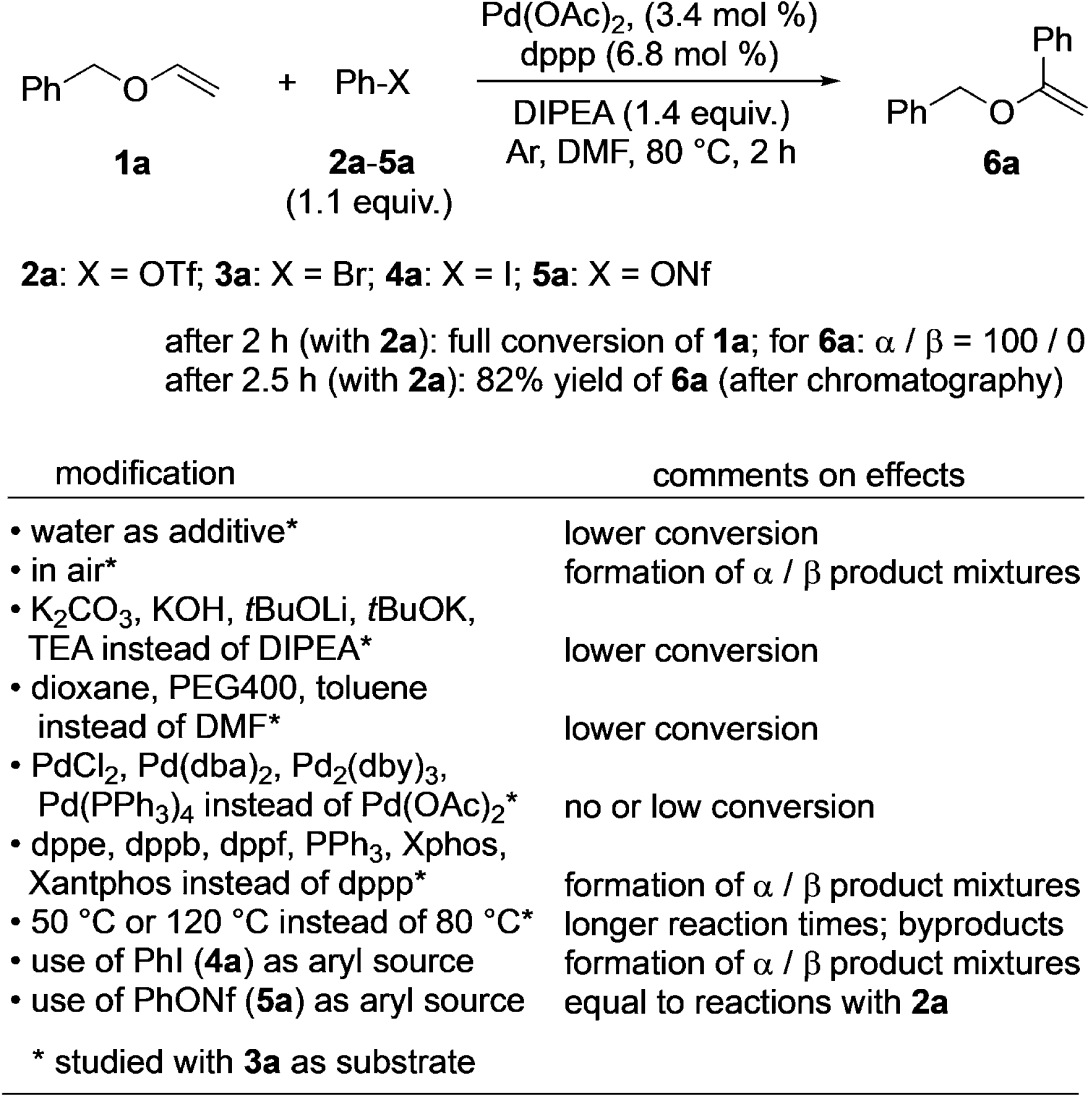
Optimized procedure for the α‐phenylation of **1 a** to give **6 a** and variations thereof; for details see Table S1 in the Supporting Information.

At the beginning of our investigation, a simple and efficient method for the preparation of benzyl‐type vinyl ethers **1** was needed. In order to ensure that the subsequent reaction development was not affected by the presence of trace amounts of metallic contaminations,^[9]^ the protocol by Matsubara^[10]^ involving benzyl alcohols, calcium carbide, and a superbase consisting of KOH in DMSO was applied for synthesizing key starting materials **1**. For the initial screening and optimization of the reaction conditions, vinyl ether **1 a** was selected as representative starting material. α‐Phenylations of **1 a** to give **6 a** were set in the focus first. Scheme 2 gives an overview on the findings, and all results are detailed in the Supporting Information (Table S1).

Under the optimized conditions with a combination of palladium acetate and dppp [1,3‐bis(diphenylphosphino)propane] in the presence of Hünig's base (DIPEA) in DMF under argon for 2 h at 80 °C, product **6 a** was obtained from benzyl vinyl ether (**1 a**) and phenyl triflate (**2 a**) in 82 % yield after column chromatography. Noteworthy, for achieving this result, only 1.1 equiv of **2 a** was necessary, contrasting previous methods, which required a large access of substrate (vide supra). From early screenings with bromobenzene (**3 a**) instead of phenyl triflate as aryl source it was apparent that water had a deleterious effect on the yield due to partial hydrolysis of starting material **1 a**.^[11]^ Similarly, the presence of even small amounts of atmospheric oxygen proved unfavorable leading to mixtures of α‐and β‐regioisomers. Thus, for achieving a high α‐arylation the catalysis had to be performed in degassed DMF under argon. The use of other bases than DIPEA led to lower conversions of **1 a** (Scheme 2). Interestingly, the regioselectivity remained unaffected. Among dioxane, PEG400, toluene, and DMF the latter solvent gave the best conversion of **1 a**. Screening of various palladium complexes revealed that Pd(OAc)_2_ was the most effective catalyst metal species (Scheme 2). The ligand type affected the regioselectivity of the arylation (Scheme 2 and Table S1 in the Supporting Information) with dppp providing the best results for the α‐phenylation.^[12]^ Performing the catalysis at 50 °C and 120 °C instead of 80 °C led to longer reaction times and the formation of undesired by‐products, respectively. Applying iodobenzene (**4 a**) instead of phenyl triflate afforded mixtures of α‐ and β‐regioisomers. Phenyl nonaflate (**5 a**) was an equally good aryl source as phenyl triflate (Scheme 2).

**Scheme 3:**
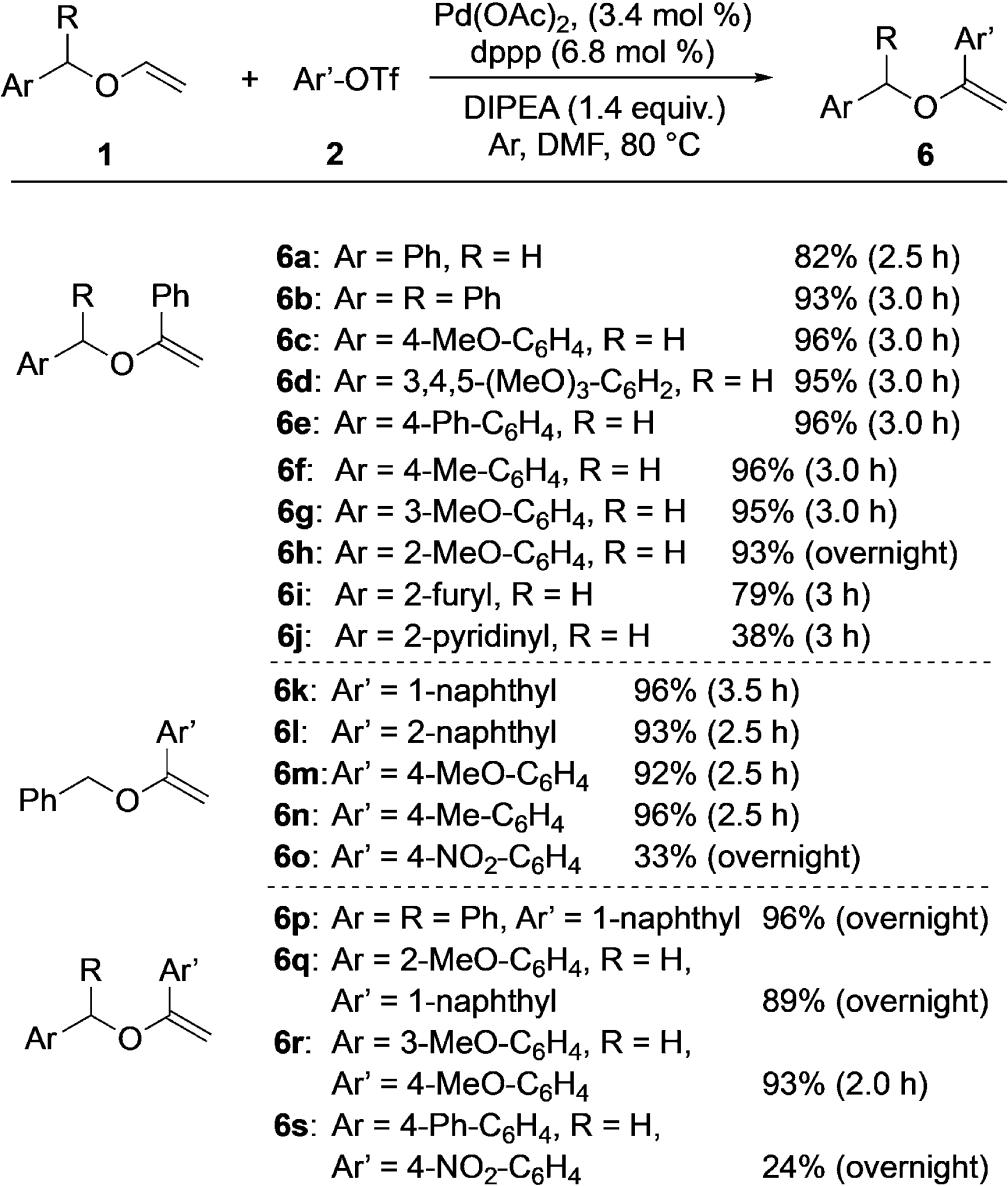
Substrate scope of the α‐arylation.

Next, the substrate scope of the α‐arylation process was investigated. The optimal reaction conditions as depicted in Scheme 2 were used as initial starting point. The structures of both coupling partners, vinyl ether **1** as well as aryl triflate **2**, were varied. Noteworthy, both compounds were applied in a 1.0:1.1 ratio thereby avoiding the previously required large excess of the arylating agent. Scheme 3 shows the results.

In the first series of experiments (leading to products **6 a**–**j**), the aryl group of the benzyl moiety of vinyl ether **1** was modified. Phenyl triflate (**2 a**) served as aryl source. In most cases the yields of the corresponding products were high, reaching 96 % for compounds **6 c**, **6 e**, and **6 f**. Diphenylmethyl vinyl ether (**1 b**) reacted well too, leading to **6 b** in 93 % yield. Phenyl, methyl, and methoxy substituents were tolerated and the reactions proceeded without significant impact. Probably due to steric factors, an extension of the reaction time from 3 h to overnight was required for achieving a yield of 93 % of product **6 h** with a 2‐methoxy‐substituted aryl group. Vinyl ethers with hetarenes led to lower yields of the corresponding products as found for 2‐furyl‐ and 2‐pyridinyl‐substituted **6 i** and **6 j**, which were obtained in 79 % and 38 % yield, respectively. From all of these results we conclude that chelating effects were not required for the high α‐selectivity of the arylation. If chelation was possible (as for **1 h**–**j**), it had no effect or appeared to hamper the process.

In the next series of experiments, benzyl vinyl ether (**1 a**) was the starting material and the aryl triflate structure was varied (Scheme 3). While the reactions with 1‐naphthyl, 2‐naphthyl‐, and 4‐substituted aryl triflates proceeded well, providing products **6 k**–**n** in yields ranging from 92 % to 96 %, the use of 4‐nitro‐containing aryl triflate **2 o** gave **6 o** in only 33 % yield even after an extended reaction time (overnight). The electron‐withdrawing nitro substituent exhibited a negative effect on the product formation.

The final four entries in Scheme 3 related to product formations of **6 p**–**s** reveal that also combinations of starting materials with substituents on both vinyl ethers and aryl triflates could be applied. Although extended reaction times (overnight) proved beneficial in these cases, the yields were generally high (up to 96 %). The only exception was the catalysis leading to **6 s**, which was formed in only 24 % yield. Thus, the aforementioned negative impact of the nitro group on the aryl triflate became apparent here too.

**Scheme 4:**
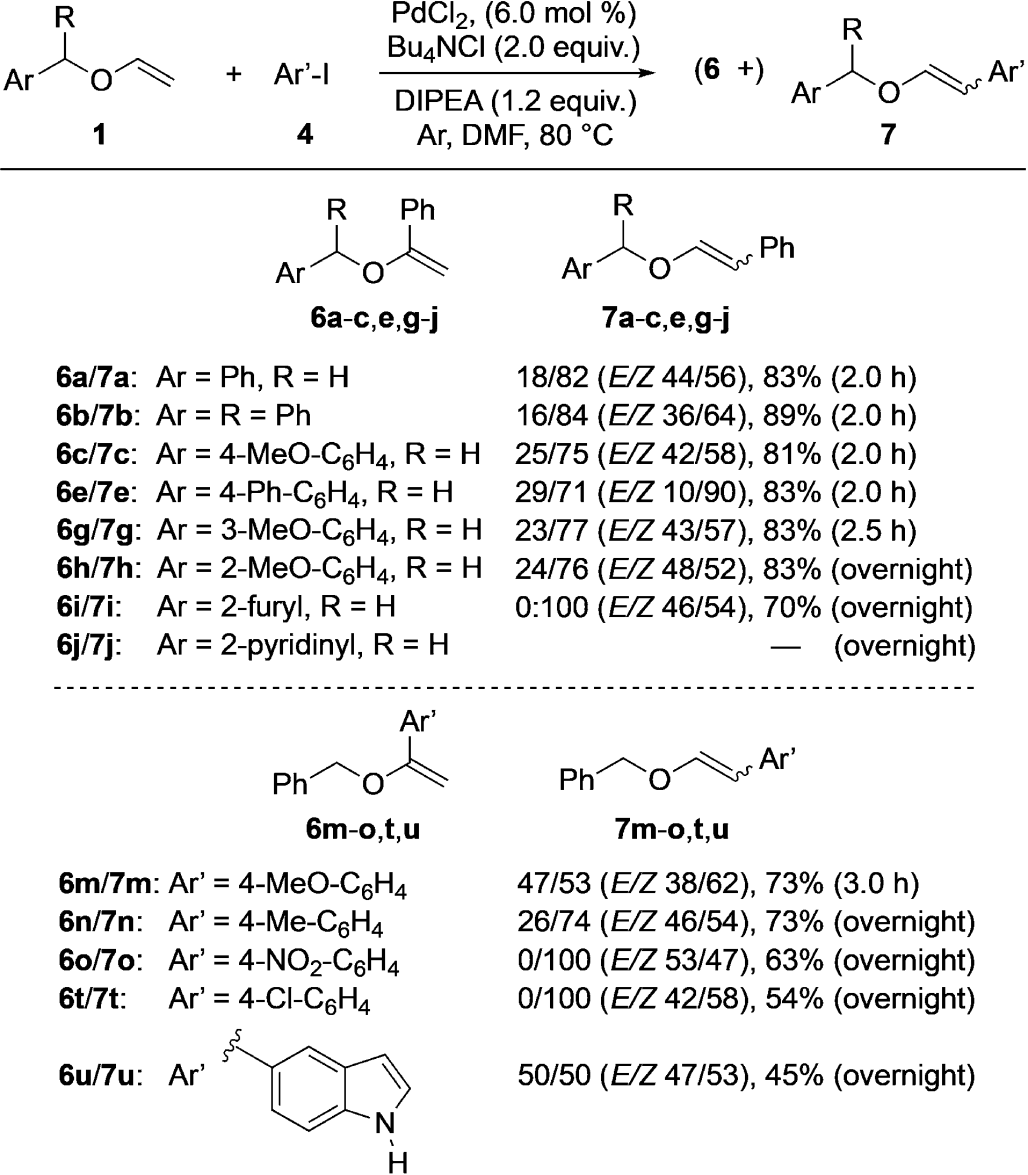
Substrate scope of the β‐selective arylation.

In his early work, Hallberg and co‐workers had also reported β‐arylations of enol ethers (Scheme 1).^[8]^ For achieving a high β‐selectivity, chelation control was the key to success. Without this effect, the α/β‐selectivity remained modest. Seeing a challenge there, the development of a protocol for β‐selective arylations of simple enol ethers was targeted. Considering Hallberg's conditions as the starting point, phenyl iodide (**4 a**) was used as aryl source. Scheme 4 reveals the optimized conditions and presents results for β‐selective arylations of various substrates. A detailed presentation of all optimized factors developed by studying reactions between **1 a** and **4 a** can be found in the Supporting Information (Table S2). A brief summary is given here: first, the presence of dppp lowered the β‐selectivity, and thus, performing the catalysis ligand‐free was beneficial. Second, the palladium source affected the conversion of **1 a** but played a minor role in determining the α/β‐selectivity of the vinyl ether arylation. Third, additives, in particular chloride salts (LiCl, Bu_4_NCl, and Aliquat 336), had a positive effect on both conversion of **1 a** and β‐regioselectivity. Fourth, the character of the base influenced the *E*/*Z* ratio of the β‐arylation product. While with DIPEA the *E* isomer dominated, inorganic bases (NaOAc and K_2_CO_3_) gave the *Z* isomer in preference. Fifth, DMF and THF proved to be the best solvents. Because the latter required a longer reaction time (24 h versus 3 h for DMF) for full conversion of **1 a**, DMF was chosen for subsequent studies. Interestingly, among all tested seven solvents (DMF, PhMe, octafluorotoluene, DCE, THF, and MeCN) acetonitrile was the only one that led to a 2:1 α‐selectivity in the phenylation of **1 a** (at a conversion of 6 % after 2 h). Finally, the best result was achieved with 6 mol % of palladium dichloride as metal catalyst with 2.0 equiv of tetrabutylammonium chloride as additive and 1.2 equiv of DIPEA as base in DMF under argon for 2 h at 80 °C.^[13]^ Under these conditions, a 18/82 mixture of **6 a** and **7 a** was obtained in 83 % yield, where β‐substituted **7 a** had an *E*/*Z* ratio of 44/56. Reactions between structurally related benzyl vinyl ethers with substituted arenes and **4 a** gave similar results (Scheme 4, top). Thus, the **6 b**/**7 b**, **6 c**/**7 c**, **6 e**/**7 e**, **6 g**/**7 g**, and **6 h**/**7 h** mixtures were isolated in yields ranging from 81 % to 89 % with the β‐arylated products **7** being formed in significant excess over their α‐arylated counterparts **6**. Probably due to chelation, 2‐furyl‐substituted **1 i** showed an exclusive regioselectivity leading to 70 % of β‐phenylated product **7 i** only. In each case, a slight preference (ca. 2:3) of the *Z* diastereomers was observed. For unknown reasons, product **7 e** was an exception with an *E*/*Z* ratio of 1:9. 2‐Pyridinyl‐substituted **1 j** did not react at all under these conditions. Couplings of benzyl vinyl ether (**1 a**) with aryl iodides other than **4 a** proceeded well too (Scheme 4, bottom). While the transformations providing **6 m**/**7 m** and **6 n**/**7 n** gave similar results as described above, the reactions with 4‐nitro iodobenzene (**4 d**) and 4‐chloro iodobenzene (**4 e**) were remarkable as β‐arylated **7 o** and **7 t**, respectively, were obtained as single regioisomers. Most likely, these results were due to the electron‐withdrawing nature of the additional substituents (nitro and chloro) on the aryl source, which supported the β‐selectivity of the process. Of interest was also the coupling of iodoindole **4 f** with **1 a**. Although the product yield was only 45 %, a 1:1 mixture of α‐arylated **6 u** and β‐substituted **7 u** was obtained. This result is noteworthy because no protection of the indole NH was required to allow the catalysis to occur.

**Scheme 5:**
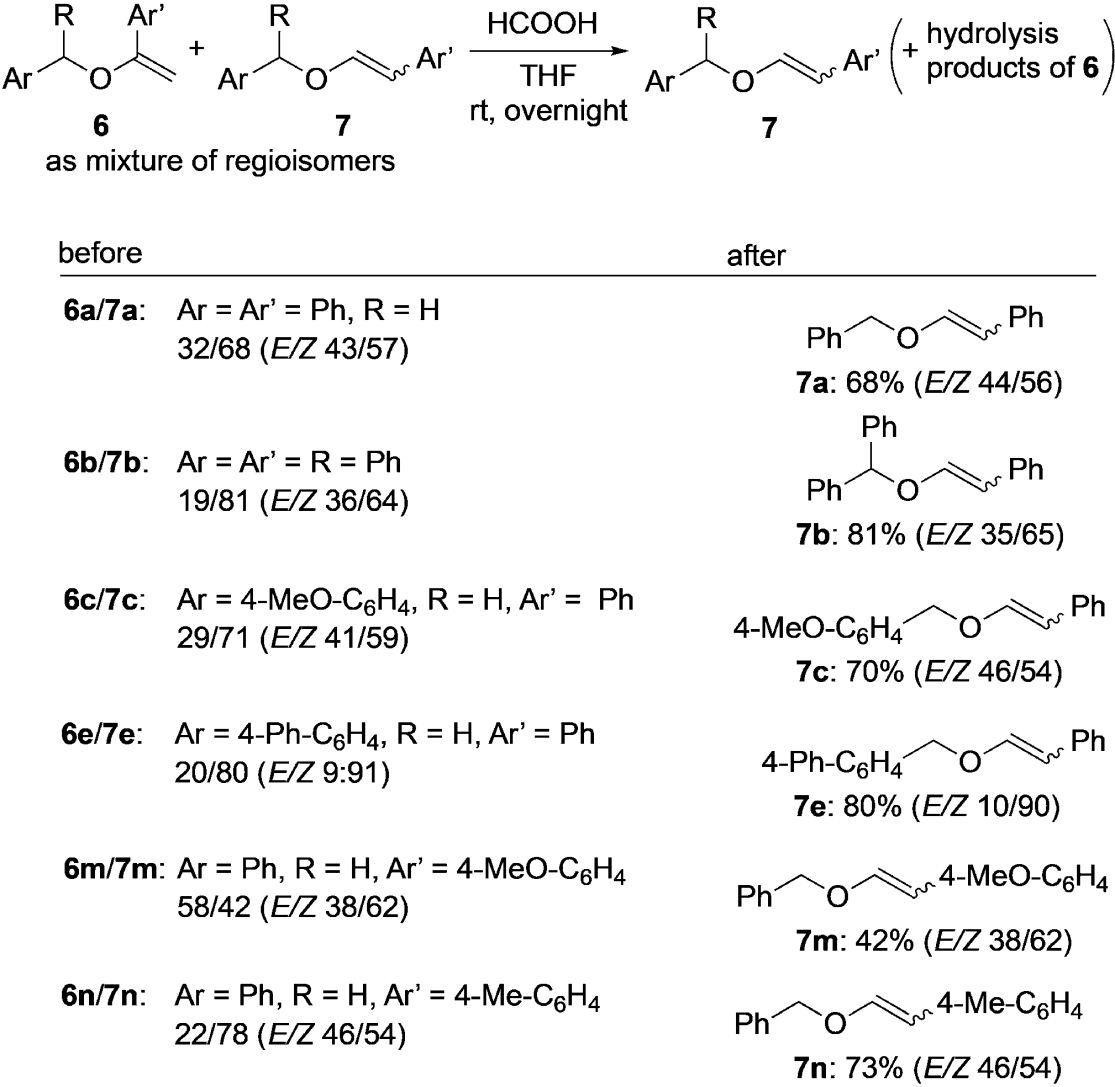
Selective hydrolysis allowing access to pure β‐substituted products.

As the data in Scheme 4 show, the development of a highly β‐selective arylation of simple vinyl ethers proved challenging. Even after an extensive experimentation, mostly mixtures of α‐ and β‐substituted products were obtained. Although the latter products dominated, only a few couplings proceeded with complete regiocontrol. Although we could not fully reach our goal, an interesting phenomenon came to our support which finally allowed us to obtain pure β‐arylated products. While attempting to achieve higher regioselectivities, we found different reaction rates of hydrolysis of the two regioisomers. A short optimization revealed that the use of a 0.01 M solution of formic acid in THF was most effective for this process. Thus, stirring a mixture of α‐arylated **6** and β‐substituted **7** in that reaction media overnight led to an exclusive hydrolysis of **6** allowing an easy isolation of **7** as pure regioisomer (Scheme 5). With minor variations the double bond geometry of **7** was retained. This was also true for product **7 e**, where the *E*/*Z* ratio was particularly high. Further experiments showed that this approach was general and could be utilized in the synthesis of various β‐arylated vinyl ethers.

**Scheme 6:**
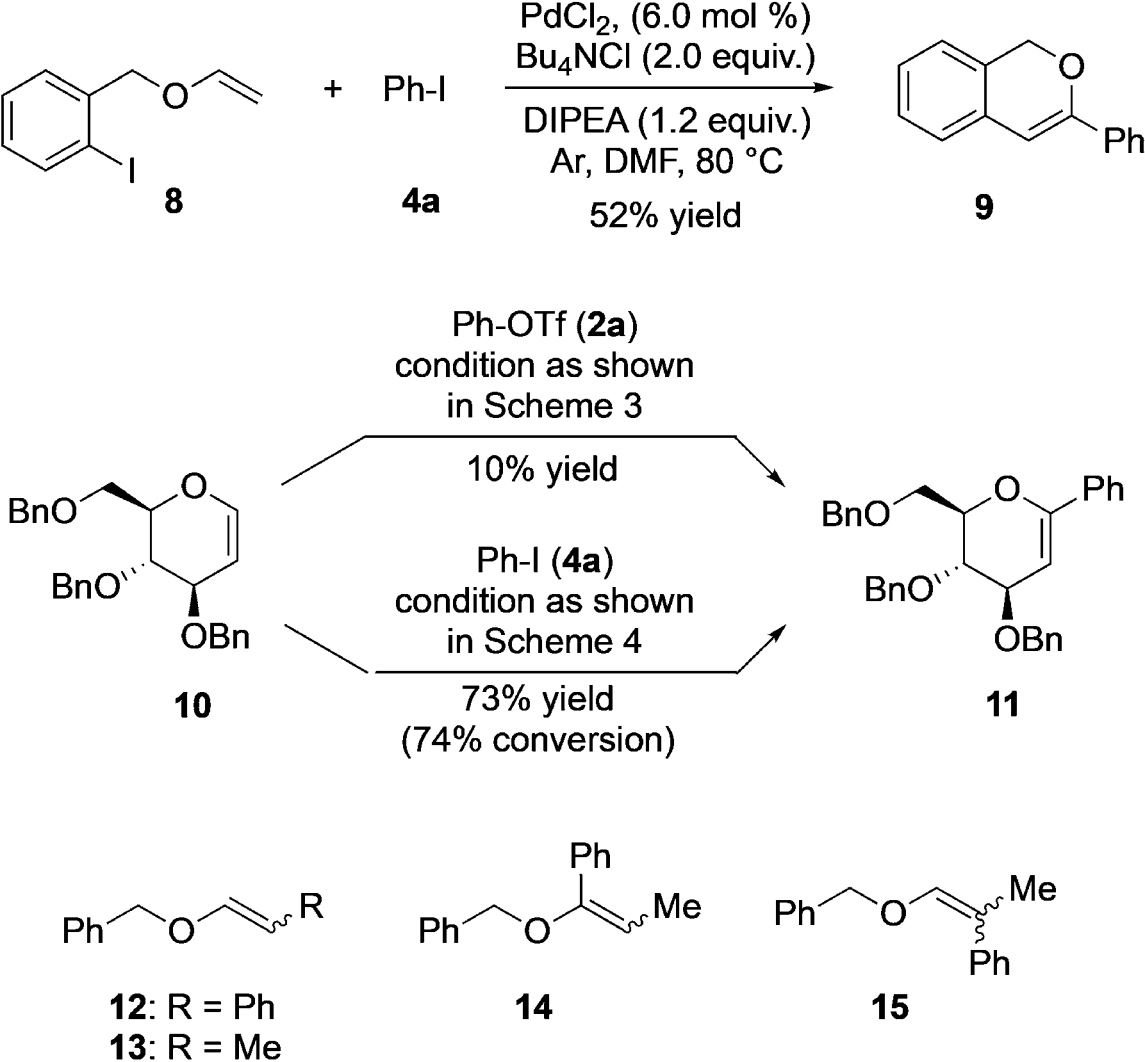
Expansion of the substrate scope.

With the intention to demonstrate the utility of the developed methodology and to extend its application, a domino diarylation involving two consecutive palladium‐catalyzed C−C‐bond formations was attempted. To our delight, this approach proved successful. Hence, using the β‐arylation conditions as summarized in Scheme 4, a mixture of 1‐[(ethenoxy)methyl]‐2‐iodobenzene (**8**) and **4 a** afforded isochromene derivative **9** in 52 % yield (Scheme 6, top). Although the precise details of the reaction sequence are unknown, we assume, based on the α‐position of the newly introduced phenyl group stemming from **4 a**, that the intramolecular ring‐closure (with β‐arylation selectivity under the chosen reaction conditions) occurs first, and that the resulting palladated heterocylic intermediate then cross‐couples with **4 a** in a second step.^[14]^

As second example, the arylation of tribenzylated glucal **10** was chosen. This transformation was of interest for two reasons: first, it involved a more complex starting material with various functional groups allowing directing effects and, second, the vinyl fragment was disubstituted and part of a 6‐membered heterocycle. Furthermore, to the best of our knowledge, regioselective glucal arylations have only been achieved with organometallic reagents until now,^[15]^ whereas direct metal‐catalyzed reactions of this type have remained unstudied. To our disappointment, the previously devised α‐arylation protocol [with phenyl triflate (**2 a**) as aryl source as summarized in Scheme 3] proved ineffective, providing phenylated **11** in only 10 % yield (Scheme 6). However, an improvement was achieved by following the alternative approach [with phenyl iodide (**4 a**) as aryl source as summarized in Scheme 4]. Now, **11** was obtained in 73 % yield (with 74 % conversion of **10**). This result revealing a high α‐selectivity was unexpected because previous applications of this protocol had predominantly provided β‐arylated products. Assuming that this switch from β‐to α‐selectivity was linked to the disubstitution of the vinylic portion of **10**, the arylation behavior of compounds **12** and **13** was studied. While **12** proved inactive allowing no arylation with neither the α‐ nor the β‐selective protocol, **13** reacted well and, confirming our aforementioned hypothesis, *both* approaches provided α‐arylated product **14** in preference. In detail, under the conditions shown in Scheme 3 with phenyl triflate (**2 a**) as aryl source, **14** was obtained as the sole product in 69 % yield (after 24 h). Finally, applying the second palladium catalysis with phenyl iodide (**4 a**) as aryl source and using conditions summarized in Scheme 4 until complete conversion, a mixture of α‐arylated **14** and β‐arylated **15** resulted with the former product in a 68:32 preference. Hence, it seems that enol ethers with a 1,2‐disubstituted vinyl unit have a pronounced α‐arylation selectivity, which we have not been able to override by conditional changes until now.

In order to shed light on the key factors determining the regioselectivity of the vinyl ether arylation, a DFT computational study, specifically focused on the vinyl insertion into the Pd−Ph bond and its regioselectivity, was carried out (see Supplementary Information for computational details). As noted before, the regioselectivity is highly sensitive towards several factors in the reaction mixture, including the palladium source, the ligand (or its absence), the base, and the aryl (pseudo)halide. For favoring the α‐selectivity, the presence of dppp as ligand and the use of an aryl triflate were key. As previously observed by Jutand and co‐workers in stoichiometric reactions,^[16]^ dppp in combination with aryl triflates and Pd(OAc)_2_ favors the generation of cationic ArPd(dppp)]^+^ species. In contrast, aryl iodides provide mixtures of neutral and cationic species. In addition, the absence of ligand and the presence of chloride ions favor the vinyl insertion at the β‐position.

**Scheme 7:**
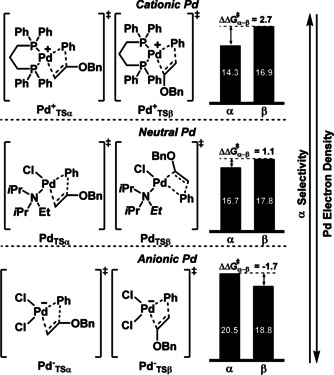
Free energy barriers of α‐ versus β‐alkene insertion into cationic, neutral and anionic Pd^II^ complexes as models for catalyst speciation under experimental conditions. Energies in kcal mol^−1^ referenced to the corresponding alkene‐L_*n*_Pd(II)‐Ph intermediate. For the neutral palladium complexes, both geometrical isomers were explored, and the most stable is shown here.

To further analyse this effect, the free energy activation barrier for vinyl insertion into the Pd−Ph bond using three different models was calculated: first, an electron‐deficient cationic (dppp)–Pd complex, second, a neutral Pd complex that might be formed from PdCl_2_ as precursor and DIPEA as additive, and finally third, an anionic electron‐rich Pd complex that would be favored under excess of Cl^−^ ions (Scheme 7). Although many other species could co‐exist in solution, we aimed to identify a trend that correlated the electron density of the palladium center with the competition between the α and β vinyl positions. Interestingly, the data showed that a cationic Pd complex (Scheme 7, top) strongly favored the functionalization at the α position, in very good agreement with experimental observation (ΔΔ*G* ^≠^_α‐*β*_=2.7). On the contrary, an anionic palladium complex (Scheme 7, bottom) supported the functionalization at the β position (ΔΔ*G* ^≠^_α‐*β*_=−1.7). Finally, the neutral palladium complex was located in between of the other two species, slightly favoring the α‐position by 1.1 kcal mol^−1^. These results can also explain the difficulties in improving the β selectivity, as the absence of a ligand prevents the formation of well‐defined species, and consequently both neutral and anionic species can co‐exist in solution.

## Conclusion

In summary, we developed two protocols for α‐ and β‐selective arylations of benzyl vinyl ethers. Both approaches are catalytic Heck‐type cross‐couplings, where activity and the regioselectivity are determined by a number of factors including the palladium species, the presence or absence of a ligand and its specific type, the aryl source, additives, and fine details of the reaction conditions. In contrast to previous studies, an almost equimolar substrate ratio was sufficient for high product formation, and the regioselectivity of the arylation did not rely on chelating effects. A selective hydrolysis of the α‐arylated product over its β‐substituted analogue allows isolating the latter in case an unsatisfying α/β‐selectivity characterizes the coupling reactions. More structurally complex enol vinyl ethers can be applied but both protocols lead to an α‐arylation preference. However, direct α‐arylation of glycals developed here can potentially become a valuable tool in the synthesis of gliflozins medicines used to treat type II diabetes mellitus. A DFT computational study revealed the key factors leading to the pronounced regioselectivities, and the obtained results were in good agreement with the experimental observations.

## Conflict of interest

The authors declare no conflict of interest.

## Supporting information

As a service to our authors and readers, this journal provides supporting information supplied by the authors. Such materials are peer reviewed and may be re‐organized for online delivery, but are not copy‐edited or typeset. Technical support issues arising from supporting information (other than missing files) should be addressed to the authors.

Supporting Information
