# Supplementary material for: Selective Approaches to α‐ and β‐Arylated Vinyl Ethers
Source: Angew Chem Int Ed Engl. 2021 Nov 23;61(1):e202109801. doi: 10.1002/anie.202109801 (PMC9299197; doi:10.1002/anie.202109801)

Angewandte Chemie
Eine Zeitschrift der Gesellschaft Deutscher Chemiker

Supporting Information

Selective Approaches to α- and β-Arylated Vinyl Ethers

*Anna Domzalska-Pieczykolan, Ignacio Funes-Ardoiz, Bartłomiej Furman, and Carsten Bolm**

anie_202109801_sm_miscellaneous_information.pdf

Table of Content

1. Abbreviations S2
2. General S2
3. Instruments and methods S2
4. Optimization process tables S3
5. Characterizing data of starting materials **1** S5
6. General procedure for the α-arylation of benzyl-type vinyl ethers using ArOTf (GP1) S6
7. Characterizing data of products **6** S6
8. General procedure for the β-arylation of benzyl-type vinyl ethers using ArI (GP2) S9
9. Characterizing data of products **7** S9
10. General procedure for the selective hydrolysis of **6** in mixtures of **6** and **7** (GP3) S12
11. Characterizing data of products **7** (obtained by GP3 as single regioisomers) S12
12. Characterizing data of products **9** and **11** S13
13. Characterizing data of products **13** - **15** S13
14. Computational details and xyz coordinates and energies of calculated species S14
15. References S22
16. NMR spectra S22

1. Abbreviations

The following abbreviations are used throughout the text of the ESI file: AcOEt, ethyl acetate; APCI, atmospheric pressure chemical ionization; DCM, dichloromethane; DIPEA, N,N-diisopropylethylamin; DMF, N,N-dimethylformamide; DMSO, dimethylsulfoxide; dppb, 1,4-bis-(diphenylphosphino)butane; dppe, 1,2-bis(diphenylphosphino)ethane; dppf, 1,1'-ferrocenediyl-bis(diphenylphosphine); dppp, 1,3-bis(diphenylphosphino)propane; EI, electron ionization; ESI, electrospray ionization; HRMS, high-resolution mass spectrometry; MeCN, acetonitrile; MS, low-resolution mass spectrometry/spectrum; PEG400, polyethylene glycol 400; PhMe, toluene; rt, room temperature; TEA, triethylamine; THF, tetrahydrofuran; TLC, thin-layer chromatography; Xantphos, 4,5-bis(diphenylphosphino)-9,9-dimethylxanthene; Xphos, 2-dicyclohexylphosphino-2′,4′,6′-triisopropylbiphenyl.

2. General

All commercial materials (Sigma-Aldrich, Fluka, Fluorochem, etc.) were used without further purification. All solvents were reagent or HPLC (Honeywell) grade. Unless otherwise noted, all reactions were run under argon atmosphere in flame-dried glassware. Reactions were stirred using Teflon-coated magnetic stir bars. TLC was performed on aluminum sheets, Merck 60F with fluorescent indicator F254. Plates were visualized by treatment with UV or aqueous ceric ammonium molybdate (Hanessian's stain; CAM) with gentle heating. Products were purified by flash column chromatography using the solvent systems indicated. Column chromatography was performed on Merck silica gel 60, 230–400 mesh.

3. Instruments and Methods

All reported ^{1}H NMR spectra were recorded using Varian Mercury 300, Varian VNMRS 400, Varian VNMRS 600, Bruker Avance Neo 400 or Bruker Avance Neo 600 spectrometers at 25 °C, if not otherwise stated, and they were processed and analyzed with the program MestReNova. Chemical shifts are quoted on the (*δ* ppm) scale, multiplicity (s = singlet, bs = broad singlet, d = doublet, t = triplet, q = quartet, m = multiplet and combinations thereof), coupling constant in Hz, and integration. Solvent signal was indicated as the internal standard ($CDCl_3$, ^{1}H NMR 7.26 ppm, ^{13}C{^{1}H} NMR 77.00 ppm; C_6D_6, ^{1}H NMR 7.16 ppm, ^{13}C{^{1}H} NMR 128.06 ppm; DMSO-d_6 ^{1}H NMR 2.50 ppm, ^{13}C{^{1}H} NMR 39.40 ppm) unless otherwise noted, *J* values are given in Hz. IR spectra were recorded with a PerkinElmer Spectrum 100 FT-IR spectrometer with an UATR Diamond KRS-5 unit. The wavenumbers of the bands are given in cm^{-1}. High resolution mass spectra (HRMS) were obtained via electron ionization (EI-MS), electrospray ionization (ESI-MS) or atmospheric-pressure chemical ionization (APCI-MS). Mass spectra (HRMS) were recorded on a Thermo Scientific LTQ Orbitrap XL or Finnigan MAT 95 spectrometer. Melting points (m.p.) were determined on a Büchi B-540 melting point apparatus.

4. Optimization process tables

Table S1. Optimization of the α-arylation process using benzyl vinyl ether (**1a**) as substrate.

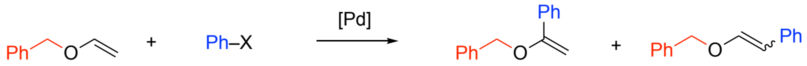

1a **2a-5a** **6a** **7a**

	**2a-5a** [equiv.]	palladium source [mol %]	ligand [mol %]	base [equiv.]	additive [mol %]	solvent	T [˚C]	t [h]	atm.	conv. of **1a** [%]	**6a**/**7a** [%]
1	PhBr (0.5)	$Pd(OAc)_2$ (3.0)	dppp (6.0)	K_2CO_3 (1.2)	H_2O (6.0)	DMF	80	20	argon	38*	100/0
2	PhBr (0.5)	$Pd(OAc)_2$ (3.0)	dppp (6.0)	K_2CO_3 (1.2)	H_2O (6.0)	DMF	80	20	air	94*	45/55
3	PhBr (1.0)	$Pd(OAc)_2$ (3.0)	dppp (6.0)	K_2CO_3 (1.2)	H_2O (6.0)	DMF	80	20	argon	35*	100/0
4	PhBr (1.0)	$Pd(OAc)_2$ (3.0)	dppp (6.0)	K_2CO_3 (1.2)	H_2O (6.0)	DMF	80	20	air	70*	39/61
5	PhBr (2.0)	$Pd(OAc)_2$ (6.0)	dppp (12.0)	K_2CO_3 (1.2)	H_2O (6.0)	DMF	80	21	argon	24*	100/0
6	PhBr (2.0)	$Pd(OAc)_2$ (6.0)	dppp (12.0)	K_2CO_3 (2.4)	H_2O (12.0)	DMF	80	19	argon	94*	100/0
7	PhBr (2.0)	$Pd(OAc)_2$ (6.0)	dppp (12.0)	K_2CO_3 (2.4)	-	DMF	80	21	argon	45	100/0
8	PhBr (2.0)	$Pd(OAc)_2$ (6.0)	dppp (12.0)	*t*BuOLi (1.2)	-	DMF	80	21	argon	16	100/0
9	PhBr (2.0)	$Pd(OAc)_2$ (6.0)	dppp (12.0)	*t*BuOK (1.2)	-	DMF	80	21	argon	6	100/0
10	PhBr (2.0)	$Pd(OAc)_2$ (6.0)	dppp (12.0)	KOH (1.2)	-	DMF	80	21	argon	54	100/0
11	PhBr (2.0)	$Pd(OAc)_2$ (6.0)	dppp (12.0)	TEA (1.2)	-	DMF	80	21	argon	42	100/0
12	PhBr (2.0)	$Pd(OAc)_2$ (6.0)	dppp (12.0)	DIPEA (1.2)	-	DMF	80	21	argon	59	100/0
13	PhBr (2.0)	$Pd(OAc)_2$ (6.0)	dppp (12.0)	DIPEA (1.2)	-	dioxane	80	20	argon	45	100/0
14	PhBr (2.0)	$Pd(OAc)_2$ (6.0)	dppp (12.0)	DIPEA (1.2)	-	PEG400	80	20	argon	63	100/0
15	PhBr (2.0)	$Pd(OAc)_2$ (6.0)	dppp (12.0)	DIPEA (1.2)	-	toluene	80	20	argon	40	100/0
16	PhBr (2.0)	$PdCl_2$ (6.0)	dppp (12.0)	DIPEA (1.2)	-	DMF	80	19	argon	Substrate recovery	
17	PhBr (2.0)	$Pd(dba)_2$ (6.0)	dppp (12.0)	DIPEA (1.2)	-	DMF	80	20	argon	Substrate recovery	
18	PhBr (2.0)	$Pd_2(dba)_3$ (6.0)	dppp (12.0)	DIPEA (1.2)	-	DMF	80	19	argon	3	53/47
19	PhBr (2.0)	$Pd(PPh_3)_4$ (6.0)	dppp (12.0)	DIPEA (1.2)	-	DMF	80	19	argon	Substrate recovery	
20	PhBr (2.0)	$Pd(OAc)_2$ (6.0)	-	DIPEA (2.4)	-	DMF	80	20	argon	8	33/67
21	PhBr (2.0)	$Pd(OAc)_2$ (6.0)	dppe (12.0)	DIPEA (1.2)	-	DMF	80	20	argon	2	100/0
22	PhBr (2.0)	$Pd(OAc)_2$ (6.0)	dppb (12.0)	DIPEA (1.2)	-	DMF	80	20	argon	41	61/39
23	PhBr (2.0)	$Pd(OAc)_2$ (6.0)	dppf (12.0)	DIPEA (1.2)	-	DMF	80	20	argon	83	63/37
24	PhBr (2.0)	$Pd(OAc)_2$ (6.0)	PPh_3 (12.0)	DIPEA (2.4)	-	DMF	80	19	argon	54	89/11
25	PhBr (2.0)	$Pd(OAc)_2$ (6.0)	Xphos (12.0)	DIPEA (2.4)	-	DMF	80	19	argon	85	34/66
26	PhBr (2.0)	$Pd(OAc)_2$ (6.0)	Xantphos (12.0)	DIPEA (2.4)	-	DMF	80	19	argon	72	38/62
27	PhBr (2.0)	$Pd(OAc)_2$ (6.0)	dppp (12.0)	DIPEA (1.2)	-	DMF	120	21	argon	79	98/2
28	PhBr (2.0)	$Pd(OAc)_2$ (6.0)	dppp (12.0)	DIPEA (1.2)	-	DMF	50	70	argon	13	100/0
29	PhI (1.1)	$Pd(OAc)_2$ (6.0)	dppp (12.0)	DIPEA (1.2)	-	DMF	80	20	argon	64	66/44
30	PhONf (1.1)	$Pd(OAc)_2$ (6.0)	dppp (12.0)	DIPEA (1.2)	-	DMF	80	20	argon	100	100/0
31	PhONf (1.1)	$Pd(OAc)_2$ (6.0)	dppp (12.0)	DIPEA (1.2)	-	DMF	80	2.5	argon	78	100/0
32	PhOTf (1.1)	$Pd(OAc)_2$ (6.0)	dppp (12.0)	DIPEA (1.2)	-	DMF	80	20	argon	100	100/0
33	PhOTf(1.1)	$Pd(OAc)_2$ (6.0)	dppp (12.0)	DIPEA (1.4)	-	DMF	80	2	argon	100	100/0
**34**	**PhOTf (1.1)**	**$Pd(OAc)_2$ (3.4)**	**dppp (6.8)**	**DIPEA (1.4)**	**-**	**DMF**	**80**	**2**	**argon**	**100**	**100/0**
35	PhOTf (1.1)	$Pd(OAc)_2$ (3.4)	dppp (6.8)	TEA (1.4)	-	DMF	80	2	argon	27	100/0
36	PhOTf (1.1)	$Pd(OAc)_2$ (3.4)	dppf (6.8)	DIPEA (1.4)	-	DMF	80	2	argon	100	100/0
37	PhOTf(1.1)	$Pd(OAc)_2$ (3.4)	PPh_3(6.8)	DIPEA (1.4)	-	DMF	80	2	argon	18	69/31
38	PhOTf (1.1)	$PdCl_2$ (3.4)	dppp (6.8)	DIPEA (1.4)	-	DMF	80	2	argon	8	100/0

* Water was added. Thus, the product mixture also contains hydrolysed enol ethers.

Table S2. Optimization of the β-arylation process using benzyl vinyl ether (**1a**) as substrate.

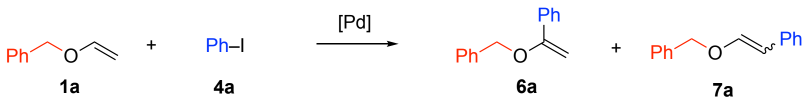

	PhI [equiv.]	palladium source [mol %]	ligand [mol %]	base [equiv.]	additive [equiv.]	solvent	T [°C]	t [h]	conv. of **1a** [%]	**6a/7a** [%]	*E/Z* of [**7a**]
1	2.0	$Pd(OAc)_2$ (6.0)	dppp (12)	DIPEA (1.2)	-	DMF	80	20	64	66/34	66/34
2	2.0	$Pd(OAc)_2$ (6.0)	-	DIPEA (1.2)	-	DMF	80	20	100	36/64	40/60
3	1.0	$Pd(OAc)_2$ (6.0)	-	DIPEA (1.2)	-	DMF	80	20	100	45/55	42/58
4	1.1	$PdSO_4$ (3.4)	-	DIPEA (2.0)	-	DMF	80	2.0	62	50/50	40/60
5	1.1	$Pd(CN)_2$ (3.4)	-	DIPEA (2.0)	-	DMF	80	2.0	77	47/53	43/57
6	1.1	$Pd(CN)_2$ (3.4)	-	DIPEA (2.0)	Bu_4NCl (1.0)	DMF	80	2.0	98	30/70	43/57
7	1.1	$Pd(dba)_2$ (3.4)	-	DIPEA (2.0)	-	DMF	80	2.0	47	45/55	47/53
8	1.1	$[PdCl(C_3H_5)]_2$ (3.4)	-	DIPEA (2.0)	-	DMF	80	2.0	6	35/65	53/47
9	1.1	Pd/C (3.4)	-	DIPEA (2.0)	-	DMF	80	2.0	50	45/55	43/57
10	1.1	$Pd(OAc)_2$ (3.4)	-	DIPEA (2.0)	LiCl (2.0)	DMF	80	2.0	96	29/71	37/63
11	1.1	$Pd(OAc)_2$ (3.4)	-	DIPEA (2.0)	Bu_4NCl (1.0)	DMF	80	2.0	100	29/71	52/48
12	1.1	$Pd(OAc)_2$ (3.4)	-	DIPEA (2.0)	Bu_4NCl (2.0)	DMF	80	2.0	100	25/75	49/51
13	1.1	$Pd(OAc)_2$ (3.4)	-	DIPEA (2.0)	LiCl/ Bu_4NCl (2.0./ 1.0)	DMF	80	2.0	100	26/74	38/62
14	1.1	$Pd(OAc)_2$ (3.4)	-	DIPEA (2.0)	LiOAc (1.0)	DMF	80	2.0	47	46/54	66/34
15	1.1	$Pd(OAc)_2$ (3.4)	-	DIPEA (2.0)	Bu_4NI (2.0)	DMF	80	2.0	76	42/52	41/59
16	1.1	$Pd(OAc)_2$ (3.4)	-	DIPEA (2.0)	Bu_4NHSO_4 (2.0)	DMF	80	2.0	79	54/46	39/61
17	1.1	$Pd(OAc)_2$ (3.4)	-	DIPEA (1.2)	Aliquat (1.2)	DMF	80	2.0	100	28/72	50/50
18	1.1	$PdCl_2$ (3.4)	-	DIPEA (2.0)	Bu_4NBr (1.0)	DMF	80	2.0	87	36/64	42/58
19	1.1	$PdCl_2$ (3.4)	-	DIPEA (2.0)	LiOAc (1.0)	DMF	80	2.0	77	44/56	57/43
20	1.1	$PdCl_2$ (3.4)	-	DIPEA (2.0)	LiCl (1.0)	DMF	80	2.0	95	33/67	40/60
**21**	**1.1**	**$PdCl_2$ (3.4)**	**-**	**DIPEA (2.0)**	**Bu_4NCl (1.0)**	**DMF**	**80**	**3.0**	**100**	**27/73**	**38/62**
22	1.1	$PdCl_2$ (3.4)	-	DIPEA (2.0)	Bu_4NCl (2.0)	DMF	80	2.0	100	28/72	43/57
23	1.1	$PdCl_2$ (3.4)	-	DIPEA (1.2)	Bu_4NCl (2.0)	DMF	80	2.0	100	23/77	41/59
24	1.1	$PdCl_2$ (3.4)	-	K_2CO_3 (1.2)	Bu_4NCl (2.0)	DMF	80	2.0	100	31/69	73/27
25	1.1	$PdCl_2$ (3.4)	-	NaOAc (2.0)	Bu_4NCl (1.0)	DMF	80	2.0	94	31/69	79/21
26	1.1	$PdCl_2$ (3.4)	-	DIPEA (2.0)	Bu_4NCl (1.0)	toluene	80	2.0	91	28/72	33/67
27	1.1	$PdCl_2$ (3.4)	-	DIPEA (2.0)	Bu_4NCl (1.0)	oktafluoro toluene	80	3.0	13	32/68	36/64
28	1.1	$PdCl_2$ (3.4)	-	DIPEA (2.0)	Bu_4NCl (1.0)	DCE	50	2.0	20	22/78	42/58
29	1.1	$PdCl_2$ (3.4)	-	DIPEA (2.0)	Bu_4NCl (1.0)	DCE	50	24	83	26/74	34/66
30	1.1	$PdCl_2$ (3.4)	-	DIPEA (2.0)	Bu_4NCl (1.0)	THF	50	24	100	24/76	**27/73**
31	1.1	$PdCl_2$ (3.4)	-	DIPEA (2)	Bu_4NCl (1.0)	MeCN	50	2.0	6	67/33	44/56

Table S3. Inprovement of the β-arylation process by variation of the reagent amounts with benzyl vinyl ether (**1a**) and phenyl iodide (**4a**) as substrates.

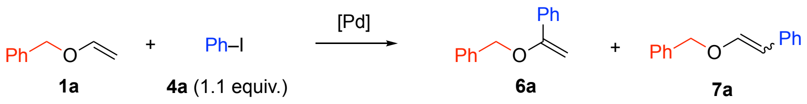

	coupling partner	palladium source [mol %]	base (equiv.)	additive (equiv.)	solvent	t [h]	conv. of **1a** [%]	yield of **6a/7a** [%]	ratio of **6a/7a** [%]	*E/Z* of [**7a**]
1	PhI	$Pd(OAc)_2$ (6.0)	DIPEA (1.2)	Bu_4NCl (2.0)	DMF	2.0	100	80	22/78	52/48
2	PhI	$Pd(OAc)_2$ (3.4)	DIPEA (2.0)	Bu_4NCl (2.0)	DMF	2.5	100	87	25/75	49/51
3	PhI	$Pd(OAc)_2$ (3.4)	DIPEA (2.0)	Bu_4NCl (1.0)	DMF	2.5	100	87	29/71	52/48
4	PhI	$PdCl_2$ (6.0)	DIPEA (1.2)	Bu_4NCl (2.0)	THF	overnight	100	81	21/79	30/70
**5**	**PhI**	**$PdCl_2$ (6.0)**	**DIPEA (1.2)**	**Bu_4NCl (2.0)**	**DMF**	**2.0**	**96**	**83**	**19/81**	**44/56**
6	PhI	$PdCl_2$ (6.0)	DIPEA (1.2)	-	DMF	2.0	53	46	44/56	44/56
7	PhI	$PdCl_2$ (3.4)	DIPEA (2.0)	-	DMF	2.0	47	42	45/55	44/56
8	PhI	$PdCl_2$ (3.4)	DIPEA (2.0)	Bu_4NCl (1.0)	DMF	2.0	98	91	29/71	43/57
9	PhI	$PdCl_2$ (3.4)	DIPEA (2.0)	Bu_4NCl (2.0)	DMF	2.0	100	94	28/72	43/57
**10**	**PhI**	**$PdCl_2$ (3.4)**	**DIPEA (1.2)**	**Bu_4NCl (2.0)**	**DMF**	**2.0**	**100**	**80**	**23/77**	**41/59**
**11**	**PhI**	**$PdCl_2$ (3.4)**	**DIPEA (1.2)**	**Bu_4NCl (2.0)**	**PhMe**	**3.0**	**33**	**23**	**20/80**	**39/61**
12	PhI	$PdCl_2$ (3.4)	DIPEA (1.2)	Bu_4NCl (2.0)	THF	overnight	65	58	21/79	30/70
13	PhBr	$PdCl_2$ (3.4)	DIPEA (2.0)	Bu_4NCl (1.0)	DMF	overnight	-	12	20/80	54/46
14	PhI	$PdCl_2$ (3.4)	K_2CO_3 (0.8)	Bu_4NCl (2.0)	DMF	2.0	90	65	26/74	52/48
**15**	**PhI**	**$PdCl_2$ (3.4)**	**K_2CO_3 (1.2)**	**Bu_4NCl (2.0)**	**DMF**	**2.0**	**-**	**79**	**31/69**	**73/27**
16	PhI	$PdCl_2$ (3.4)	DIPEA (2.0)	LiCl (1.0)	DMF	2.0	95	-	33/67	40/60
17[a]	PhI	$PdCl_2$ (3.4)	DIPEA (2.0)	-	DMF	2.0	58	-	31/69	36/64
18[a]	PhI	$Pd(OAc)_2$ (3.4)	DIPEA (2.0)	-	DMF	2.0	41	-	31/69	44/56
19	PhI	$PdCl_2$ (1.0)	DIPEA (1.2)	Bu_4NCl (2.0)	DMF	2.0	60	58	25/75	44/56
20	PhI	$PdCl_2$ (12.0)	DIPEA (1.2)	Bu_4NCl (2.0)	DMF	2.0	-	73	20/80	46/54

[a] Use of diphenylmethyl vinyl ether (**1b**) instead of **1a**.

5. Charactarizing data of starting materials 1

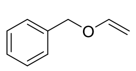

Benzyl vinyl ether (1a): Synthesized using a literature procedure[S1] starting from 7.8 g (122.1 mmol) of calcium carbide and 4.0 mL (39 mmol) of benzyl alcohol. The product was isolated by flash column chromatography on silica gel (eluting with pentane with 1% v/v TEA) to give 3.99 g (29.7 mmol, 77%) of **1a** as colourless liquid. **^{1}H NMR** (600 MHz, $CDCl_3$): δ = 7.43 – 7.37 (m, 4H), 7.37 – 7.32 (m, 1H), 6.60 (dd, *J*=14.3, 6.7 Hz, 1H), 4.79 (s, 2H), 4.34 (dt, *J*=14.3, 1.7 Hz, 1H), 4.12 (dt, *J*=6.7, 1.7 Hz, 1H); **^{13}C{^{1}H} NMR** (151 MHz, $CDCl_3$): δ = 151.8, 137.0, 128.6, 128.0, 127.7, 87.5, 70.2. The spectroscopic data are in agreement with the previously reported ones.[S2,S3]

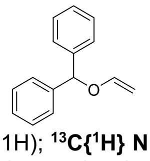

Diphenylmethyl vinyl ether (1b): Synthesized using a literature procedure[S1] starting from 5.2 g (81.1 mmol) of calcium carbide and 4.8 g (26 mmol) of diphenylmethanol. The product was isolated by flash column chromatography on silica gel (eluting with pentane with 1% v/v TEA) to give 4.75 g (22.6 mmol, 87%) of **1b** as colourless liquid. **^{1}H NMR** (600 MHz, C_6D_6): δ = 7.27 (d, *J*=7.4 Hz, 4H), 7.09 (t, *J*=7.7 Hz, 4H), 7.01 (t, *J*=7.4 Hz, 2H), 6.35 (dd, *J*=14.1, 6.6 Hz, 1H), 5.63 (s, 1H), 4.45 (dd, *J*=14.1, 1.6 Hz, 1H), 4.01 (dd, *J*=6.6, 1.6 Hz, 1H); **^{13}C{^{1}H} NMR** (151 MHz, C_6D_6): δ = 151.0, 141.8, 128.7, 127.8, 127.3, 90.2, 83.1. The spectroscopic data are in agreement with the previously reported ones.[S4,S5]

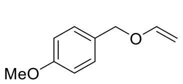

1-[(Ethenoxy)methyl]-4-methoxybenzene (1c): Synthesized using a literature procedure[S1] starting from 2.56 g (40 mmol) of calcium carbide and 1.6 mL (13 mmol) of 4-methoxybenzyl alcohol. The product was isolated by flash column chromatography on silica gel (eluting with pentane:AcOEt 99:1 with 1% v/v TEA) to give 1.54 g (9.4 mmol, 72%) of **1c** as colourless liquid. **^{1}H NMR** (400 MHz, C_6D_6): δ = 7.11 (d, *J*=8.5 Hz, 2H), 6.74 (d, *J*=8.6 Hz, 2H), 6.48 (dd, *J*=14.3, 6.8 Hz, 1H), 4.45 (s, 2H), 4.27 (dd, *J*=14.3, 1.8 Hz, 1H), 4.01 (dd, *J*=6.8, 1.8 Hz, 1H), 3.28 (s, 3H); **^{13}C{^{1}H} NMR** (101 MHz, C_6D_6): δ = 159.9, 152.1, 129.5, 114.2, 87.2, 70.0, 54.8. The spectroscopic data are in agreement with the previously reported ones.[S6]

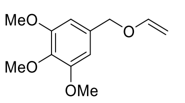

1-[(Ethenoxy)methyl]-3,4,5-trimethoxybenzene (1d): Synthesized using a literature using literature procedure[S1] starting from 2.6 g (40.5 mmol) of calcium carbide and 2.1 mL (13 mmol) of 3,4,5-trimethoxybenzyl alcohol. The product was isolated by flash column chromatography on silica gel (eluting with pentane:AcOEt 98:2 with 0.5% v/v TEA followed by pentane:AcOEt 9:1 with 0.5% v/v TEA) to give 1.44 g (6.5 mmol, 50%) of **1d** as slightly yellow liquid. **^{1}H NMR** (400 MHz, $CDCl_3$): δ = 7.28 – 7.20 (m, 3H), 5.36 (s, 2H), 4.98 (dd, *J*=14.3, 2.1 Hz, 1H), 4.77 (dd, *J*=6.8, 2.1 Hz, 1H), 4.54 (s, 6H), 4.51 (s, 3H); **^{13}C{^{1}H} NMR** (101 MHz, $CDCl_3$): δ = 153.4, 151.6, 137.7, 132.5, 104.7, 87.5, 70.3, 60.9, 56.2; **IR** (film): $\tilde{v}$=2938, 2836, 1592, 1505, 1458, 1421, 1373, 1331, 1236, 1190, 1123, 1005, 962, 822 782, 685 cm^{-1}; **HRMS** (ESI): *m/z* calcd for $C_{12}H_{16}O_4$+Na^+: 247.0941 [M+Na]$^+$; found: 247.0935.

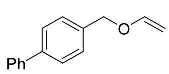

1-[(Ethenoxy)methyl]-4-phenylbenzene trimethoxybenzene (1e): Synthesized using a literature procedure[S1] starting from 5.1 g (80 mmol) of calcium carbide and 4.8 g (26 mmol) of biphenyl-4-methanol. The product was isolated by flash column chromatography on silica gel (eluting with pentane with 1% v/v TEA followed by pentane:AcOEt 98:2 with 1% v/v TEA) to give 4.98 g (23.7 mmol, 91%) of **1e** as white solid. **m.p.** 57.5°C; **^{1}H NMR** (400 MHz, C_6D_6): δ = 7.48 – 7.37 (m, 4H), 7.26 – 7.06 (m, 9H), 6.49 (dd, *J*=14.3, 6.8 Hz, 1H), 4.50 (s, 2H), 4.27 (dd, *J*=14.3, 1.9 Hz, 1H), 4.02 (dd, *J*=6.8, 1.9 Hz, 1H); **^{13}C{^{1}H} NMR** (101 MHz, C_6D_6): δ = 152.0, 141.3, 141.2, 136.5, 129.1, 128.2, 127.5, 127.5, 87.4, 69.9; **IR** (film): $\tilde{v}$=3116, 3033, 2914, 2866, 1610, 1485,1458, 1403, 1379, 1317, 1200, 989, 961, 819, 758, 695 cm^{-1}; **HRMS** (ESI): *m/z* calcd for $C_{15}H_{14}O$+Na^+: 233.0937 [M+Na]$^+$; found: 233.0935.

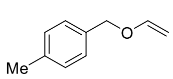

1-[(Ethenoxy)methyl]-4-methylbenzene (1f): Synthesized using a literature procedure[S1] starting from 5.2 g (81.1 mmol) of calcium carbide and 3.2 g (26 mmol) of 4-methylbenzyl alcohol. The product was isolated by flash column chromatography on silica gel (eluting with pentane with 1% v/v TEA) to give 2.60 g (17.6 mmol, 67%) of **1f** as colourless liquid. **^{1}H NMR** (400 MHz, C_6D_6): δ = 7.12 (d, *J*=7.9 Hz, 2H), 6.96 (d, *J*=7.9 Hz, 2H), 6.47 (dd, *J*=14.3, 6.8 Hz, 1H), 4.48 (s, 2H), 4.25 (dd, *J*=14.3, 1.9 Hz, 1H), 3.99 (dd, *J*=6.8, 1.9 Hz, 1H), 2.08 (s, 3H); **^{13}C{^{1}H} NMR** (101 MHz, C_6D_6): δ = 152.1, 137.5, 134.6, 129.3, 127.9, 87.3, 70.1, 21.1. The spectroscopic data are in agreement with the previously reported ones.[S6]

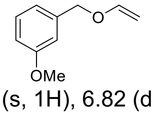

1-[(Ethenoxy)methyl]-3-methoxybenzene (1g): Synthesized using a literature procedure[S1] starting from 5.1 g (80 mmol) of calcium carbide and 3.2 mL (26 mmol) of 3-methoxybenzyl alcohol. The product was isolated by flash column chromatography on silica gel (eluting with pentane:AcOEt 99:1 with 1% v/v TEA) to give 3.96 g (24.1 mmol, 93%) of **1g** as slightly yellow liquid. **^{1}H NMR** (400 MHz, C_6D_6): δ = 7.07 (t, *J*=8.0 Hz, 1H), 6.90 (s, 1H), 6.82 (d, *J*=7.5 Hz, 1H), 6.72 (dd, *J*=8.0, 2.3 Hz, 1H), 6.45 (dd, *J*=14.3, 6.8 Hz, 1H), 4.47 (s, 2H), 4.24 (dd, *J*=14.3, 1.9 Hz, 1H), 3.99 (dd, *J*=6.8, 1.9 Hz, 1H), 3.30 (s, 3H); **^{13}C{^{1}H} NMR** (101 MHz, C_6D_6): δ = 160.0, 151.6, 138.7, 129.3, 119.5, 113.4, 112.8, 87.1, 69.7, 54.4; **IR** (film): $\tilde{v}$= 3115, 3003, 2940, 2837, 1605, 1490, 1460, 1370, 1318, 1265, 1192, 1155, 1047, 996, 958, 820, 780, 745, 691 cm^{-1}; **HRMS** (ESI): *m/z* calcd for $C_{10}H_{12}O_2$+Na^+: 187.0729 [M+Na]$^+$; found: 187.0725.

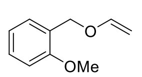

1-[(Ethenoxy)methyl]-2-methoxybenzene (1h): Synthesized using a literature procedure[S1] starting from 2.6 g (40.5 mmol) of calcium carbide and 2.1 mL (13 mmol) of 2-methoxybenzyl alcohol. The product was isolated by flash column chromatography on silica gel (eluting with pentane:AcOEt 99:1 with 1% v/v TEA) to give 1.70 g (10.4 mmol, 80%) of **1h** as orange liquid.^{1}**H NMR** (600 MHz, $CDCl_3$): *δ* = 7.40 (d, *J*=7.5, 1H), 7.31 (t, *J*=8.3 Hz, 1H), 6.99 (t, *J*=7.5 Hz, 1H), 6.90 (d, *J*=8.3 Hz, 1H), 6.60 (dd, *J*=14.3, 6.7 Hz, 1H), 4.84 (s, 2H), 4.35 (dd, *J*=14.3, 1.5 Hz, 1H), 4.09 (dd, *J*=6.7, 1.5 Hz, 1H), 3.86 (s, 3H); ^{13}**C{^{1}H} NMR** (151 MHz, $CDCl_3$): *δ* = 157.1, 152.0, 129.1, 129.0, 125.3, 120.6, 110.4, 87.2, 65.4, 55.5; **IR** (film): $\tilde{\nu}$= 3116, 3043, 3004, 2940, 2838, 2325, 2113, 1988, 1907, 1611, 1494, 1461, 1371, 1318, 1287, 1244, 1195, 1118, 1029, 957, 819, 752 cm^{-1}; **HRMS** (ESI): *m/z* calcd for $C_{10}H_{12}O_2$+Na^+: 187.0730 [M+Na]$^+$; found: 187.0726.

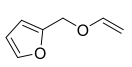

2-[(Vinyloxy)methyl]furan (1i): Synthesized using a literature procedure[S1] starting from 5.6 g (87.3 mmol) of calcium carbide and 2.55 g (26 mmol) of furfuryl alcohol. The product was isolated by flash column chromatography on silica gel (eluting with pentane with 0.5% v/v TEA) to give 435.5 mg (3.5 mmol, 27%) of **1i** as yellow liquid. ^{1}**H NMR** (400 MHz, $CDCl_3$): *δ* = 7.06 – 7.01 (m, 1H), 6.36 (dd, *J*=14.3, 6.8 Hz, 1H), 6.09 – 5.92 (m, 2H), 4.36 (s, 2H), 4.21 (dd, *J*=14.3, 2.0 Hz, 1H), 3.94 (dd, *J*=6.8, 2.1 Hz, 1H); ^{13}**C{^{1}H} NMR** (101 MHz, $CDCl_3$): *δ* = 151.6, 151.0, 143.0, 110.6, 109.8, 87.5, 62.5. The spectroscopic data are in agreement with the previously reported ones.[S6–S8]

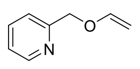

2-[(Vinyloxy)methyl]pyridine (1j): Synthesized using a literature procedure[S1] starting from 5.2 g (81.1 mmol) of calcium carbide and 2.8 g (26 mmol) of 2-pyridinylmethanol. The product was isolated by flash column chromatography on silica gel (eluting with pentane:DCM with 1.5% v/v TEA) to give 3.21 g (23.8 mmol, 92%) of **1j** as brown liquid. ^{1}**H NMR** (600 MHz, $CDCl_3$): *δ* = 8.56 (m, 1H), 7.70 (m, 1H), 7.42 (m, 1H), 7.22 – 7.17 (m, 1H), 6.59 (dd, *J*=14.3, 6.8 Hz, 1H), 4.89 (s, 2H), 4.31 (dd, *J*=14.3, 2.1 Hz, 1H), 4.11 (dd, *J*=6.8, 2.1 Hz, 1H); ^{13}**C{^{1}H} NMR** (151 MHz, $CDCl_3$): *δ* = 157.2, 151.4, 151.4, 149.3, 136.8, 122.7, 121.3, 88.0, 70.7; **IR** (film): $\tilde{\nu}$= 3414, 3116, 3060, 3016, 2927, 2870, 2326, 2086, 1911, 1619, 1591, 1475, 1436, 1366, 1321, 1194, 1073, 997, 956, 824, 756 cm^{-1}; **HRMS** (ESI): *m/z* calcd for C_8H_9NO+H^+: 136.0757 [M+H]$^+$; found: 136.0755.

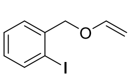

1-[(Ethenoxy)methyl]-2-iodobenzene (1k): Synthesized using a literature procedure[S1] starting from 1 g (15.6 mmol) of calcium carbide and 1.2 g (5 mmol) of 2-iodobenzyl alcohol. The product was isolated by flash column chromatography on silica gel (eluting with pentane with 1% v/v TEA) to give 183.8 mg (0.7 mmol, 14%) of **1k** as colourless oil. ^{1}**H NMR** (600 MHz, $CDCl_3$): *δ* = 7.62 – 7.53 (m, 1H), 7.28 – 7.24 (m, 1H), 6.98 – 6.88 (m, 1H), 6.56 – 6.47 (m, 1H), 6.37 (dd, *J*=14.3, 6.8 Hz, 1H), 4.56 (s, 2H), 4.26 (dd, *J*=14.3, 2.0 Hz, 2H), 3.98 (dt, *J*=6.8, 2.0 Hz, 2H); ^{13}**C{^{1}H} NMR** (151 MHz, $CDCl_3$): *δ* = 151.6, 139.4, 129.4, 128.8, 128.3, 97.5, 87.9, 74.1; **IR** (film): $\tilde{\nu}$=3057, 2922, 2325, 2098, 1916, 1635, 1442, 1370, 1319, 1194, 1149, 1010, 956, 930, 819, 747, 693 cm^{-1}; **MS** (EI) *m/z* (%): 216.9 (100), 209.1 (15), 91.1 (9).

6. General procedure for the α-arylation of benzyl-type vinyl ethers using ArOTf (GP1)

In the flame-dried, argon-filled Schlenk flask, $Pd(OAc)_2$ (7.6 mg, 0.034 mmol, 3.4 mol %) and dppp (28 mg, 0.068 mmol, 6.8 mol %) were placed. The reagents were suspended in freshly degassed DMF (2.5 mL) and then, DIPEA (245 μL, 1.4 mmol), ArOTf (**2**, 1.1 mmol) and vinyl ether **1** (1.0 mmol) were added. Subsequently, the reaction mixture was degassed, positioned into the preheated oil bath (80 ˚C), and stirred (610 rpm) at this temperature for 2-24 h. In less than 1 h as cholor change occurred from slightly yellow to deep dark red. After that time, the reaction mixture was cooled to room temperature and dilluted by Et_2O (5 mL) followed by addition of NaOH (10 mL of a 10% aq. solution). After the phase separation, the organic phase was washed one time with NaOH (10 mL of a 10% aq. solution). Then, the collected water phases were one time extracted with Et_2O (10 mL). Finally, the combined organic phases were dried over Na_2SO_4, filtered and concentrated in vacuum. The product was isolated by flash column chromatography (20 mL silica in pentane with 1% v/v TEA) using pentane with 1% v/v TEA as eluent or pentane + 1% v/v TEA with AcOEt as additive for more polar derivatives (as marked below).

Note: The reaction flask must not be opened while the reaction is running. Even small amounts of air can affect the regioselectivity of the process.

7. Characterizing data of products 6

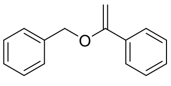

α-(Benzyloxy)styrene (6a): The product was synthesized following GP1 starting from 134.2 mg (1 mmol) of benzyl vinyl ether (**1a**) and 180 μL (1.1 mmol) of PhOTf (**2a**). Flash column chromatography on silica gel (eluting with pentane with 1% v/v TEA) led to 173.3 mg (0.82 mmol, 82%) of **6a** as yellow oil. ^{1}**H NMR** (600 MHz, C_6D_6): *δ* = 7.77 – 7.72 (m, 2H), 7.27 – 7.23 (m, 2H), 7.18 – 7.07 (m, 5H), 4.74 (d, *J*=2.9 Hz, 1H), 4.67 (s, 2H), 4.19 (d, *J*=2.9 Hz, 1H); ^{13}**C{^{1}H} NMR** (151 MHz, C_6D_6): *δ* = 160.2, 137.6, 137.0, 128.8, 128.7, 128.5, 128.0, 127.8, 125.9, 83.3, 70.0. The spectroscopic data are in agreement with the previously reported ones.[S9,S10]

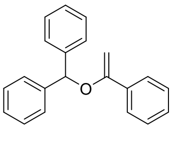

Diphenylmethyl 1-phenyl vinyl ether (6b): The product was synthesized following GP1 starting from 210.1 mg (1 mmol) of diphenylmethyl vinyl ether (**1b**) and 180 μL (1.1 mmol) of PhOTf (**2a**). Flash column chromatography on silica gel (eluting with pentane:AcOEt 99:1 with 1% v/v TEA) led to 267.0 mg (0.93 mmol, 93%) of **6b** as slightly yellow solid. **m.p.** 64.5 °C; ^{1}**H NMR** (600 MHz, C_6D_6): *δ* = 7.88 – 7.62 (m, 2H), 7.49 – 7.24 (m, 3H), 7.20 – 7.06 (m, 8H), 7.05 – 6.99 (m, 2H), 6.08 (s, 1H), 4.74 (d, *J*=2.9 Hz, 1H), 4.26 (d, *J*=2.9 Hz, 1H); ^{13}**C{^{1}H} NMR**

(151 MHz, C_6D_6): δ = 158.8, 142.0, 137.1, 128.8, 128.8, 128.5, 128.3, 127.8, 127.1, 126.0, 86.4, 81.7; **IR** (film): $\tilde{\nu}$= 3058, 1660, 1620, 1575, 1492, 1446, 1391, 1325, 1277, 1181, 1114, 1077, 1022, 959, 922, 818, 765, 742, 694 cm^{-1}; **HRMS** (ESI): *m/z* calcd for $C_{21}H_{18}O+Na^+$: 309.1250 [M+Na]$^+$; found: 309.1244.

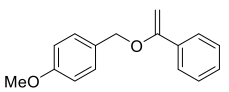

***p*-Methoxybenzyl 1-phenyl vinyl ether (6c):** The product was synthesized following GP1 starting from 164.1 mg (1 mmol) of 1-[(ethenoxy)methyl]-4-methoxybenzene (**1c**) and 180 µL (1.1 mmol) of PhOTf (**2a**). Flash column chromatography on silica gel (eluting with pentane with 0.5% v/v AcOEt and 1% v/v TEA) led to 230.5 mg (0.96 mmol, 96%) of **6c** as orange solid. **m.p.** 58.2 °C; **^{1}H NMR** (400 MHz, C_6D_6): δ = 7.81 – 7.72 (m, 2H), 7.22 – 7.07 (m, 7H), 6.82 – 6.74 (m, 1H), 4.76 (d, *J*=2.7 Hz, 1H), 4.66 (s, 2H), 4.24 (d, *J*=2.7 Hz, 1H), 3.30 (s, 3H); **^{13}C{^{1}H} NMR** (101 MHz, C_6D_6): δ = 160.4, 160.0, 137.1, 129.5, 128.7, 128.4, 128.3, 125.9, 114.2, 83.1, 69.9, 54.8. The spectroscopic data are in agreement with the previously reported ones.[S6]

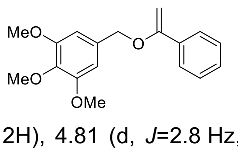

1,2,3-Trimethoxy-5-{[(1-phenylvinyl)oxy]methyl}benzene (6d): The product was synthesized following GP1 starting from 224.1 mg (1 mmol) of 1-[(ethenoxy)methyl]-3,4,5-trimethoxybenzene (**1d**) and 180 µL (1.1 mmol) of PhOTf (**2a**). Flash column chromatography on silica gel (eluting with pentane:AcOEt 95:5 with1% v/v DCM and 1% v/v TEA) led to 285.1 mg (0.95 mmol, 95%) of **6d** as slightly yellow solid. **m.p.** 69.5 °C; **^{1}H NMR** (600 MHz, C_6D_6): δ = 7.82 – 7.75 (m, 3H), 7.19 – 7.13 (m, 1H), 7.13 – 7.08 (m, 1H), 6.54 (s, 2H), 4.81 (d, *J*=2.8 Hz, 1H), 4.70 (s, 2H), 4.30 (d, *J*=2.8 Hz, 1H), 3.85 (s, 3H), 3.39 (s, 6H); **^{13}C{^{1}H} NMR** (151 MHz, C_6D_6): δ = 160.4,154.3, 139.2, 137.0, 132.7, 128.9, 128.5, 125.9, 105.8, 83.3, 70.6, 60.5, 55.9; **IR** (film): $\tilde{\nu}$= 3069, 3006, 2936, 2835, 1592, 1504, 1455, 1417, 1391, 1328, 1283, 1237, 1122, 1029, 1001, 949, 918, 822, 770, 685 cm^{-1}; **HRMS** (ESI): *m/z* calcd for $C_{18}H_{20}O_4+Na^+$: 323.1254 [M+Na]$^+$; found: 323.1249.

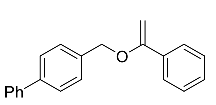

4-{[(1-Phenylethenyl)oxy]methyl}biphenyl (6e): The product was synthesized following GP1 starting from 210.3 mg (1 mmol) of 1-[(ethenoxy)methyl]-4-phenylbenzene (**1e**) and 180 µL (1.1 mmol) of PhOTf (**2a**). Flash column chromatography on silica gel (eluting with pentane:AcOEt 99:1 with 1% v/v TEA) led to 274.7 mg) 0.96 mmol, 96%) of **6e** as yellow solid. **m.p.** 38.8 °C; **^{1}H NMR** (400 MHz, C_6D_6): δ = 7.81 – 7.74 (m, 2H), 7.51 – 7.42 (m, 4H), 7.32 – 7.26 (m, 2H), 7.26 – 7.18 (m, 2H), 7.20 – 7.06 (m, 2H), 4.77 (d, *J*=2.8 Hz, 1H), 4.72 (s, 2H), 4.23 (d, *J*=2.8 Hz, 1H); **^{13}C{^{1}H} NMR** (101 MHz, C_6D_6): δ = 160.3, 141.4, 141.2, 137.0, 136.6, 129.1, 128.8, 128.5, 128.4, 127.6, 127.5, 125.9, 83.3, 69.8; **IR** (film): $\tilde{\nu}$= 3032, 2963, 2898, 2862, 1644, 1602, 1571, 1488, 1452, 1382, 1281, 1122, 1077, 1042, 951, 919, 807, 761, 694 cm^{-1}; **HRMS** (ESI): *m/z* calcd for $C_{21}H_{18}O+Na^+$: 309.1236 [M+Na]$^+$; found:309.1240.

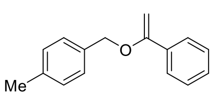

***p*-Methylbenzyl 1-phenyl vinyl ether (6f):** The product was synthesized following GP1 starting from 148.2 mg (1 mmol) of 1-[(ethenoxy)methyl]-4-methylbenzene (**1f**) and 180 µL (1.1 mmol) of PhOTf (**2a**). Flash column chromatography on silica gel (eluting with pentane with 1% v/v TEA) led to 214.7 mg (0.96 mmol, 96%) of **6f** as white solid. **m.p.** 48.9 °C; **^{1}H NMR** (400 MHz, C_6D_6): δ = 7.80 – 7.71 (m, 2H), 7.24 – 7.04 (m, 5H), 7.03 – 6.97 (m, 2H), 4.75 (d, *J*=2.7 Hz, 1H), 4.69 (s, 2H), 4.22 (d, *J*=2.7 Hz, 1H), 2.11 (s, 3H); **^{13}C{^{1}H} NMR** (101 MHz, C_6D_6): δ = 160.3, 137.5, 137.1, 134.7, 129.4, 128.7, 128.4, 128.0, 125.9, 83.2, 70.0, 21.2; **IR** (film): $\tilde{\nu}$=3055, 2917, 2858, 1643, 1602, 1571, 1516, 1492, 1447, 1383, 1282, 1216, 1121, 1077, 1029, 955, 804, 766, 687 cm^1; **HRMS** (ESI): *m/z* calcd for $C_{16}H_{16}O+Na^+$: 247.1080 [M+Na]$^+$; found: 247.1086.

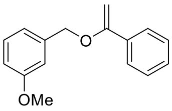

***m*-Methoxybenzyl 1-phenyl vinyl ether (6g):** The product was synthesized following GP1 starting from 164.1 mg (1 mmol) of 1-[(ethenoxy)methyl]-3-methoxybenzene (**1g**) and 180 µL (1.1 mmol) of PhOTf (**2a**). Flash column chromatography on silica gel (eluting with pentane with 1% v/v AcOEt and 1% v/v TEA) led to 228.1 mg (0.95 mmol, 95%) of **6g** as yellow solid. **m.p.** 39.6 °C; **^{1}H NMR** (400 MHz, C_6D_6): δ = 7.79 – 7.71 (m, 2H), 7.19 – 7.06 (m, 4H), 6.98 (s, 1H), 6.95 – 6.90 (m, 1H), 6.79 – 6.71 (m, 1H), 4.74 (d, *J*=2.8 Hz, 1H), 4.69 (s, 2H), 4.20 (d, *J*=2.8 Hz, 1H), 3.31 (s, 3H); **^{13}C{^{1}H} NMR** (101 MHz, C_6D_6): δ = 160.4, 160.2, 139.2, 137.0, 129.8, 128.7, 128.5, 125.9, 120.0, 113.7, 113.4, 83.4, 70.0, 54.8; **IR** (film): $\tilde{\nu}$= 3054, 2997, 2937, 2839, 1647, 1596, 1490, 1459, 1433, 1386, 1267, 1190, 1161, 1119, 1077, 1026, 950, 918, 857, 801, 768, 742, 691 cm^{-1}; **HRMS** (ESI): *m/z* calcd for $C_{16}H_{16}O_2+Na^+$: 263.1029 [M+Na]$^+$; found: 263.1034.

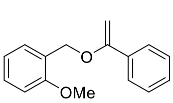

***o*-Methoxybenzyl 1-phenyl vinyl ether (6h):** The product was synthesized following GP1 starting from 164.1 mg (1 mmol) of 1-[(ethenoxy)methyl]-2-methoxybenzene (**1h**) and 180 µL (1.1 mmol) of PhOTf (**2a**). Flash column chromatography on silica gel (eluting with pentane with 0.25% v/v AcOEt and 1% v/v TEA) led to 223.3 mg (0.93 mmol, 93%) of **6h** as orange oil. **^{1}H NMR** (400 MHz, C_6D_6): δ = 7.80 – 7.73 (m, 2H), 7.62 – 7.55 (m, 1H), 7.20 – 7.04 (m, 4H), 6.95 – 6.86 (m, 1H), 6.56 – 6.49 (m, 1H), 5.08 (s, 2H), 4.74 (d, *J*=2.7 Hz, 1H), 4.34 (d, *J*=2.7 Hz, 1H), 3.27 (s, 3H); **^{13}C{^{1}H} NMR** (101 MHz, C_6D_6): δ = 160.4, 157.3, 137.3, 128.9, 128.6, 128.4, 126.0, 120.9, 110.4, 83.2, 65.3, 54.9; **IR** (film): $\tilde{\nu}$= 3057, 2935, 2838, 2324, 2076, 1898, 1686, 1640, 1602, 1494, 1461, 1385, 1285, 1244, 1176, 112, 1077, 1028, 953, 926, 804, 753, 696 cm^{-1}; **HRMS** (APCI): *m/z* calcd for $C_{16}H_{16}O_2+H^+$: 241.1229 [M+H]$^+$; found: 241.1233.

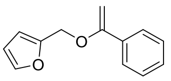

2-{[(1-Phenylethenyl)oxy]methyl}furan (6i): The product was synthesized following GP1 starting from 49.6 mg (0.4 mmol) of 2-[(vinyloxy)methyl]furan (**1i**) and 180 µL (1.1 mmol) of PhOTf (**2a**). Flash column chromatography on silica gel (eluting with pentane:DCM 1:1 with 1% v/v TEA) led to 63.0 mg (0.31 mmol, 79%) of **6i** as yellow oil.**^{1}H NMR** (600 MHz, C_6D_6): δ = 7.70 – 7.65 (m, 2H), 7.12 – 7.03 (m, 5H), 6.12 – 6.09 (m, 1H), 6.07 – 6.03 (m, 1H), 4.68 (d, *J*=3.0 Hz, 1H), 4.58 (s, 2H), 4.13 (d, *J*=3.0 Hz, 1H); **^{13}C{^{1}H} NMR** (151 MHz, C_6D_6): δ = 160.1, 151.0, 143.1, 136.8, 128.7, 128.4, 125.9, 110.7, 109.9, 83.2, 62.4; **IR** (film): $\tilde{\nu}$= 3123, 3059, 2926, 2865, 1641, 1608, 1572, 1495, 1448, 1282, 1220,

1148, 1119, 1078, 1020, 922, 885, 808, 768, 740, 695 cm^{-1}; **HRMS** (ESI): *m/z* calcd for $C_{13}H_{12}O_2$+Na^+: 223.0729 [M+Na]$^+$; found: 223.0724.

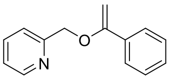

2-{[(1-Phenylethenyl)oxy]methyl}pyridine (6j): The product was synthesized following GP1 starting from 135.2 mg (1 mmol) of 2-[(vinyloxy)methyl]pyridine (**1j**) and 180 µL (1.1 mmol) of PhOTf (**2a**). Flash column chromatography on silica gel (eluting with pentane:DCE 1:1 with 1.5% v/v TEA) led to 80.2 mg (0.38 mmol, 38%) of **6j** as orange oil.**^{1}H NMR** (600 MHz, C_6D_6): δ = 8.46 (d, *J*=5.1 Hz, 1H), 7.75 – 7.69 (m, 2H), 7.35 – 7.30 (m, 1H), 7.20 – 7.05 (m, 4H), 6.62 (dd, *J*=7.0, 5.1 Hz, 1H), 5.07 (s, 2H), 4.67 (d, *J*=3.1 Hz, 1H), 4.22 (d, *J*=3.1 Hz, 1H); **^{13}C{^{1}H} NMR** (151 MHz, C_6D_6): δ = 159.7, 158.0, 149.4, 136.9, 136.2, 128.8, 128.5, 125.9, 122.3, 121.0, 84.1, 71.1; **IR** (film): $\tilde{\nu}$= 3058, 3020,2924, 2865, 2665, 2326, 2088, 1994, 1894, 1592, 1475, 1437, 1288, 1125, 1078, 1046, 994, 950, 901, 809, 763, 695 cm^{-1}; **HRMS** (ESI): *m/z* calcd for $C_{14}H_{13}NO$+H^+: 212.1070 [M+H]$^+$; found: 212.1065.

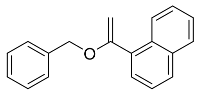

1-[1-(Benzyloxy)ethenyl]naphthalene (6k): The product was synthesized following GP1 starting from 134.2 mg (1 mmol) of benzyl vinyl ether (**1a**) and 215 µL (1.1 mmol) of 1-naphthyl trifluoromethanesulfonate (**2b**). Flash column chromatography on silica gel (eluting with pentane with 1% v/v TEA) led to 249.7 mg (0.96 mmol, 96%) of **6k** as colourless oil.**^{1}H NMR** (400 MHz, C_6D_6): δ = 8.47 – 8.38 (m, 1H), 7.66 – 7.55 (m, 3H), 7.35 – 7.28 (m, 1H), 7.28 – 7.23 (m, 3H), 7.23 – 7.17 (m, 1H), 7.14 – 7.04 (m, 3H), 4.76 (s, 2H), 4.50 (s, 2H); **^{13}C{^{1}H} NMR** (101 MHz, C_6D_6): δ = 161.6, 137.5, 136.4, 134.3, 132.1, 129.3, 128.6, 128.6, 127.9, 127.7, 127.2, 126.5, 126.4, 126.1, 125.4, 88.3, 70.1; **IR** (film): $\tilde{\nu}$= 3041, 2908, 2868, 1613, 1502, 1456, 1418, 1294, 1250, 1207, 1142, 1064, 1027, 949, 900, 802, 775, 740, 696 cm^{-1}; **HRMS** (ESI): *m/z* calcd for $C_{19}H_{16}O$+Na^+: 283.1093 [M+Na]$^+$; found: 283.1089.

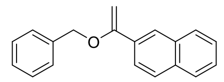

2-[1-(Benzyloxy)ethenyl]naphthalene (6l): The product was synthesized following GP1 starting from 134.2 mg (1 mmol) of benzyl vinyl ether (**1a**) and 215 µL (1.1 mmol) of 2-naphthyl trifluoromethanesulfonate (**2c**). Flash column chromatography on silica gel (eluting with pentane with 1% v/v TEA) led to 241.9 mg (0.93 mmol, 93%) of **6i** as slightly yellow solid. **m.p.** 57.7 °C; **^{1}H NMR** (400 MHz, C_6D_6): δ = 8.33 (s, 1H), 7.84 – 7.77 m, 1H), 7.65 – 7.53 (m, 3H), 7.33 – 7.27 (m, 2H), 7.23 – 7.17 (m, 4H), 7.14 – 7.08 (m, 1H), 4.88 (d, *J*=2.9 Hz, 1H), 4.75 (s, 2H), 4.30 (d, *J*=2.9 Hz, 1H); **^{13}C{^{1}H} NMR** (101 MHz, C_6D_6): δ = 160.1, 137.6, 134.2, 134.0, 133.8, 129.0, 128.8, 128.2, 127.9, 126.4, 126.4, 125.1, 123.9, 84.1, 70.2; **IR** (film): $\tilde{\nu}$= 3056, 2913, 2865, 1653, 1611, 1570, 1501, 1458, 1360, 1295, 1237, 1197, 1133, 1096, 1017, 953, 907, 867, 814, 749, 695 cm^{-1}; **HRMS** (ESI): *m/z* calcd for $C_{19}H_{16}O$+H^+: 261.1274 [M+H]$^+$; found:261.1272.

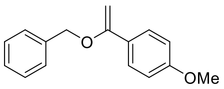

1-[1-(Benzyloxy)ethenyl]-4-methoxybenzene (6m): The product was synthesized following GP1 starting from 134.2 mg (1 mmol) of benzyl vinyl ether (**1a**) and 281.8 mg (1.1 mmol) of 4-methoxyphenyl trifluoromethanesulfonate (**2d**). Flash column chromatography on silica gel (eluting with pentane with 0.5% v/v AcOEt and 1.5% v/v TEA) led to 220.9 mg (0.92 mmol, 92%) of **6m** as white solid. **m.p.** 52.7 °C; **^{1}H NMR** (400 MHz, C_6D_6): δ = 7.75 – 7.65 (m, 2H), 7.32 – 7.24 (m, 2H), 7.20 – 7.14 (m, 2H), 7.14 – 7.07 (m, 1H), 6.79 – 6.71 (m, 2H), 4.71 (s, 2H), 4.70 (d, *J*=2.7 Hz, 1H), 4.17 (d, *J*=2.7 Hz, 1H), 3.27 (s, 3H); **^{13}C{^{1}H} NMR** (101 MHz, C_6D_6): δ = 160.6, 160.2, 137.8, 129.6, 128.7, 127.9, 127.2, 113.9, 81.7, 70.0, 54.8; **IR** (film): $\tilde{\nu}$=3066, 3034, 2963, 2909, 2862, 2837, 1642, 1602, 1505, 1458, 1409, 1287, 1244, 1177, 1116, 1027, 951, 835, 802,736, 698 cm^{-1}; **HRMS** (ESI): *m/z* calcd for $C_{16}H_{16}O_2$+Na^+: 263.1042 [M+Na]$^+$; found: 263.1039.

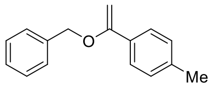

1-[1-(Benzyloxy)ethenyl]-4-methylbenzene (6n): The product was synthesized following GP1 starting from 670.9 mg (5 mmol) of benzyl vinyl ether (**1a**) and 985 µL (5.5 mmol) of *p*-tolyl trifluoromethanesulfonate (**2e**). Flash column chromatography on silica gel (eluting with pentane with 0.5% v/v Et_2O and 1% v/v TEA led to 1.08 g (4.8 mmol, 96%) of **6n** as white oil.**^{1}H NMR** (400 MHz, C_6D_6): δ = 7.75 – 7.67 (m, 2H), 7.31 – 7.23 (m, 2H), 7.20 – 7.13 (m, 2H), 7.13 – 7.07 (m, 1H), 7.02 – 6.95 (m, 2H), 4.76 (d, *J*=2.7 Hz, 1H), 4.70 (s, 2H), 4.19 (d, *J*=2.7 Hz, 1H), 2.08 (s, 3H); **^{13}C{^{1}H} NMR** (101 MHz, C_6D_6): δ = 160.4, 138.4, 137.7, 134.3, 129.2, 128.7, 127.9, 125.9, 82.6, 70.0, 21.1; **IR** (film): $\tilde{\nu}$= 3124,3031, 2918, 2866, 2318, 2086, 1904, 1805, 1637, 1604, 1568, 1509,1454, 1385, 1291, 1183, 1119, 1027, 950, 905, 798, 737, 696 cm^{-1}; **HRMS** (ESI): *m/z* calcd for $C_{16}H_{16}O$+H^+: 225.1274 [M+H]$^+$; found: 225.1271.

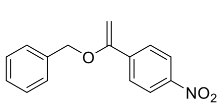

1-[1-(Benzyloxy)ethenyl]-4-nitrobenzene (6o): The product was synthesized following GP1 starting from 134.2 mg (1 mmol) of benzyl vinyl ether (**1a**) and 281.8 mg (1.1 mmol) of 4-nitrophenyl trifluoromethanesulfonate (**2f**). Flash column chromatography on silica gel (eluting with pentane:AcOEt 99:1 with 1% v/v TEA) led to 84.2 mg (0.33 mmol, 33%) of **6o** as purple solid. **m.p.** 66.2 °C; **^{1}H NMR** (400 MHz, C_6D_6): δ= 7.83 – 7.76 (m, 2H), 7.36 – 7.28 (m, 2H), 7.21 – 7.16 (m, 4H), 7.16 – 7.09 (m, 1H), 4.56 (d, *J*=3.3 Hz, 1H), 4.54 (s, 2H), 4.15 (d, *J*=3.3 Hz, 1H); **^{13}C{^{1}H} NMR** (101 MHz, C_6D_6): δ = 157.9, 148.1, 142.0, 136.9, 128.8, 128.3, 127.8, 126.1, 123.5, 86.4, 70.3; **IR** (film): $\tilde{\nu}$= 3112, 3083, 3030, 2925, 2874, 1594, 1512, 1428, 1339, 1287, 1216. 1180, 1136, 1104, 1021, 951, 885, 857, 824, 750, 698 cm^{-1}; **HRMS** (ESI): *m/z* calcd for $C_{15}H_{13}NO_3$+Na^+: 278.0785 [M+Na]$^+$; found: 278.0784.

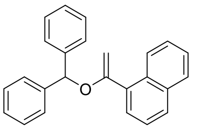

1-{[1-(Diphenylmethyl)oxy]ethenyl}naphthalene (6p): The product was synthesized following GP1 starting from 210.1 mg (1 mmol) of diphenylmethyl vinyl ether (**1b**) and 215 µL (1.1 mmol) 1-naphthyl trifluoromethanesulfonate (**2b**). Flash column chromatography on silica gel (eluting with pentane with 0.5% v/v AcOEt and 1% v/v TEA) led to 337.1 mg (0.96 mmol, 96%) of **6p** as creamy solid. **m.p.** 86.9 °C; **^{1}H NMR** (600 MHz, C_6D_6): δ = 8.47 – 8.43 (m, 1H), 7.65 – 7.58 (m, 2H), 7.58 – 7.53 (m, 1H), 7.38 – 7.31 (m, 4H), 7.30 – 7.17 (m, 3H), 7.13 – 7.07 (m, 4H), 7.05 – 6.99 (m, 2H), 6.11 (s, 1H), 4.66 (d, *J*=2.1 Hz, 1H), 4.50 (d, *J*=2.1 Hz, 1H); **^{13}C{^{1}H} NMR** (151 MHz, C_6D_6): δ = 159.7, 142.1, 136.3, 134.2, 132.1, 129.3, 128.8, 128.6, 127.8, 127.4, 127.2, 126.5,

126.5, 126.1, 125.4, 91.9, 81.8; **IR** (film): $\tilde{\nu}$= 3030, 1632, 1587, 493,1448, 1276, 1251, 1200, 1140, 1020, 956, 917, 831, 807, 779, 742, 697 cm^{-1}; **HRMS** (ESI): *m/z* calcd for $C_{25}H_{20}O+Na^+$: 359.1406 $[M+Na]^+$; found: 359.1402.

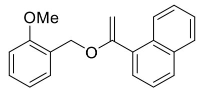

1-{[1-(2-Methoxybenzyl)oxy]ethenyl}naphthalene (6q): The product was synthesized following GP1 starting from 164.1 mg (1 mmol) of 1-[(ethenoxy)methyl]-2-methoxybenzene (**1h**) and 215 µL (1.1 mmol) 1-naphthyl trifluoromethanesulfonate (**2b**). Flash column chromatography on silica gel (eluting with pentane with 0.5% v/v AcOEt and 1% v/v TEA) led to 258.2 mg (0.89 mmol, 89%) of **6q** as creamy solid. **m.p.** 95.3 °C; **^{1}H NMR** (600 MHz, C_6D_6): δ = 8.55 – 8.49 (m, 1H), 7.65 – 7.57 (m, 3H), 7.51 – 7.47 (m, 1H), 7.33 – 7.28 (m, 1H), 7.27 – 7.23(m, 1H), 7.21 – 7.17 (m, 1H), 7.10 – 7.04 (m, 1H), 6.84 – 6.78 (m, 1H), 6.51 – 6.46 (m, 1H), 5.14 (s, 2H), 4.65 (d, *J*=2.0 Hz, 1H), 4.52 (d, *J*=2.0 Hz, 1H), 3.29 (s, 3H); **^{13}C{^{1}H} NMR** (151 MHz, C_6D_6): δ = 162.0, 157.3, 136.7, 134.3, 132.2, 129.2, 129.2, 129.0, 128.5, 127.2, 126.8, 126.3, 126.0, 125.4, 120.9, 110.3, 87.8, 65.6, 54.8; **IR** (film): $\tilde{\nu}$= 3042, 2928, 2879, 2836, 2079, 1652, 1606, 1496, 1459, 1400, 1298, 1249, 1199, 1144, 1119, 1062, 1011, 945, 805, 775, 751, 667 cm^{-1}; **HRMS** (ESI): *m/z* calcd for $C_{20}H_{18}O_2+Na^+$: 313.1199 $[M+Na]^+$; found: 313.1193.

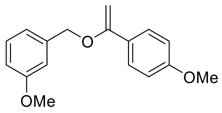

1-Methoxy-3-({[1-(4-methoxyphenyl)ethenyl]oxy}methyl)benzene (6r): The product was synthesized following GP1 starting from 164.1 mg (1 mmol) of 1-[(ethenoxy)methyl]-3-methoxybenzene (**1g**) and 281.8 mg (1.1 mmol) 4-methoxyphenyl trifluoromethane-sulfonate (**2d**). Flash column chromatography on silica gel (eluting with pentane with 0.5% v/v AcOEt and 1.5% v/v TEA) led to 251.2 mg (0.93 mmol, 93%) wof **6r** as white olid. **m.p.** 58.5 °C; **^{1}H NMR** (600 MHz, C_6D_6): δ = 7.74 – 7.68 (m, 2H), 7.15 – 7.11 (m, 1H), 7.01 (s, 1H), 6.97 – 6.93 (m, 1H), 6.78 – 6.71 (m, 3H), 4.73 (s, 2H), 4.71 (d, *J*=2.8 Hz, 1H), 4.19 (d, *J*=2.8 Hz, 1H), 3.32 (s, 3H), 3.26 (s, 3H); **^{13}C{^{1}H} NMR** (151 MHz, C_6D_6): δ = 160.5, 160.4, 160.1, 139.4, 129.8, 129.6, 127.2, 120.0, 113.9, 113.7, 113.4, 81.8, 69.9, 54.8; **IR** (film): $\tilde{\nu}$=2997, 2959, 2930, 2839, 2046, 1601, 1512, 1463, 1289, 1249, 1174, 1116, 1029, 956, 917, 836, 809, 782, 756, 689 cm^{-1}; **HRMS** (ESI): *m/z* calcd for for $C_{17}H_{18}O_3+Na^+$: 293.1148 $[M+Na]^+$; found: 293.1145.

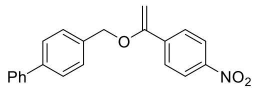

4-({[1-(4-Nitrophenyl)ethenyl]oxy}methyl)biphenyl (6s): The product was synthesized following GP1 starting from 210.3 mg (1 mmol) 1-(ethenoxymethyl)-4-phenylbenzene **1e** and 298.3 mg (1.1 mmol) 4-nitrophenyl trifluoromethanesulfonate (**2f**). Flash column chromatography on silica gel (eluting with pentane with 0.5% v/v AcOEt and 1.5% v/v TEA followed by pentane:AcOEt 9:1) led to 85.0 mg (0.24 mmol, 24%) of **6s** as purple solid. **m.p.** 127.5 °C; **^{1}H NMR** (600 MHz, C_6D_6): δ = 7.87 – 7.78 (m, 2H), 7.53 – 7.45 (m, 4H), 7.41 – 7.32 (m, 2H), 7.27 – 7.20 (m, 4H), 7.19 – 7.13 (m, 1H), 4.62 – 4.55 (m, 3H), 4.20 (d, *J*=3.4 Hz, 1H); **^{13}C{^{1}H} NMR** (151 MHz, C_6D_6): δ = 158.0, 148.1, 142.0, 141.7, 141.1, 135.8, 129.2, 128.4, 127.7, 127.5, 126.1, 123.6, 118.8, 86.4, 70.1; **IR** (film): $\tilde{\nu}$= 3036, 2932, 1698, 1591, 1512, 1488, 1335, 1283, 1249, 1106, 1015, 953, 854, 831, 807, 756, 726, 691 cm^{-1}; **HRMS** (ESI): *m/z* calcd for $C_{21}H_{17}NO_3+Na^+$: 354.1101 $[M+Na]^+$; found: 354.1091.

8. General procedure for the β-arylation of benzyl-type vinyl ethers using ArI (GP2)

In the flame-dried, argon-filled Schlenk flask, $PdCl_2$ (10.6 mg, 0,06 mmol, 6.0 mol %) and Bu_4NCl (555.8 mg, 2.0 mmol, 2.0 equiv.) were placed. The reagents were suspended in freshly degassed DMF (2.5 mL) and then, DIPEA (210 µL, 1.2 mmol), ArI (**4**, 1.1 mmol) and vinyl ether **1** (1.0 mmol) were added. Subsequently, the reaction mixture was degassed, positioned into the preheated oil bath (80 ˚C), and stirred (610 rpm) at this temperature for 2-24 h. After that time, the reaction mixture was cooled to room temperature and dilluted by Et_2O (5 mL) followed by addition of NaOH (10 mL of a 10% aq. solution). After the phase separation, the organic phase was washed with water (2 x 20 mL). Then, the collected water phases were one time extracted with Et_2O (10 mL). Finally, the combined organic phases were dried over Na_2SO_4, filtered and concentrated in vacuum. The product was isolated by flash column chromatography (20 mL silica in pentane with 1% v/v TEA) using pentane with 1% v/v TEA as eluent or pentane + 1% v/v TEA with AcOEt as additive for more polar derivatives (as marked below).

Note: The reaction flask must not be opened while the reaction is running. Even small amounts of air can affect the regioselectivity of the process.

9. Characterizing data for products 7

Except noted otherwise, products **7** were characterized as *E/Z* mixtures and in the presence of their regioisomeric counterparts **6**.

***alpha*-regioisomer (6a)**

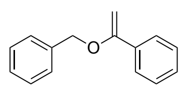

***beta*-regioisomer (7a)**

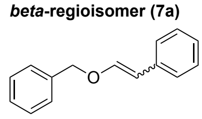

Mixture of α-(benzyloxy)styrene (6a) and β-(benzyloxy)styrene (7a): Obtained following GP2 starting from 134.2 mg (1 mmol) of benzyl vinyl ether (**1a**) and 125 µL (1.1 mmol) of iodobenzene (**4a**). Flash column chromatography on silica gel (eluting with pentane with 1% v/v AcOEt and 1% v/v TEA) led to 174.7 mg (0.83 mmol, 83%) of a yellow oil consisting of an 18:82 mixture of **6a** and **7a** (*E/Z* 46/54) as determined by NMR spectroscopy. **^{1}H NMR** (400 MHz, C_6D_6) α-regioisomer: δ = 7.81 – 7.75 (m, 2H), 4.78 (d, *J*=2.8 Hz, 1H), 4.71 (s, 3H), 4.23 (d, *J*=2.8 Hz, 1H); β-regioisomer, *E*-diastereomer: δ = 7.00 (d, *J*=12.9 Hz, 1H), 5.98 (d, *J*=12.9 Hz, 1H), , 4.53 (s, 3H); β-regioisomer, *Z*-diastereomer: δ = 5.98 (d, *J*=7.1 Hz, 1H), 5.28 (d, *J*=7.1 Hz, 1H), 4.50 (s, 3H); **^{13}C{^{1}H} NMR** (101 MHz, C_6D_6 α-regioisomer: δ = 160.2, 83.3, 70.0; β-regioisomer,

E-diastereomer: *δ* = 148.1, 107.3, 71.8; β-regioisomer, *Z*-diastereomer: *δ* = 146.5, 106.9, 74.8; **IR** (film): $\tilde{\nu}$ = 3029, 2927, 2868, 2334, 2084, 1882, 1813, 1646, 1600, 1493, 1449, 1369, 1280, 1210, 1144, 1082, 1026, 956, 919, 809, 775, 738, 694 cm^{-1}; **HRMS** (APCI): *m/z* calcd for $C_{15}H_{14}O+H^+$: 211.1123 [M+H]$^+$; found: 211.1128.

***alpha*-regioisomer (6b)**

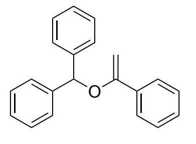

***beta*-regioisomer (7b)**

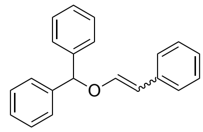

Mixture of diphenylmethyl 1-phenyl vinyl ether (6b) and diphenylmethyl 2-phenyl vinyl ether (7b): Obtained following GP2 starting from 210.1 mg (1 mmol) of diphenylmethyl vinyl ether (**1b**) and 125 µL (1.1 mmol) of iodobenzene (**4a**). Flash column chromatography on silica gel (eluting with pentane with 1% v/v AcOEt and 1% v/v TEA) led to 254.6 mg (0.89 mmol, 89%) of a yellow oil consisting of a 16:84 mixture of **6b** and **7b** (*E/Z* 36/64) as determined by NMR spectroscopy. **^{1}H NMR** (400 MHz, C_6D_6) α-regioisomer: *δ* = 7.79 – 7.75 (m, 2H), 6.08 (s, 1H), 4.74 (d, *J*=2.9 Hz, 1H), 4.26 (d, *J*=3.0 Hz, 1H); β-regioisomer, *E*-diastereomer: *δ* = 6.95 (d, *J*=12.8 Hz, 1H), 6.19 (d, *J*=12.6 Hz, 1H), 5.64 (s, 1H); β-regioisomer, *Z*-diastereomer: *δ* = 7.90 – 7.84 (m, 2H), 6.11 (d, *J*=7.1 Hz, 1H), 5.53 (s, 1H), 5.26 (d, *J*=7.1 Hz, 1H); **^{13}C{^{1}H} NMR** (151 MHz, C_6D_6) α-regioisomer: *δ* = 142.0, 86.4, 81.7; β-regioisomer, *E*-diastereomer: *δ* = 141.6, 147.0, 109.8, 84.7; β-regioisomer, *Z*-diastereomer: *δ* = 141.5, 145.5, 107.4, 86.7; **IR** (film): $\tilde{\nu}$ = 3059, 3029, 1649, 1492, 1449, 1304, 1261, 1188, 1137, 1076, 1008, 919, 746, 694 cm^{-1}; **HRMS** (APCI): *m/z* calcd for $C_{21}H_{18}O-H^+$: 285.1279 [M-H]$^+$; found: 285.1269.

***alpha*-regioisomer (6c)**

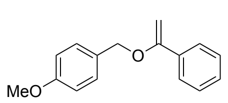

***beta*-regioisomer (7c)**

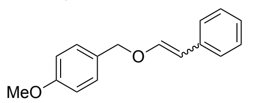

Mixture of *p*-methoxybenzyl 1-phenyl vinyl ether (6c) and *p*-methoxybenzyl 2-phenyl vinyl ether (7c): Obtained following GP2 starting from 164.1 mg (1 mmol) of 1-[(ethenoxy)methyl]-4-methoxybenzene (**1c**) and 125 µL (1.1 mmol) of iodobenzene (**4a**). Flash column chromatography on silica gel (eluting with pentane with 1% v/v AcOEt and 1% v/v TEA) led to 194.5 mg (0.81 mmol, 81%) of a yellow oil consisting of a 25:75 mixture of **6c** and **7c** (*E/Z* 42/58) as determined by NMR spectroscopy. **^{1}H NMR** (600 MHz, C_6D_6) α-regioisomer: *δ* = 4.77 (d, *J*=2.8 Hz, 1H), 4.66 (s, 2H), 4.24 (d, *J*=2.8 Hz, 1H), 3.30 (s, 3H); β-regioisomer, *E*-diastereomer: *δ* = 5.97 (d, *J*=13.0 Hz, 1H), 4.50 (s, 2H), 3.28 (s, 3H); β-regioisomer, *Z*-diastereomer: *δ* = 6.02 (d, *J*=7.1 Hz, 1H), 5.26 (d, *J*=7.1 Hz, 1H), 4.47 (s, 2H), 3.26 (s, 3H); **^{13}C{^{1}H} NMR** (151 MHz, C_6D_6) α-regioisomer: *δ* = 160.3, 160.0, 137.1, 114.2, 83.1, 69.9, 54.8; β-regioisomer, *E*-diastereomer: *δ* = 160.1, 148.2, 137.0, 114.2, 107.2, 71.7, 54.8; β-regioisomer, *Z*-diastereomer: *δ* = 160.1, 146.5, 136.8, 114.3, 106.7, 74.7, 54.8; **IR** (film): $\tilde{\nu}$=3060, 3029, 3002, 2921, 2870, 2837, 1639, 1610, 1511, 1448, 1372, 1303, 1245, 1176, 1145, 1076, 1029, 938, 816, 774, 747, 693 cm^{-1}; **HRMS** (APCI): *m/z* calcd for $C_{16}H_{16}O_2-H^+$: 239.1072 [M-H]$^+$; found: 239.1071.

***alpha*-regioisomer (6e)**

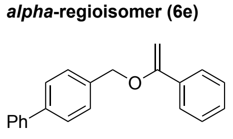

***beta*-regioisomer (7e)**

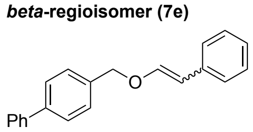

Mixture of 4-{[(1-phenylethenyl)oxy]methyl}biphenyl (6e) and 4-{[(2-phenylethenyl)oxy]methyl} biphenyl (7e): Obtained following GP2 starting from 210.3 mg (1 mmol) of 1-[(ethenoxy)methyl]-4-phenylbenzene (**1e**) and 125 µL (1.1 mmol) of iodobenzene (**4a**). Flash column chromatography on silica gel (eluting with pentane with 1% v/v AcOEt and 1% v/v TEA) led to 237.5 mg (0.83 mmol, 83%) of a brown waxy solid consisting of a 29:71 mixture of **6e** and **7e** (*E/Z* 10/90) as determined by NMR spectroscopy. **^{1}H NMR** (400 MHz, DMSO-d_6) α-regioisomer: *δ* = 4.99 (s, 2H), 4.84 (d, *J*=2.9 Hz, 1H), 4.44 (d, *J*=2.9 Hz, 1H); β-regioisomer, *E*-diastereomer: *δ* = 5.95 (d, *J*=12.9 Hz, 1H), 4.95 (s, 2H); β-regioisomer, *Z*-diastereomer: *δ* = 6.48 (d, *J*=7.0 Hz, 1H), 5.26 (d, *J*=7.1 Hz, 1H), 5.05 (s, 2H); **^{13}C{^{1}H} NMR** (101 MHz, DMSO-d_6) α-regioisomer: *δ* = 158.4, 139.8, 139.7, 136.2, 135.8, 84.0, 68.9; β-regioisomer, *E*-diastereomer: *δ* = 148.3, 139.9, 139.7, 136.6, 135.9, 106.4, 71.1; β-regioisomer, *Z*-diastereomer: *δ* = 147.3, 139.9, 139.7, 136.6, 135.9, 105.2, 74.1; **IR** (film): $\tilde{\nu}$= 3031, 2920, 2868, 2778, 1629, 1485, 1449, 1406, 1376, 1314, 1222, 1077, 1028, 1003, 934, 852, 825, 757, 688 cm^{-1}; **HRMS** (APCI): *m/z* calcd for $C_{21}H_{18}O+H^+$: 287.1436 [M+H]$^+$; found: 287.1426.

***alpha*-regioisomer (6g)**

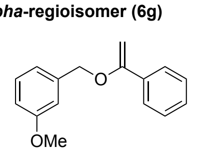

***beta*-regioisomer (7g)**

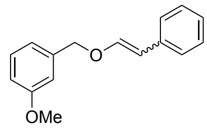

Mixture of *m*-methoxybenzyl 1-phenyl vinyl ether (6g) and *m*-methoxybenzyl 2-phenyl vinyl ether (7g): Obtained following GP2 starting from 164.1 mg (1 mmol) of 1-[(ethenoxy)methyl]-3-methoxybenzene (**1g**) and 125 µL (1.1 mmol) of iodobenzene (**4a**). Flash column chromatography on silica gel (eluting with pentane with 1% v/v AcOEt and 1% v/v TEA) led to 199.3 mg (0.83 mmol, 83%) of a yellow oil consisting of a 23:77 mixture of **6g** and **7g** (*E/Z* 43/57) as determined by NMR spectroscopy. **^{1}H NMR** (400 MHz, C_6D_6) α-regioisomer: *δ* = 4.73 (d, *J*=2.8 Hz, 1H), 4.68 (s, 2H), 4.19 (d, *J*=2.8 Hz, 1H); β-regioisomer, *E*-diastereomer: *δ* = 5.94 (d, *J*=12.9 Hz, 1H), 4.50 (s, 2H); β-regioisomer, *Z*-diastereomer: *δ* = 5.95 (d, *J*=7.1 Hz, 1H), 5.23 (d, *J*=7.1 Hz, 1H), 4.47 (s, 2H); **^{13}C{^{1}H} NMR** (101 MHz, C_6D_6) α-regioisomer: *δ* = 160.5, 160.2, 139.2, 137.0, 120.0, 113.7, 113.4, 83.4, 70.0, 54.8; β-regioisomer, *E*-diastereomer: *δ* = 160.5, 148.1, 139.0, 136.9, 119.9, 113.8, 113.3, 107.4, 71.7, 54.8; β-regioisomer, *Z*-diastereomer: *δ* = 160.5, 146.5, 139.3, 136.6, 119.5, 114.0, 112.9, 107.0, 74.7, 54.7; **IR** (film): $\tilde{\nu}$=3027, 2938, 2837, 1645, 1598, 1492, 1457, 1368, 1246, 1148, 1081, 1031, 924, 752, 693 cm^{-1}; **HRMS** (APCI): *m/z* calcd for $C_{16}H_{16}O_2+H^+$: 241.1229 [M+H]$^+$; found: 241.1224.

***alpha*-regioisomer (6h)**

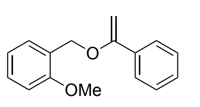

***beta*-regioisomer (7h)**

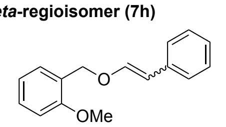

Mixture of *o*-methoxybenzyl 1-phenyl vinyl ether (6h) and *o*-methoxybenzyl 2-phenyl vinyl ether (7h): Obtained following GP2 starting from 164.1 mg (1 mmol) of 1-[(ethenoxy)methyl]-2-methoxybenzene (**1h**) and 125 µL (1.1 mmol) of iodobenzene (**4a**). Flash column chromatography on silica gel (eluting with pentane with 1% v/v AcOEt and 1% v/v TEA) led to 199.3 mg (0.83 mmol, 83%) of a yellow oil consisting of a 24:76 mixture of **6h** and **7h** (*E/Z* 48/52) as determined by NMR spectroscopy. **^{1}H NMR** (600 MHz, C_6D_6) α-regioisomer: *δ* = 5.08 (s, 2), 4.74 (d, *J*=2.8 Hz, 1H), 4.34 (d, *J*=2.8 Hz, 1H); β-regioisomer, *E*-diastereomer: *δ* = 6.10 (d, *J*=7.0 Hz, 1H), 5.23 (d, *J*=7.0 Hz, 1H), 4.91 (s, 2H); β-regioisomer, *Z*-diastereomer: *δ* = 6.03 (d, *J*=12.9 Hz, 1H), 4.88 (s, 2H); **^{13}C{^{1}H} NMR** (151 MHz, C_6D_6) α-regioisomer: *δ* = 160.4, 157.3, 137.3, 120.9, 107.0, 83.3, 65.3, 54.9; β-regioisomer, *E*-diastereomer: *δ* = 157.2, 148.6, 137.1, 120.9, 106.6, 67.4, 54.8; β-

regioisomer, *Z*-diastereomer: *δ* = 15157.2, 147.2, 136.8, 120.9, 110.4, 70.7, 54.8; **IR** (film): $\tilde{\nu}$=3027, 2938, 2838, 1644, 1599, 1493, 1459, 1370, 1285, 1244, 1144, 1121, 1082, 1028, 925, 751, 690 cm^{-1}; **HRMS** (APCI): *m/z* calcd for $C_{16}H_{16}O_2$+H$^+$: 241.1229 [M+H]$^+$; found: 241.1226.

***beta*-regioisomer (7i)**

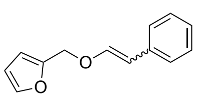

2-{[(2-Phenylethenyl)oxy]methyl}furan (7i): Obtained (as single regioisomer) following GP2 starting from starting from 62.1 mg (0.5 mmol) 2-[(vinyloxy)methyl]furan (**1g**) and 65 µL (0.55 mmol) of iodobenzene (**4a**). Flash column chromatography on silica gel (eluting with pentane with 1% v/v AcOEt and 1% v/v TEA) led to 70.6 mg (0.35 mmol, 70%) as a yellow oil of **7i** (*E/Z* 46/54 as determined by NMR spectroscopy). **^{1}H NMR** (400 MHz, C_6D_6) β-regioisomer, *E*-diastereomer: *δ* = 6.90 (d, *J*=12.9 Hz, 1H), 5.92 (d, *J*=12.9 Hz, 1H), 4.40 (s, 1H); β-regioisomer, *Z*-diastereomer: *δ* = 5.22 (d, *J*=7.1 Hz, 1H), 4.35 (s, 1H); **^{13}C{^{1}H} NMR** (101 MHz, C_6D_6) β-regioisomer, *E*-diastereomer: *δ* = 147.6, 143.2, 110.7, 110.0, 107.7, 64.0; β-regioisomer, *Z*-diastereomer: *δ* = 145.8, 143.4, 110.6, 110.0, 107.2, 66.5; **IR** (film): $\tilde{\nu}$= 3028, 2925, 2868, 1684, 1647, 1599, 1496, 1448, 1360, 1288, 1257, 1226, 1149, 1072, 1007, 918, 814, 743, 694 cm^{-1}; **HRMS** (APCI): *m/z* calcd for $C_{13}H_{11}O_2$+H$^+$: 201.0916 [M+H]$^+$; found: 201.0922.

***alpha*-regioisomer (6m)**

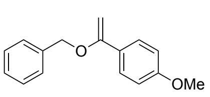

***beta*-regioisomer (7m)**

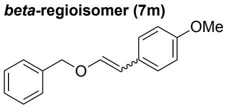

Mixture of 1-[1-(benzyloxy)ethenyl]-4-methoxybenzene (6m) and 1-[2-(benzyloxy)ethenyl]-4-methoxybenzene (7m): Obtained following GP2 starting from 134.2 mg (1 mmol) benzyl vinyl ether (**1a**) and 203.6 mg (1.1 mmol) of 1-iodo-4-methoxybenzene (**4b**). Flash column chromatography on silica gel (eluting with pentane with 1% v/v AcOEt and 1% v/v TEA) led to 175.6 mg (0.73 mmol, 73%) of a yellow waxy solid consisting of a 47:53 mixture of **6m** and **7m** (*E/Z* 38/62) as determined by NMR spectroscopy. **^{1}H NMR** (600 MHz, C_6D_6) α-regioisomer: *δ* = 4.71 (m, 3H), 4.17 (d, *J*=2.8 Hz, 1H), 3.26 (s, 5H); β-regioisomer, *E*-diastereomer: *δ* = 6.91 (d, *J*=12.9 Hz, 1H), 5.98 (d, *J*=12.9 Hz, 1H), 4.53 (s, 2H), 3.33 (s, 3H); β-regioisomer, *Z*-diastereomer: *δ* = 5.93 (d, *J*=7.1 Hz, 1H), 5.26 (d, *J*=7.0 Hz, 1H), 4.49 (s, 2H), 3.30 (s, 3H); **^{13}C{^{1}H} NMR** (151 MHz, C_6D_6) α-regioisomer: *δ* = 160.5, 160.1, 137.8, 113.9, 81.7, 70.0, 54.8; β-regioisomer, *E*-diastereomer: *δ* = 158.4,144.9, 137.9, 114.1, 106.6, 74.7, 54.7; β-regioisomer, *Z*-diastereomer: *δ* = 158.6, 146.8, 137.7, 114.5, 107.0, 71.8, 54.8; **IR** (film): $\tilde{\nu}$= 3032, 2933, 2836, 2063, 1644, 1606, 1508, 1456, 1369, 1287, 1249, 1176, 1147, 1088, 1030, 834, 803, 737, 696 cm^{-1}; **HRMS** (APCI): *m/z* calcd for $C_{16}H_{16}O_2$+H$^+$: 241.1229 [M+H]$^+$; found: 241.1218.

***alpha*-regioisomer (6n)**

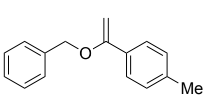

***beta*-regioisomer (7n)**

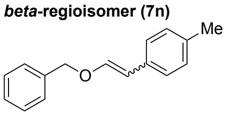

Mixture of 1-[1-(benzyloxy)ethenyl]-4-methylbenzene (6n) and 1-[2-(benzyloxy)ethenyl]-4-methylbenzene (7m): Obtained following GP2 starting from 268.4 mg (2 mmol) benzyl vinyl ether and 479.7 mg (2.2 mmol) of 1-iodo-4-methylbenzene (**4c**). Flash column chromatography on silica gel (eluting with pentane with 1% v/v AcOEt and 1% v/v TEA) led to 367.6 mg (1.64 mmol, 73%) of a yellow waxy solid consisting of a 26:74 mixture of **6n** and **7n** (*E/Z* 46/54) as determined by NMR spectroscopy. **^{1}H NMR** (600 MHz, C_6D_6) α-regioisomer: *δ* = 4.76 (d, *J*=2.7 Hz, 1H), 4.70 (s, 2H), 4.19 (d, *J*=2.8 Hz, 1H), 2.08 (s, 3H); β-regioisomer, *E*-diastereomer: *δ* = 5.98 (d, *J*=12.9 Hz, 1H), 4.51 (s, 2H), 2.14 (s, 3H); β-regioisomer, *Z*-diastereomer: *δ* = 5.94 (d, *J*=7.0 Hz, 1H), 5.27 (d, *J*=7.0 Hz, 1H), 4.48 (s, 2H), 2.12 (s, 3H); **^{13}C{^{1}H} NMR** (151 MHz, C_6D_6) α-regioisomer: *δ* = 160.3, 138.4, 137.7, 134.3, 82.6, 70.3, 21.2; β-regioisomer, *E*-diastereomer: *δ* = 147.5, 137.6, 135.5, 134.0, 107.3, 71.8, 21.1; β-regioisomer, *Z*-diastereomer: *δ* = 145.9, 137.8, 135.3, 133.9, 107.0, 74.7, 21.2; The spectroscopic data are in agreement with the previously reported ones.[S11,S12]

***beta*-regioisomer (7o)**

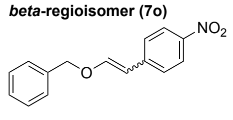

1-[2-(Benzyloxy)ethenyl]-4-nitrobenzene (7o): Obtained (as single regioisomer) following GP2 starting from starting from 134.2 mg (1 mmol) benzyl vinyl ether (**1a**) and 273.9 mg (1.1 mmol) of 1-nitro-4-iodobenzene (**4d**). Flash column chromatography on silica gel (eluting with pentane with 1% v/v Et_2O and 1% v/v TEA) led to 159.7 mg (0.63 mmol, 63%) of a yellow waxy solid of **7o** (*E/Z* 53/37 as determined by NMR spectroscopy). **^{1}H NMR** (600 MHz, C_6D_6) β-regioisomer, *E*-diastereomer: *δ* = 6.78 (d, *J*=12.9 Hz, 1H), 5.61 (d, *J*=12.9 Hz, 1H), 4.45 (s, 2H); β-regioisomer, *Z*-diastereomer: *δ* = 5.91 (d, *J*=7.0 Hz, 1H), 4.95 (d, *J*=7.0 Hz, 1H), 4.39 (s, 2H); **^{13}C{^{1}H} NMR** (151 MHz, C_6D_6) β-regioisomer, *E*-diastereomer: *δ* = 149.8, 145.7, 142.6, 136.7, 104.8, 75.5; β-regioisomer, *Z*-diastereomer: *δ* = 151.3, 145.9, 143.4, 136.8, 105.7, 72.4; **IR** (film): $\tilde{\nu}$= 3067, 3036, 2922, 2871, 1630, 1587, 1499, 1456, 1335, 1237, 1156, 1105, 1076, 1011, 941, 854, 735, 696 cm^{-1}; **HRMS** (APCI): *m/z* calcd for $C_{15}H_{13}NO_3$+H$^+$: 256.0974 [M+H]$^+$; found: 256.0966.

***beta*-regioisomer (7t)**

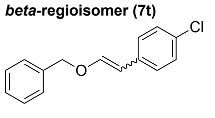

1-[2-(Benzyloxy)ethenyl]-4-chlorobenzene (7t): Obtained (as single regioisomer) following GP2 starting from starting from 268.4 mg (2 mmol) benzyl vinyl ether (**1a**) and 524.6 mg (2.2 mmol) of 1-chloro-4-iodobenzene (**4e**). Flash column chromatography on silica gel (eluting with pentane with 1% v/v AcOEt and 1% v/v TEA) led to 262.9 mg (1.08 mmol, 54%) of a yellow waxy solid of **7t** (*E/Z* 42/58 as determined by NMR spectroscopy). **^{1}H NMR** (600 MHz, C_6D_6) β-regioisomer, *E*-diastereomer: *δ* = 6.85 (d, *J*=12.9 Hz, 1H), 5.80 (d, *J*=12.9 Hz, 1H), 4.54 (s, 2H); β-regioisomer, *Z*-diastereomer: *δ* = 5.95 (d, *J*=7.0 Hz, 1H), 5.12 (d, *J*=7.0 Hz, 1H), 4.48 (s, 2H); **^{13}C{^{1}H} NMR** (151 MHz, C_6D_6) β-regioisomer, *E*-diastereomer: *δ* = 148.5, 137.2, 135.3, 133.7, 106.1, 71.9; β-regioisomer, *Z*-diastereomer: *δ* = 146.9, 137.4, 135.9, 135.0, 105.6, 74.9; **IR** (film): $\tilde{\nu}$=3033, 2929, 2870, 1646, 1489, 1455, 1368, 1291, 1262, 1234, 1149, 1088, 1011, 919, 833, 732, 697 cm^{-1}; **HRMS** (APCI): *m/z* calcd for $C_{15}H_{13}ClO$+H$^+$: 245.0728 [M+H]$^+$; found: 245.0735.

***alpha*-regioisomer (6u)**

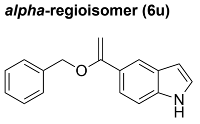

***beta*-regioisomer (7u)**

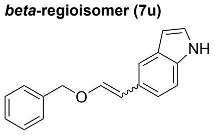

Mixture of 5-[1-(benzyloxy)ethenyl]-1H-indole (6u) and 5-[2-(benzyloxy)ethenyl]-1H-indole (7u): Obtained following GP2 starting from 26.8 mg (0.2 mmol) benzyl vinyl ether (**1a**) and 54.3 mg of (0.22 mmol) 5-iodoindole (**4f**). Flash column chromatography on silica gel (eluting with pentane with 1% v/v AcOEt and 1% v/v TEA) led to 22.5 mg (0.09 mmol, 45%) of a yellow oil consisting of a 1:1 mixture of **6u** and **7u** (*E/Z* 47/53) as determined by NMR spectroscopy. **^{1}H NMR** (600 MHz, C_6D_6) α-regioisomer: *δ* = 4.89 (d, *J*=2.7 Hz, 1H), 4.80 (s, 2H), 4.27 (d, *J*=2.7 Hz, 1H); β-regioisomer, *E*-diastereomer: *δ* = 6.25 (d, *J*=12.9 Hz, 1H), 4.58 (s, 2H); β-regioisomer, *Z*-diastereomer: *δ* = 6.01 (d, *J*=7.0 Hz, 1H), 5.51 (d, *J*=7.0 Hz, 1H), 4.55 (s, 2H); **^{13}C{^{1}H} NMR** (151 MHz, C_6D_6)

α-regioisomer: δ = 161.7, 81.8, 70.0; β-regioisomer, *E*-diastereomer: δ = 146.4, 108.8, 71.8; β-regioisomer, *Z*-diastereomer: δ = 144.4, 108.3, 74.6; **IR** (film): $\tilde{\nu}$= 33414, 3031, 2925, 2868, 2321, 2019, 1879, 1644, 1455, 1414, 1320, 1250, 1131, 1094, 1023, 884, 802, 727, 699 cm^{-1}; **HRMS** (ESI): *m/z* calcd for $C_{17}H_{15}NO+H^+$: 250.1226 $[M+H]^+$; found: 250.1228.

10. General procedure for the selective hydrolysis of 6 in mixtures of 6 and 7 (GP3)

The mixture of regioisomers **6** and **7** (1 mmol) obtained using procedure GP2 was dissolved in dry THF (6 mL) and HCOOH (2 mL) was added dropwise. After stirring the reaction mixture (510 rpm) at room temperature for 20 h, Et_2O (12 mL) was added, and the mixture was neutralized (pH = 7) by slow addition of NaOH (10% aq. solution). After phase separation, the organic phase was washed with NaOH (10% aq., 2 x 10mL). Finally, the organic phase was dried over Na_2SO_4, filtered and concentrated in vacuum. The product was isolated by flash column chromatography using silica gel and pentane with 1% v/v TEA as an eluent or pentane + 1% v/v TEA with AcOEt as additive for more polar derivatives (as marked below).

11. Characterizing data of products 7 (obtained by GP3 as single regioisomers)

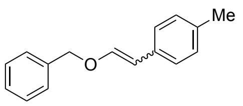

β-(Benzyloxy)styrene (7a): Obtained following GP3 starting from 210.1 mg (1 mmol) of a mixture of α and β (*E/Z* 43/57) regioisomers in a ratio of 32/68. Flash column chromatography on silica gel (eluting with pentane with 1% v/v 1% v/v TEA) led to 143.0 mg (0.68 mmol, 68%) of a yellow oil of **7a** as single regioisomer (*E/Z* 44/56) as determined by NMR spectrososcopy. **^{1}H NMR** (600 MHz, C_6D_6) *E*-diastereomer: δ = 7.00 (d, *J*=12.9 Hz, 1H), 4.53 (s, 2H); *Z*-diastereomer: δ = 5.28 (d, *J*=7.0 Hz, 1H), 4.50 (s, 2H); **^{13}C{^{1}H} NMR** (151 MHz, C_6D_6) *E*-diastereomer: δ = 148.1, 107.3, 71.8; *Z*-diastereomer: δ = 146.5, 106.9, 74.8; **IR** (film): $\tilde{\nu}$= 3029, 2925, 2866, 2326, 2088, 1928, 1646, 1599, 1493, 1449, 1369, 1311, 1267, 1210, 1145, 1082, 1025, 920, 777, 736, 693 cm^{-1}; **MS** (EI): *m/z* (%): 211 (18) $[M+H]^+$, 210 (100) $[M]^+$, 181 (18), 167(8); 91 (30) $[C_7H_7]^+$. The spectroscopic data in agreement with the previously reported in the literature.[S13–S15]

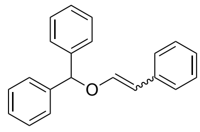

Diphenylmethyl 2-phenyl vinyl ether (7b): Obtained following GP3 starting from 285.1 mg (1 mmol) of a mixture of α and β (*E/Z* 36/64) regioisomers in a ratio of 19/81. Flash column chromatography on silica gel (eluting with pentane with 1% v/v AcOEt and 1% v/v TEA) led to 231.9 mg (0.81 mmol, 81%) of **7b** as single regioisomer (*E/Z* 35/65) as determined by NMR spectrososcopy. **^{1}H NMR** (600 MHz, C_6D_6) *E*-diastereomer: δ = 6.95 (d, *J*=12.7 Hz, 1H), 6.19 (d, *J*=12.7 Hz, 1H), 5.65 (s, 1H); *Z*-diastereomer: δ = 6.11 (d, *J*=7.1, 1H), 5.53 (s, 1H), 5.26 (d, *J*=7.1, 1H); **^{13}C{^{1}H} NMR** (151 MHz, C_6D_6) *E*-diastereomer: δ = 147.0, 141.6, 136.6, 109.8, 84.7; *Z*-diastereomer: δ =145.5, 141.5, 136.6, 109.8, 86.7; **IR** (film): $\tilde{\nu}$= 3030, 1649, 1492, 1448, 1260, 1137, 1076, 1006, 919, 776, 747, 694 cm^{-1}; **HRMS** (APCI): *m/z* calcd for $C_{21}H_{18}O-H^+$: 285.1279 $[M-H]^+$; found: 285.1267.

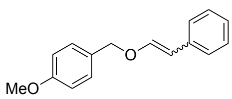

***p*-Methoxybenzyl 2-phenyl vinyl ether (7c):** Obtained following GP3 starting from 240.3 mg (1 mmol) of a mixture of α and β (*E/Z* 41/59) regioisomers in a ratio of 29/71. Flash column chromatography on silica gel (eluting with pentane with 1% v/v AcOEt and 1% v/v TEA) led to 168.2 mg (0.70 mmol, 70%) of a white waxy solid of **7c** as single regioisomer (*E/Z* 46/54) as determined by NMR spectrososcopy. **^{1}H NMR** (400 MHz, C_6D_6) *E*-diastereomer: δ = 5.97 (d, *J*=12.9 Hz, 1H), 4.51 (s, 2H), 3.30 (s, 3H); *Z*-diastereomer: δ = 6.03 (d, *J*=7.1 Hz, 1H), 5.26 (d, *J*=7.1 Hz, 1H), 4.48 (s, 2H), 3.27 (s, 3H); **^{13}C{^{1}H} NMR** (101 MHz, C_6D_6) *E*-diastereomer: δ = 160.1, 148.2, 137.0, 114.2, 107.2, 71.7, 54.8; *Z*-diastereomer: δ =160.1, 146.5, 136.8, 114.2, 106.7, 74.7, 54.8; **IR** (film): $\tilde{\nu}$= 3057, 3029, 2989, 2960, 2931, 2835,1632, 1611, 1511, 1448, 1334, 1308, 1247, 1148, 1110, 1077, 1032, 937, 818, 745, 692 cm^{-1}; HRMS (EI) *m/z* calcd for $C_{16}H_{16}O_2+H^+$: 241.1223 $[M+H]^+$; found: 241.1225.

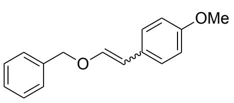

1-[2-(Benzyloxy)ethenyl]-4-methoxybenzene (7m): Obtained following GP3 starting from 144.2 mg (0.6 mmol) of a mixture of α and β (*E/Z* 38/62) regioisomers in a ratio of 58/42. Flash column chromatography on silica gel (eluting with pentane with 1% v/v AcOEt and 1% v/v TEA) led to 61.1mg (0.25 mmol, 42%) of a yellow waxy solid of **7m** as single regioisomer (*E/Z* 38/62) as determined by NMR spectrososcopy. **^{1}H NMR** (400 MHz, C_6D_6) β-regioisomer, *E*-diastereomer: δ = 6.91 (d, *J*=12.9 Hz, 1H), 5.97 (d, *J*=12.9 Hz, 1H), 4.53 (s, 2H), 3.33 (s, 3H); β-regioisomer, *Z*-diastereomer: δ = 5.93 (d, *J*=7.0 Hz, 1H), 5.26 (d, *J*=7.0 Hz, 1H), 4.49 (s, 2H), 3.30 (s, 3H); **^{13}C{^{1}H} NMR** (101 MHz, C_6D_6) *E*-diastereomer: δ = 158.6, 146.8, 137.6, 114.1, 107.0, 71.7, 54.8 ; *Z*-diastereomer: δ = 158.4, 144.9, 137.9, 114.5, 106.6, 74.7, 54.7; **IR** (film): $\tilde{\nu}$= 3035, 2997, 2916, 2868, 2840, 1636, 1607, 1573, 1508, 1458, 1375, 1322, 1288, 1246, 1178, 1150, 1089, 1031, 933, 838, 747, 696 cm^{-1}; **MS** (EI): *m/z* (%): 240 (100) $[M]^+$, 211 (57), 149 (41), 121(27); 91 (12) $[C_7H_7]^+$; HRMS (EI) *m/z* calcd for $C_{16}H_{16}O_2+H^+$: 241.1223 $[M+H]^+$; found: 241.1225.

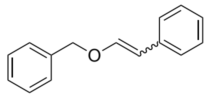

1-[2-(benzyloxy)ethenyl]-4-methylbenzene (7n): Obtained following GP3 starting from 224.1 mg (1 mmol) of a mixture of α and β (*E/Z* 46/54) regioisomers in a ratio of 22/78. Flash column chromatography on silica gel (eluting with pentane with 1% v/ AcOEt and 1% v/v TEA) led to 164.4 mg (0.73 mmol, 73%) of a yellow waxy solid of **7n** as single regioisomer (*E/Z* 46/54) as determined by NMR spectrososcopy. **^{1}H NMR** (600 MHz, C_6D_6) *E*-diastereomer: δ = 5.97 (d, *J*=12.9 Hz, 1H), 4.51 (s, 2H), 2.14 (s, 3H); *Z*-diastereomer: δ = 5.94 (d, *J*=7.0 Hz, 1H), 5.27 (d, *J*=7.0 Hz, 1H), 4.48 (s, 2H), 2.12 (s, 3H); **^{13}C{^{1}H} NMR** (151 MHz, C_6D_6) *E*-diastereomer: δ = 147.5, 137.6, 135.4, 135.3, 133.9, 107.3, 71.8, 21.1; *Z*-diastereomer: δ = 145.9, 137.8, 135.5, 134.0, 106.9, 74.7, 21.2; **IR** (film): $\tilde{\nu}$=3020, 2915, 2863, 1635, 1510, 1454, 1375, 1323, 1223, 1147, 1103, 939, 824, 743, 696 cm^{-1}; **HRMS** (ESI): *m/z* calcd for $C_{16}H_{16}O+H^+$: 225.1274 $[M+H]^+$; found: 225.1276.

12. Characterizing data of products 9 and 11

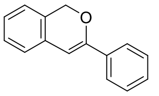

3-Phenyl-1*H*-isochromene (9): Obtained following GP2 starting from 93.3mg (0.35 mmol) of 1-[(ethenoxy)methyl]-2-iodobenzene (**8**) and 45 µL (0.39 mmol) of iodobenzene (**4a**). Flash column chromatography on silica gel (eluting with pentane with 1% v/v TEA) led to 32.1 mg (0.15 mmol, 52%) of **9** as slightly yellow oil.**^{1}H NMR** (400 MHz, $CDCl_3$): δ = 8.03 – 7.96 (m, 2H), 7.39 – 7.30 (m, 2H), 7.23 – 7.18 (m, 1H), 7.13 – 7.07 (m, 1H), 7.03 – 6.92 (m, 3H), 6.72 – 6.65 (m, 1H), 6.01 (s, 1H), 4.86 (s, 2H); **^{13}C{^{1}H} NMR** (101 MHz, $CDCl_3$): δ = 156.6, 139.6, 137.1, 135.3, 128.7, 128.6, 128.5, 125.8, 121.3, 120.2, 97.1, 74.8; **IR** (film): $\tilde{v}$= 3026, 2926, 2870, 1650, 1596, 1490, 1449, 1373, 1102, 1073, 103, 814, 756, 964 cm^{-1}; **HRMS** (ESI): *m/z* calcd for $C_{15}H_{12}O+H^+$: 209.0957 [M+H]$^+$; found: 209.0961.

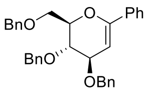

(2*R*,3*S*,4*R*)-3,4-Bis(benzyloxy)-2-[(benzyloxy)methyl]-6-phenyl-3,4-dihydro-2*H*-pyran (11): Obtained following GP2 starting from 208.3 mg (0.5 mmol) 3,4,6-tri-*O*-benzyl-D-glucal **10** and 65 µL of (0.55 mmol) of iodobenzene (**4a**). Flash column chromatography on silica gel (eluting with pentane with 1% v/v TEA) led to 157.1 mg (0.3 mmol, 73%) of **11** as slightly yellow oil.**^{1}H NMR** (600 MHz, $CDCl_3$): δ = 7.51 – 7.45 (m, 2H), 7.27 – 7.23 (m, 2H), 7.22 – 7.18 (m, 4H), 7.18 – 6.99 (m, 12H), 5.32 (d, *J*=3.2, 1H), 4.89 (d, *J*=11.6, 1H), 4.69 (d, *J*=3.5, 1H), 4.58 (d, *J*=11.6, 1H), 4.45 (s, 2H), 4.33 (d, *J*=12.2, 1H), 4.29 (dd, *J*=6.2, 1.3, 1H), 4.24 (d, *J*=12.2, 1H), 4.18 (ddd, *J*=6.2, 5.2, 4.0 Hz, 1H), 3.60 (dd, *J*=10.4, 5.2, 1H), 3.47 (dd, *J*=10.4, 4.0, 1H); **^{13}C{^{1}H} NMR** (151 MHz, $CDCl_3$): δ = 153.7, 141.9, 139.4, 139.2, 137.5, 128.7, 128.6, 128.5, 128.5, 128.4, 128.3, 128.0, 127.8, 127.7, 127.6, 99.5, 73.9, 73.7, 73.4, 73.3, 72.2, 69.8, 69.2; **IR** (film): $\tilde{v}$= 3061, 3030, 2864, 1666, 1494, 1452, 1360, 1206, 1178, 1093, 1024, 874, 735, 697 cm^{-1}; **HRMS** (ESI): *m/z* calcd for $C_{33}H_{32}O_4+Na^+$: 515.2193 [M+H]$^+$; found 515.218.

13. Characterizing data of compounds 13 – 15

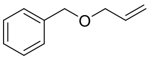

1-[(Prop-1-enyloxy)methyl]benzene: Obtained following a literature[S16] procedure from 3.2 g (30 mmol) benzyl alcohol and 2.4 g of (16 mmol) of allyl bromide. The product was isolated by flash column chromatography on silica gel (eluting with pentane:AcOEt 95:5) to give 4.2 g (28.3 mmol, 95%) of **1-[(prop-1-enyloxy)methyl]benzene** as yellow liquid. **^{1}H NMR** (400 MHz, $CDCl_3$): δ = 7.41 – 7.34 (m, 4H), 7.34 – 7.28 (m, 1H), 5.98 (ddt, *J*=17.3, 10.7, 5.7, 1H), 5.33 (dd, *J*=17.3, 1.8, 1H), 5.23 (dd, *J*=10.7, 1.5, 1H), 4.55 (s, 2H), 4.05 (dt, *J*=5.7, 1.5, 2H); **^{13}C{^{1}H} NMR** (151 MHz, $CDCl_3$): δ = 138.4, 134.9, 128.5, 127.9, 127.7, 117.2, 72.2, 71.3.The spectroscopic data are in agreement with the previously reported ones.[S17-S18]

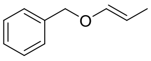

1-[(Prop-1-enyloxy)methyl]benzene (13): Obtained following a literature[S19] procedure from 2.4 g (16 mmol) **1-[(prop-1-enyloxy)methyl]benzene**. The product was isolated by flash column chromatography on silica gel (eluting with pentane:AcOEt 97:3) to give 1.5 g (10.1 mmol, 66%) of **13** as colourless liquid. **^{1}H NMR** (400 MHz, $CDCl_3$): δ = 7.21 – 7.09 (m, 4H), 7.08 – 7.03 (m, 1H), 5.88 (dd, J=6.2, 1.7, 1H), 4.44 (s, 3H), 4.45 – 4.37 (m, 1H), 1.73 (dd, J=6.8, 1.7, 4H); **^{13}C{^{1}H} NMR** (101 MHz, $CDCl_3$): δ = 145.8, 138.4, 128.6, 127.9, 127.5, 101.6, 73.5, 9.7. The spectroscopic data are in agreement with the previously reported ones.[S20]

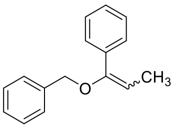

1-[(Prop-1-enyloxy)methyl]benzene (14): Obtained following GP1 starting from 148.2 mg (1 mmol) of 1-[(prop-1-enyloxy)methyl]benzene (**13**) and 180 µL (1.1 mmol) of PhOTf (**2a**). Flash column chromatography on silica gel (eluting with pentane with 1% v/v TEA) led to 155.5 mg (0.69 mmol, 69%) of **14** as slightly yellow oil. **^{1}H NMR** (400 MHz, C_6D_6): α-regioisomer, minor stereoisomer: δ = 5.26 (q, *J*=6.8, 1H), 4.56 (s, 2H), 1.69 (d, *J*=6.8, 3H); α-regioisomer, major stereoisomer: δ = 4.78 (q, *J*=7.0, 1H), 4.63 (s, 2H), 1.65 (d, *J*=7.0, 2H); **^{13}C{^{1}H} NMR** (101 MHz, $CDCl_3$): α-regioisomer, minor stereoisomer: δ = 154.7, 138.5, 137.0, 110.0, 72.2, 13.1; α-regioisomer, major stereoisomer : δ = 155.2, 138.3, 136.8, 96.4, 69.8, 11.3; **IR** (film): $\tilde{v}$= 3060, 3032,2922, 2861, 1654, 1601, 1494, 1449, 1363, 1312, 1260, 1226, 1125, 1069, 1025, 914, 797, 767, 734, 696 cm^{-1}; **HRMS** (ESI): *m/z* calcd for $C_{16}H_{16}O+Na^+$: 247.1099 [M+Na]$^+$; found: 247.1086.

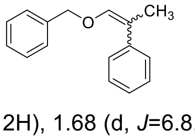

Benzyl 2-phenylprop-1-enyl ether (15): Obtained following GP2 starting from 149.2 mg (1 mmol) 1-[(prop-1-enyloxy)methyl]benzene **13** and 1255 µL of (1.1 mmol) of iodobenzene (**4a**). Flash column chromatography on silica gel (eluting with pentane with 1% v/v AcOEt and 1% v/v TEA) led to 163.9 mg (0.7 mmol, 73%) of **15** as slightly yellow oil. **^{1}H NMR** (400 MHz, C_6D_6): α-regioisomer, major stereoisomer: δ = 5.26 (q, *J*=6.9, 1H), 4.55 (s, 2H), 1.68 (d, *J*=6.8, 3H); α-regioisomer, minor stereoisomer: δ = 4.76 (q, *J*=7.0, 1H), 4.62 (s, 2H), 1.64 (d, *J*=7.0, 2H); β-regioisomer: δ = 6.45 (q, *J*=1.4, 1H), 4.49 (s, 2H), 2.11 (d, *J*=1.4, 3H); **^{13}C{^{1}H} NMR** (101 MHz, $CDCl_3$): α-regioisomer, major stereoisomer: δ = 154.7, 138.5, 137.0, 110.0, 72.2, 13.1; α-regioisomer, minor stereoisomer : δ = 155.2, 138.3, 136.8, 96.4, 69.7, 11.4; β-regioisomer: δ = 143.5, 141.7, 141.1, 138.2, 115.3, 74.0; **IR** (film): $\tilde{v}$= 3031, 2921, 2863, 2329, 1652,1598, 1492, 1448, 1369, 1312, 1142, 1071, 1025, 735, 695 cm^{-1}.

14. Computational Details

All the Density Functional Theory (DFT) calculations were carried out using Gaussian16 program package.[S21] The structures were optimized with the M06L functional[S22,S23] in combination with the Def2SVP basis set.[S24,S25] Frequency calculations were performed at the same level of theory to characterize the nature of stationary points as minima (no imaginary frequencies) or transition states (one imaginary frequency) and to calculate the thermochemistry corrections. Transition states were verified by relaxation towards reactants and products and by IRC calculations when needed. Potential energies were further refined using a larger basis set (Def2TZVPP) and the same functional. Solvation effects were introduced through the SMD implicit solvent model (N,N-dimethylformamide as the solvent) both in optimization-frequency and single point calculations.[S26] Final free energies were calculated combining the single point energy at M06L/Def2TZVPP plus thermochemistry corrections at 353.15 K (80 ºC) and 1M standard state.

XYZ Coordinates and Energies of the Calculated Species

Pd+

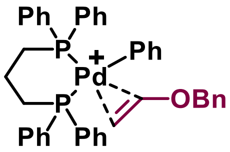

E = -2511.442508

G_{Corr}= 0.6074696

P	-0.85360200	-1.60385000	1.03898700
C	0.08820600	-2.87544000	1.97558800
H	-0.58744500	-3.21790500	2.77532700
H	0.23546600	-3.73670400	1.30498000
C	1.41681100	-2.42967000	2.56297300
H	1.72622900	-3.18738100	3.29879500
H	1.28665400	-1.50024800	3.14454500
C	2.54230600	-2.28382100	1.55071100
H	2.61745400	-3.19574900	0.93596900
H	3.51728400	-2.18301600	2.05243200
P	2.37699900	-0.90453000	0.35373800
Pd	0.24436400	-0.66375100	-0.75357900
C	2.71291400	0.61974300	1.29517700
C	2.44720000	1.84563300	0.66371400
C	3.19292000	0.62302500	2.61160200
C	2.67105000	3.04855300	1.32886300
H	2.05755900	1.85271200	-0.36076100
C	3.39894900	1.82977500	3.28182900
H	3.41155900	-0.31390300	3.12942000
C	3.14217000	3.04214600	2.64322000
H	2.47226800	3.99582900	0.82131000
H	3.76744500	1.81894400	4.31031600
H	3.30844800	3.98482900	3.17005200
C	3.84569000	-1.15826100	-0.70349400
C	3.82529300	-2.23733300	-1.60096500
C	4.96323700	-0.31512800	-0.67225300
C	4.90964700	-2.47555700	-2.44147100
H	2.94876800	-2.89232800	-1.64704100
C	6.04436600	-0.55304600	-1.52268600
H	4.99663300	0.53320900	0.01690000
C	6.02124400	-1.63120100	-2.40597700
H	4.88359300	-3.32053000	-3.13371700
H	6.91132400	0.11132400	-1.49086600
H	6.86877500	-1.81339000	-3.07096400
C	-1.23432900	-0.34273300	2.29744100
C	-1.89905100	-0.69642700	3.48224500
C	-0.79369800	0.97553000	2.12249500
C	-2.11325600	0.25685900	4.47445800
H	-2.25239800	-1.72169600	3.62997700
C	-1.00336000	1.92592000	3.12250600
H	-0.27794500	1.25123400	1.19487100
C	-1.66168500	1.56670600	4.29772000
H	-2.63252300	-0.02481900	5.39347400
H	-0.64957200	2.95026900	2.98202300
H	-1.82557000	2.31026500	5.08133100
C	-2.41626500	-2.47222800	0.68076800
C	-2.36912300	-3.74926900	0.10142800
C	-3.66005900	-1.85850900	0.88131900
C	-3.54622800	-4.40381700	-0.25635300
H	-1.40939700	-4.23735900	-0.09011400
C	-4.83463200	-2.51525900	0.51612900
H	-3.71665300	-0.85773800	1.31825900
C	-4.78144900	-3.78837700	-0.05140600
H	-3.49569000	-5.39943200	-0.70345700
H	-5.79801500	-2.02471700	0.67605000
H	-5.70336800	-4.30060600	-0.33680800
C	-1.66893800	-0.42182900	-1.51207900
C	-2.17697800	-1.35107800	-2.43078400
C	-2.49042100	0.63694700	-1.10725100
C	-3.47497300	-1.22401300	-2.93268000
H	-1.56104200	-2.19568600	-2.76107200
C	-3.78862100	0.76953400	-1.61414100
H	-2.13124000	1.37494400	-0.38082900
C	-4.28631600	-0.16177400	-2.52505900
H	-3.85572500	-1.96175900	-3.64570600
H	-4.41530200	1.60420300	-1.28440100
H	-5.30319000	-0.06440000	-2.91434200
C	1.44477300	0.04592500	-2.61753100
H	1.42427500	-0.95624800	-3.05454300
H	2.40959200	0.44291900	-2.29204600
C	0.44619500	0.90973700	-2.97583500
H	-0.44466700	0.56480100	-3.51452100
O	0.52939200	2.20988100	-2.76151700
C	-0.60574400	3.02734800	-3.08848200
H	-1.36798500	2.41832300	-3.60009400
H	-0.24661400	3.77433800	-3.81480900
C	-1.16912900	3.68818000	-1.86759600
C	-0.36371000	3.97233300	-0.75948400
C	-2.52388700	4.03744800	-1.83129700
C	-0.90909300	4.57500700	0.37410600
H	0.69814500	3.71918700	-0.78658800
C	-3.06802200	4.64287900	-0.69986000
H	-3.16104400	3.81300600	-2.69177500
C	-2.26412700	4.90579800	0.41067700
H	-0.26940400	4.79120500	1.23399500
H	-4.13021600	4.89980300	-0.68082700
H	-2.69258300	5.37191600	1.30144200

Pd+TSα

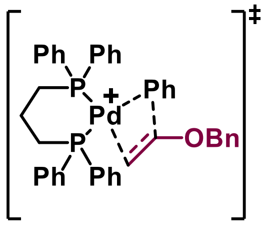

E = -2511.421051

G_{Corr}= 0.60874211

P	-0.73866100	-1.25111300	1.41099600
C	0.16415400	-2.24504900	2.66914700
H	-0.47098400	-2.31727800	3.56557800
H	0.22707600	-3.26659200	2.25886400
C	1.54375500	-1.72972200	3.04780200
H	1.89779100	-2.32423800	3.90422600
H	1.46371000	-0.69804300	3.43027500
C	2.61274800	-1.81514900	1.96677200
H	2.68238600	-2.84360300	1.57483100
H	3.60537300	-1.58990500	2.38837500
P	2.39503600	-0.75979200	0.48084600
Pd	0.38979300	-0.99152400	-0.66065900
C	2.58390500	0.96701300	1.03736200
C	2.20932600	1.99187100	0.15314900
C	3.07175900	1.30686100	2.30659800
C	2.33107700	3.32772100	0.52737300
H	1.79989900	1.73949600	-0.83086600
C	3.17024100	2.64632700	2.68624000
H	3.38609200	0.53213200	3.00994400
C	2.80384500	3.65734600	1.79889900
H	2.04141700	4.11634500	-0.17153200
H	3.54296300	2.89834100	3.68191300
H	2.88497500	4.70489600	2.09889000
C	3.90424900	-1.12872100	-0.47928700
C	3.93272200	-2.30689900	-1.24040100
C	5.02580400	-0.28995600	-0.47017600
C	5.06941100	-2.64552900	-1.97059900
H	3.05356400	-2.95953700	-1.26549400
C	6.15909400	-0.62867400	-1.21094700
H	5.02059100	0.63369500	0.11526900

C	6.18407800	-1.80465500	-1.95922900
H	5.08220300	-3.56660000	-2.55816500
H	7.02802600	0.03371400	-1.20093100
H	7.07248200	-2.06600100	-2.53911100
C	-0.96170500	0.39070000	2.17492600
C	-1.18447800	0.55091900	3.55111900
C	-0.88303000	1.52856600	1.35870900
C	-1.32030400	1.82609000	4.09670900
H	-1.25164600	-0.32346000	4.20502900
C	-1.02947700	2.80305200	1.90569600
H	-0.68360100	1.41214700	0.28587500
C	-1.24460200	2.95275000	3.27539500
H	-1.48697100	1.94101700	5.17044400
H	-0.96178800	3.68133900	1.25816200
H	-1.35005100	3.95121200	3.70675700
C	-2.38412700	-2.04045300	1.42133500
C	-2.47645500	-3.36477700	0.96581500
C	-3.54677200	-1.37710800	1.83602100
C	-3.70678900	-4.01849100	0.94448600
H	-1.57889300	-3.88810200	0.62010400
C	-4.77740600	-2.03329800	1.80563600
H	-3.49705600	-0.34057400	2.18112300
C	-4.86045200	-3.35317900	1.36245700
H	-3.76579000	-5.05049900	0.58973600
H	-5.67782000	-1.50628200	2.13080800
H	-5.82591400	-3.86441500	1.34016300
C	-1.43374800	-1.14158800	-1.84500300
C	-1.61118800	-2.41252700	-2.41532700
C	-2.56292000	-0.39135200	-1.49232800
C	-2.89207000	-2.93721200	-2.58828600
H	-0.74081900	-2.99541000	-2.73609600
C	-3.84498600	-0.91211900	-1.67417400
H	-2.44190000	0.60461200	-1.05111800
C	-4.01210300	-2.18666200	-2.21940800
H	-3.01871100	-3.93446000	-3.01892700
H	-4.71789200	-0.32179500	-1.37989900
H	-5.01624700	-2.59531600	-2.35940200
C	1.24439500	-0.57254200	-2.53765300
H	1.49033100	-1.51146600	-3.04595100
H	2.07986000	0.12941100	-2.44628300
C	-0.02580200	-0.00890300	-2.87638300
H	-0.55480500	-0.35125300	-3.77423400
O	-0.21058000	1.28399900	-2.56254000
C	-1.27318700	1.97813300	-3.23853700
H	-2.11759400	1.29347800	-3.41666300
H	-0.89412900	2.29942500	-4.22414500
C	-1.68617700	3.15023900	-2.40636100
C	-0.73971900	4.11419400	-2.03061000
C	-3.01130900	3.29994300	-1.98318100
C	-1.11392300	5.20646300	-1.25098900
H	0.29801500	4.00647600	-2.35985400
C	-3.38866500	4.39803900	-1.20843600
H	-3.75416200	2.54843300	-2.26712300
C	-2.44110400	5.35225400	-0.84009500
H	-0.36713000	5.95153000	-0.96438100
H	-4.42788400	4.50475400	-0.88786800
H	-2.73482700	6.21056700	-0.23073800

Pd^{+}$_{TS\beta}$

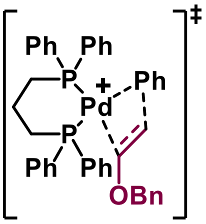

E = -2511.42038

G_{Corr}= 0.61229484

P	-1.50957500	0.20149200	0.93400500
C	-0.74643300	-0.00692700	2.59439600
H	-1.53254500	0.13890900	3.35107400
H	-0.44384200	-1.06375600	2.66951400
C	0.43972900	0.89779400	2.88740700
H	0.66078900	0.81602100	3.96270700
H	0.15806600	1.95324500	2.73321000
C	1.73000000	0.57852700	2.14371100
H	2.02012700	-0.47142100	2.32801200
H	2.55030900	1.19670100	2.54171100
P	1.71271000	0.76259200	0.32079200
Pd	-0.00136900	-0.43312600	-0.83906400
C	1.37618500	2.52706500	-0.02955000
C	0.94437200	2.87605700	-1.31838200
C	1.55779100	3.53631100	0.92594200
C	0.71624700	4.20926000	-1.65200300
H	0.77197300	2.08816800	-2.06158300
C	1.29877400	4.86724100	0.59752500
H	1.90760300	3.29293000	1.93252200
C	0.88444100	5.20647200	-0.69091900
H	0.38570400	4.46782600	-2.66098400
H	1.43214700	5.64516400	1.35320200
H	0.68842600	6.25106400	-0.94491200
C	3.47998600	0.61737700	-0.13926400
C	4.44360800	0.09829100	0.73592300
C	3.87769400	1.00766600	-1.42962800
C	5.77037800	-0.03646400	0.32366500
H	4.17362900	-0.20614900	1.74858400
C	5.20293600	0.87415700	-1.83579200
H	3.14525100	1.42847600	-2.12588200
C	6.15345900	0.34550900	-0.96059100
H	6.50908100	-0.44329600	1.01890900
H	5.49359500	1.18495700	-2.84203300
H	7.19301000	0.23766500	-1.27883300
C	-2.11458100	1.92283600	0.91801600
C	-2.55952800	2.55858900	2.08726700
C	-2.15639200	2.61939100	-0.29603200
C	-3.02668400	3.87040900	2.03785900
H	-2.54399900	2.03087200	3.04484700
C	-2.63594200	3.92813300	-0.34524700
H	-1.80082400	2.13109300	-1.20832300
C	-3.06705400	4.55632000	0.82242600
H	-3.36414300	4.35966900	2.95471000
H	-2.66163000	4.46151200	-1.29876200
H	-3.43457000	5.58477200	0.78733200
C	-3.02742700	-0.80459900	1.06560000
C	-2.93681000	-2.12740700	1.52824500
C	-4.27448000	-0.30981900	0.66028300
C	-4.07407500	-2.92782700	1.60538100
H	-1.96984400	-2.54273200	1.82670800
C	-5.40929900	-1.11858200	0.73059600
H	-4.36649600	0.71463600	0.28799900
C	-5.31363100	-2.42602800	1.20511700
H	-3.98940200	-3.95270900	1.97478800
H	-6.37557600	-0.71961500	0.41191300
H	-6.20476100	-3.05575400	1.26226900
C	-1.60729700	-1.18785800	-2.04682000
C	-2.08535200	-2.47156400	-1.74253000
C	-2.46572000	-0.27936500	-2.68416000
C	-3.40326600	-2.82328900	-2.03579700

H	-1.42281700	-3.21133800	-1.28131000
C	-3.78346900	-0.63474400	-2.97478500
H	-2.09837800	0.71081900	-2.97141200
C	-4.25872800	-1.90790300	-2.65247300
H	-3.76174500	-3.82535000	-1.78270300
H	-4.44100100	0.08965800	-3.46368500
H	-5.28999200	-2.18644700	-2.88335900
C	0.20945400	-1.17829500	-2.99992100
H	0.15074700	-0.25277900	-3.57792000
H	-0.17249000	-2.05506100	-3.52482500
C	1.38455500	-1.35509600	-2.23498800
H	2.22161900	-0.65066200	-2.31353500
O	1.71002200	-2.62659100	-1.89142200
C	2.84832100	-2.79313200	-1.06086100
H	3.56697400	-1.97008700	-1.22653000
H	3.34766400	-3.71036800	-1.41160500
C	2.51873800	-2.92977400	0.39962900
C	1.20833300	-3.07825800	0.86838700
C	3.57458900	-2.97490900	1.32062400
C	0.96467400	-3.26938400	2.23094100
H	0.37270300	-3.05859200	0.16209600
C	3.32983400	-3.15555900	2.67981100
H	4.60228500	-2.86958500	0.96044800
C	2.02016900	-3.30404100	3.14101400
H	-0.06113900	-3.40342100	2.58379600
H	4.16645200	-3.18535600	3.38256400
H	1.82420500	-3.45388100	4.20551000

$[Pd]^{+}_{Prod\alpha}$

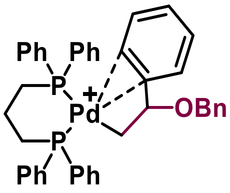

E = -2511.455177
GCorr= 0.6105524

P	-1.53341200	-1.28890500	1.02601800
C	-1.12904600	-2.67077600	2.17250300
H	-1.87236600	-2.67825800	2.98455100
H	-1.29350300	-3.59853000	1.60021300
C	0.27628100	-2.64707300	2.75080400
H	0.33732400	-3.44205500	3.50990700
H	0.43625400	-1.70834900	3.30754700
C	1.41078600	-2.88424700	1.76370300
H	1.22237000	-3.79105000	1.16561700
H	2.35480600	-3.07326800	2.29998100
P	1.76998700	-1.56576300	0.54140600
Pd	0.02855800	-1.03263400	-0.79612800
C	2.34170100	-0.11856600	1.48659500
C	2.40075200	1.12994200	0.84705800
C	2.73670200	-0.22341000	2.82823300
C	2.86193900	2.24807600	1.53844100
H	2.06362600	1.23139100	-0.19106200
C	3.17196700	0.90597600	3.52150500
H	2.71556400	-1.18618200	3.34410300
C	3.23974400	2.14059700	2.87766200
H	2.91249800	3.21515200	1.03295800
H	3.46763400	0.81468100	4.56924400
H	3.58578300	3.02303800	3.42123500
C	3.23800000	-2.21424500	-0.32400200
C	3.05933800	-3.26727800	-1.23365400
C	4.52223000	-1.69996300	-0.10788100
C	4.15290100	-3.80768500	-1.90475100
H	2.05449200	-3.65931900	-1.42251800
C	5.61364200	-2.23982900	-0.79027100
H	4.67784800	-0.87552800	0.59326200
C	5.43237800	-3.29276900	-1.68539200
H	4.00502300	-4.62968900	-2.60904500
H	6.61229500	-1.83130100	-0.61858300
H	6.28920100	-3.71179000	-2.21828300
C	-1.41324500	0.22329800	2.04423600
C	-1.82290000	0.25393600	3.38602300
C	-0.87241400	1.38330400	1.46968400
C	-1.68383000	1.41987000	4.13645300
H	-2.25432100	-0.63721800	3.85127300
C	-0.74260200	2.55194900	2.22008700
H	-0.52743100	1.36365400	0.42826400
C	-1.14380400	2.56953200	3.55541000
H	-1.99965500	1.43190000	5.18239900
H	-0.31258000	3.44635700	1.76044900
H	-1.03450800	3.48101100	4.14819200
C	-3.31955900	-1.54775200	0.74889900
C	-3.74652600	-2.77289600	0.21176300
C	-4.26831900	-0.54867500	1.00371700
C	-5.09705200	-2.99985000	-0.04220600
H	-3.01748200	-3.55654300	-0.01697300
C	-5.61939200	-0.77751000	0.74063100
H	-3.95524200	0.41874100	1.40642400
C	-6.03780400	-2.00231500	0.22196900
H	-5.41626000	-3.96067300	-0.45339100
H	-6.34925000	0.00970000	0.94531000
H	-7.09679900	-2.17982500	0.01978800
C	-0.97063700	0.18369900	-2.69507300
C	-1.40833300	-1.08528800	-3.12906900
C	-1.91138500	1.04436900	-2.09472500
C	-2.75248800	-1.45485300	-3.01337000
H	-0.70307700	-1.76659600	-3.61191700
C	-3.24889200	0.67389400	-1.99407800
H	-1.58673200	2.01174000	-1.70652200
C	-3.67582100	-0.57067700	-2.46366800
H	-3.07342200	-2.43887300	-3.36405400
H	-3.96605800	1.36090400	-1.53764600
H	-4.72747500	-0.85654000	-2.37882000
C	1.34604600	-0.52064300	-2.32540700
H	1.41783500	-1.39315800	-2.99171300
H	2.35443700	-0.18414200	-2.04710400
C	0.49887600	0.59107300	-2.89305300
H	0.69532400	0.75840100	-3.97478000
O	0.78365000	1.78036200	-2.17991500
C	0.51401700	2.97604100	-2.89917000
H	-0.42776500	2.89184300	-3.47070800
H	1.31951400	3.15110200	-3.63914300
C	0.42768300	4.10884100	-1.92293900
C	1.55539400	4.48158200	-1.17891800
C	-0.77738800	4.78705400	-1.70607400
C	1.47959900	5.50941400	-0.24085200
H	2.50278700	3.95941000	-1.34451800
C	-0.85474900	5.82100600	-0.77143500
H	-1.66401000	4.50224000	-2.28096500
C	0.27310300	6.18266100	-0.03523400
H	2.36783000	5.79156200	0.33071000
H	-1.80172400	6.34409400	-0.61629900
H	0.21436900	6.99134000	0.69756400

Pd

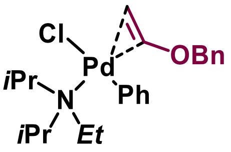

E = -1615.407682
GCorr=0.44215294

Pd	-0.30981000	-0.41559000	-0.77470900
C	0.18187800	1.51818800	-0.72489500
C	-0.16682200	2.48423700	-1.67488100
C	0.96502000	1.89651800	0.37590900
C	0.22476600	3.81706400	-1.50081900
H	-0.76279100	2.21875400	-2.55333400
C	1.35968300	3.22608100	0.54261100
H	1.25943300	1.15224800	1.12650600
C	0.98512300	4.19290300	-0.39356000

H	-0.06908500	4.56546000	-2.24285300
H	1.96499500	3.50519900	1.41072200
H	1.29152700	5.23392800	-0.26326200
C	0.43745800	-0.62715100	-2.75010100
H	0.09482000	0.27139000	-3.26866000
H	0.10314200	-1.58999000	-3.14422900
C	1.63322100	-0.55871700	-2.04350000
H	2.12472300	0.40968300	-1.87495600
O	2.38779300	-1.63615000	-1.81520100
C	3.64106500	-1.37741300	-1.16938800
H	4.23765500	-0.69019200	-1.79117900
H	4.15550900	-2.34876000	-1.17385900
C	3.50858000	-0.84819600	0.22693700
C	2.62432800	-1.44097700	1.13888900
C	4.30028500	0.22699800	0.64656700
C	2.56357700	-0.98309000	2.45474800
H	1.97458700	-2.25814100	0.80615000
C	4.23297400	0.68902000	1.96133300
H	4.97917400	0.70347700	-0.06694900
C	3.36789400	0.08015500	2.87032400
H	1.88125200	-1.45859300	3.16372200
H	4.85660200	1.53017400	2.27467600
H	3.31605700	0.43584000	3.90239200
Cl	-0.51621900	-2.92170900	-0.69470300
N	-2.26895900	0.02808000	0.64181900
C	-2.17263700	1.36054200	1.29691300
H	-1.40851800	1.27993200	2.08198400
H	-1.75500900	2.06909400	0.57145900
C	-3.45890700	-0.02445300	-0.26489200
H	-4.34732200	0.25105800	0.33590200
C	-2.22610600	-1.10514700	1.61970500
H	-2.24531200	-2.00219200	0.98753900
C	-3.32142500	0.98495700	-1.38946700
H	-3.11237500	2.00636600	-1.04373300
H	-2.51219600	0.68665300	-2.07638700
H	-4.25307000	1.02408500	-1.97109900
C	-3.71848300	-1.39808500	-0.84851700
H	-4.53599900	-1.32470900	-1.57932600
H	-2.82850300	-1.78765400	-1.36499600
H	-4.02626300	-2.14129400	-0.10095300
C	-0.89911900	-1.14259300	2.34740200
H	-0.77081300	-2.11686800	2.84130400
H	-0.06226300	-1.02763700	1.63938300
H	-0.80040700	-0.37054900	3.12596800
C	-3.38981600	-1.18698600	2.59651500
H	-3.38333200	-2.17458900	3.08070600
H	-3.31900500	-0.44126700	3.40137400
H	-4.36936600	-1.07518600	2.10994900
C	-3.43707200	1.98374800	1.86254400
H	-3.98209800	1.34852900	2.57061600
H	-3.15779600	2.90052200	2.40140000
H	-4.14337500	2.28843900	1.07718500

Pd$_{TS\alpha}$

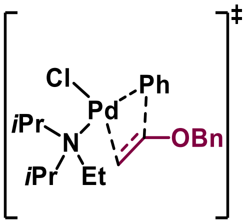

E = -1615.396371
G_{Corr}= 0.45750599

Pd	1.00323600	0.37255800	0.29025500
C	-0.92826700	1.12955400	0.43665600
C	-1.17457400	2.36485700	1.05453600
C	-1.68590500	0.76506700	-0.68705100
C	-2.12281600	3.23955800	0.52560300
H	-0.62024000	2.64275200	1.95679900
C	-2.62269500	1.64571100	-1.22564700

H	-1.54796500	-0.22404500	-1.13784500
C	-2.84289900	2.88508200	-0.61916100
H	-2.30657400	4.20170900	1.01163800
H	-3.19918900	1.35538800	-2.10937100
H	-3.59021000	3.56973100	-1.02848100
C	0.37362800	-1.02354000	1.65995200
H	1.06017500	-0.85008800	2.49772400
H	0.36951200	-2.04749900	1.26693500
C	-0.90622200	-0.41720500	1.80443400
H	-1.06734000	0.28088500	2.63581500
O	-1.97411300	-1.13835500	1.43327600
C	-3.25621000	-0.56233600	1.67899400
H	-3.17232300	0.53574200	1.74885700
H	-3.60660900	-0.91169700	2.66670100
C	-4.22859300	-0.94070400	0.60436600
C	-3.94027300	-1.92555400	-0.34552400
C	-5.46155200	-0.27782200	0.54267200
C	-4.86578800	-2.23535400	-1.34366000
H	-2.97931600	-2.44294700	-0.31155500
C	-6.38513300	-0.58946500	-0.45226000
H	-5.69002300	0.50055200	1.27718500
C	-6.08915000	-1.56937300	-1.40239800
H	-4.62503500	-3.00348300	-2.08310700
H	-7.33952800	-0.05830700	-0.49122900
H	-6.81051900	-1.81022900	-2.18704600
Cl	1.51175300	2.38619600	-1.11973000
N	3.06468000	-0.71297700	-0.08084200
C	3.34366000	-1.55123100	1.11096900
C	2.89236500	-1.54614300	-1.31611600
C	4.06733300	0.38930700	-0.27122100
H	3.28127800	-0.89776600	1.99221300
H	2.50717300	-2.24874700	1.21826500
C	4.63698700	-2.34295800	1.15548200
H	3.82092300	-2.13169800	-1.46257100
C	1.74663500	-2.53517100	-1.19205600
C	2.67060100	-0.69783000	-2.55427700
H	3.59170300	1.04885000	-1.00843900
C	4.21560400	1.20526300	0.99576800
C	5.42949200	-0.00944300	-0.82973000
H	5.51652900	-1.72271500	1.36844800
H	4.57062900	-3.08449300	1.96451400
H	4.83318400	-2.90139600	0.22883800
H	1.84366500	-3.23308200	-0.35056300
H	0.78077300	-2.01030500	-1.09884300
H	1.70056900	-3.14722700	-2.10354800
H	2.47882000	-1.35582100	-3.41302400
H	1.79786600	-0.03947100	-2.42334000
H	3.52693900	-0.06558800	-2.82114100
H	4.72992400	2.15018800	0.76943400
H	3.23143700	1.45950600	1.42222900
H	4.80810300	0.69356600	1.76968600
H	5.93932100	0.90014300	-1.18066600
H	6.09283700	-0.47447900	-0.08946800
H	5.36763900	-0.68486900	-1.69365900

Pd$_{TS\beta}$

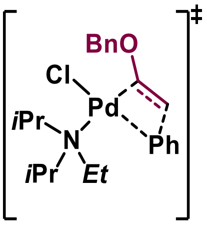

E = -1615.385513
G_{Corr}= 0.448386

Pd	-0.19610800	-0.42921300	-0.83983600
C	-1.54714800	-1.72975200	0.06315800
C	-1.25649000	-2.19724800	1.35649400
C	-2.88914200	-1.69460100	-0.35214600
C	-2.28473200	-2.50780200	2.24394700

H	-0.21595000	-2.31149400	1.67446000
C	-3.91661000	-2.01179800	0.53786600
H	-3.13403000	-1.41443200	-1.38081600
C	-3.62042500	-2.40582000	1.84373400
H	-2.03975100	-2.83949800	3.25703200
H	-4.95597900	-1.95799400	0.20188400
H	-4.42463400	-2.65635100	2.53982600
C	-0.42154800	-2.57767200	-1.39340300
H	-1.11580400	-2.59370000	-2.23690800
H	-0.45140500	-3.48365000	-0.78479900
C	0.86752000	-2.04380200	-1.65142800
H	1.13783200	-1.65975200	-2.64310600
O	1.89577800	-2.49531800	-0.89823300
C	3.12451500	-1.78192300	-1.01565100
H	3.16144300	-1.24357600	-1.97567300
H	3.91873500	-2.54604100	-1.03546500
C	3.34969300	-0.83642500	0.12848000
C	2.74522100	-1.03992700	1.37376700
C	4.22441400	0.24596600	-0.02945600
C	3.01867700	-0.18529900	2.44326100
H	2.05778300	-1.87927700	1.50697400
C	4.49920500	1.09803100	1.03825100
H	4.68800500	0.42339600	-1.00439500
C	3.89837400	0.88404000	2.28102700
H	2.53495700	-0.35631700	3.40902400
H	5.18144700	1.94061800	0.89799200
H	4.11128400	1.55452500	3.11751300
Cl	1.30315100	0.95419700	-2.28469500
N	-1.24256200	1.54096300	0.20166300
C	-2.48962700	1.67613000	-0.63178500
C	-3.65625400	2.44610700	-0.01808100
H	-2.84592500	0.63685000	-0.71898800
H	-4.50009200	2.40945900	-0.72241200
H	-4.01609200	2.00205700	0.91954700
H	-3.44535100	3.50684200	0.16963500
C	-1.63049400	1.18337300	1.58031300
C	-0.53246700	0.68409300	2.49281900
H	-2.39551800	0.39657500	1.47739900
H	-2.14901400	2.02895200	2.06928300
H	-0.97601800	0.09574600	3.30921400
H	0.17333100	0.03023000	1.95997000
H	0.04280800	1.49180800	2.96189500
C	-0.30611000	2.70245400	0.08558800
C	-0.84828500	4.02574400	0.60772000
H	-0.14219900	2.79998300	-0.99563600
H	-0.04998600	4.78205500	0.58109400
H	-1.67644500	4.41994100	0.00548600
H	-1.18600300	3.95871500	1.65386200
C	1.06740700	2.43316100	0.68347600
H	1.38160300	1.38976000	0.53947900
H	1.80903600	3.06456000	0.17242900
H	1.12447400	2.67857900	1.75271100
C	-2.19640700	2.16202100	-2.03565500
H	-2.00195000	3.24472400	-2.07804200
H	-1.32956100	1.64204300	-2.47029200
H	-3.06764000	1.96863000	-2.67744200

Pd^{iso}

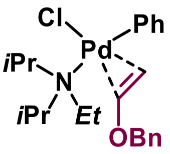

E = -1615.396371
G_{Corr}= 0.444082

Pd	-0.07838900	0.62215700	-0.11539300
C	1.66481000	1.59108700	-0.04214300
C	1.76615900	2.88094300	-0.56816700
C	2.78158700	0.98764700	0.54412600
C	2.99010700	3.56024500	-0.51429000
H	0.89900100	3.37565700	-1.01679800
C	4.00108200	1.66730800	0.58407200
H	2.70450400	-0.01282600	0.97938000
C	4.11021100	2.95523400	0.05386400
H	3.06044700	4.57176200	-0.92479600
H	4.87029800	1.18340200	1.04022600
H	5.06479400	3.48625000	0.08833200
C	-0.15556400	0.72519800	-2.27461200
H	0.06449500	1.77973200	-2.45980000
H	-1.11215900	0.35701500	-2.65596100
C	0.92046300	-0.13989300	-2.14721900
H	1.94361000	0.24512600	-2.04701200
O	0.82796100	-1.44853600	-2.39663000
C	1.99511700	-2.22305000	-2.09671800
H	2.86969300	-1.78075500	-2.60094100
H	1.80978000	-3.19829400	-2.57035400
C	2.24495500	-2.37218500	-0.62538700
C	1.18552600	-2.40407500	0.29091300
C	3.55609100	-2.50217300	-0.15249800
C	1.43546600	-2.56177900	1.65391400
H	0.15600700	-2.30246200	-0.06635500
C	3.80558000	-2.66872000	1.20936200
H	4.38898300	-2.46575100	-0.86094400
C	2.74536300	-2.69446500	2.11688300
H	0.59997800	-2.58005500	2.35868400
H	4.83433900	-2.76712200	1.56485900
H	2.93979800	-2.81618100	3.18526700
Cl	-0.13536200	1.08662400	2.27268700
N	-2.63558600	-0.13611900	0.11521300
C	-2.55014000	-1.09572600	1.23802900
H	-2.30531400	-2.07613100	0.80192200
H	-1.67838900	-0.81175300	1.84476400
C	-3.18472100	1.19841000	0.49968700
H	-2.77092900	1.36660600	1.50795100
C	-3.20458100	-0.73576200	-1.12140900
H	-3.23750600	0.09285600	-1.85054000
C	-2.66411100	2.31244300	-0.39712300
H	-1.58039000	2.47602500	-0.29223000
H	-2.87619600	2.12457800	-1.46111500
H	-3.15589000	3.26152300	-0.13822900
C	-4.70406700	1.34367400	0.58594600
H	-4.93765500	2.28276000	1.10921000
H	-5.16277500	1.42063500	-0.41126800
H	-5.20817600	0.53834300	1.13047400
C	-2.29017500	-1.82061600	-1.66941000
H	-2.46233900	-1.96975300	-2.74524200
H	-1.23223400	-1.58075200	-1.53112000
H	-2.47262400	-2.79220500	-1.18529800
C	-4.60610100	-1.34025700	-1.02850300
H	-4.89739700	-1.70682000	-2.02441600
H	-4.61637500	-2.21617400	-0.36307200
H	-5.38969900	-0.65152200	-0.69959700
C	-3.72750700	-1.24556500	2.18593600
H	-4.66614500	-1.54550000	1.70086900
H	-3.48444300	-2.02251100	2.92613500
H	-3.91991100	-0.32392900	2.75441500

$Pd^{iso}{}_{TS\alpha}$

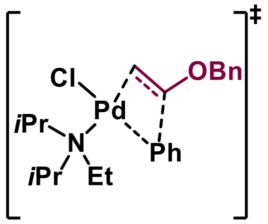

E = -1615.377392
G_{Corr}= 0.4430609

Pd	-1.08507200	1.09691900	0.34675100
C	0.49643700	-0.17980600	0.93666900
C	0.39278600	-0.91927700	2.12811500
C	1.23801700	-0.72406900	-0.12415600
C	0.92398400	-2.20529600	2.21569300
H	-0.10341200	-0.47986000	2.99925800
C	1.75634300	-2.01581300	-0.04709100
H	1.42742200	-0.12044000	-1.01681600
C	1.59190800	-2.76346000	1.12171900
H	0.82610400	-2.77236000	3.14561800
H	2.31255400	-2.43088700	-0.89343100
H	2.01215600	-3.76998000	1.19250800
C	0.14418600	2.60023200	0.96759800
H	-0.41303000	3.06424200	1.78854200
H	0.35551100	3.26513300	0.12188200
C	1.19703400	1.72855900	1.36063200
H	1.29238400	1.47239800	2.42265100
O	2.34449000	1.78081200	0.67256600
C	3.42933900	1.00996900	1.19217000
H	3.04328500	0.18680100	1.81725400
H	4.01881500	1.66083400	1.86231000
C	4.28576200	0.46710300	0.09047700
C	4.17431400	0.91399900	-1.22974900
C	5.22750400	-0.52473000	0.39504400
C	4.98537700	0.37183000	-2.22840700
H	3.43884800	1.68168400	-1.47911400
C	6.03913100	-1.06166100	-0.60125900
H	5.31322800	-0.88581400	1.42453600
C	5.91892100	-0.61651100	-1.91958700
H	4.88358900	0.72607700	-3.25742000
H	6.76471800	-1.83911000	-0.34910900
H	6.55069200	-1.04164200	-2.70329500
Cl	-2.69681300	2.88515700	-0.15775300
N	-2.63365500	-0.68866900	-0.43898200
C	-3.97497900	-0.20932700	-0.03563200
C	-2.53353400	-0.83078000	-1.92443600
C	-2.21331700	-1.94499900	0.26957000
H	-3.95016100	-0.01256900	1.04467600
H	-4.09844500	0.78299100	-0.48181600
C	-5.17167500	-1.07982200	-0.37000600
H	-3.13500200	-1.71067400	-2.22619300
C	-3.09048700	0.36677200	-2.67206800
C	-1.10394400	-1.06794000	-2.36545500
H	-1.11436100	-1.95605200	0.20517700
C	-2.57515400	-1.88567800	1.74116700
C	-2.67896400	-3.26408600	-0.34039100
H	-5.22440300	-1.99653600	0.23349400
H	-6.09532900	-0.51665600	-0.17047300
H	-5.20310500	-1.37560900	-1.42924700
H	-4.18093300	0.46609500	-2.58789600
H	-2.63952300	1.30456000	-2.31510400
H	-2.86312100	0.25924700	-3.74197000
H	-1.07727600	-1.27877300	-3.44407800
H	-0.49597600	-0.16688000	-2.18731600
H	-0.61515300	-1.91029400	-1.85850600
H	-2.01932700	-2.65592000	2.29371200
H	-2.32208300	-0.91121200	2.18449000
H	-3.64583300	-2.07071200	1.91654200
H	-2.31190700	-4.08729100	0.29043600
H	-3.77130900	-3.36348500	-0.39511900
H	-2.27475500	-3.44029800	-1.34626800

$Pd^{iso}{}_{TS\beta}$

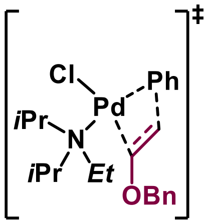

E = -1615.376068
G_{Corr}= 0.44554504

Pd	0.69249800	0.28719600	0.08777300
C	2.50411800	-0.65284400	0.41798100
C	2.61377600	-2.03725100	0.21619200
C	3.63266700	0.15914600	0.22513500
C	3.81016400	-2.58598900	-0.24264600
H	1.76185100	-2.69152300	0.42381200
C	4.82879700	-0.39854600	-0.22586600
H	3.57002700	1.23608400	0.41168800
C	4.92192900	-1.77044900	-0.47052800
H	3.87539700	-3.66346500	-0.41852000
H	5.69589400	0.24793900	-0.38794700
H	5.85988600	-2.20448300	-0.82526400
C	1.53352200	-0.14714800	2.11148200
H	1.90782800	0.83240500	2.41788200
H	2.08571300	-0.97495800	2.55990700
C	0.12568700	-0.32060200	2.02961800
H	-0.58536400	0.45376000	2.34406300
O	-0.31919200	-1.59545600	2.14830800
C	-1.72019700	-1.81470400	2.10230200
H	-2.25858900	-0.88914700	2.37372500
H	-1.94478900	-2.54185500	2.89921500
C	-2.19173400	-2.34898200	0.78064100
C	-1.30790700	-2.64256100	-0.26085300
C	-3.56068300	-2.59019200	0.59685500
C	-1.78123900	-3.17057600	-1.46384600
H	-0.24040700	-2.44281500	-0.13786100
C	-4.03257600	-3.11632600	-0.60298900
H	-4.26006300	-2.36349700	1.40752400
C	-3.14220900	-3.41074200	-1.63940800
H	-1.07602700	-3.38728600	-2.27081300
H	-5.10263300	-3.29822400	-0.73194000
H	-3.51224400	-3.82406100	-2.58091300
Cl	1.18947100	0.20688400	-2.37763400
N	-1.08768800	1.97089500	-0.08107200
C	-0.80691400	2.72487500	-1.35133100
C	0.51293500	3.46728000	-1.30199500
C	-1.93456800	3.65253600	-1.78413900
H	-0.69106500	1.94506200	-2.11627400
H	1.33874700	2.80140200	-1.01065100
H	0.74629900	3.85472300	-2.30419700
H	0.49633400	4.33108400	-0.61978400
H	-2.88991900	3.13021500	-1.93188900
H	-2.11116400	4.47762600	-1.07736800
H	-1.67306200	4.11359400	-2.74718400
C	-1.10173400	2.93467600	1.08947700
C	0.24206400	3.02246400	1.80459700
C	-2.20363900	2.70802500	2.11141900
H	-1.30042000	3.93108900	0.65879700
H	1.09816300	2.86244600	1.13698800
H	0.35876500	4.01672800	2.26144400
H	0.31839400	2.29851700	2.62886200
H	-3.21361300	2.82222200	1.69931500
H	-2.14789200	1.72498600	2.60219300
H	-2.09576800	3.46118500	2.90479500
C	-2.34802700	1.20514900	-0.20705000
C	-2.41626300	0.33650300	-1.43898700
H	-3.22535200	1.87809200	-0.19056700
H	-2.43806500	0.57712400	0.69144100

H	-3.23392800	-0.39131000	-1.33646300
H	-2.61070400	0.91220300	-2.35533000
H	-1.47859200	-0.21896500	-1.59176700

Pd⁻

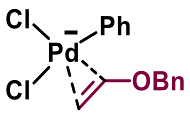

E = -1704.659363
G_{Corr}= 0.1944334

Pd	-0.61068300	1.08726900	-0.09173400
C	-1.64677000	-0.61448400	0.10269900
C	-2.95311000	-0.65065000	0.60692800
C	-1.03561400	-1.82234500	-0.26555300
C	-3.63383900	-1.86769500	0.73502200
H	-3.46130900	0.27384600	0.90067300
C	-1.71150500	-3.03676000	-0.12710700
H	-0.01744400	-1.81980800	-0.67079900
C	-3.01566900	-3.06466600	0.37358400
H	-4.65653600	-1.87533200	1.12446700
H	-1.21370000	-3.96787500	-0.41658900
H	-3.54636200	-4.01447400	0.47977900
C	-0.78852800	1.55136200	1.97936100
H	-1.79588200	1.24386900	2.27120000
H	-0.52031700	2.59988100	2.12930800
C	0.21156100	0.58607700	1.95906200
H	-0.03299400	-0.47745400	2.09444900
O	1.50804800	0.90070900	2.11294500
C	2.40799700	-0.20967000	2.07913800
H	2.14174900	-0.92674500	2.87402600
H	3.38555000	0.21751600	2.34742200
C	2.46919100	-0.89703300	0.74683500
C	2.40917600	-0.15721400	-0.44202400
C	2.60444100	-2.28824400	0.67795800
C	2.49051300	-0.80275400	-1.67537800
H	2.27839200	0.92945600	-0.39429100
C	2.69414800	-2.93234400	-0.55658100
H	2.63679400	-2.87182100	1.60301800
C	2.63523400	-2.19011700	-1.73675600
H	2.42990500	-0.21708600	-2.59640300
H	2.79877200	-4.01964100	-0.59654800
H	2.69626900	-2.69310700	-2.70517400
Cl	0.67647800	3.22733500	-0.33972700
Cl	-1.02466600	0.93607400	-2.47624100

Pd⁻ TSα

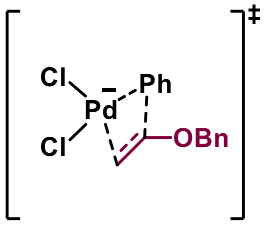

E = -1704.625774
G_{Corr}= 0.19350026

Pd	-2.06292200	-0.29088000	-0.00123800
C	-0.31113900	0.73936800	-0.49213300
C	-0.39093300	1.80273000	-1.40533800
C	0.57810200	0.83954600	0.58877100
C	0.35774400	2.96213000	-1.20802400
H	-1.04682800	1.71973900	-2.27850300
C	1.32106600	2.00115600	0.79260300
H	0.69388600	-0.00844600	1.27184500
C	1.21240600	3.06574000	-0.10613300
H	0.28294200	3.78750100	-1.92164600
H	2.00186000	2.07030600	1.64687900
H	1.80638400	3.97130600	0.04235900
C	-1.30908500	-1.86443100	-1.05174100
H	-2.03091400	-2.04315200	-1.85659500
H	-1.11256700	-2.73270900	-0.41057900
C	-0.17474200	-1.06788400	-1.39781700
H	-0.16620000	-0.60915700	-2.39551500
O	1.02721100	-1.50207600	-0.95490400
C	2.17196200	-0.82674200	-1.46347300
H	1.90560500	0.19599000	-1.78257600
H	2.51262400	-1.35473600	-2.37307400
C	3.26785500	-0.77411700	-0.44311500
C	3.20084700	-1.48703900	0.75799700
C	4.39020800	0.02641900	-0.69493900
C	4.23286700	-1.39257400	1.69350200
H	2.32767700	-2.10858800	0.96615500
C	5.42037200	0.11819800	0.23776200
H	4.44680500	0.59293400	-1.62957600
C	5.34419300	-0.59016100	1.43913300
H	4.16400400	-1.95057600	2.63094300
H	6.28569300	0.75250400	0.02916000
H	6.14901600	-0.51419200	2.17444800
Cl	-2.86216300	1.72912600	1.20960600
Cl	-4.04452100	-1.59298800	0.56099000

Pd⁻ TSβ

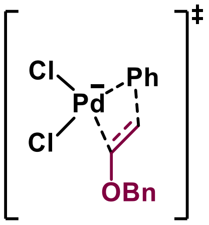

E = -1704.6309
G_{Corr}= 0.19592737

Pd	0.43435500	0.62380700	-0.49287600
C	2.18322900	0.10033700	0.50209400
C	2.15815000	-1.06789000	1.28361500
C	3.39405400	0.48031100	-0.10083000
C	3.29112200	-1.87254900	1.39123400
H	1.24338500	-1.35187700	1.81328700
C	4.52709900	-0.32380600	0.01582100
H	3.44193200	1.40462900	-0.68469800
C	4.48078900	-1.50850100	0.75438100
H	3.24591900	-2.79040900	1.98448700
H	5.45540300	-0.02173600	-0.47768800
H	5.36978600	-2.13757700	0.84533100
C	1.25445600	1.65709300	1.29310700
H	1.87850800	2.48306800	0.94233000
H	1.54634600	1.28755900	2.27963200
C	-0.15094300	1.81228300	1.09731400
H	-0.56197700	2.70998100	0.61575000
O	-0.96916000	1.22263900	2.01433200
C	-2.34949100	1.18749800	1.68719500
H	-2.60578000	2.03462400	1.02824200
H	-2.89479400	1.33756300	2.63329500
C	-2.77259600	-0.10274300	1.04457200
C	-1.91112800	-1.19942900	0.95926700
C	-4.06380400	-0.21519400	0.51522800
C	-2.32354200	-2.38071300	0.34057600
H	-0.90421000	-1.11896500	1.37411100
C	-4.47967600	-1.39511500	-0.09753400
H	-4.74472700	0.63985600	0.57247900
C	-3.60747600	-2.48247200	-0.19338000
H	-1.63159400	-3.22473300	0.27243000
H	-5.48874400	-1.46490600	-0.51237800
H	-3.93041800	-3.40538600	-0.68163400
Cl	-1.53044500	1.52687900	-1.70881000
Cl	0.97588500	-0.99703000	-2.31491200

15. References

[S1] R. Matake, Y. Adachi, H. Matsubara, *Green Chem.* **2016**, *18*, 2614–2618.
[S2] S. M. Wilkerson-Hill, S. Sawano, R. Sarpong, *J. Org. Chem.* **2016**, *81*, 11132–11144.
[S3] G. Werner, K. S. Rodygin, A. A. Kostin, E. G. Gordeev, A. S. Kashin, V. P. Ananikov, *Green Chem.* **2017**, *19*, 3032–3041.
[S4] H. Kusama, H. Funami, J. Takaya, N. Iwasawa, *Org. Lett.* **2004**, *6*, 605–608.
[S5] G. Dujardin, S. Rossignol, S. Molato, E. Brown, *Tetrahedron* **1994**, *50*, 9037–9050.
[S6] N. A. Harada, T. Nishikata, H. Nagashima, *Tetrahedron* **2012**, *68*, 3243–3252.
[S7] L. A. Oparina, O. V. Vysotskaya, A. V. Stepanov, I. V. Rodionova, G. F. Myachina, N. K. Gusarova, B. A. Trofimov, *Russ. J. Org. Chem.* **2008**, *44*, 120–123.
[S8] L. A. Oparina, O. V. Vysotskaya, A. V. Stepanov, N. K. Gusarova, B. A. Trofimov, *Russ. J. Org. Chem.* **2009**, *45*, 131–134.
[S9] F. De Nanteuil, E. Serrano, D. Perrotta, J. Waser, *J. Am. Chem. Soc.* **2014**, *136*, 6239–6242.
[S10] S. R. Sheng, H. R. Luo, W. K. Sun, X. L. Liu, Q. Xin, Q. Y. Wang, *Synth. Commun.* **2005**, *35*, 2839–2845.
[S11] J. Cuthbertson, J. D. Wilden, *Tetrahedron* **2015**, *71*, 4385–4392.
[S12] J. Barluenga, M. Escribano, P. Moriel, F. Aznar, C. Valdés, *Chem. Eur. J.* **2009**, *15*, 13291–13294.
[S13] M. Shi, Y. M. Shen, *J. Chem. Res. - Part S* **2002**, 422–427.
[S14] B. Ding, W. G. Bentrude, *J. Am. Chem. Soc.* **2003**, *125*, 3248–3259.
[S15] J. M. Dickinson, J. A. Murphy, C. W. Patterson, N. F. Wooster, *J. Chem. Soc. Perkin Trans. 1* **1990**, 1179–1184.
[S16] N. J. Estwood, N. S. Isambert (Univ St. Andrews), WO2005/121096, 2005, A2
[S17] Y. A. Lin, J. M. Chalker, N. Floyd, G. J. L. Bernardes, B. G. Davis, *J. Am. Chem. Soc.* **2008**, *130*, 9642–9643.
[S18] H. J. Zhong, B. R. Lee, J. W. Boyle, W. Wang, D. L. Ma, P. W. Hong Chan, C. H. Leung, *Chem. Commun.* **2016**, *52*, 5788–5791.
[S19] C. Lu, X. Su, P. E. Floreancig, *J. Org. Chem.* **2013**, *78*, 9366–9376.
[S20] V. Rautenstrauch, G. Büchi, H. Wiiestlb, *J. Am. Chem. Soc.* **1974**, *1701*, 2576–2580.
[S21] M. J. Frisch, G. W. Trucks, H. B. Schlegel, G. E. Scuseria, M. A. Robb, J. R. Cheeseman, G. Scalmani, V. Barone, G. A. Petersson, H. Nakatsuji, X. Li, M. Caricato, A. V. Marenich, J. Bloino, B. G. Janesko, R. Gomperts, B. Mennucci, H. P. Hratchian, J. V. Ortiz, A. F. Izmaylov, J. L. Sonnenberg, Williams, F. Ding, F. Lipparini, F. Egidi, J. Goings, B. Peng, A. Petrone, T. Henderson, D. Ranasinghe, V. G. Zakrzewski, J. Gao, N. Rega, G. Zheng, W. Liang, M. Hada, M. Ehara, K. Toyota, R. Fukuda, J. Hasegawa, M. Ishida, T. Nakajima, Y. Honda, O. Kitao, H. Nakai, T. Vreven, K. Throssell, J. A. Montgomery Jr., J. E. Peralta, F. Ogliaro, M. J. Bearpark, J. J. Heyd, E. N. Brothers, K. N. Kudin, V. N. Staroverov, T. A. Keith, R. Kobayashi, J. Normand, K. Raghavachari, A. P. Rendell, J. C. Burant, S. S. Iyengar, J. Tomasi, M. Cossi, J. M. Millam, M. Klene, C. Adamo, R. Cammi, J. W. Ochterski, R. L. Martin, K. Morokuma, O. Farkas, J. B. Foresman, D. J. Fox, **2016**, Gaussian 16, Revision C.01, Gaussian, Inc., Wallin.
[S22] Y. Zhao, D. G. Truhlar, *J. Chem. Phys.* **2006**, *125*, 194101.
[S23] Y. Zhao, D. G. Truhlar, *Acc. Chem. Res.* **2008**, *41*, 157–167.
[S24] F. Weigend, R. Ahlrichs, *Phys. Chem. Chem. Phys.* **2005**, *7*, 3297–3305.
[S25] F. Weigend, *Phys. Chem. Chem. Phys.* **2006**, *8*, 1057–1065.
[S26] A V Marenich, C. J. Cramer, D. G. Truhlar, *J. Phys. Chem. B* **2009**, *113*, 6378–6396.

16. NMR spectra

^{1}H-NMR (600 MHz, $CDCl_3$) spectrum of compound **1a** (literature procedure[1])

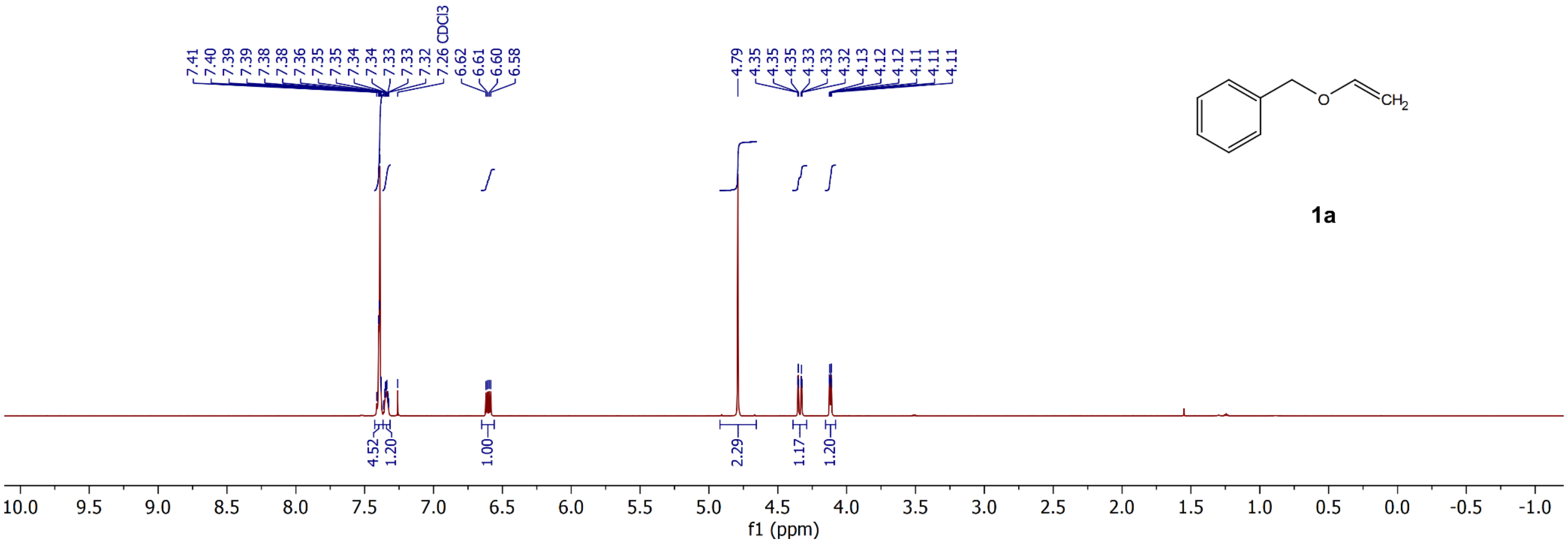

$^{13}C\{^1H\}$-NMR (151 MHz, $CDCl_3$) spectrum of compound **1a** (literature procedure[1])

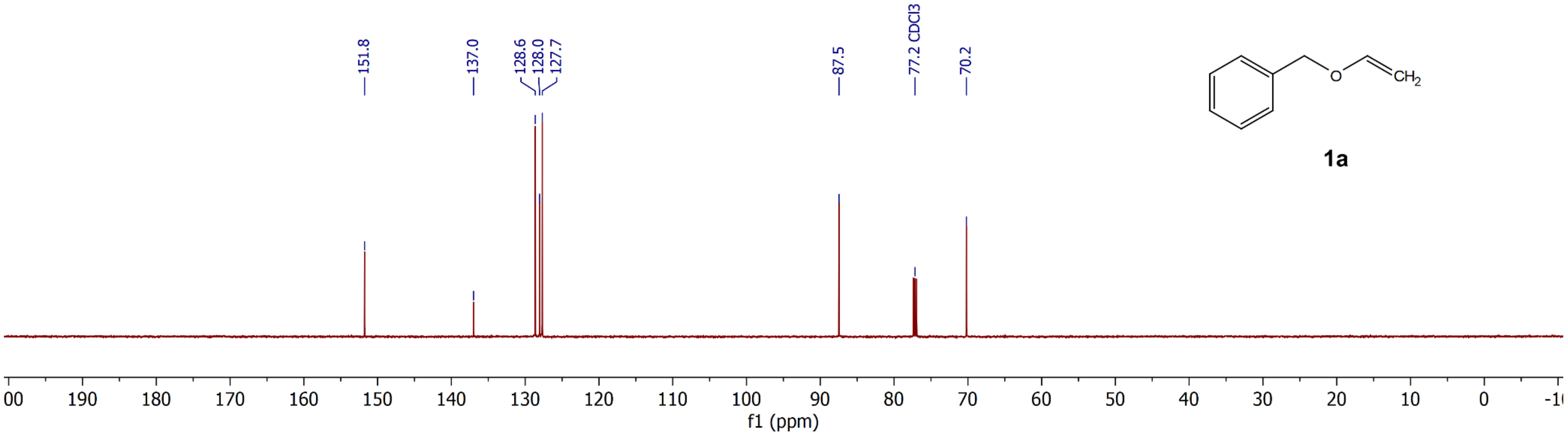

^{1}H-NMR (600 MHz, C_6D_6) spectrum of compound **1b** (literature procedure[1])

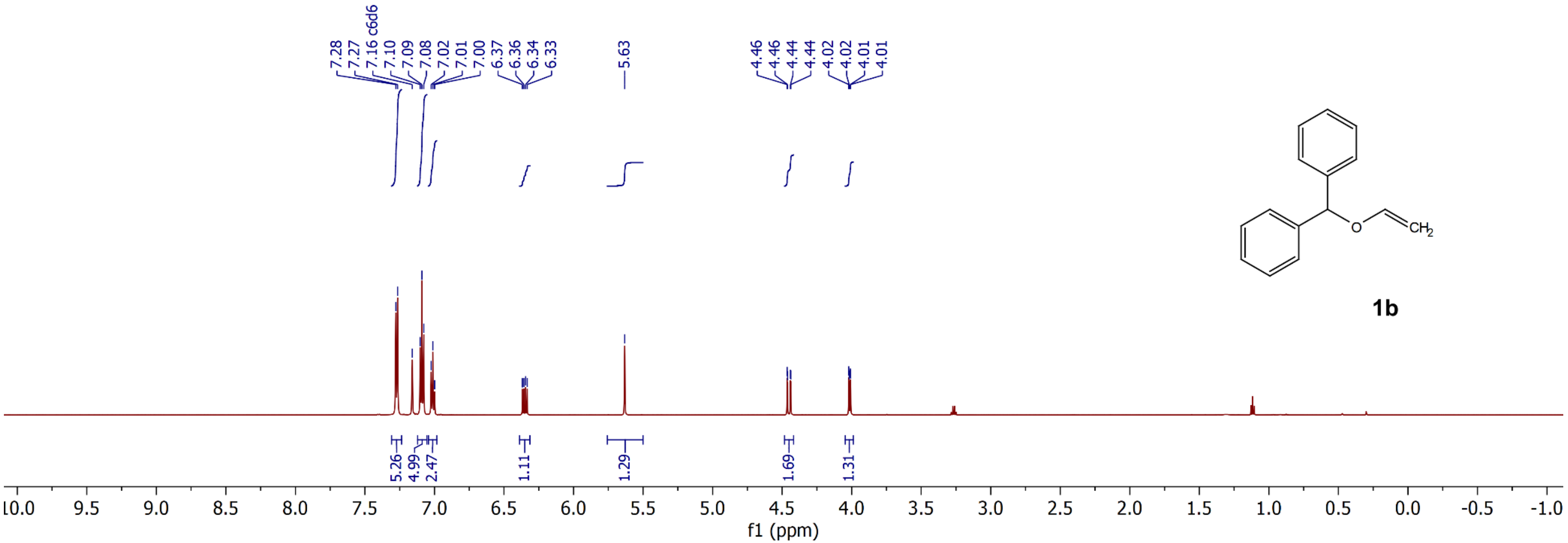

^{13}C{^{1}H}-NMR (151 MHz, C_6D_6) spectrum of compound **1b** (literature procedure[1])

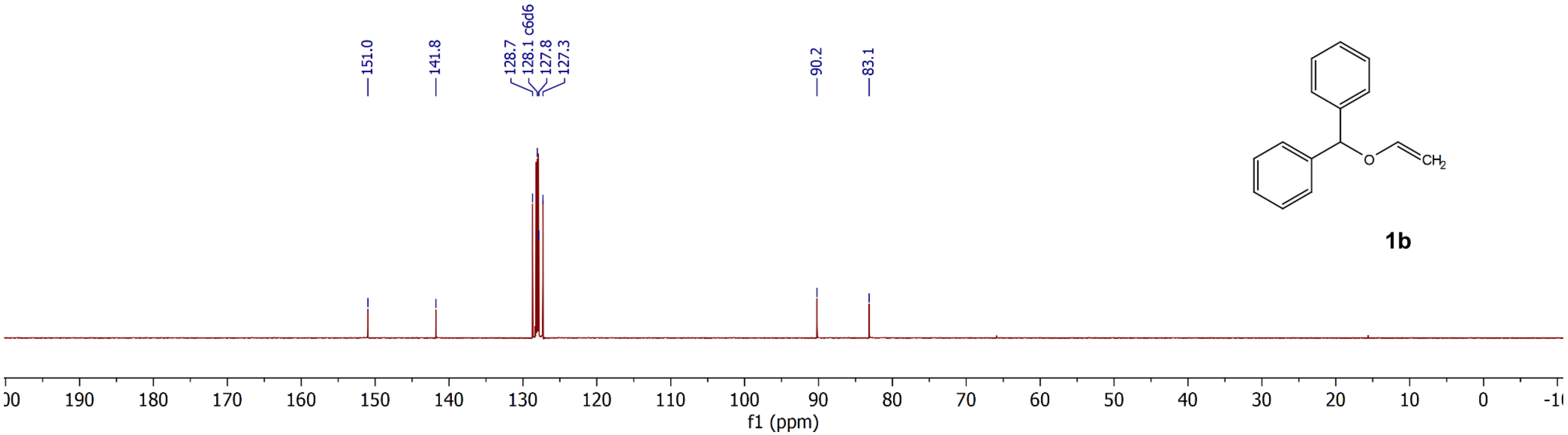

^{1}H-NMR (400 MHz, C_6D_6) spectrum of compound **1c** (literature procedure[1])

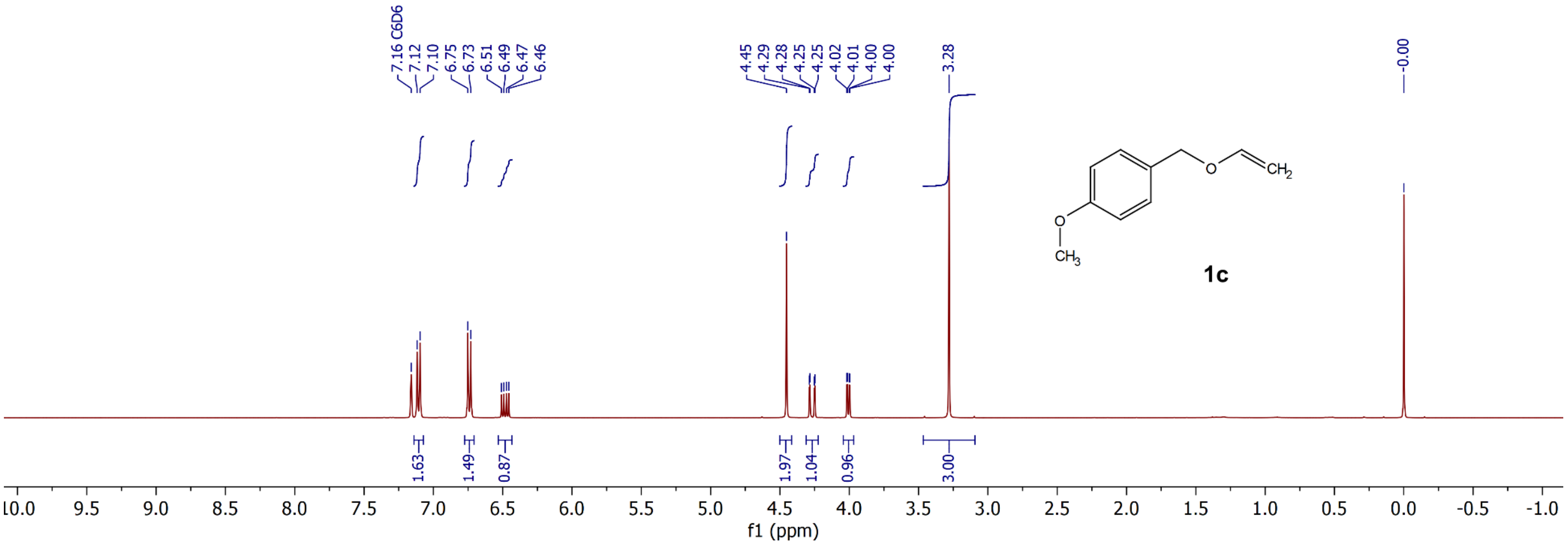

^{13}C{^{1}H}-NMR (101 MHz, C_6D_6) spectrum of compound **1c** (literature procedure[1])

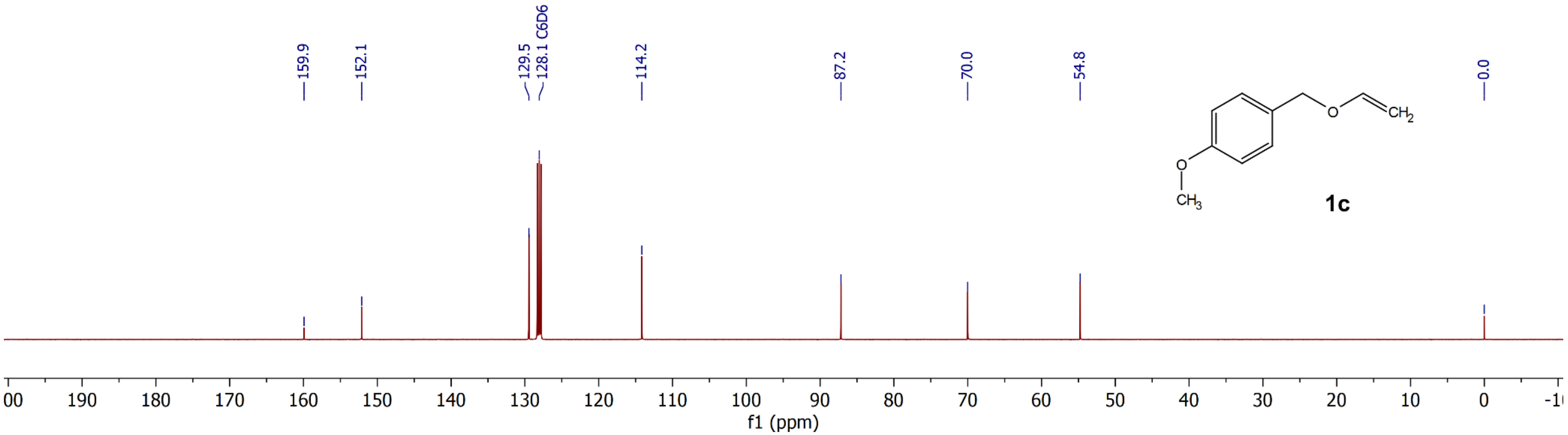

^{1}H-NMR (400 MHz, $CDCl_3$) spectrum of compound **1d** (literature procedure[1])

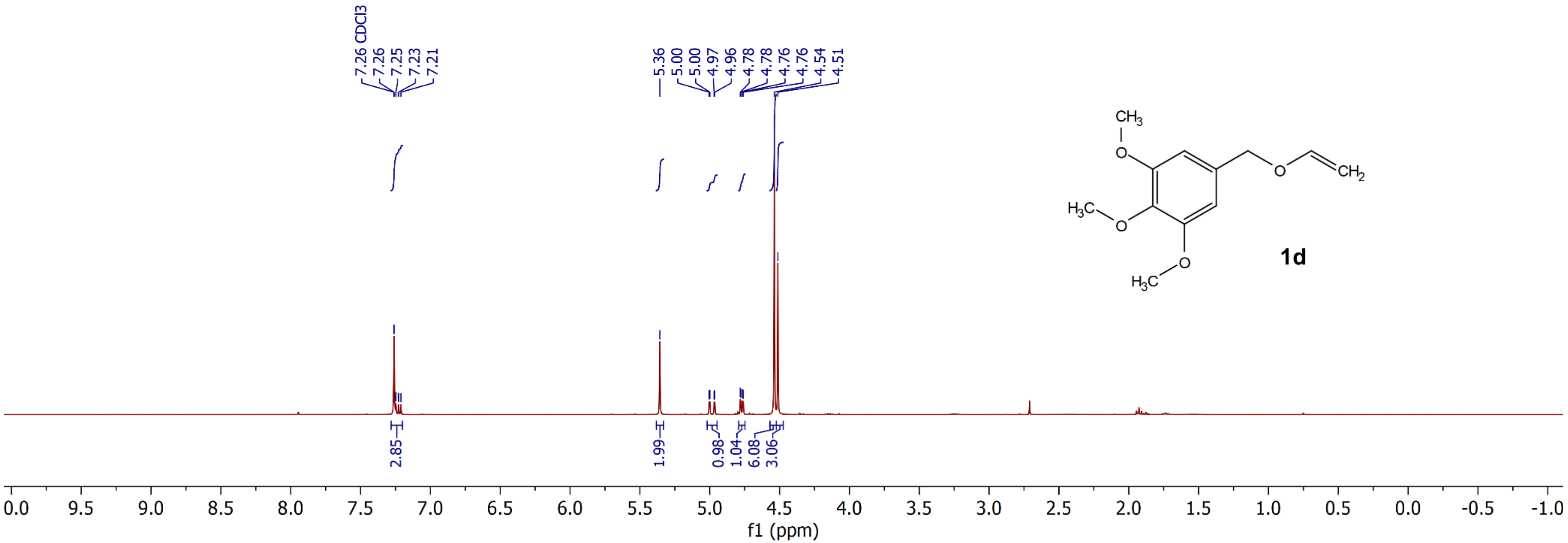

^{13}C{^{1}H}-NMR (101 MHz, $CDCl_3$) spectrum of compound **1d** (literature procedure[1])

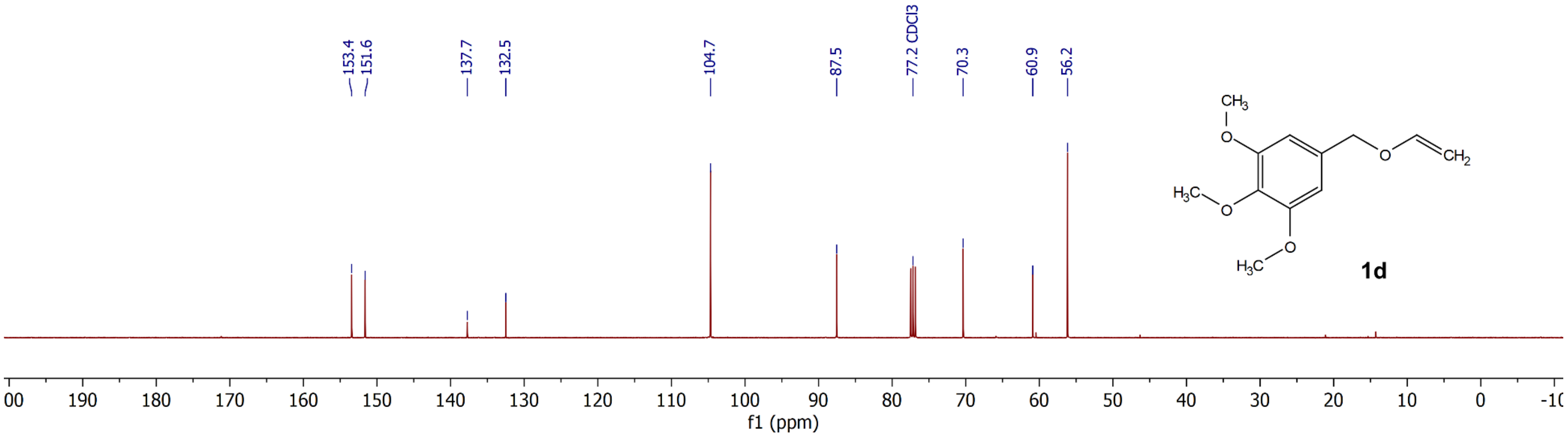

^{1}H-NMR (400 MHz, C_6D_6) spectrum of compound **1e** (literature procedure[1])

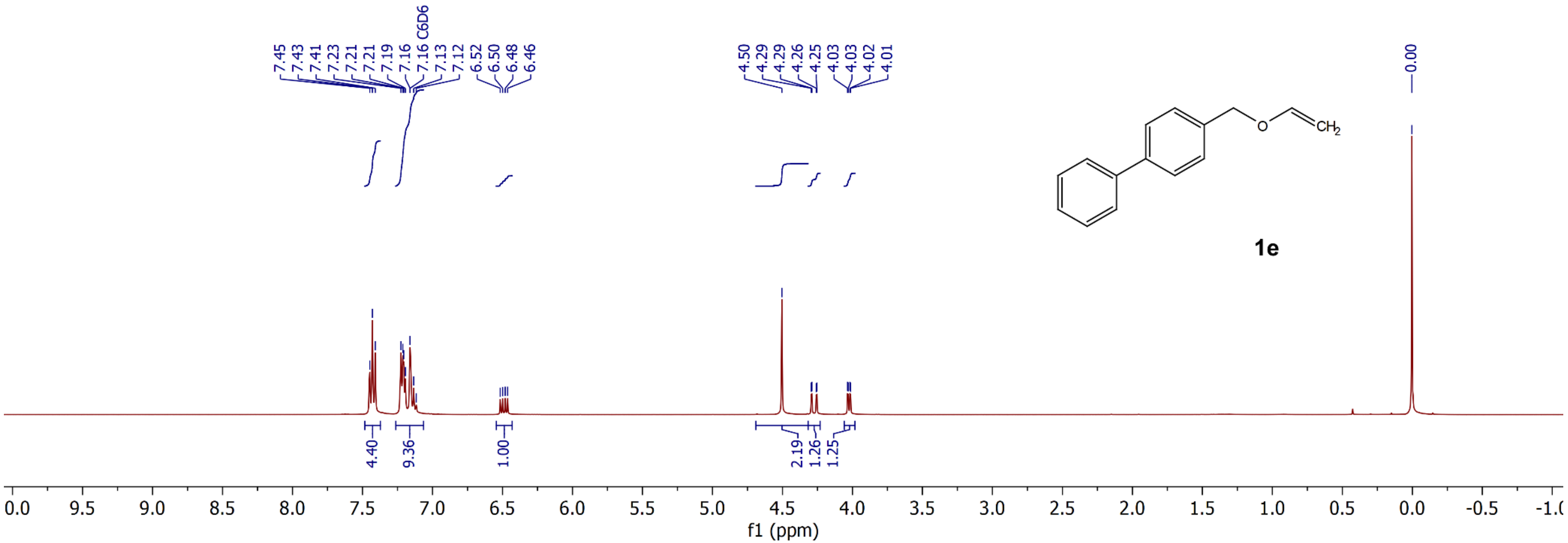

^{13}C{^{1}H}-NMR (101 MHz, C_6D_6) spectrum of compound **1e** (literature procedure[1])

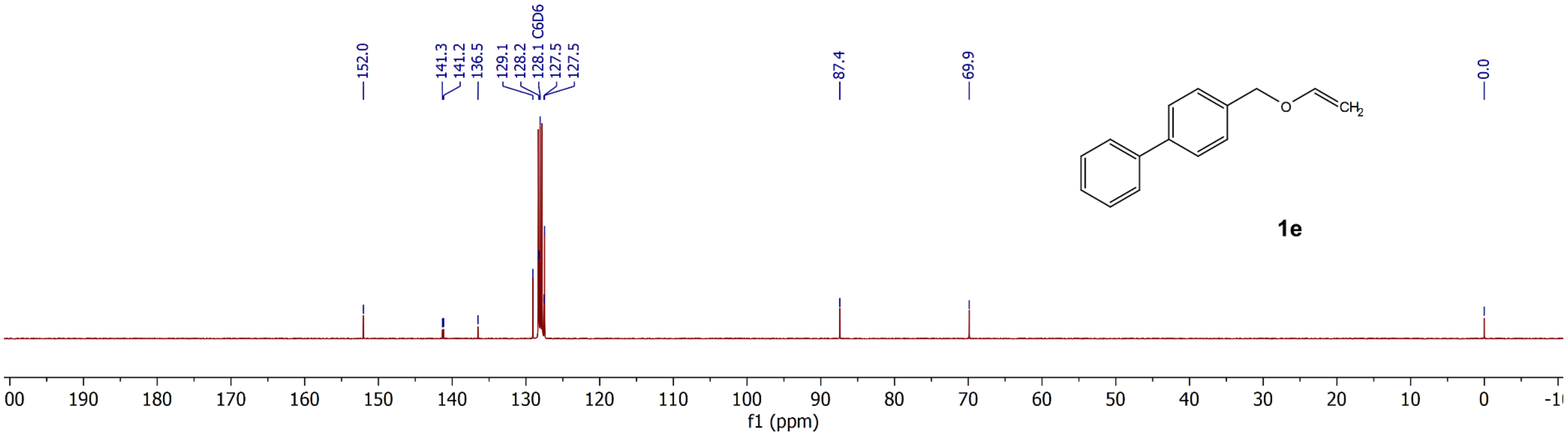

SUPPORTING INFORMATION

^{1}H-NMR (400 MHz, C_6D_6) spectrum of compound **1f** (literature procedure[1])

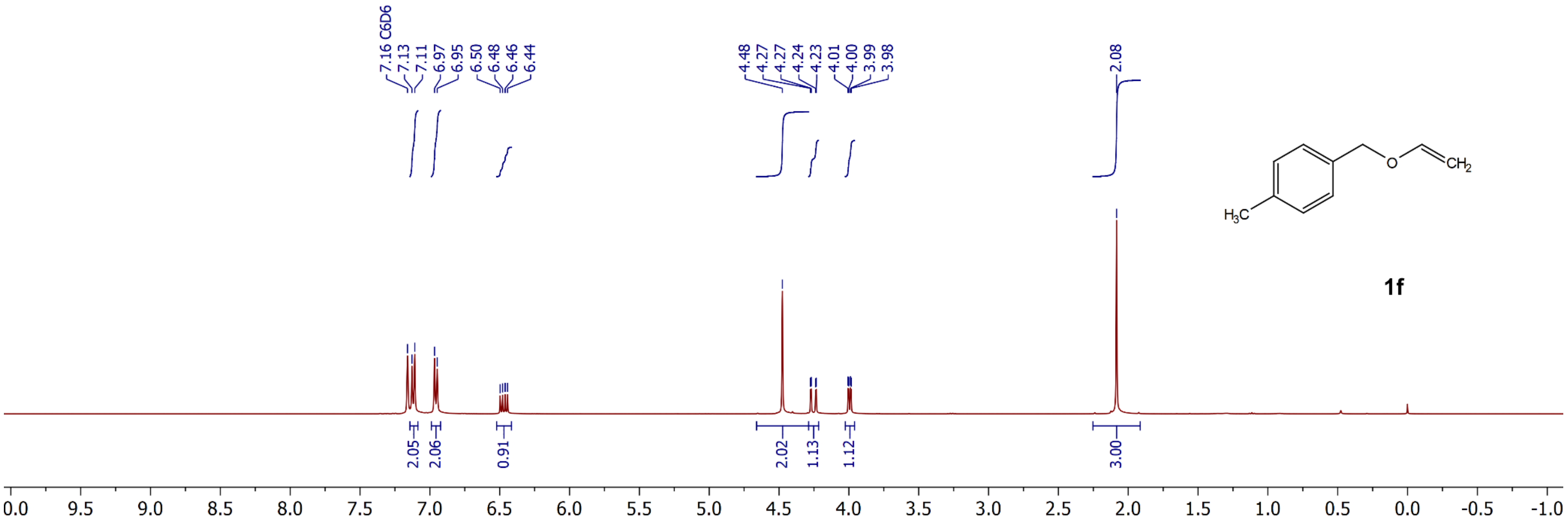

^{13}C{^{1}H}-NMR (101 MHz, C_6D_6) spectrum of compound **1f** (literature procedure[1])

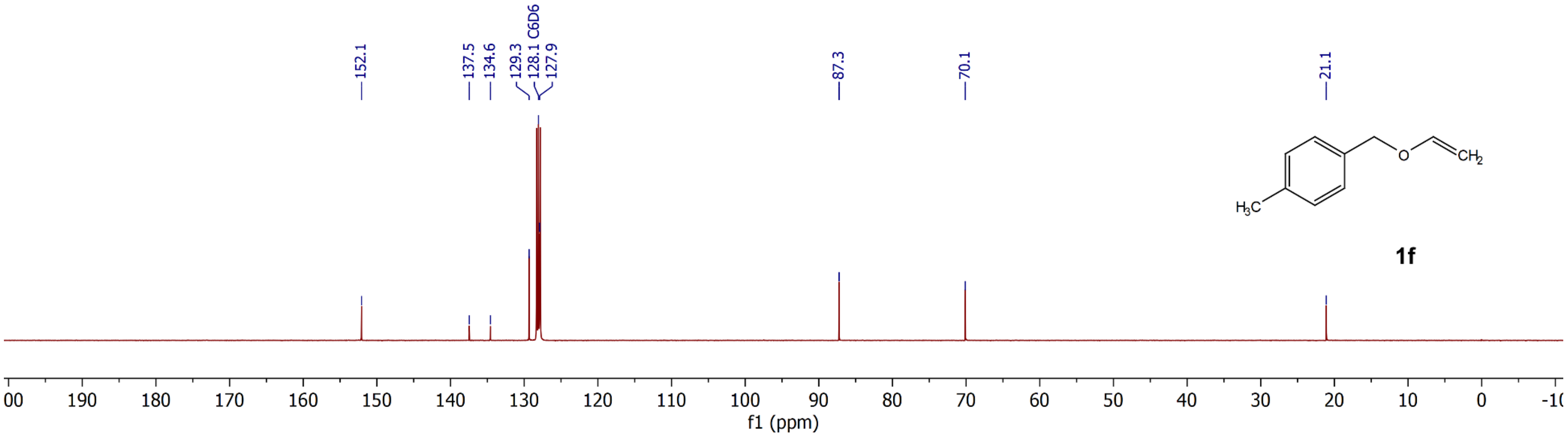

^{1}H-NMR (400 MHz, C_6D_6) spectrum of compound **1g** (literature procedure[1])

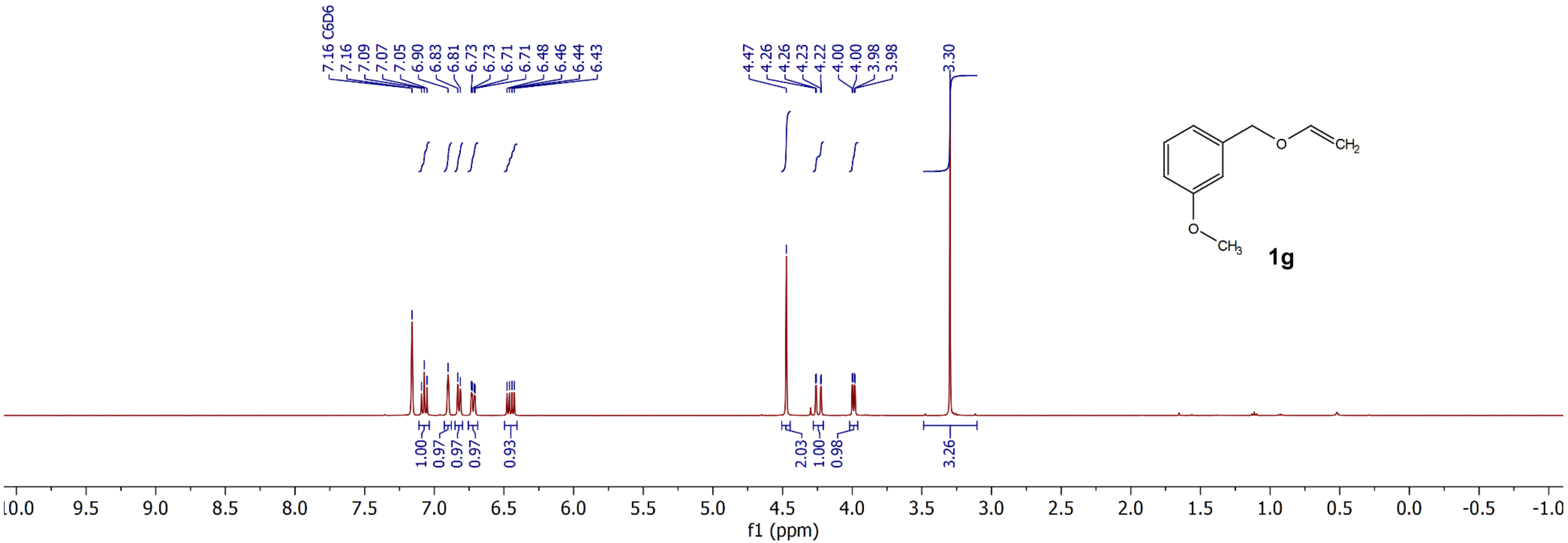

^{13}C{^{1}H}-NMR (101 MHz, C_6D_6) spectrum of compound **1g** (literature procedure[1])

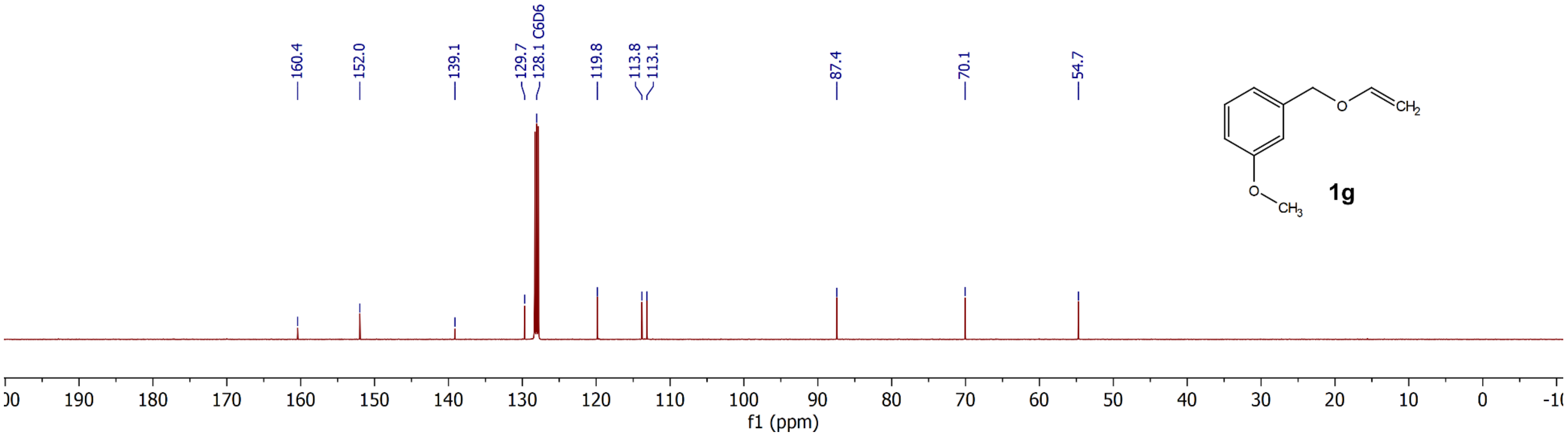

^{1}H-NMR (600 MHz, $CDCl_3$) spectrum of compound **1h** (literature procedure[1])

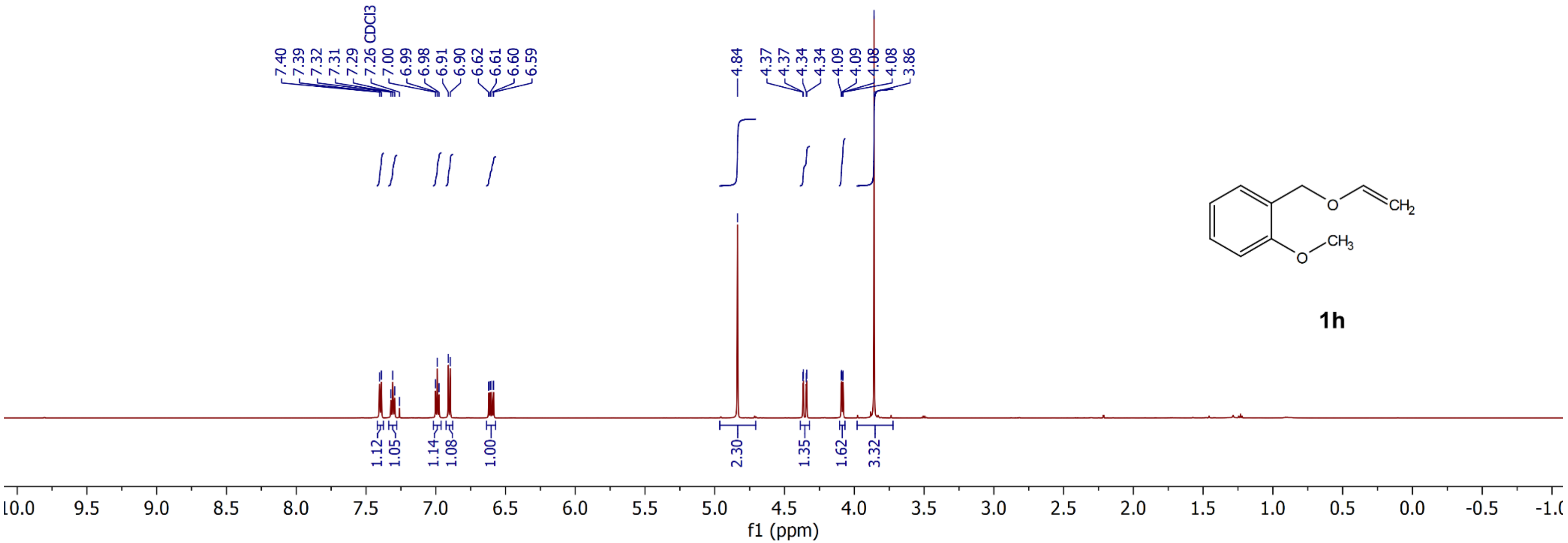

^{13}C{^{1}H}-NMR (151 MHz, $CDCl_3$) spectrum of compound **1h** (literature procedure[1])

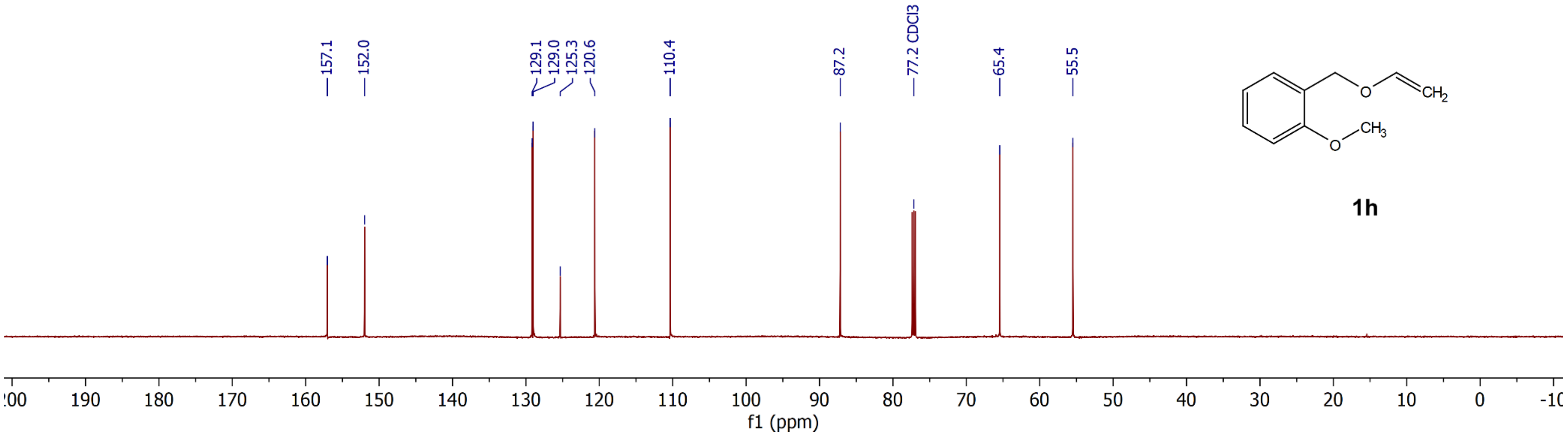

^{1}H-NMR (400 MHz, $CDCl_3$) spectrum of compound **1i** (literature procedure[1])

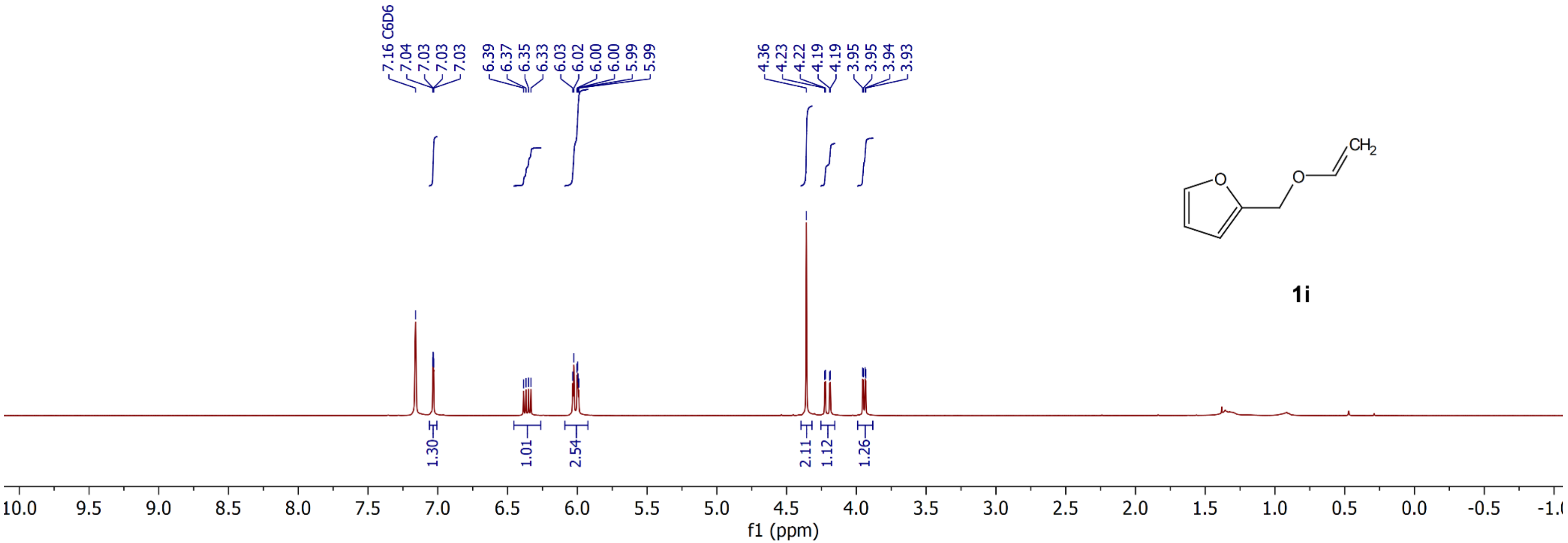

^{13}C{^{1}H}-NMR (101 MHz, $CDCl_3$) spectrum of compound **1i** (literature procedure[1])

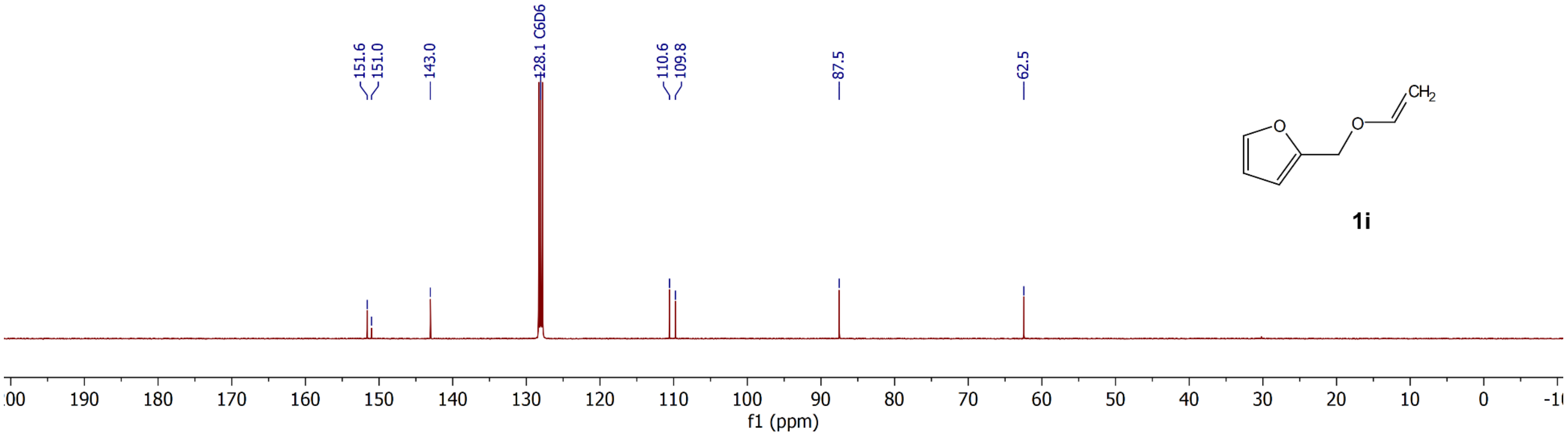

^{1}H-NMR (600 MHz, $CDCl_3$) spectrum of compound **1j** (literature procedure[1])

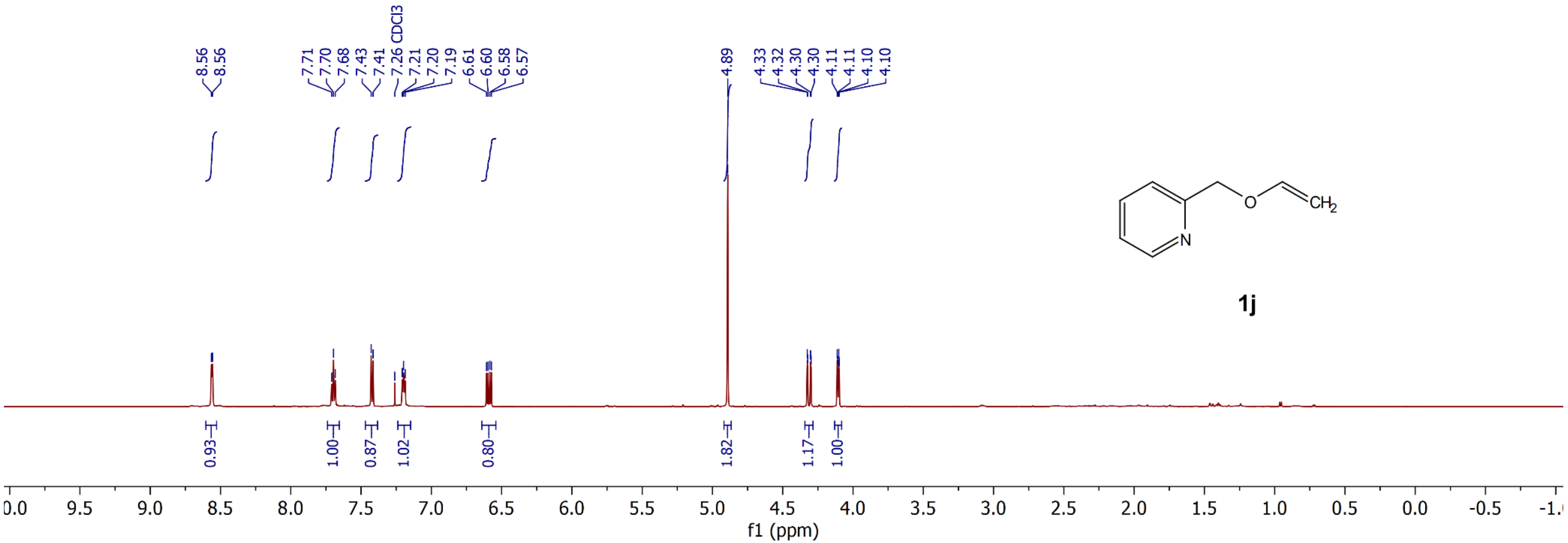

^{13}C{^{1}H}-NMR (151 MHz, $CDCl_3$) spectrum of compound **1j** (literature procedure[1])

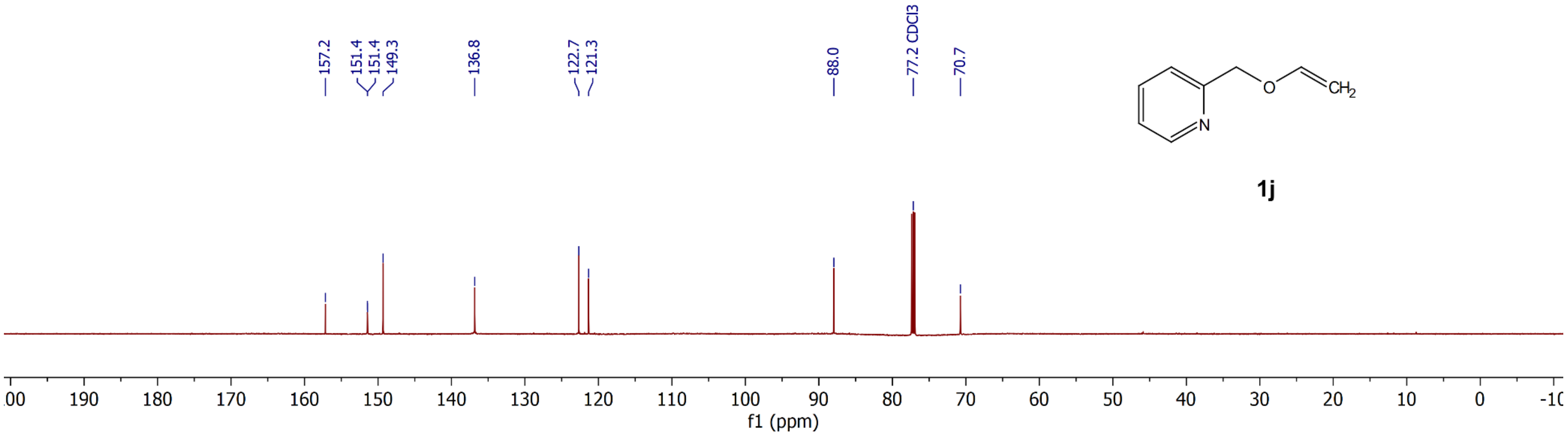

^{1}H-NMR (600 MHz, $CDCl_3$) spectrum of compound **8** (literature procedure[1])

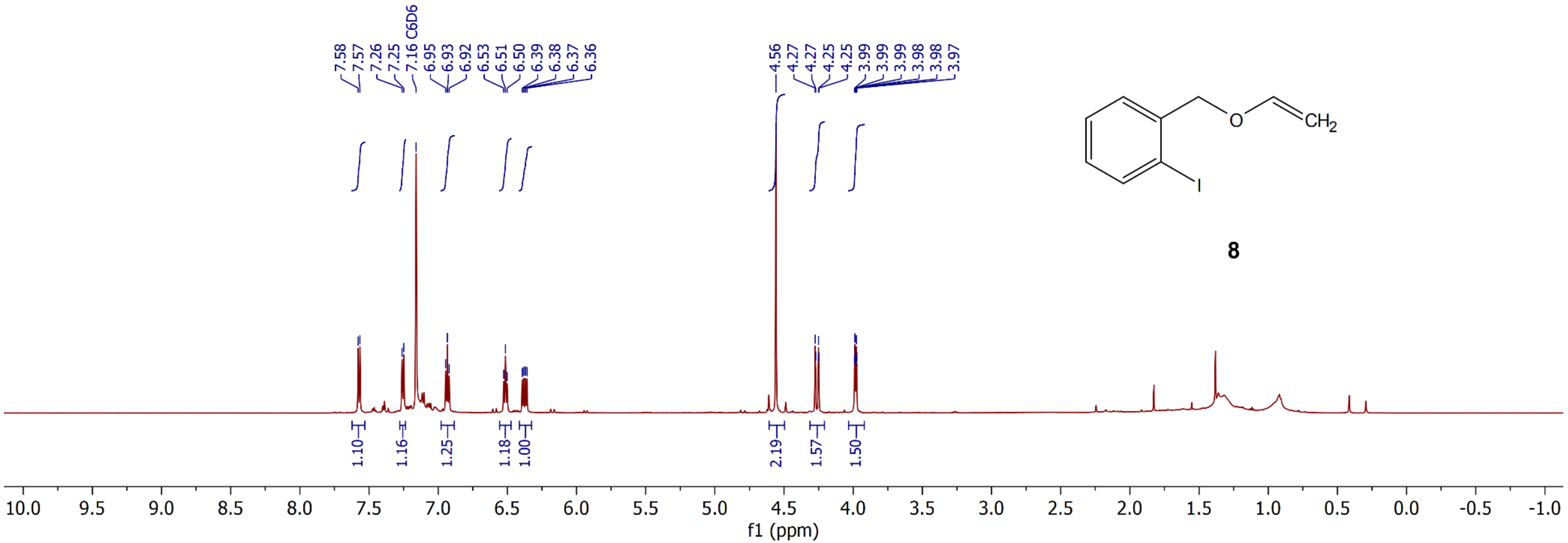

^{13}C{^{1}H}-NMR (151 MHz, $CDCl_3$) spectrum of compound **8** (literature procedure[1])

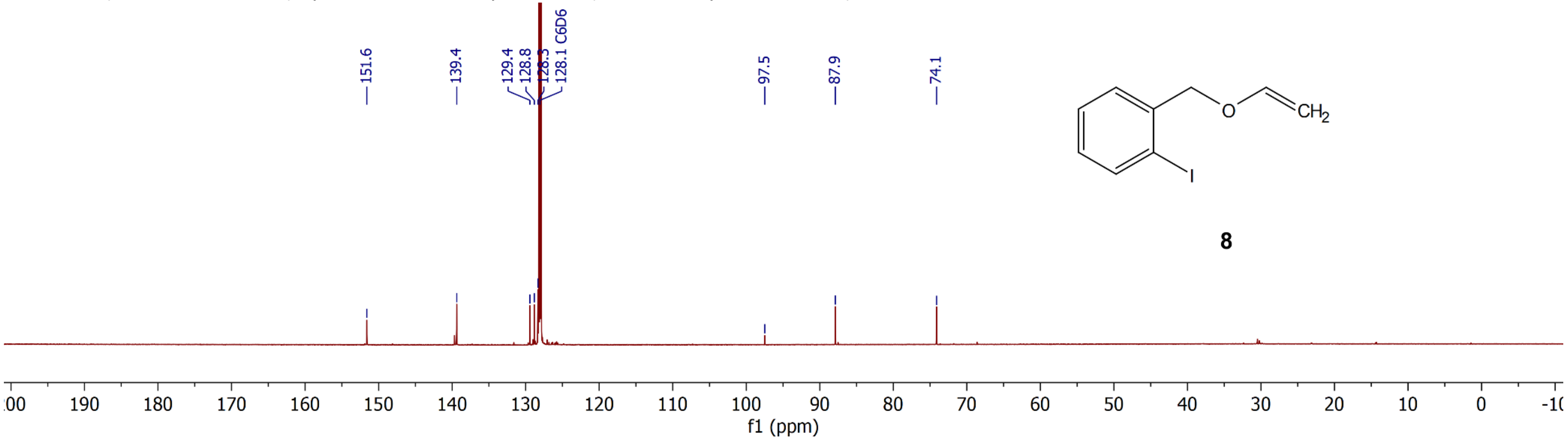

^{1}H-NMR (600 MHz, C_6D_6) spectrum of compound **6a** (procedure GP1)

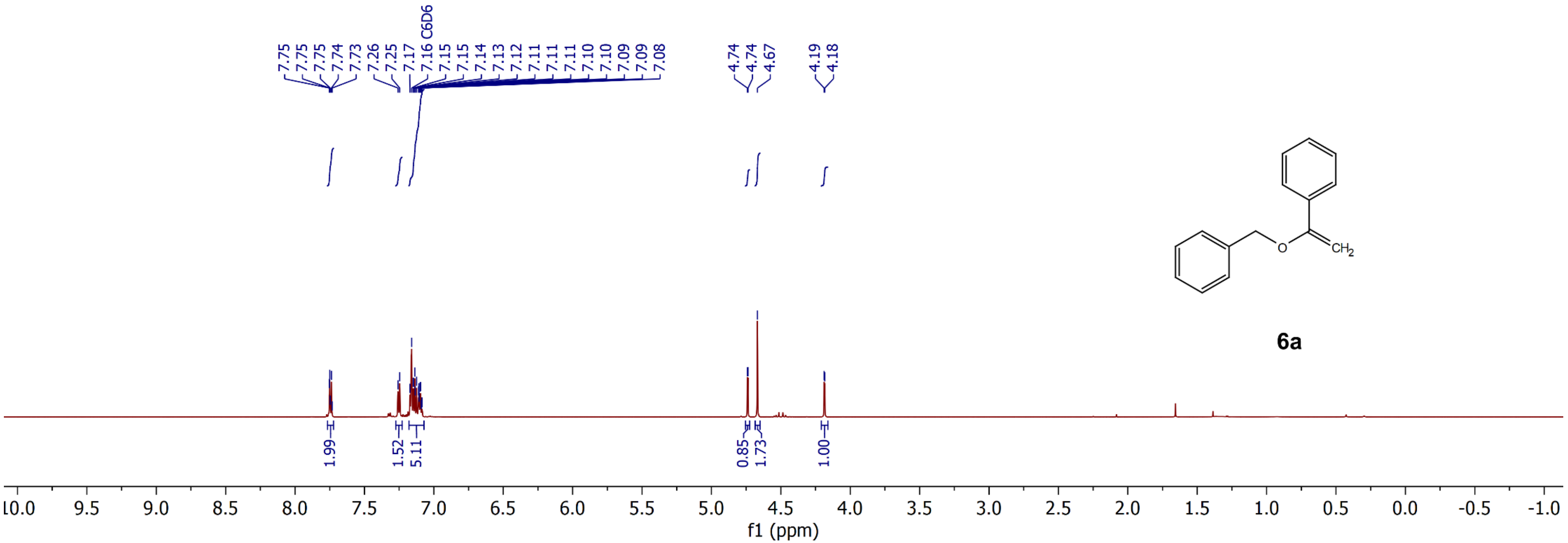

^{13}C{^{1}H}-NMR (151 MHz, C_6D_6) spectrum of compound **6a** (procedure GP1)

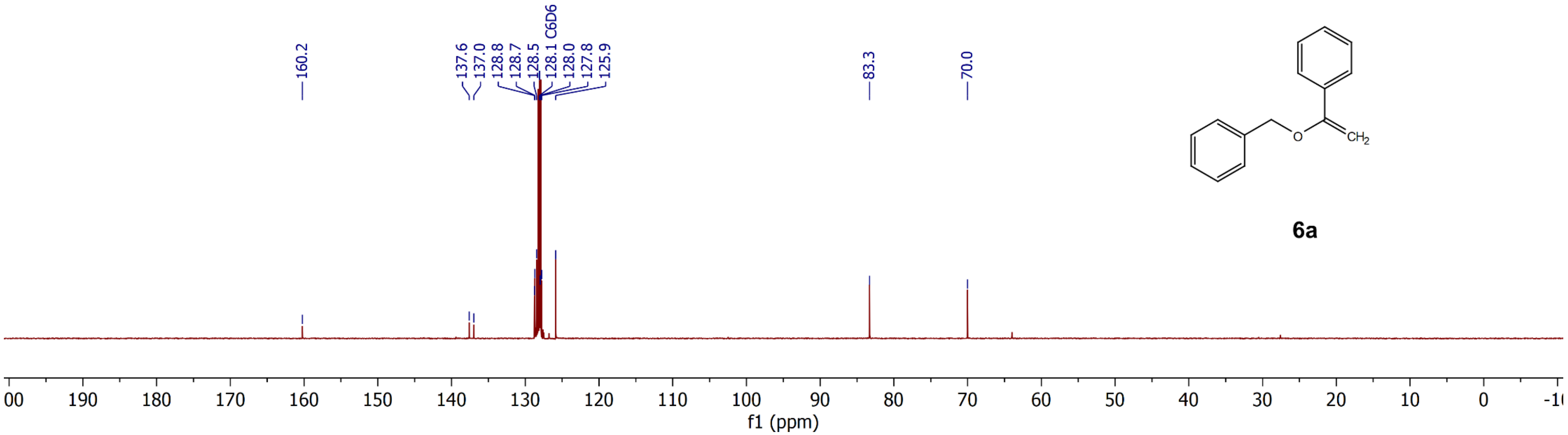

^{1}H-NMR (600 MHz, C_6D_6) spectrum of compound **6b** (procedure GP1)

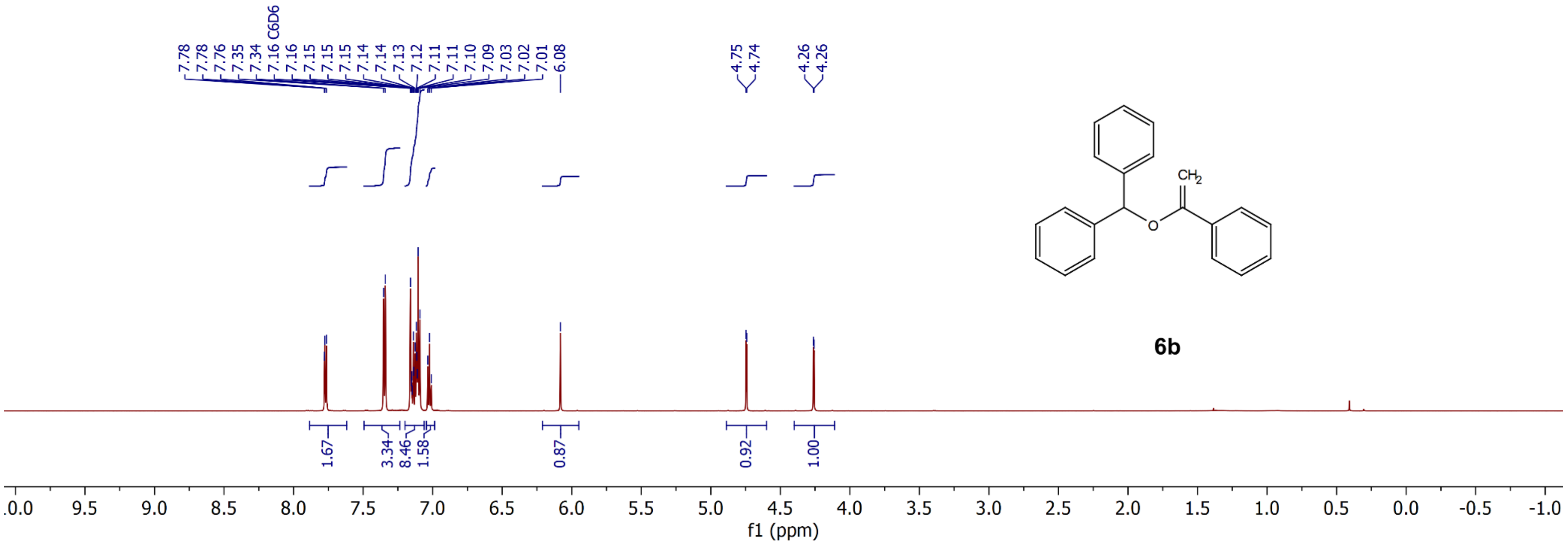

^{13}C{^{1}H}-NMR (151 MHz, C_6D_6) spectrum of compound **6b** (procedure GP1)

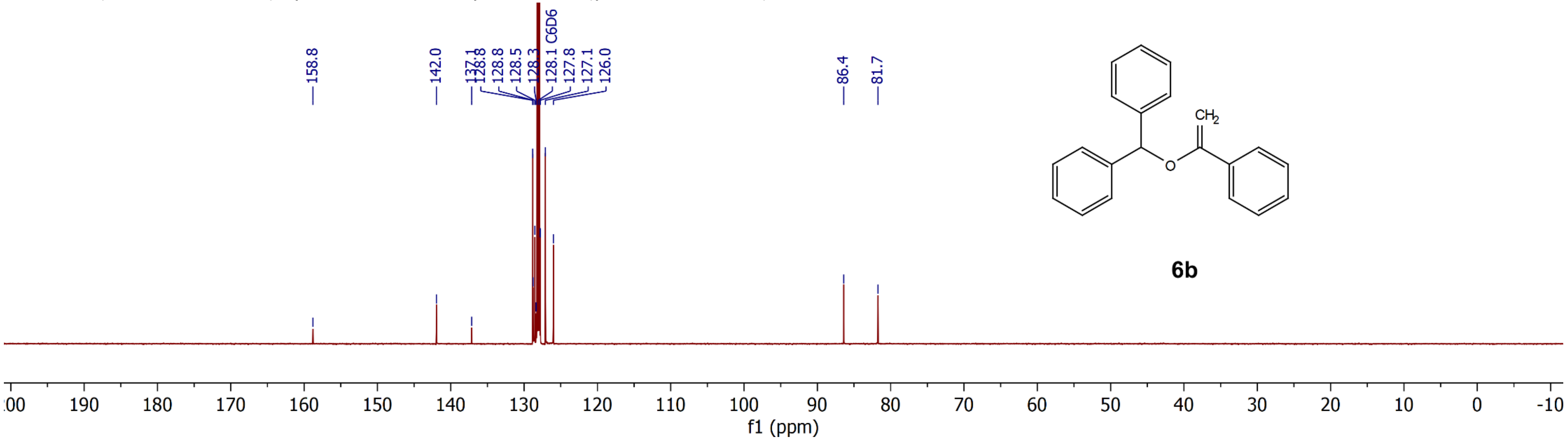

^{1}H-NMR (400 MHz, C_6D_6) spectrum of compound **6c** (procedure GP1)

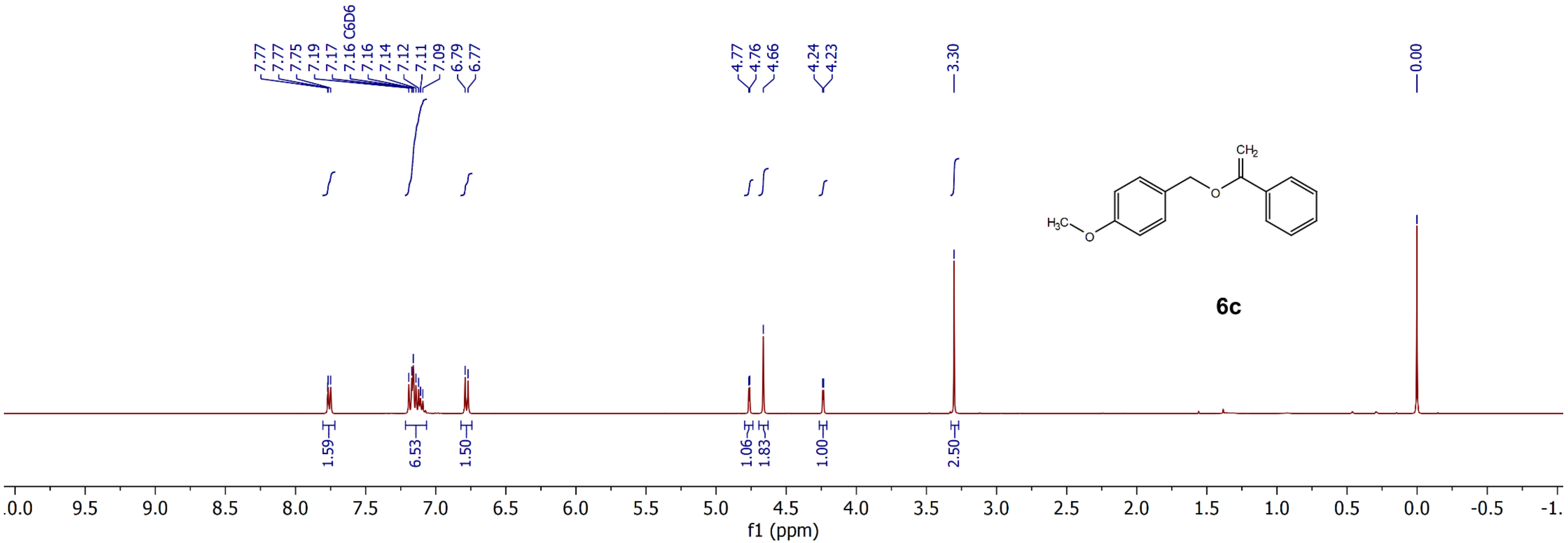

^{13}C{^{1}H}-NMR (101 MHz, C_6D_6) spectrum of compound **6c** (procedure GP1)

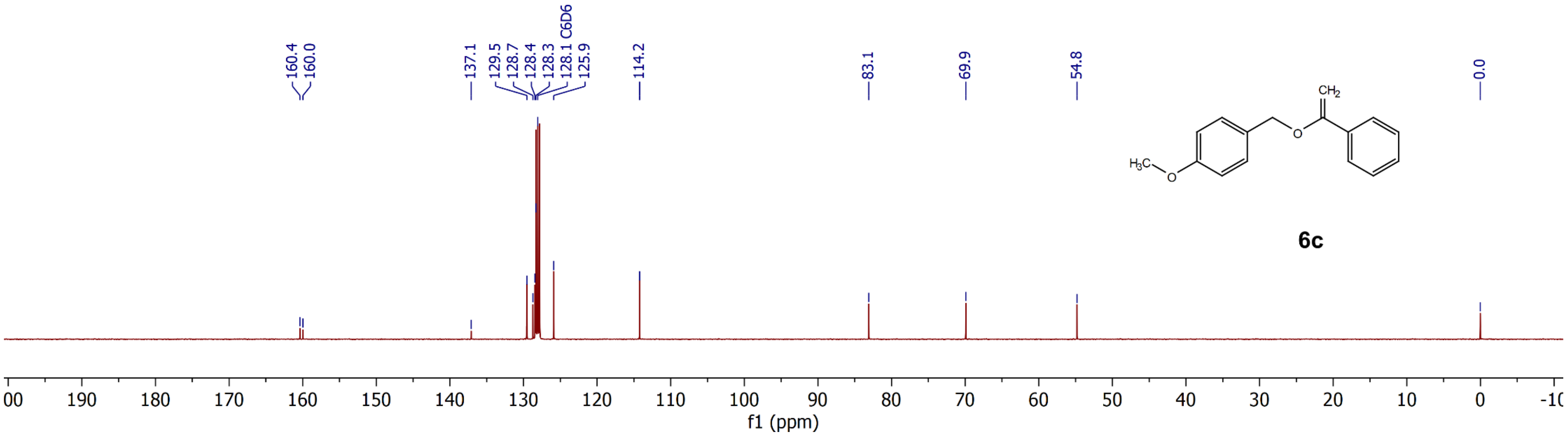

^{1}H-NMR (600 MHz, C_6D_6) spectrum of compound **6d** (procedure GP1)

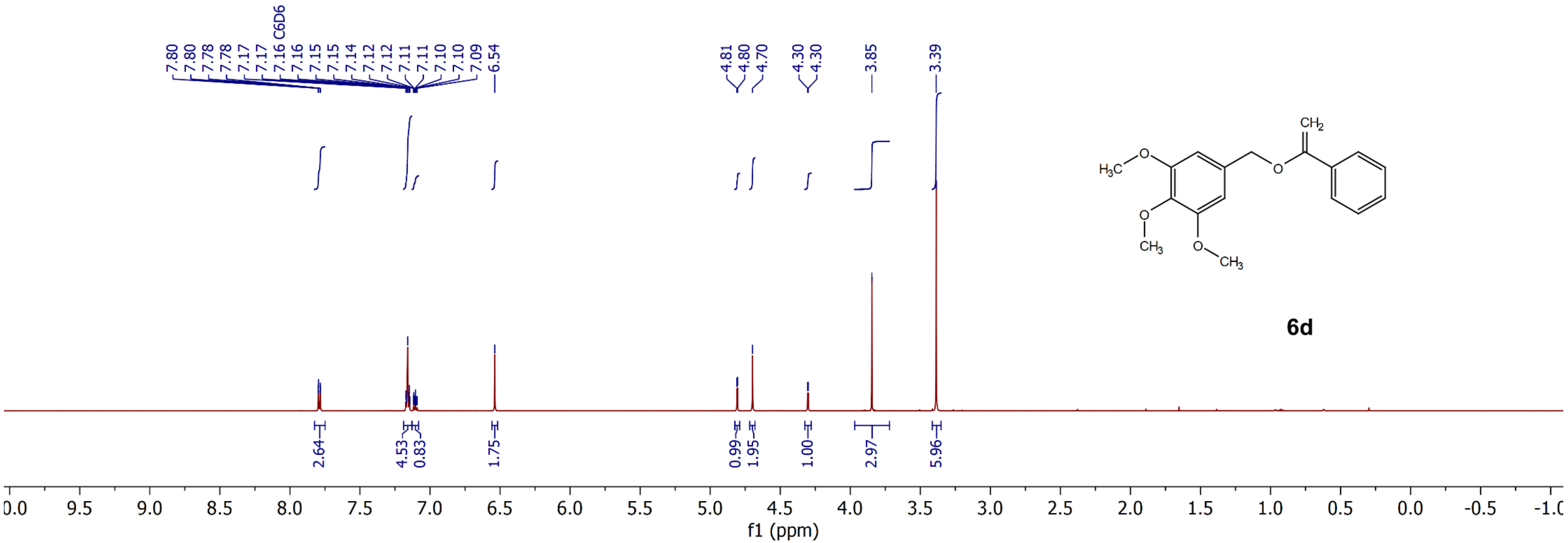

^{13}C{^{1}H}-NMR (151 MHz, C_6D_6) spectrum of compound **6d** (procedure GP1)

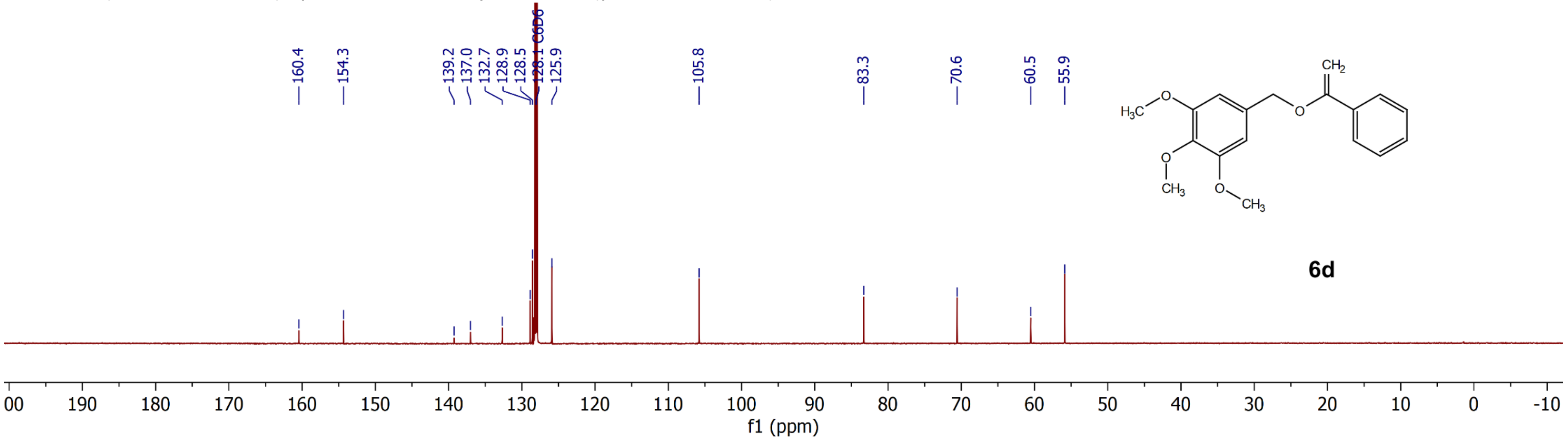

^{1}H-NMR (400 MHz, C_6D_6) spectrum of compound **6e** (procedure GP1)

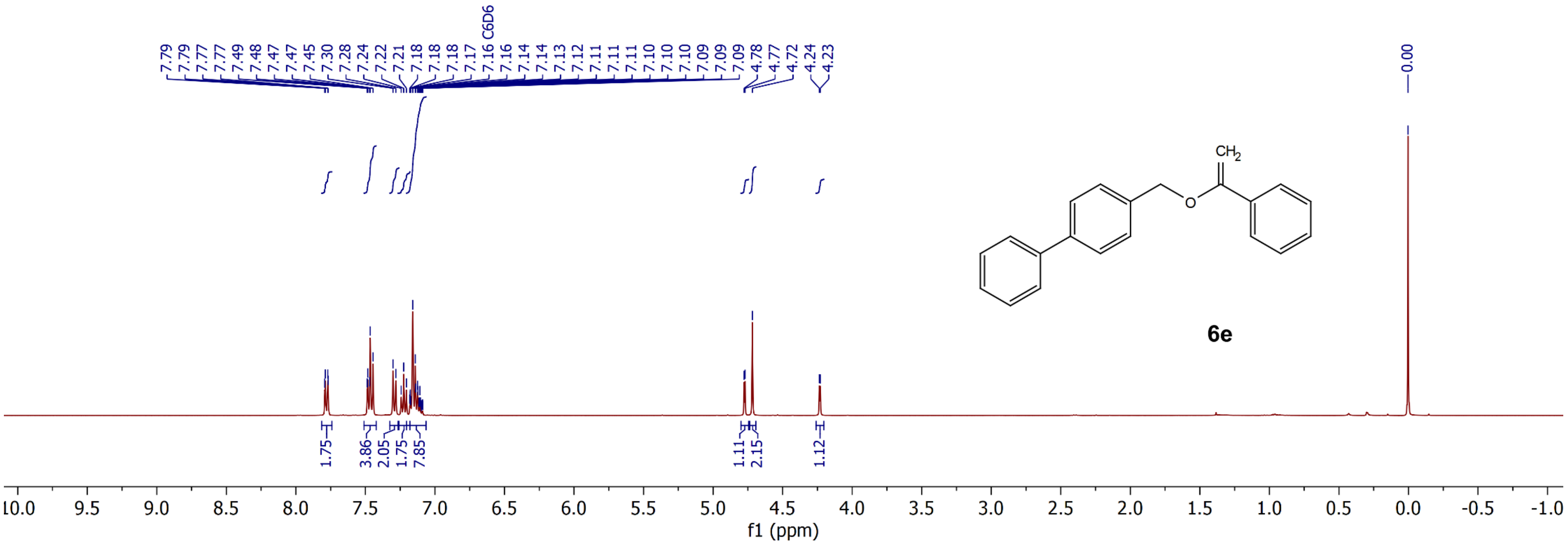

$^{13}C\{^{1}H\}$-NMR (101 MHz, C_6D_6) spectrum of compound **6e** (procedure GP1)

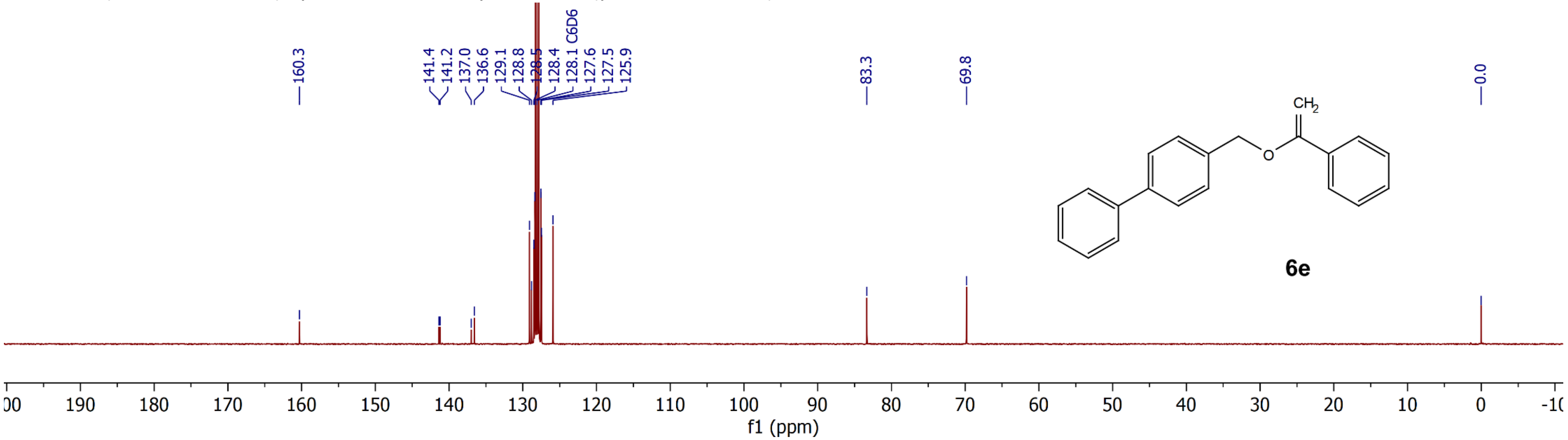

^{1}H-NMR (400 MHz, C_6D_6) spectrum of compound **6f** (procedure GP1)

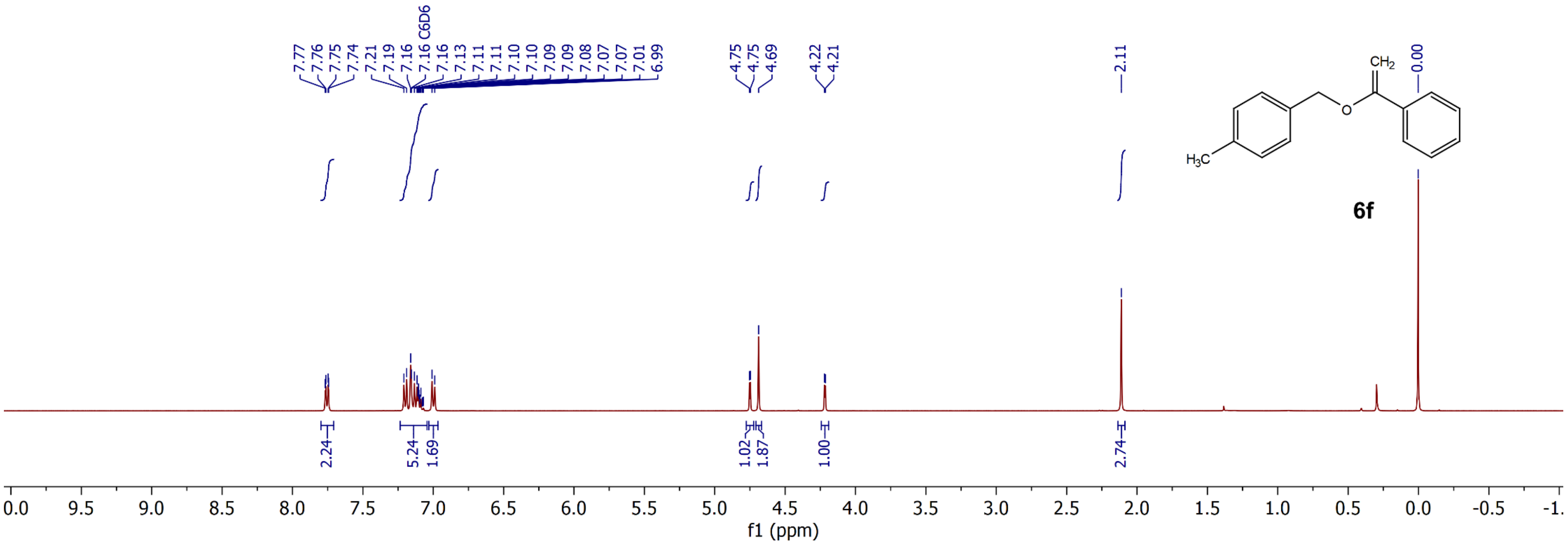

$^{13}C\{^{1}H\}$-NMR (101 MHz, C_6D_6) spectrum of compound **6f** (procedure GP1)

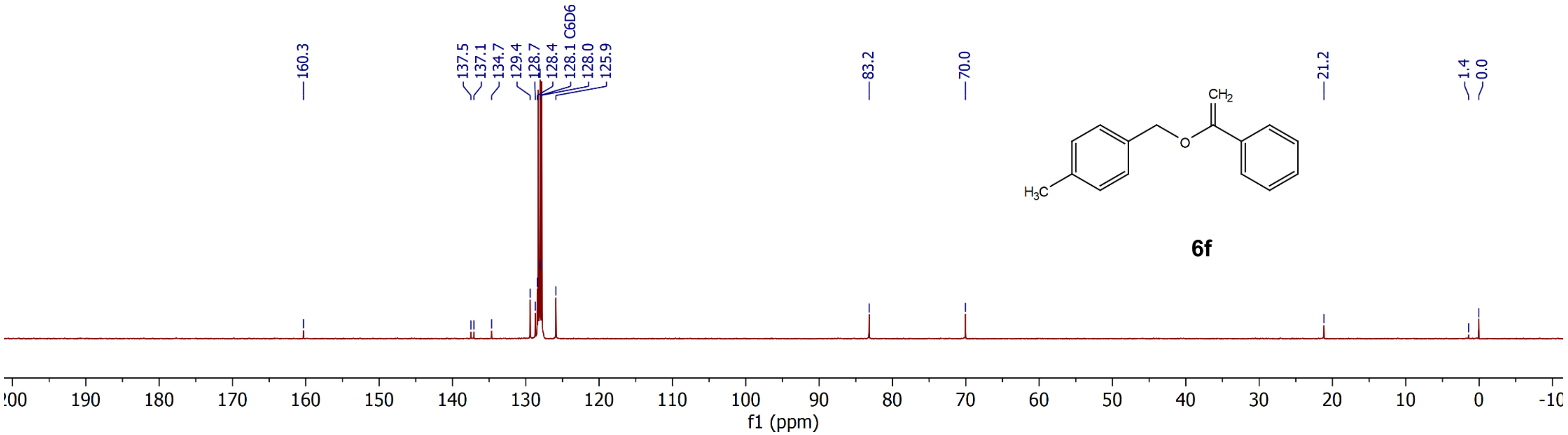

^{1}H-NMR (400 MHz, C_6D_6) spectrum of compound **6g** (procedure GP1)

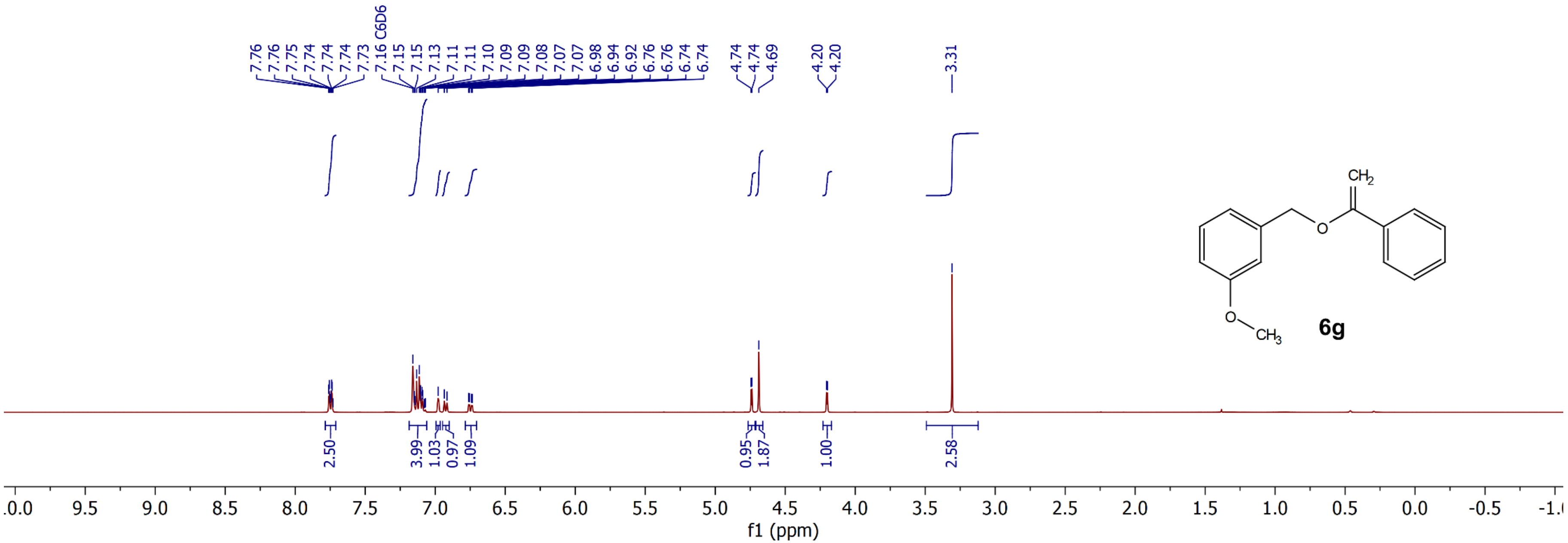

^{13}C{^{1}H}-NMR (101 MHz, C_6D_6) spectrum of compound **6g** (procedure GP1)

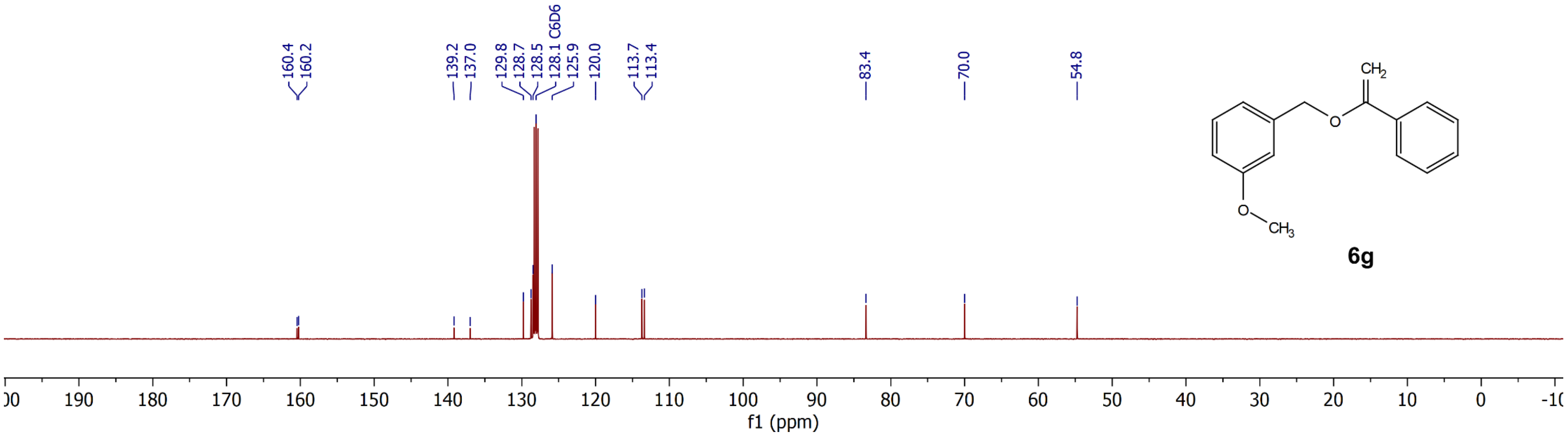

^{1}H-NMR (400 MHz, C_6D_6) spectrum of compound **6h** (procedure GP1)

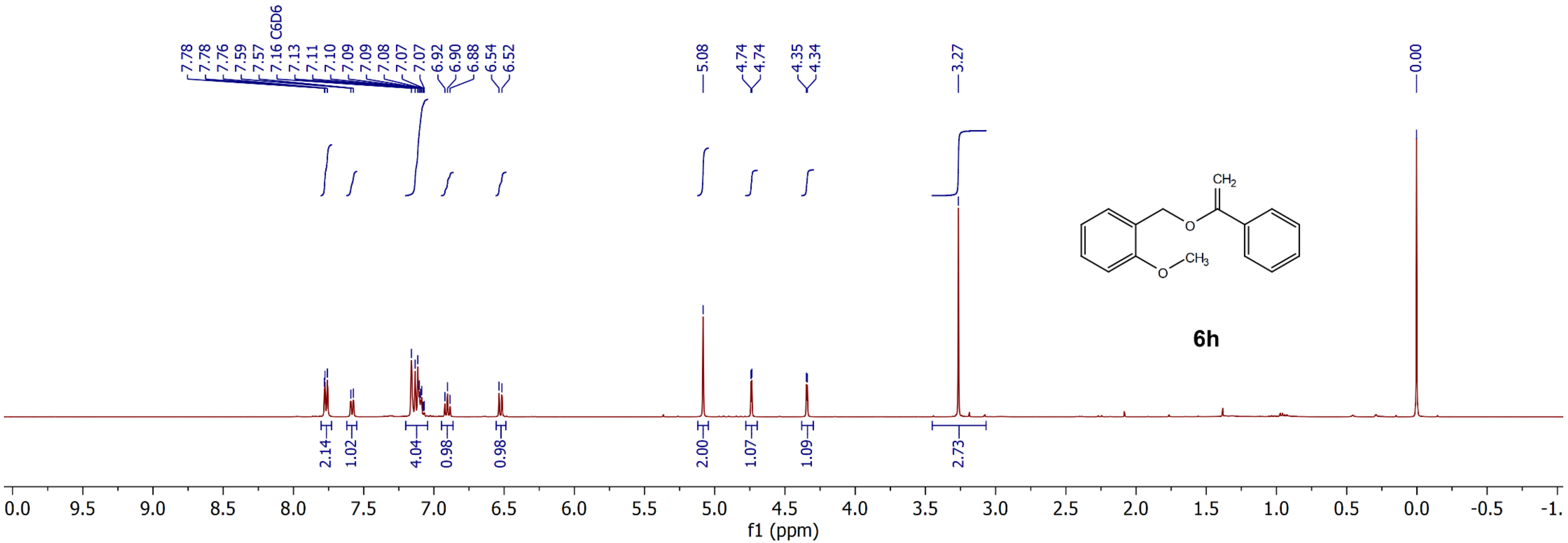

$^{13}C\{^{1}H\}$-NMR (101 MHz, C_6D_6) spectrum of compound **6h** (procedure GP1)

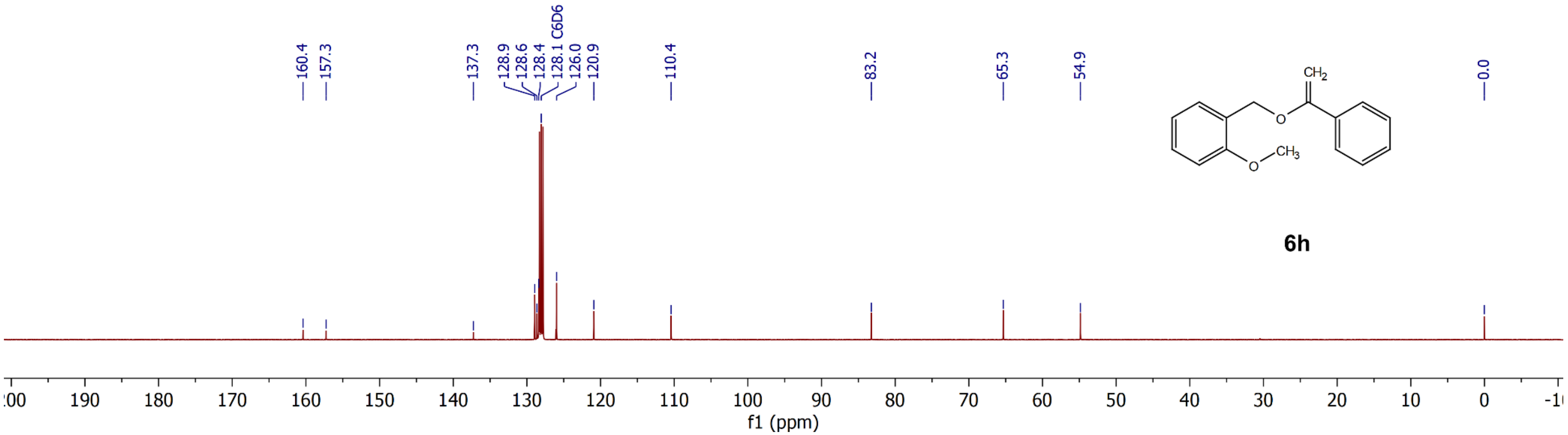

^{1}H-NMR (600 MHz, C_6D_6) spectrum of compound **6i** (procedure GP1)

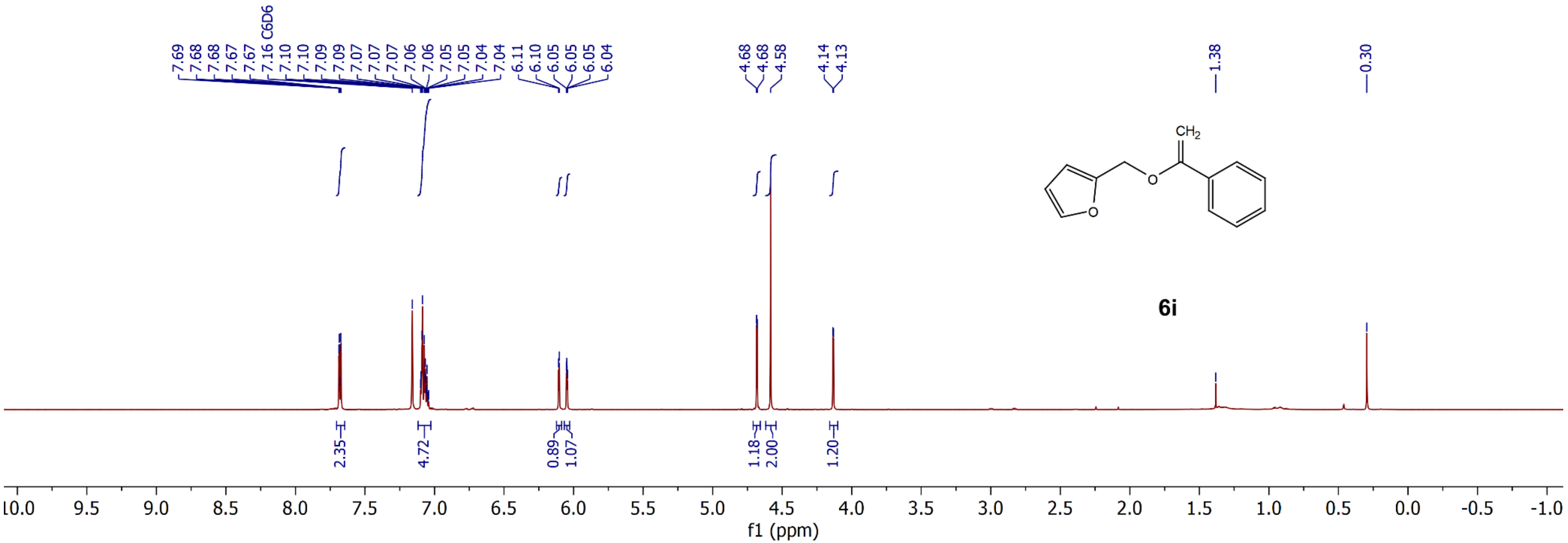

^{13}C{^{1}H}-NMR (151 MHz, C_6D_6) spectrum of compound **6i** (procedure GP1)

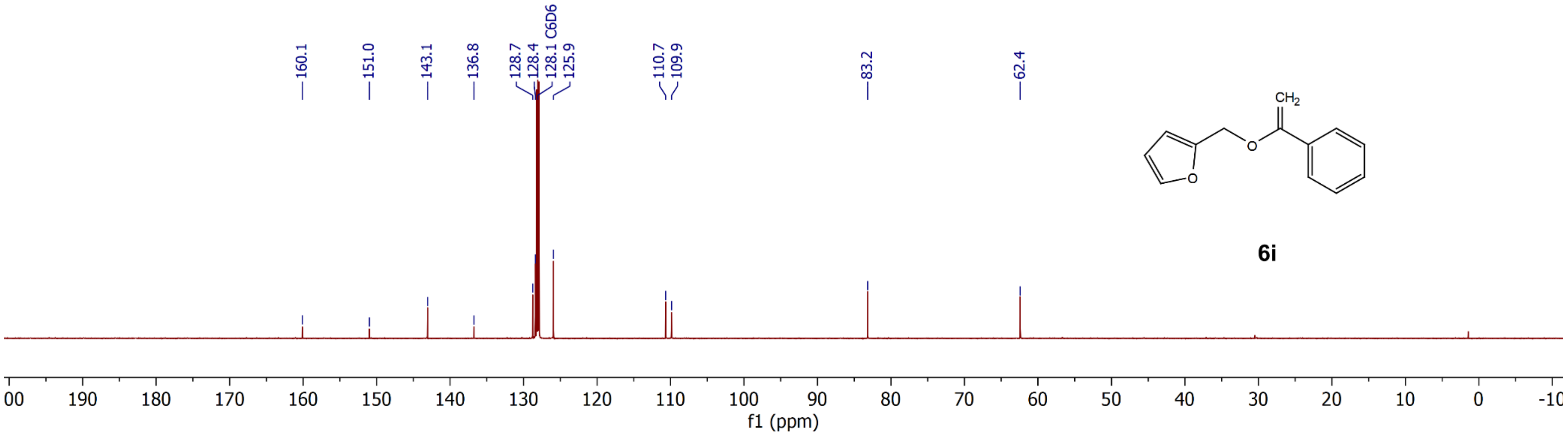

^1H-NMR (600 MHz, C_6D_6) spectrum of compound **6j** (procedure GP1)

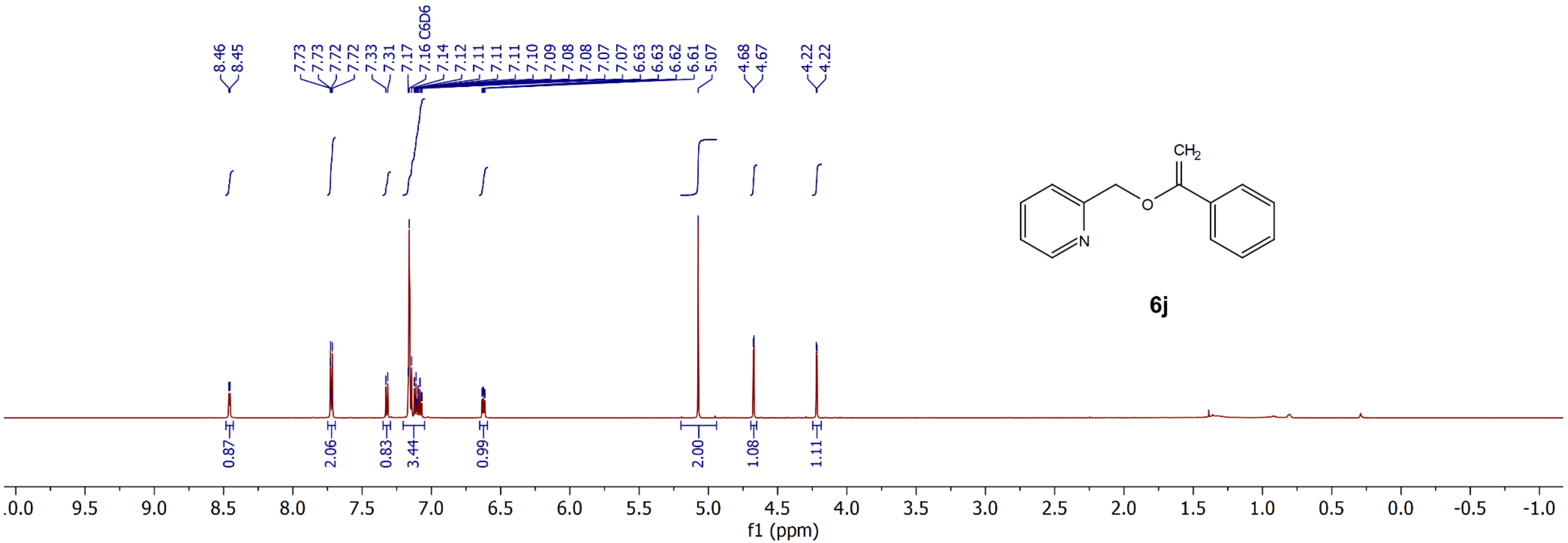

$^{13}C\{^1H\}$-NMR (151 MHz, C_6D_6) spectrum of compound **6j** (procedure GP1)

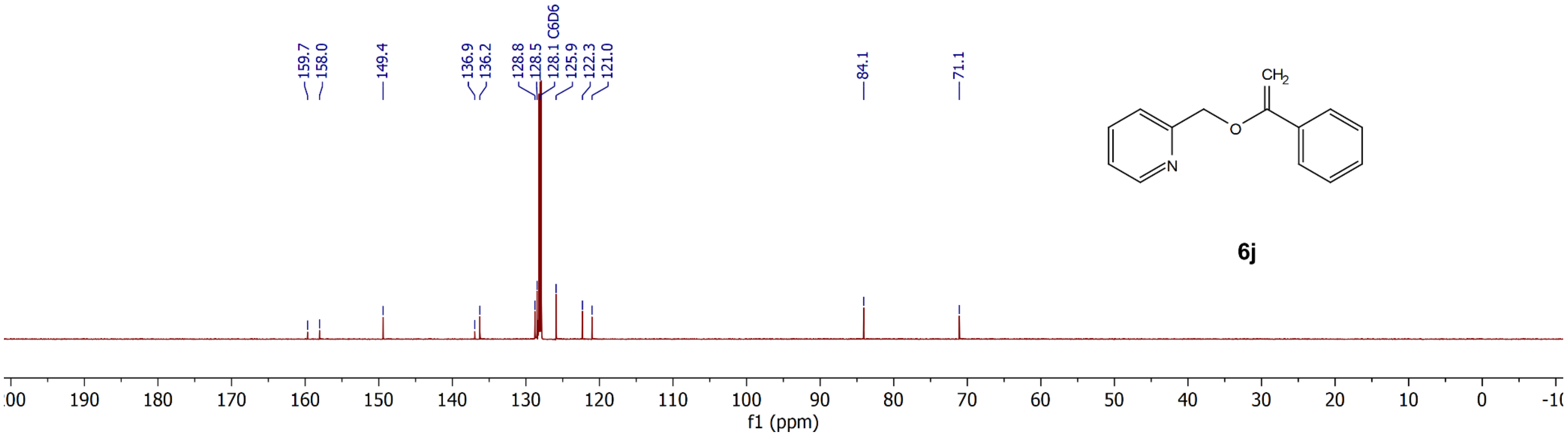

^{1}H-NMR (400 MHz, C_6D_6) spectrum of compound **6k** (procedure GP1)

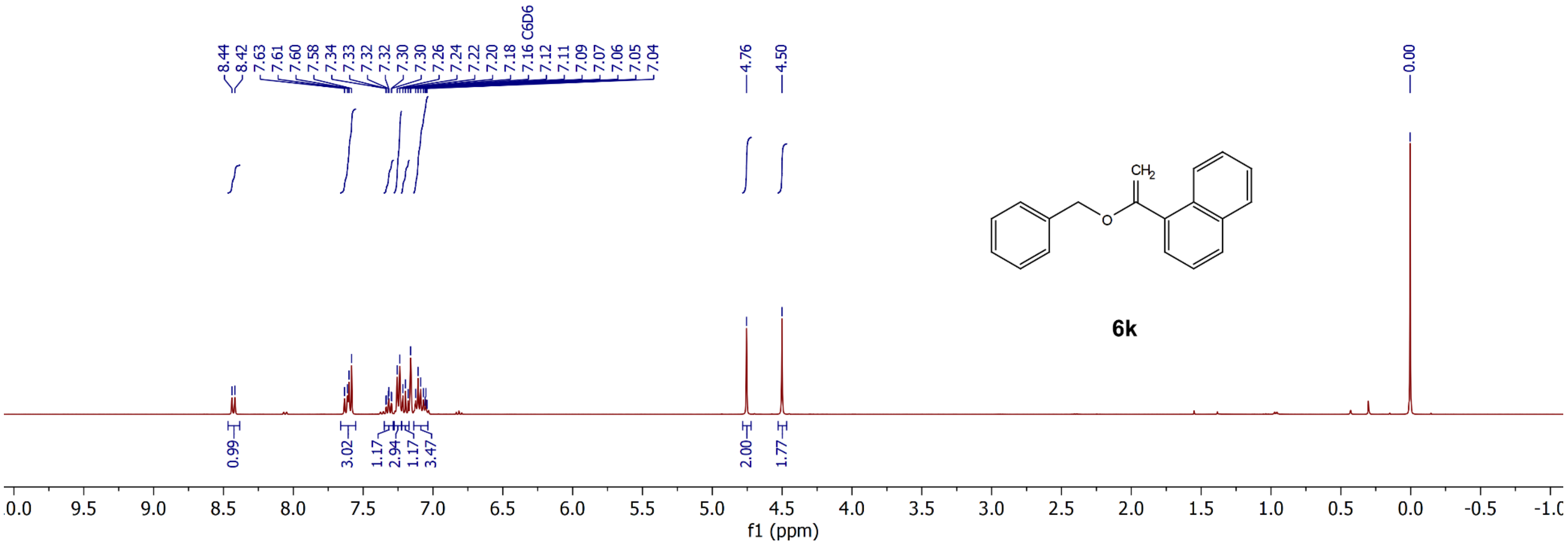

^{13}C{^{1}H}-NMR (101 MHz, C_6D_6) spectrum of compound **6k** (procedure GP1)

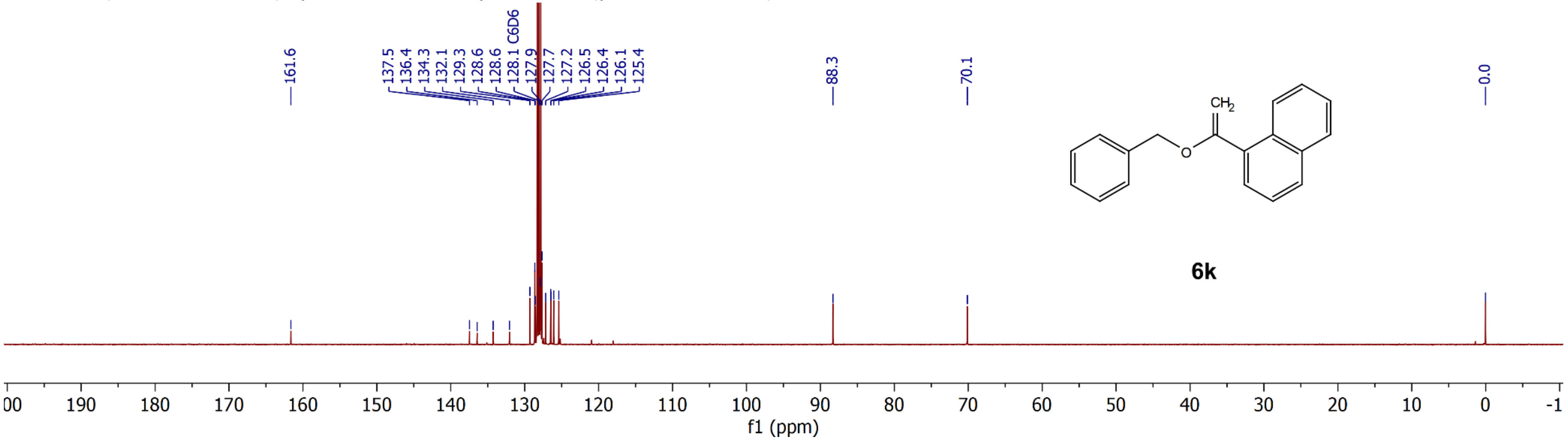

^{1}H-NMR (400 MHz, C_6D_6) spectrum of compound **6l** (procedure GP1)

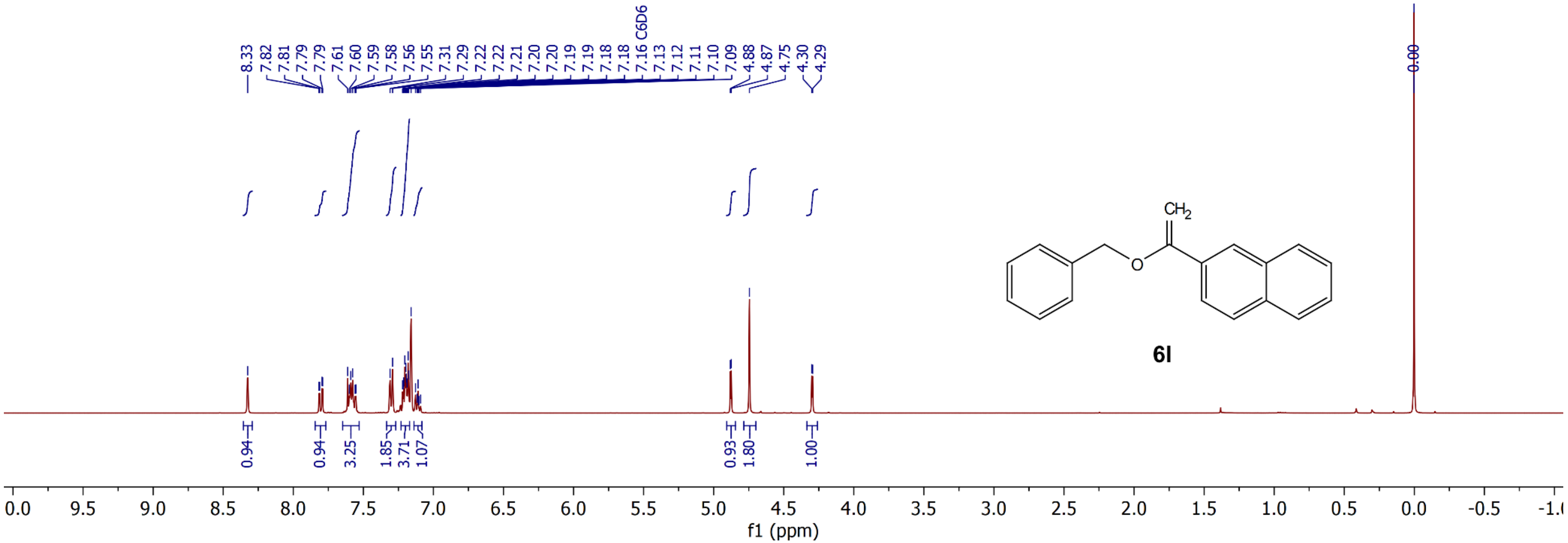

$^{13}C\{^{1}H\}$-NMR (101 MHz, C_6D_6) spectrum of compound **6l** (procedure GP1)

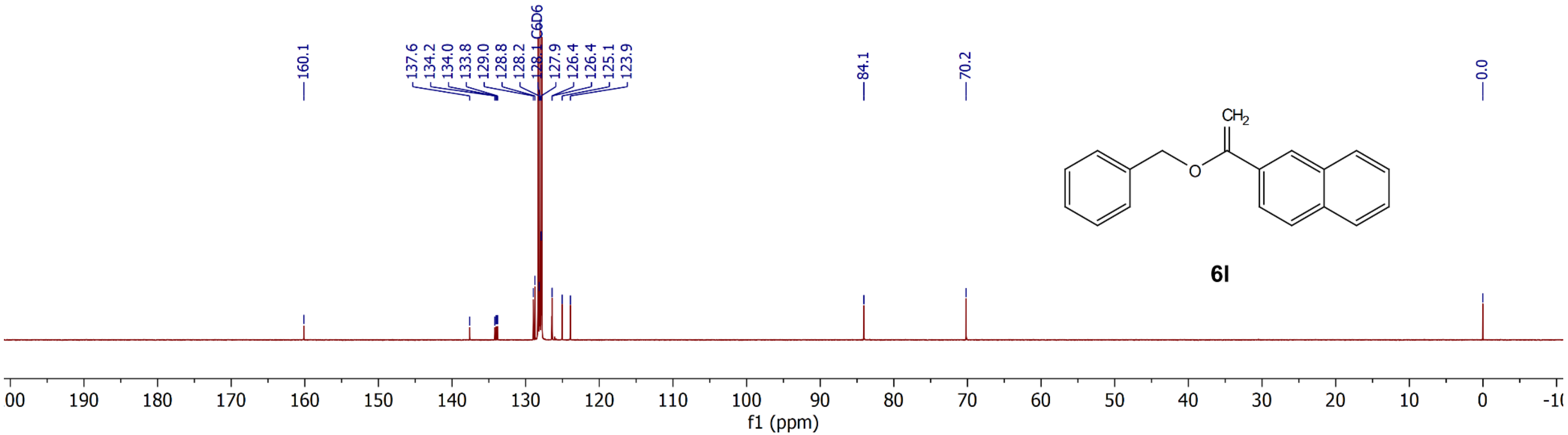

^{1}H-NMR (400 MHz, C_6D_6) spectrum of compound **6m** (procedure GP1)

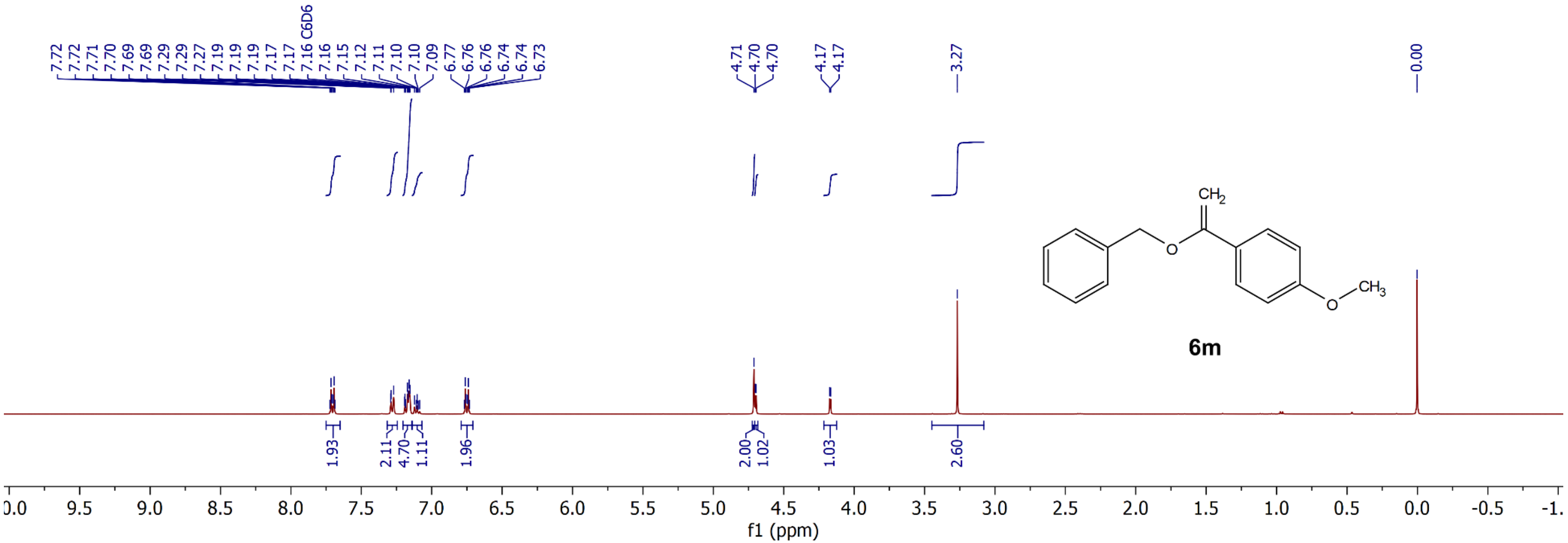

^{13}C{^{1}H}-NMR (101 MHz, C_6D_6) spectrum of compound **6m** (procedure GP1)

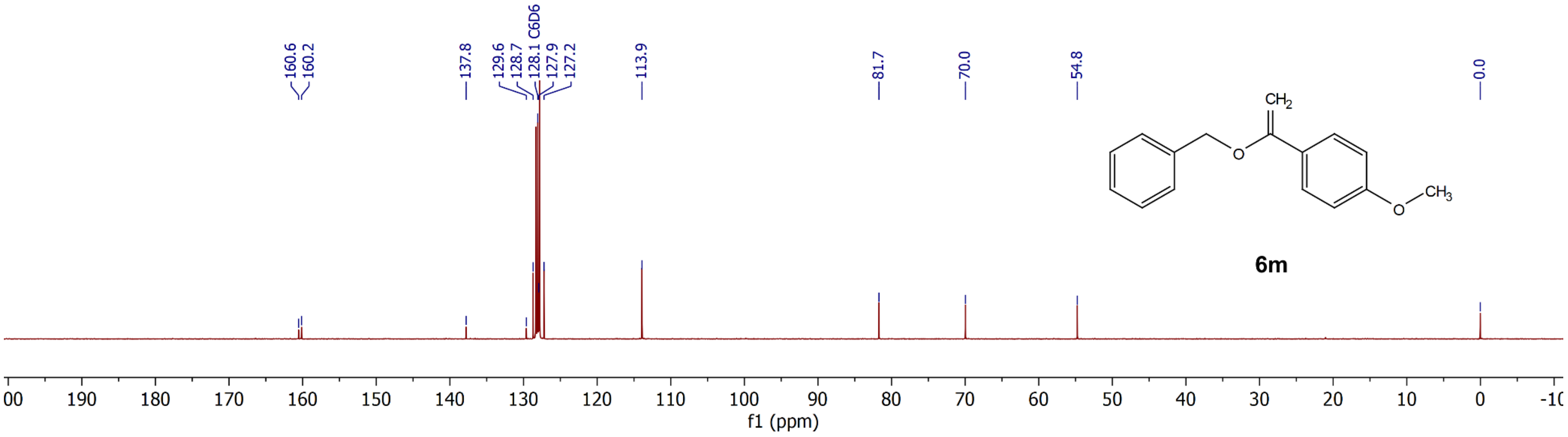

^{1}H-NMR (400 MHz, C_6D_6) spectrum of compound **6n** (procedure GP1)

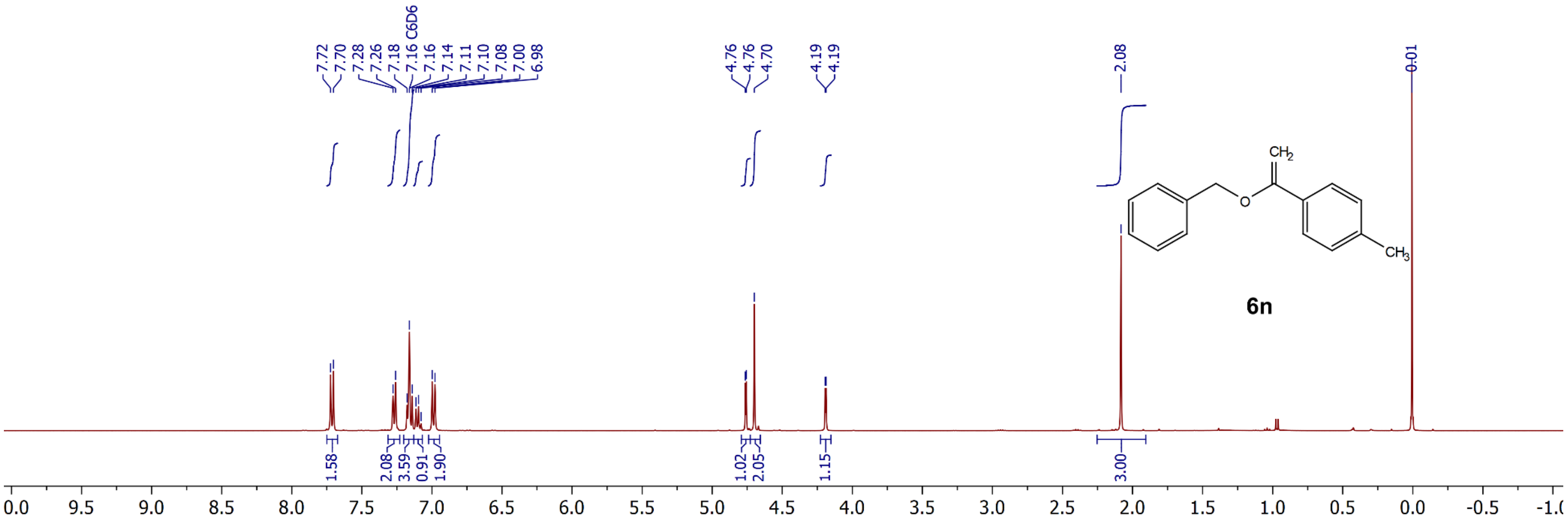

$^{13}C\{^{1}H\}$-NMR (101 MHz, C_6D_6) spectrum of compound **6n** (procedure GP1)

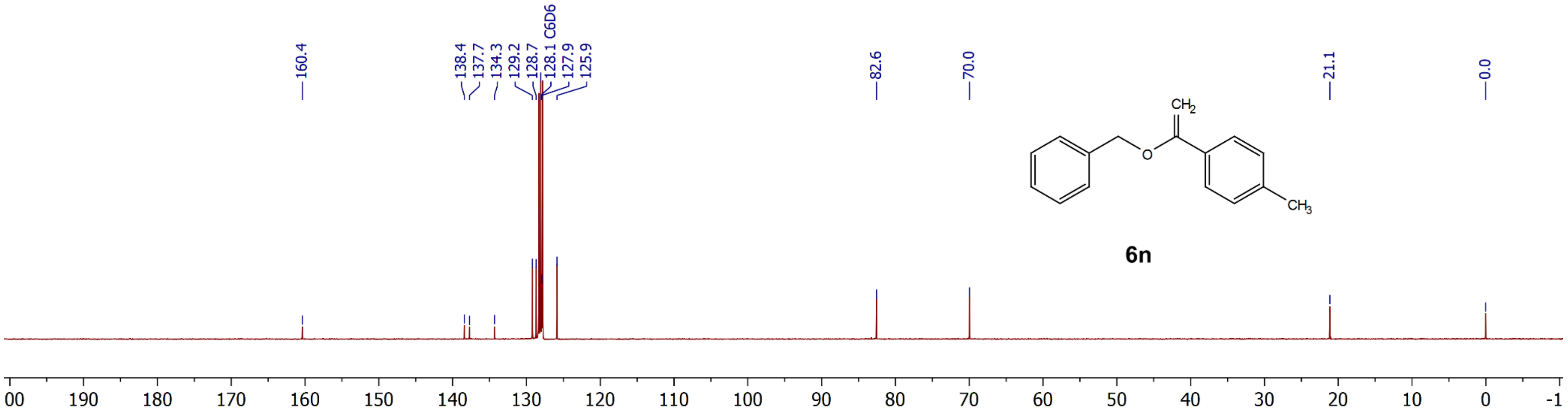

^1H-NMR (400 MHz, C_6D_6) spectrum of compound **6o** (procedure GP1)

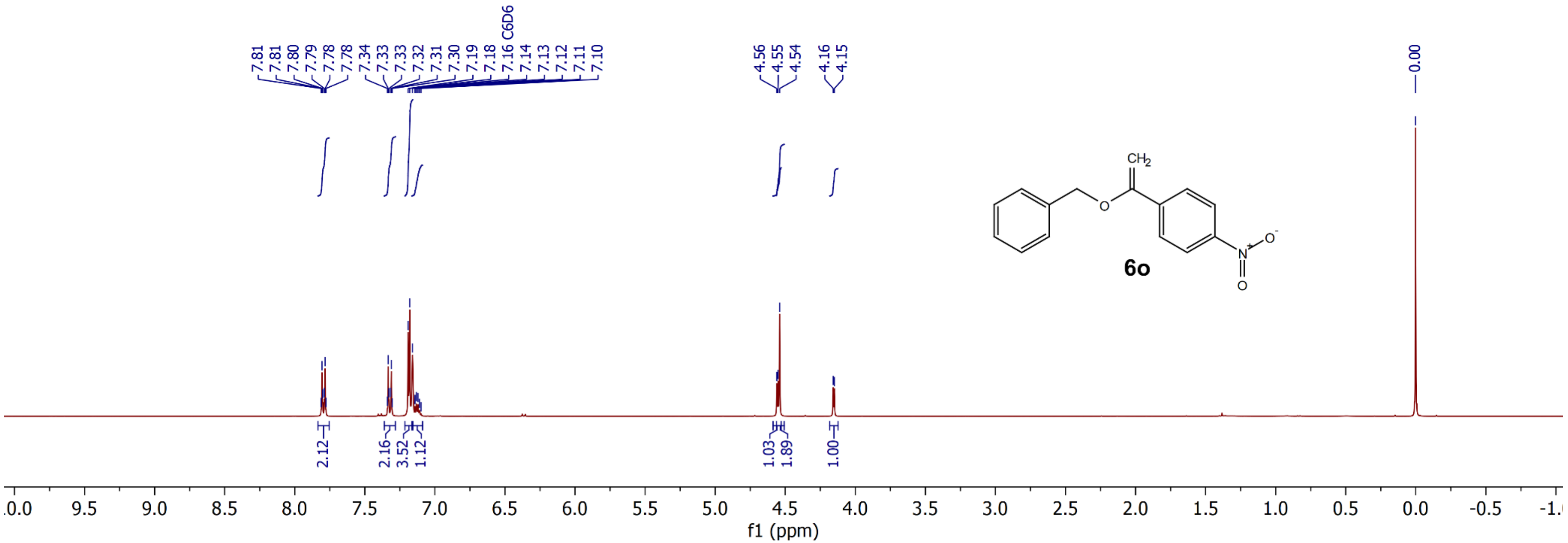

$^{13}C\{^1H\}$-NMR (101 MHz, C_6D_6) spectrum of compound **6o** (procedure GP1)

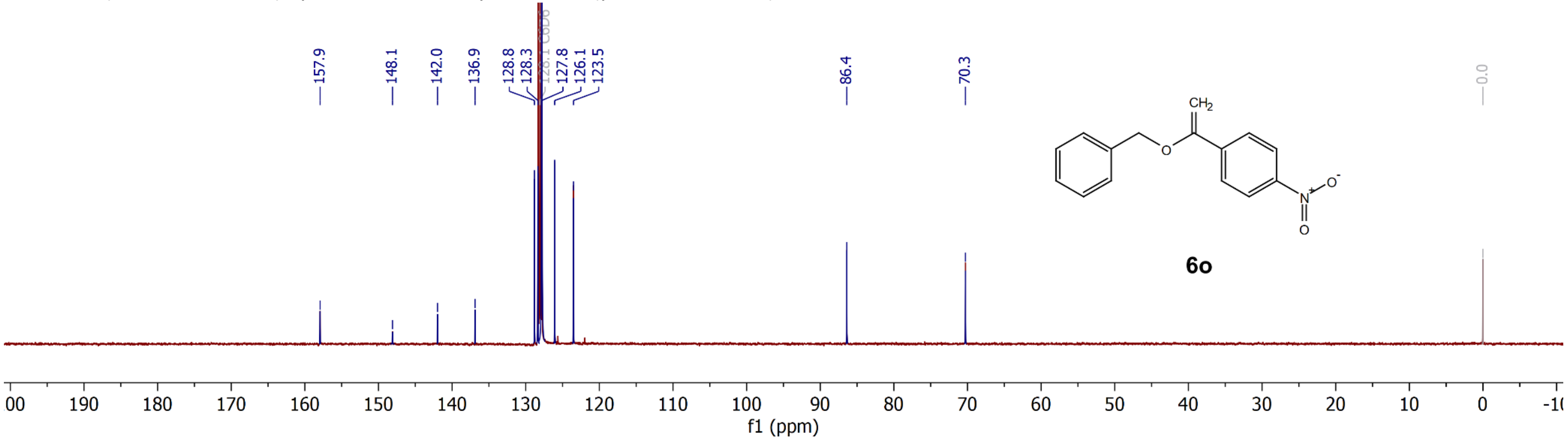

^{1}H-NMR (600 MHz, C_6D_6) spectrum of compound **6p** (procedure GP1)

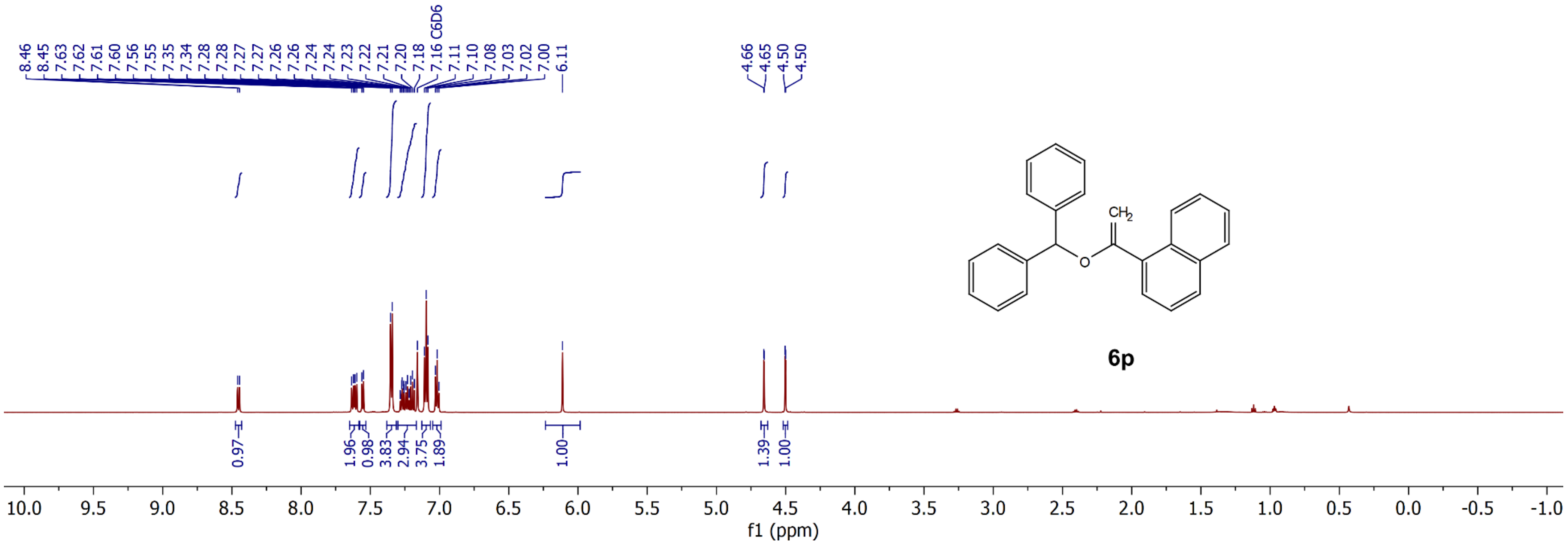

^{13}C{^{1}H}-NMR (151 MHz, C_6D_6) spectrum of compound **6p** (procedure GP1)

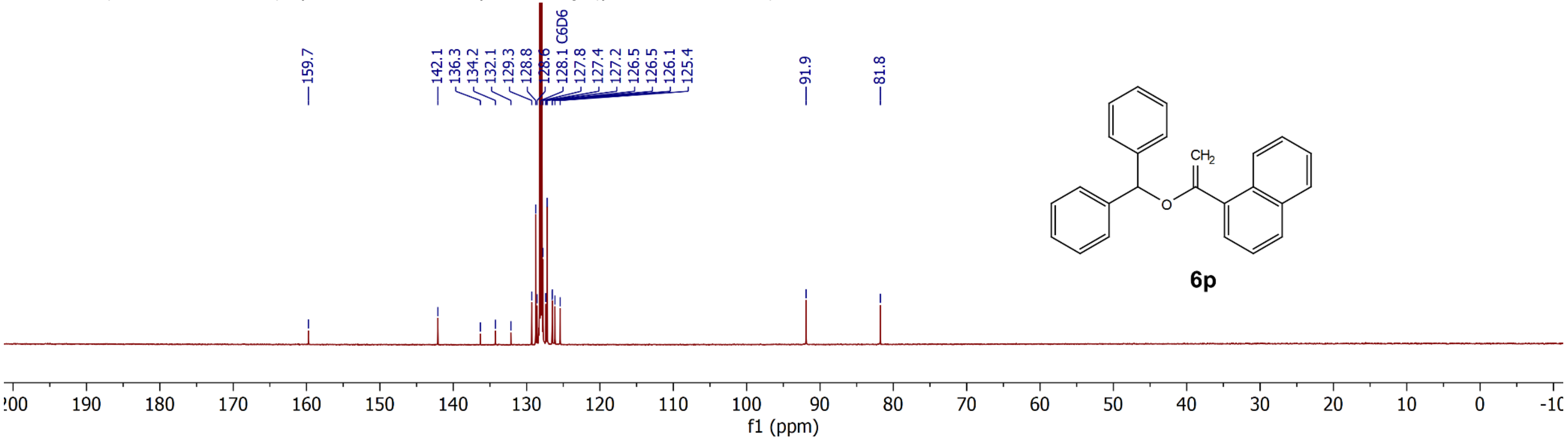

^{1}H-NMR (600 MHz, C_6D_6) spectrum of compound **6q** (procedure GP1)

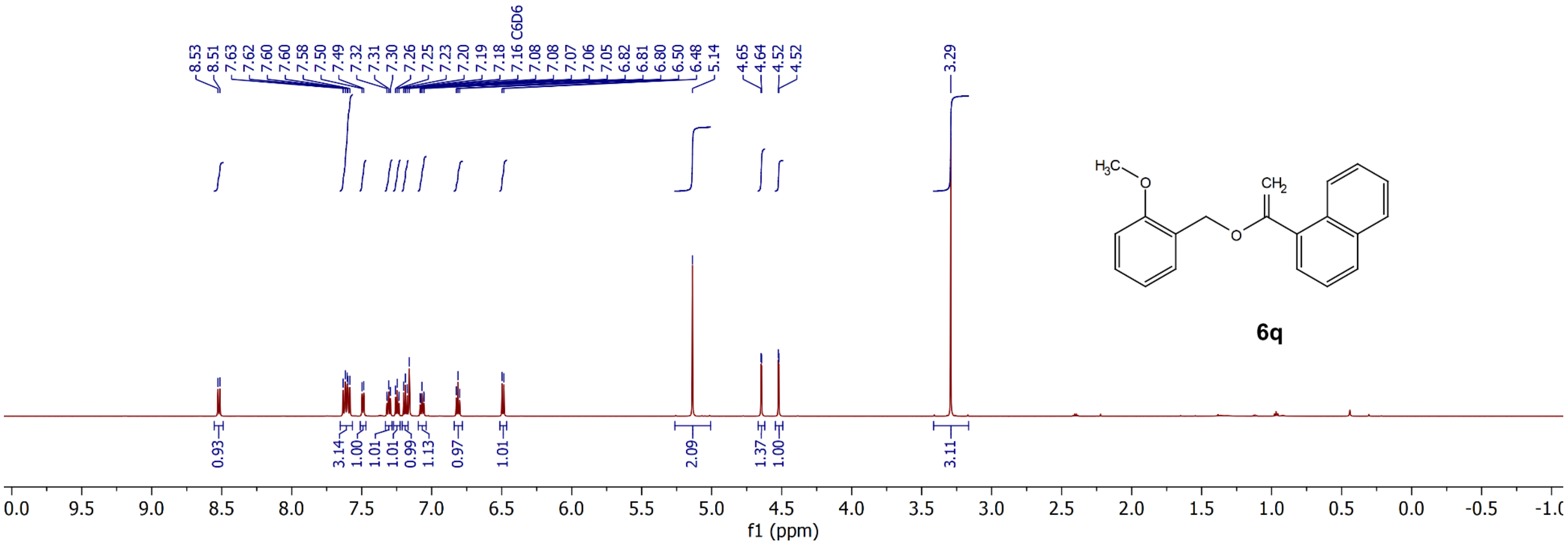

^{13}C{^{1}H}-NMR (151 MHz, C_6D_6) spectrum of compound **6q** (procedure GP1)

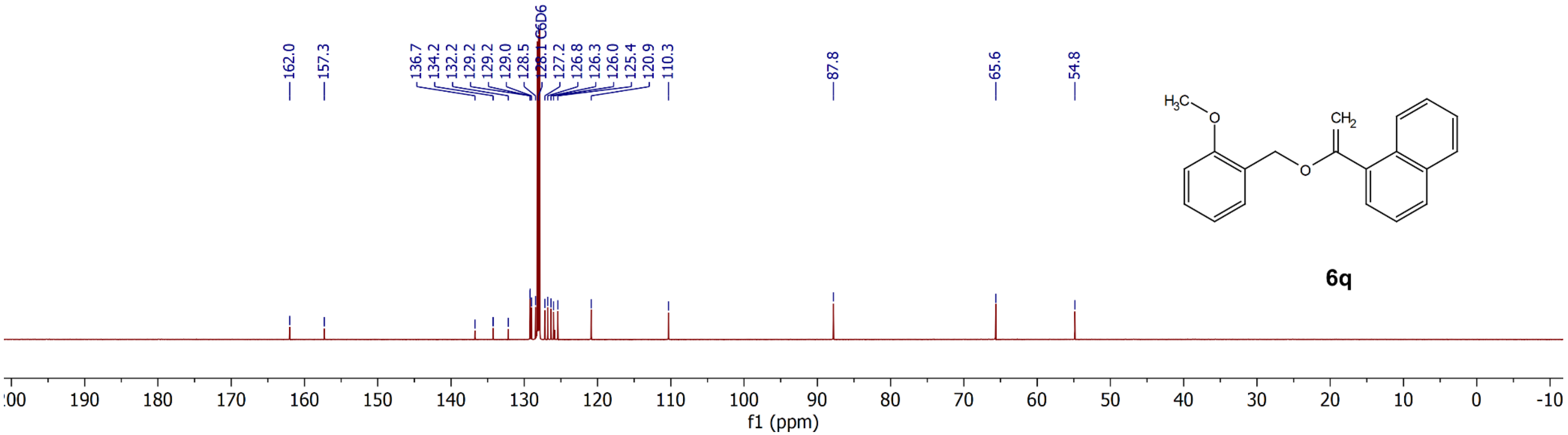

^{1}H-NMR (600 MHz, C_6D_6) spectrum of compound **6r** (procedure GP1)

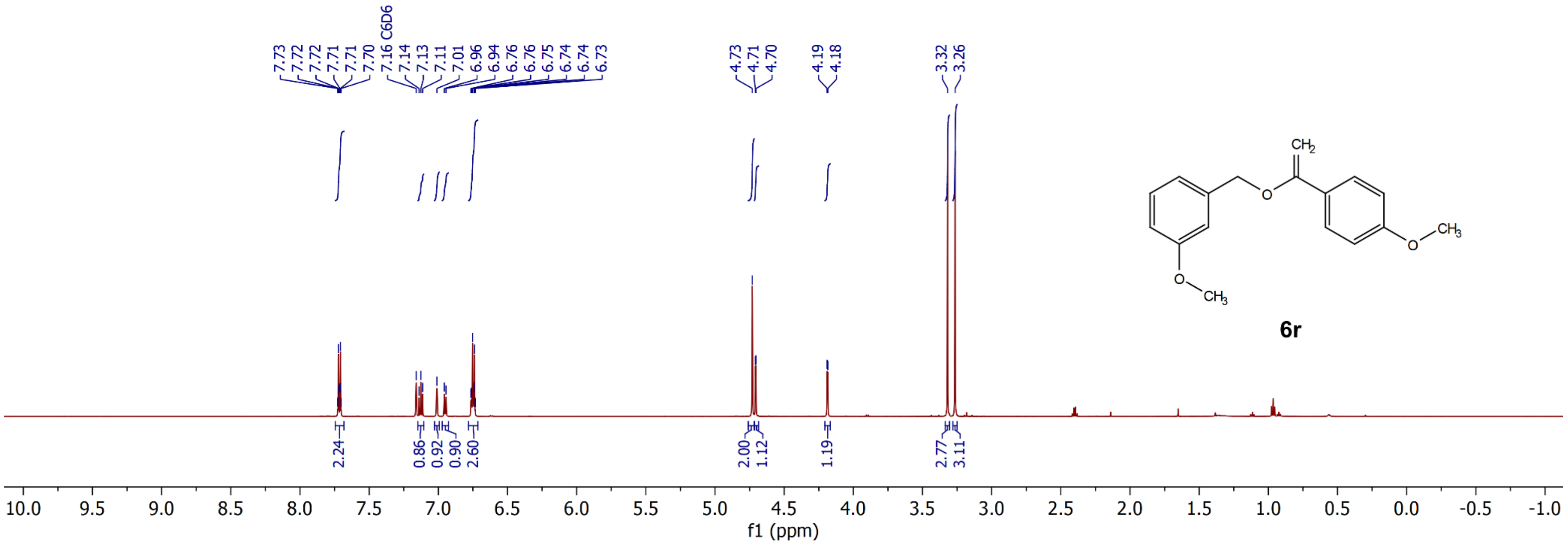

$^{13}C\{^{1}H\}$-NMR (151 MHz, C_6D_6) spectrum of compound **6r** (procedure GP1)

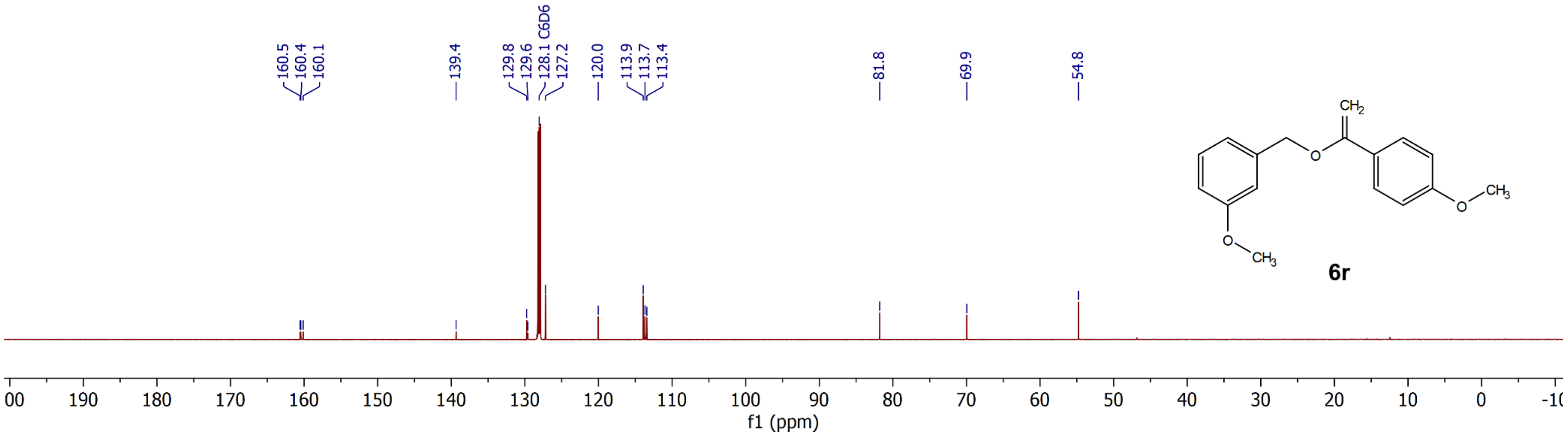

^{1}H-NMR (600 MHz, C_6D_6) spectrum of compound **6s** (procedure GP1)

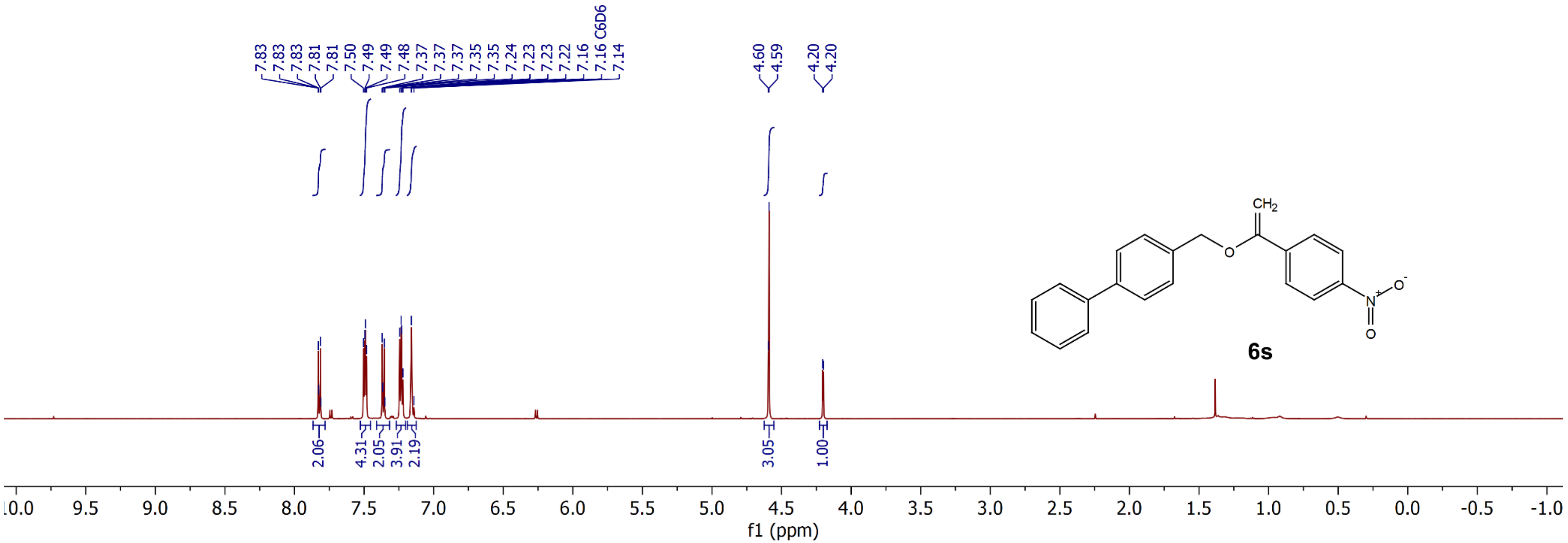

^{13}C{^{1}H}-NMR (151 MHz, C_6D_6) spectrum of compound **6s** (procedure GP1)

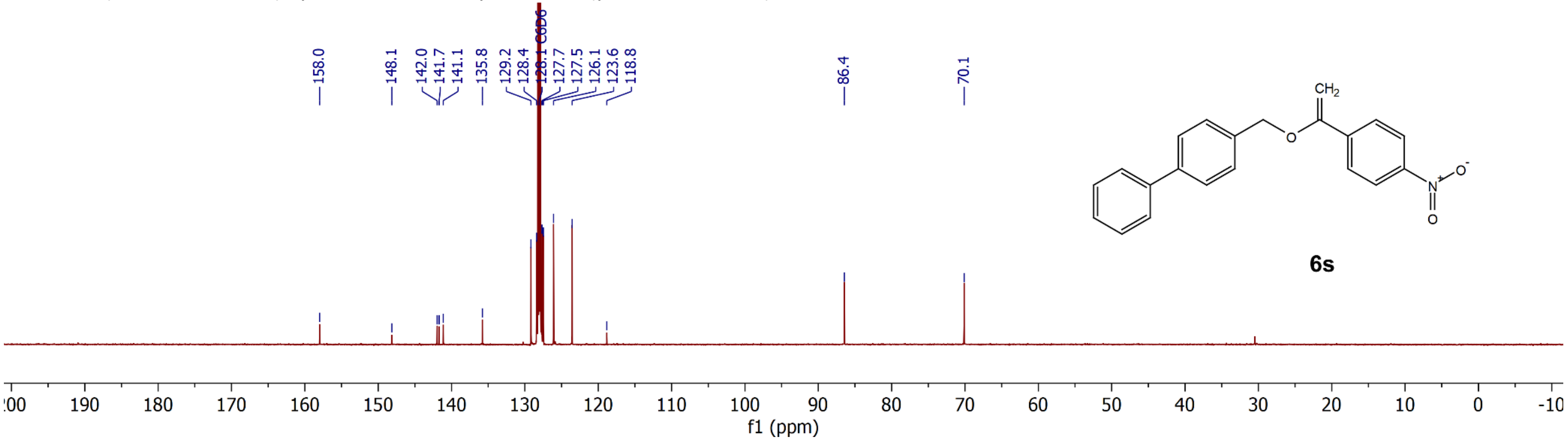

^{1}H-NMR (400 MHz, C_6D_6) spectrum of regioisomers mixture **6a/7a** (procedure GP2)

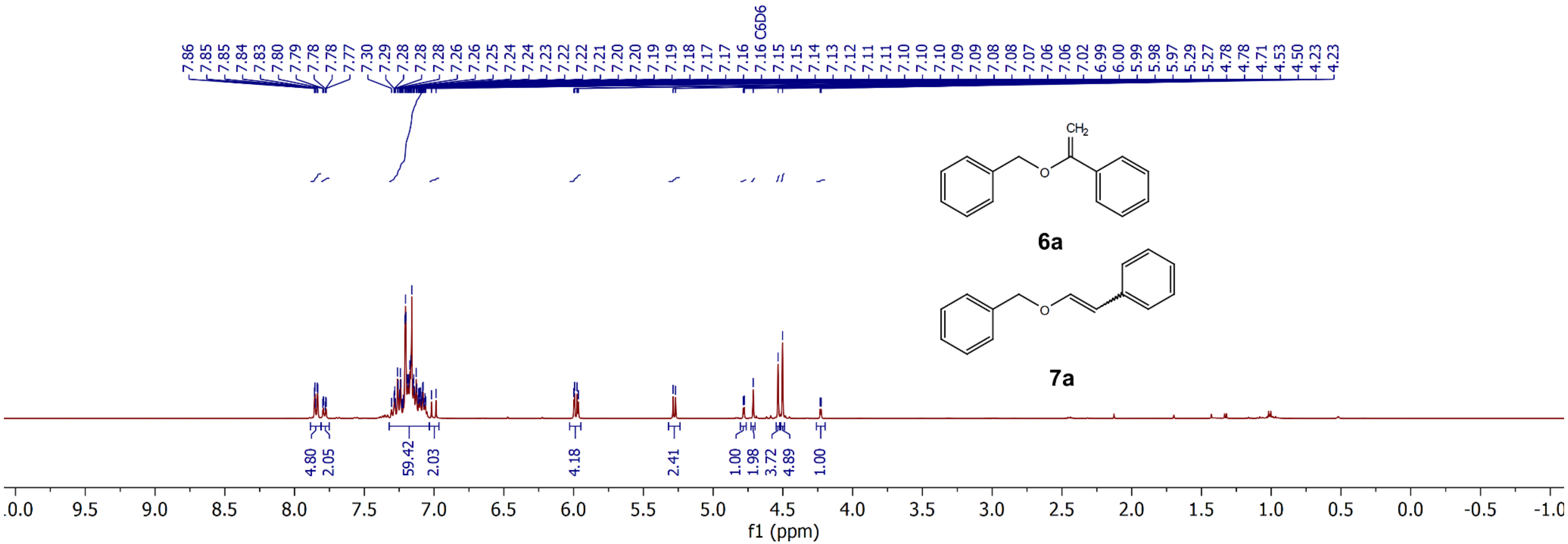

$^{13}C\{^{1}H\}$-NMR (101 MHz, C_6D_6) spectrum of regioisomers mixture **6a/7a** (procedure GP2)

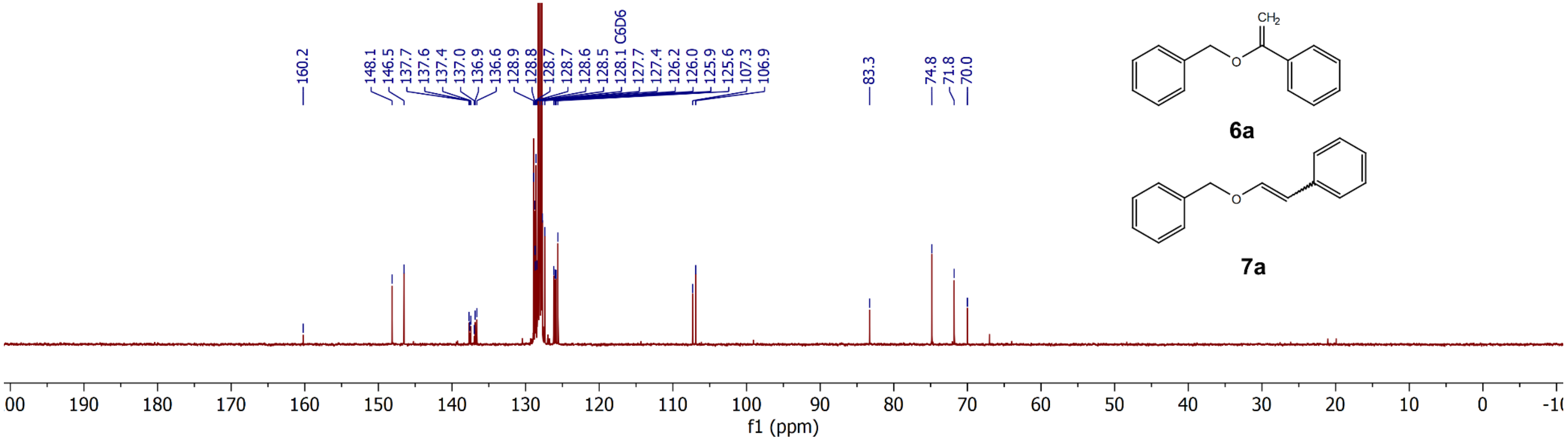

^{1}H-NMR (600 MHz, C_6D_6) spectrum of regioisomers mixture **6b/7b** (procedure GP2)

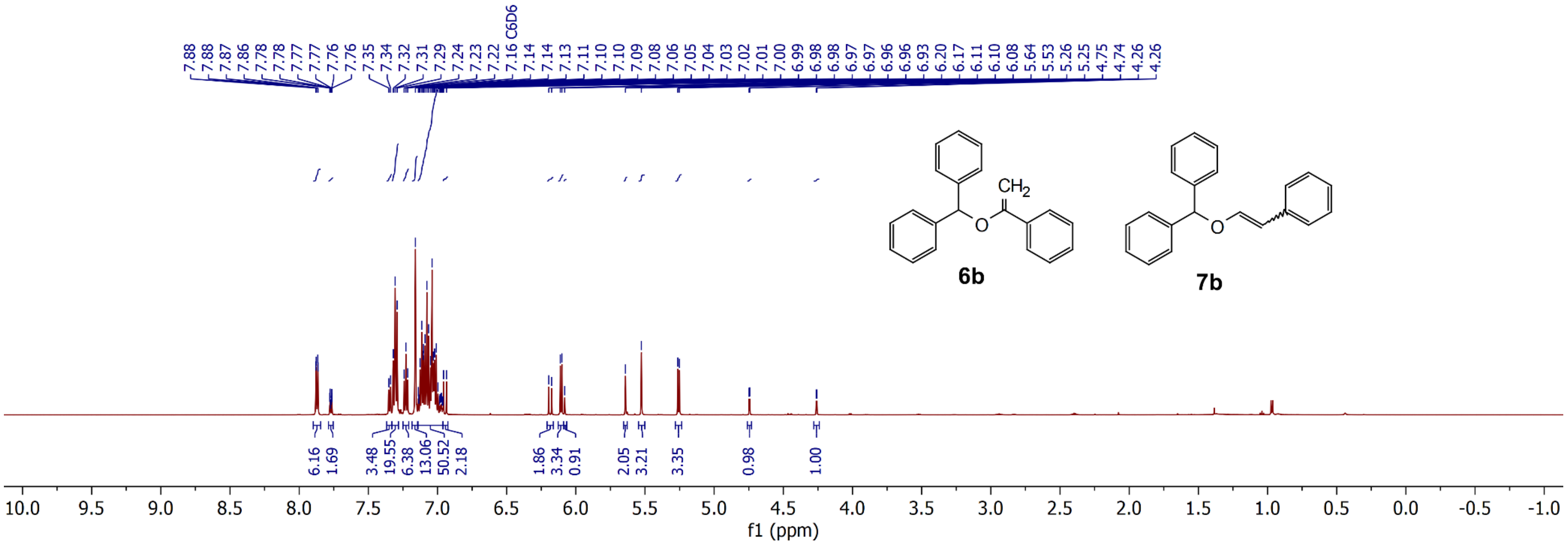

$^{13}C\{^{1}H\}$-NMR (151 MHz, C_6D_6) spectrum of regioisomers mixture **6b/7b** (procedure GP2)

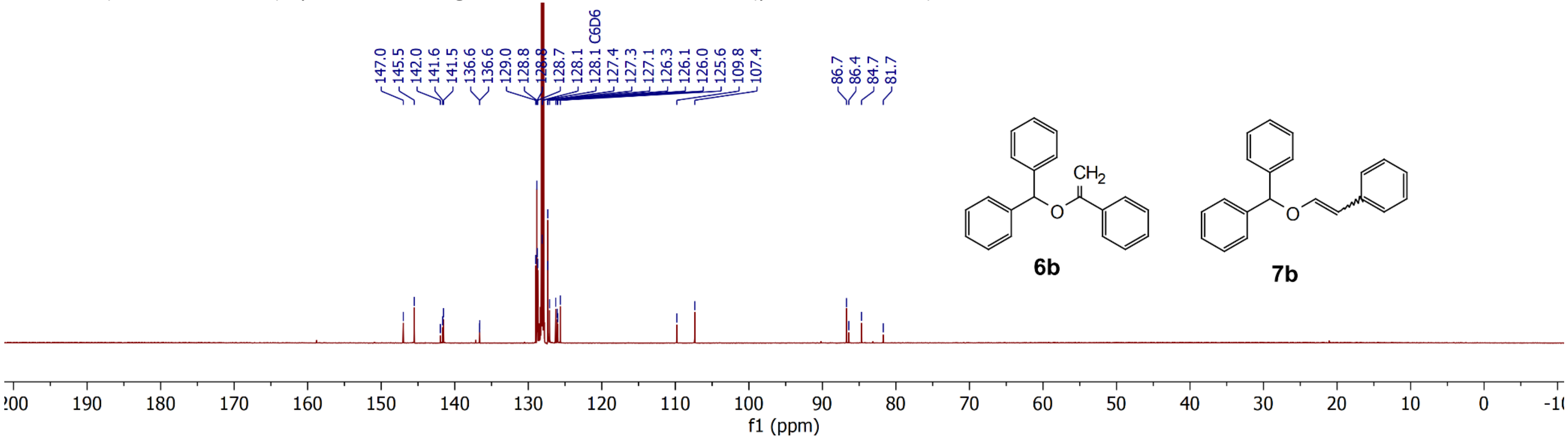

^{1}H-NMR (600 MHz, C_6D_6) spectrum of regioisomers mixture **6c/7c** (procedure GP2)

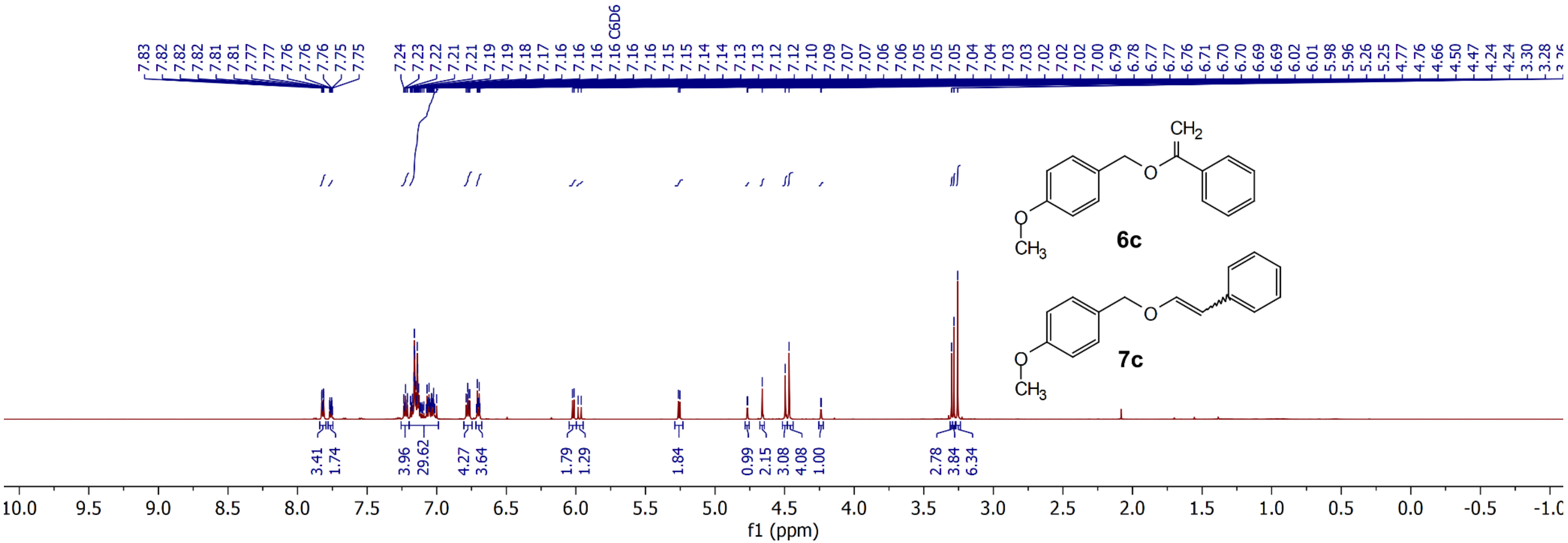

$^{13}C\{^{1}H\}$-NMR (151 MHz, C_6D_6) spectrum of regioisomers mixture **6c/7c** (procedure GP2)

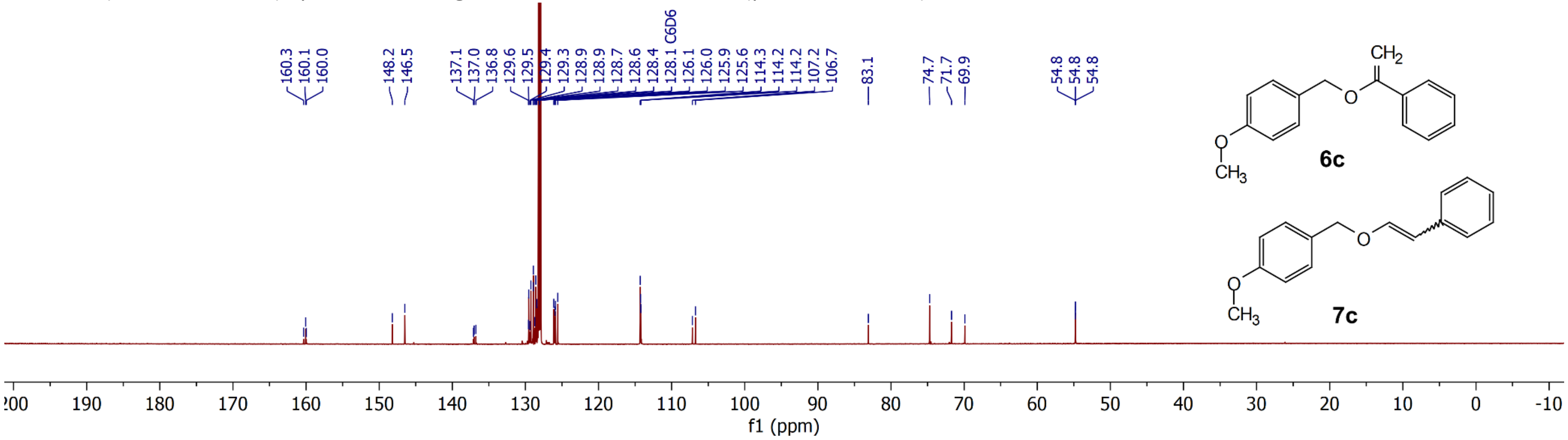

^{1}H-NMR (400 MHz, DMSO-d_6) spectrum of regioisomers mixture **6e/7e** (procedure GP2)

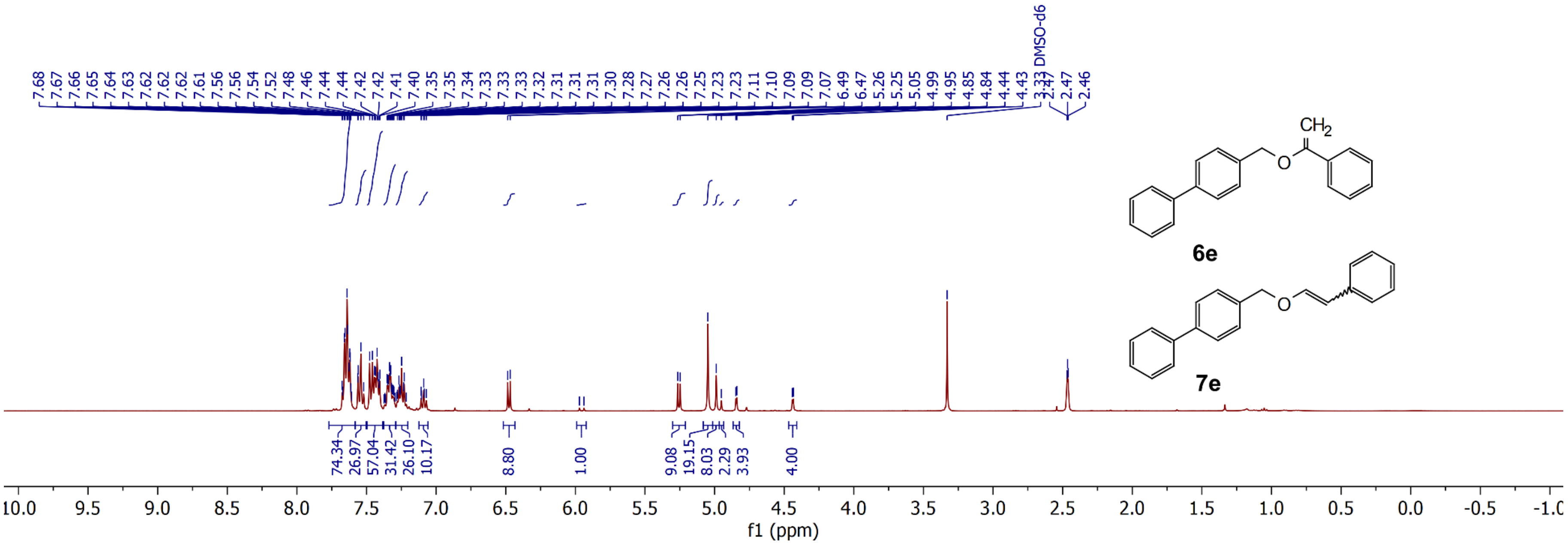

6e

7e

^{13}C{^{1}H}-NMR (101 MHz, DMSO-d_6) spectrum of regioisomers mixture **6e/7e** (procedure GP2)

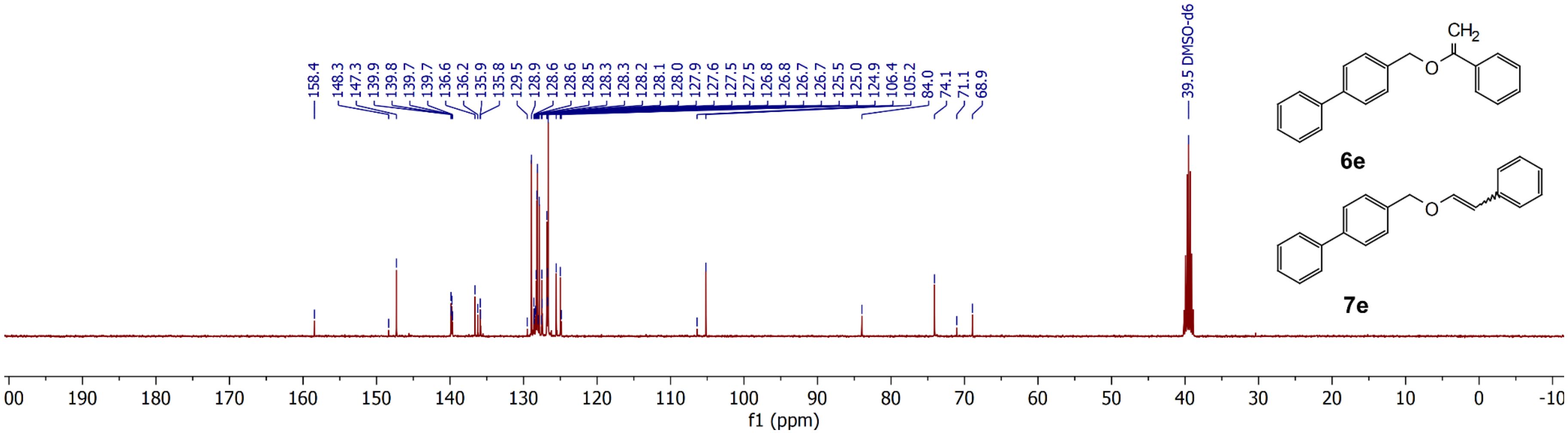

6e

7e

^{1}H-NMR (400 MHz, C_6D_6) spectrum of regioisomers mixture **6g/7g** (procedure GP2)

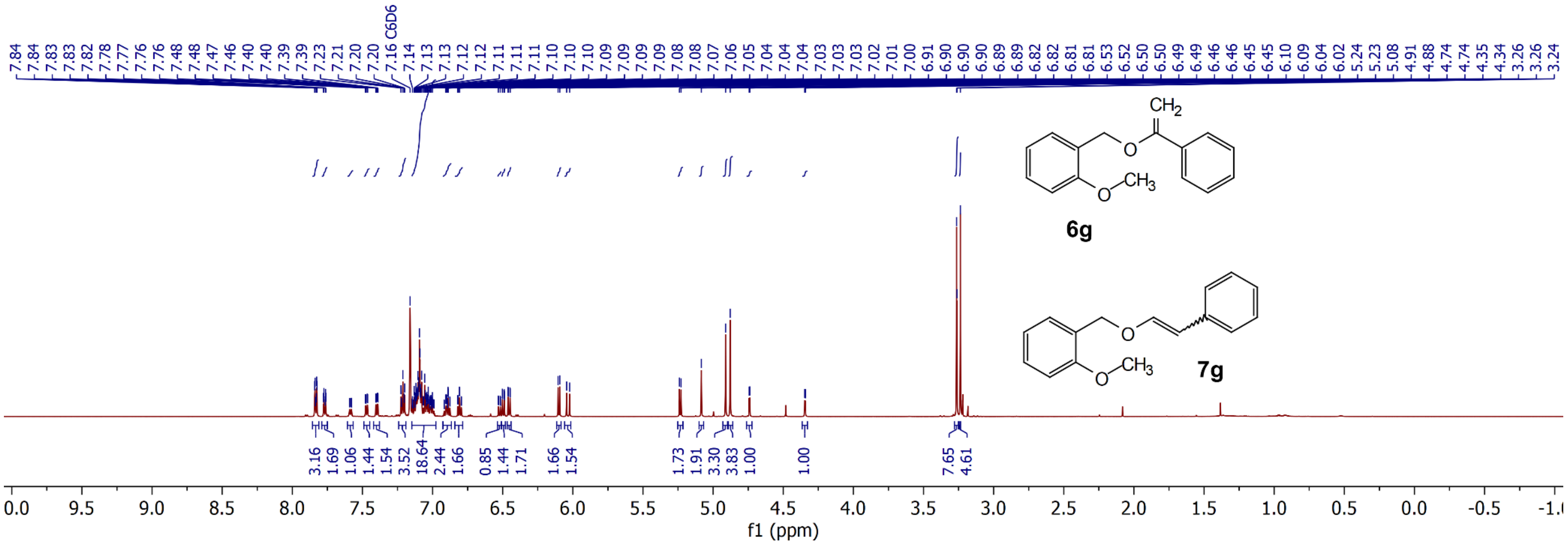

$^{13}C\{^{1}H\}$-NMR (101 MHz, C_6D_6) spectrum of regioisomers mixture **6g/7g** (procedure GP2)

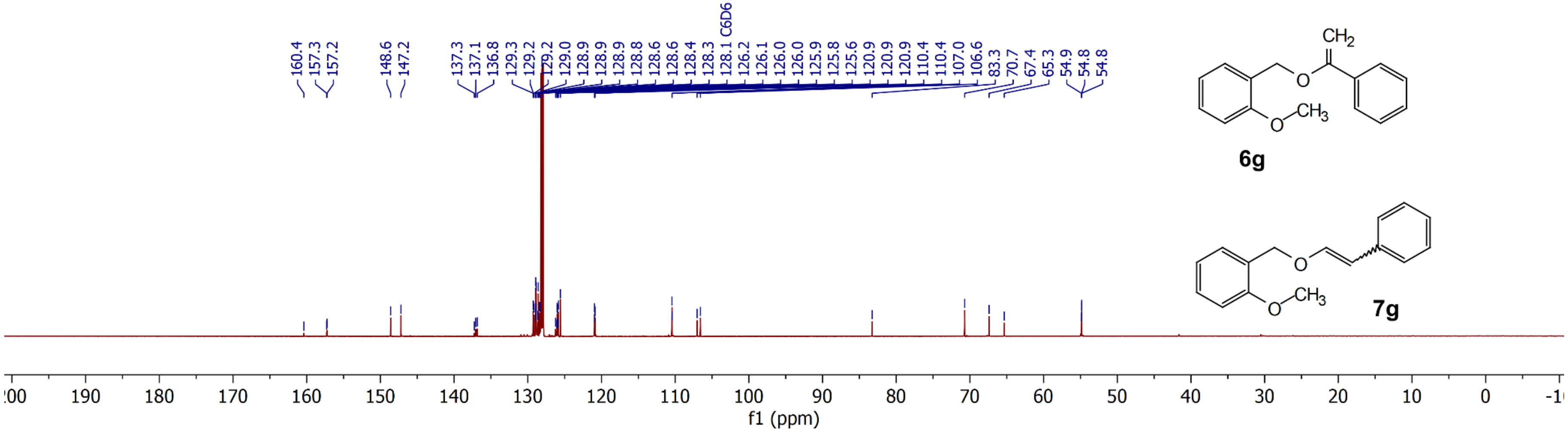

^{1}H-NMR (600 MHz, C_6D_6) spectrum of regioisomers mixture **6h/7h** (procedure GP2)

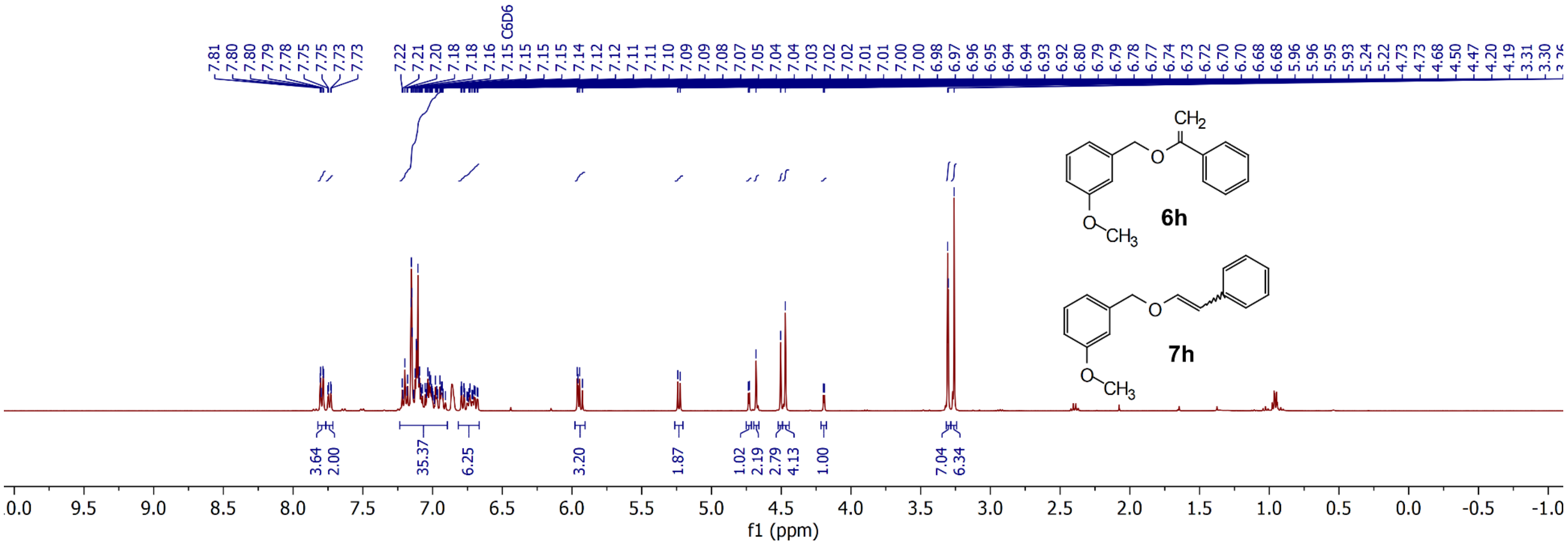

$^{13}C\{^{1}H\}$-NMR (151 MHz, C_6D_6) spectrum of regioisomers mixture **6h/7h** (procedure GP2)

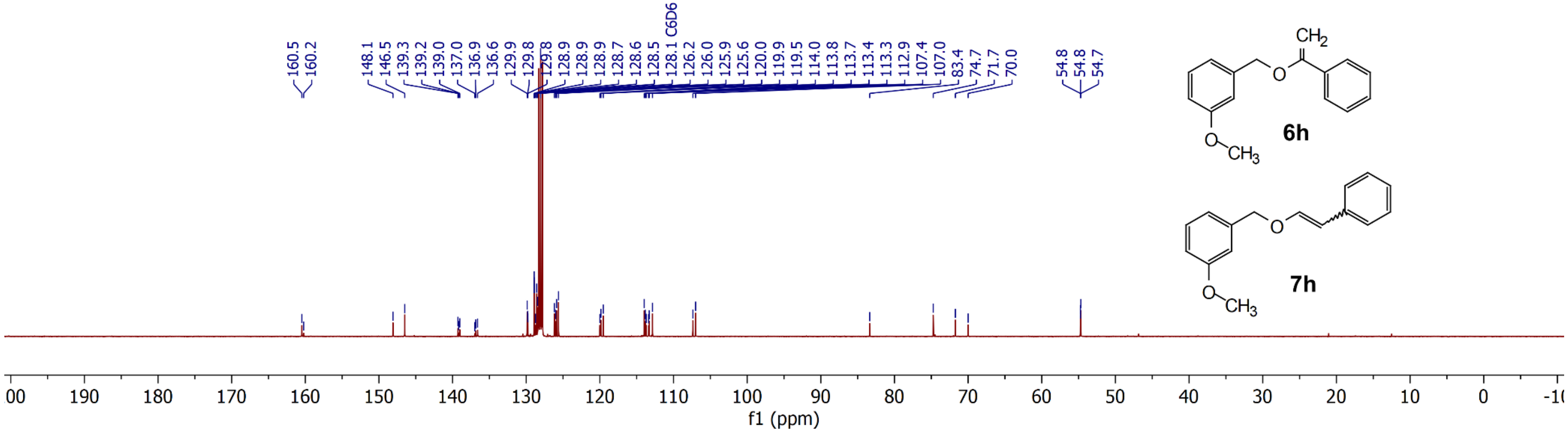

^{1}H-NMR (400 MHz, C_6D_6) spectrum of compound **7i** (procedure GP2)

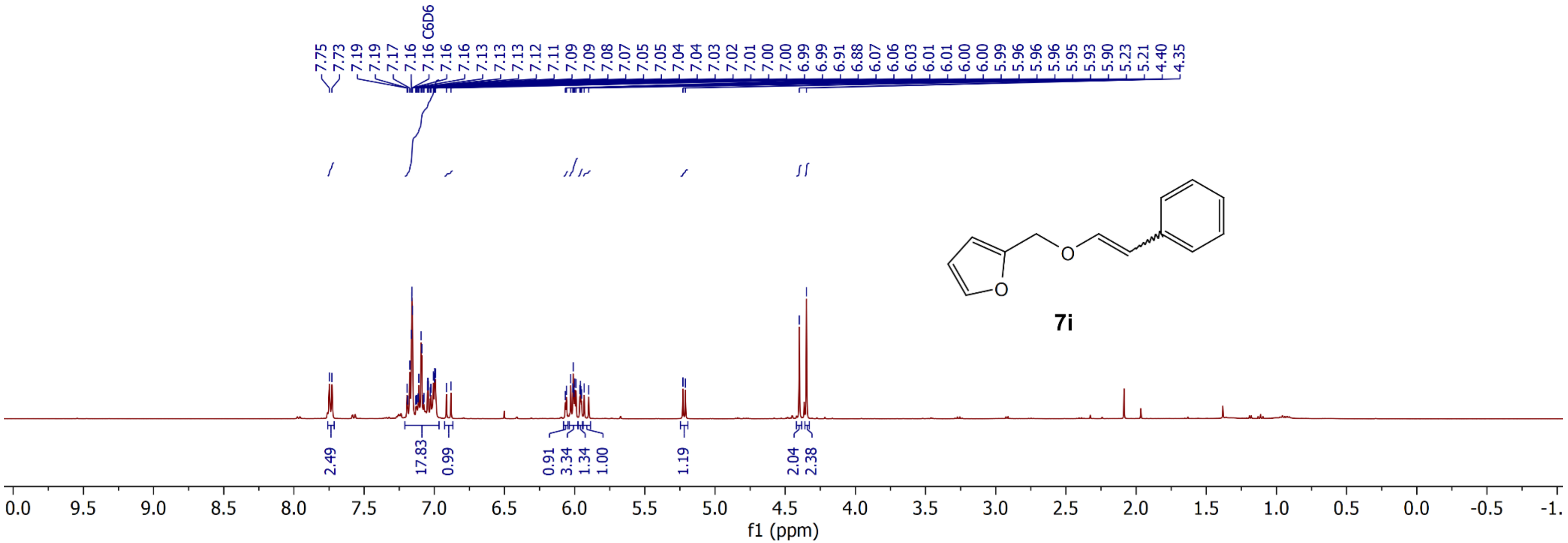

^{13}C{^{1}H}-NMR (101 MHz, C_6D_6) spectrum of compound **7i** (procedure GP2)

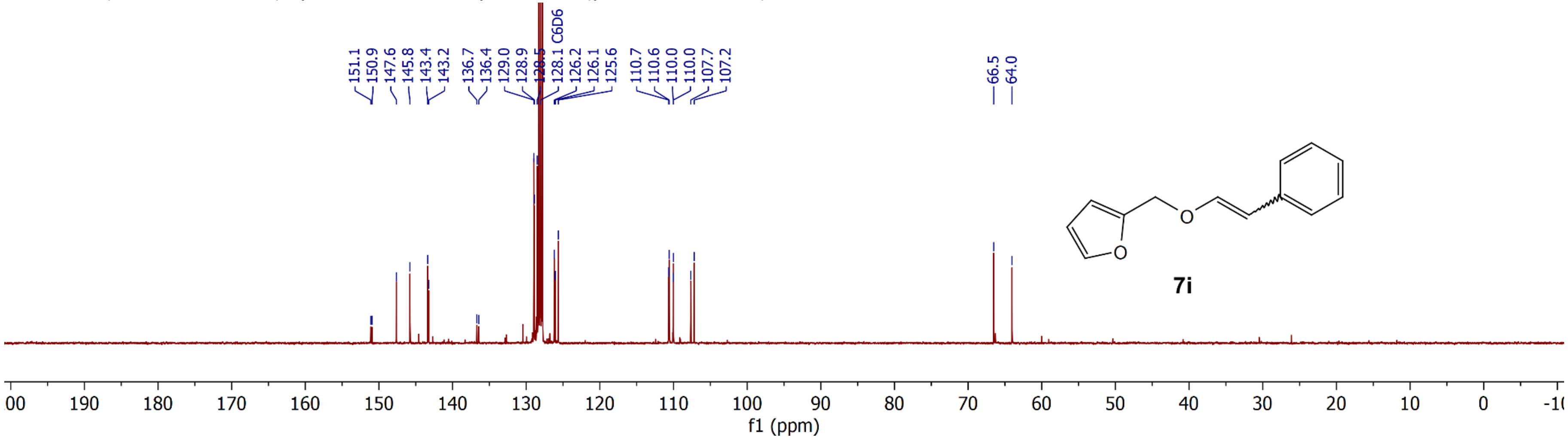

^{1}H-NMR (600 MHz, C_6D_6) spectrum of regioisomers mixture **6m/7m** (procedure GP2)

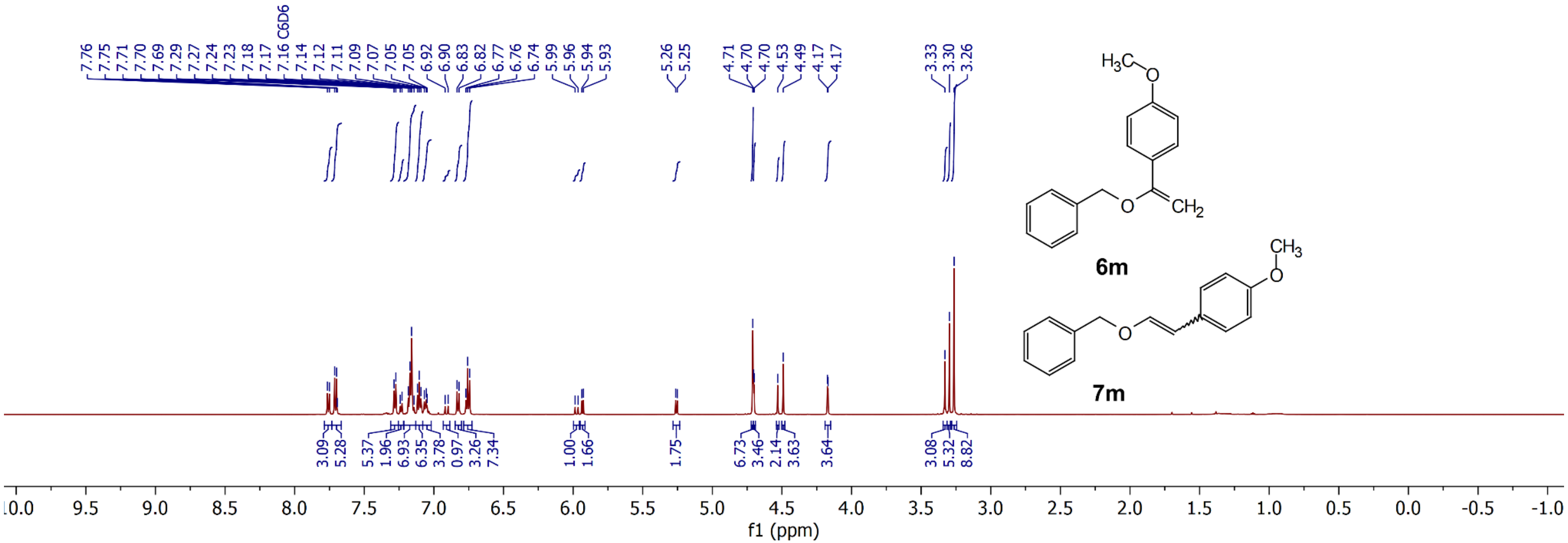

^{13}C{^{1}H}-NMR (151 MHz, C_6D_6) spectrum of regioisomers mixture **6m/7m** (procedure GP2)

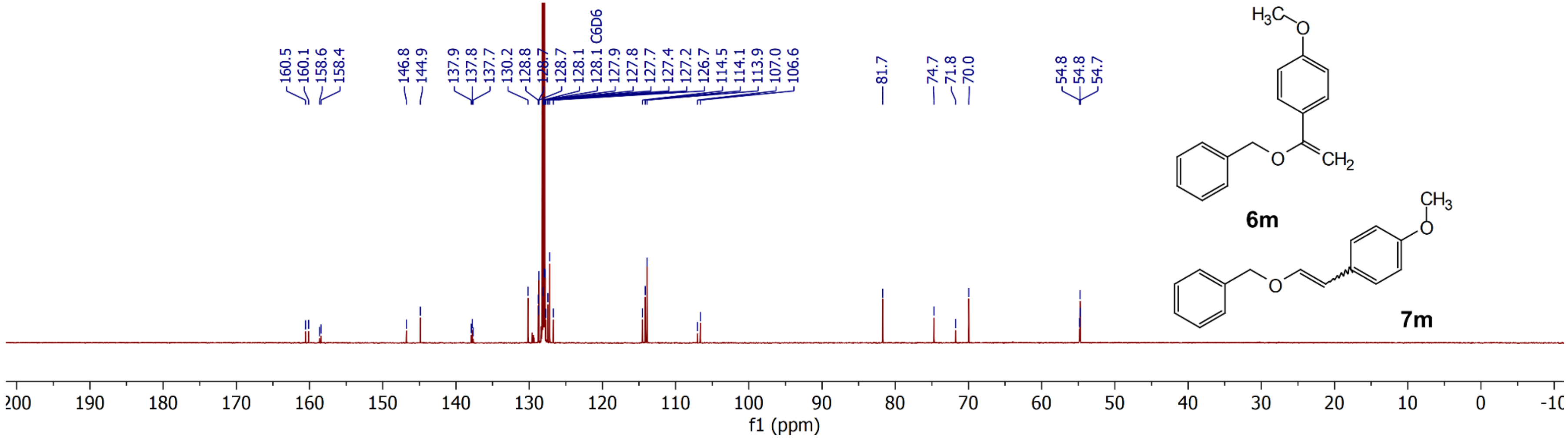

^{1}H-NMR (600 MHz, C_6D_6) spectrum of regioisomers mixture **6n/7n** (procedure GP2)

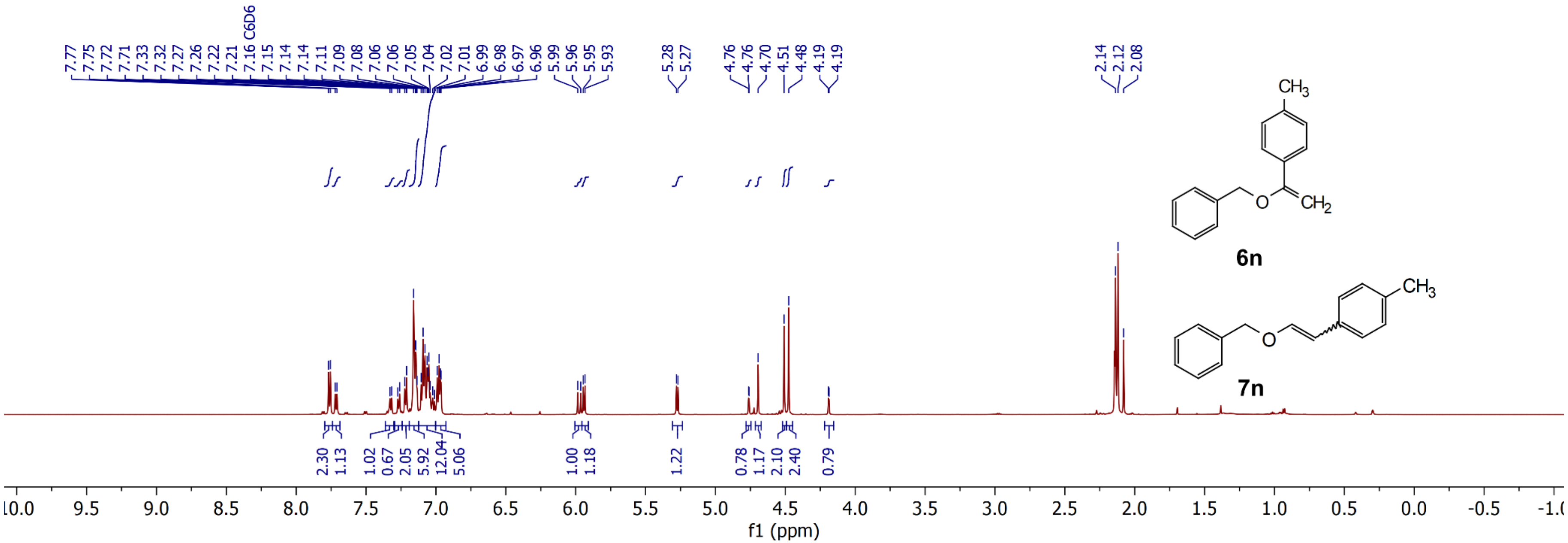

$^{13}C\{^{1}H\}$-NMR (151 MHz, C_6D_6) spectrum of regioisomers mixture **6n/7n** (procedure GP2)

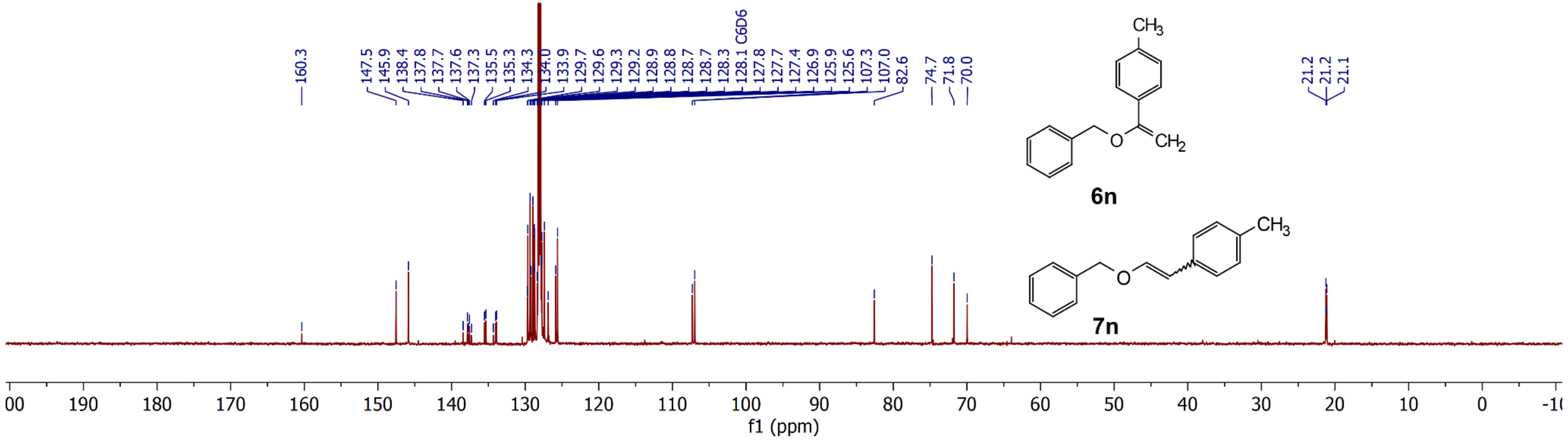

^{1}H-NMR (600 MHz, C_6D_6) spectrum of compound **7o** (procedure GP2)

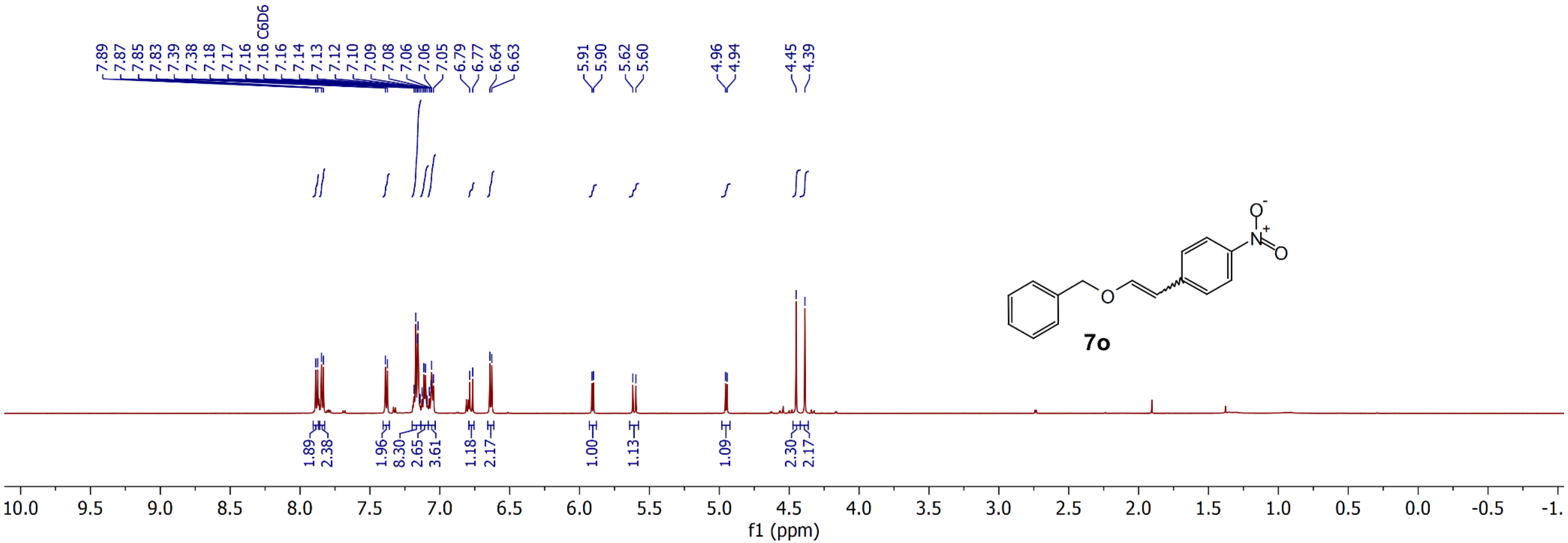

^{13}C{^{1}H}-NMR (151 MHz, C_6D_6) spectrum of rcompound **7o** (procedure GP2)

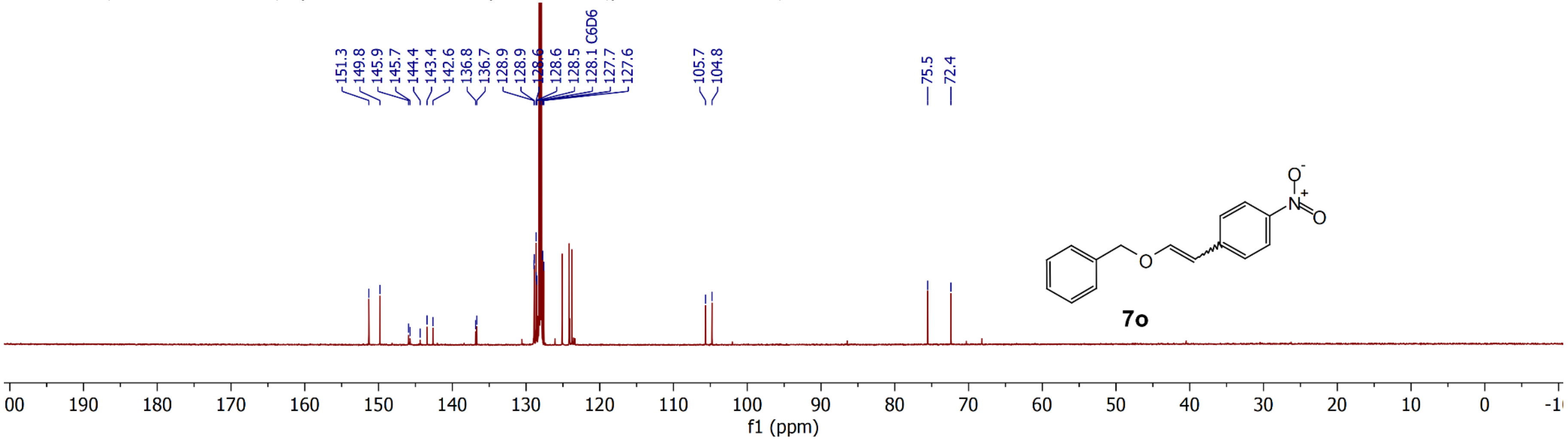

^{1}H-NMR (600 MHz, C_6D_6) spectrum of compound **7t** (procedure GP2)

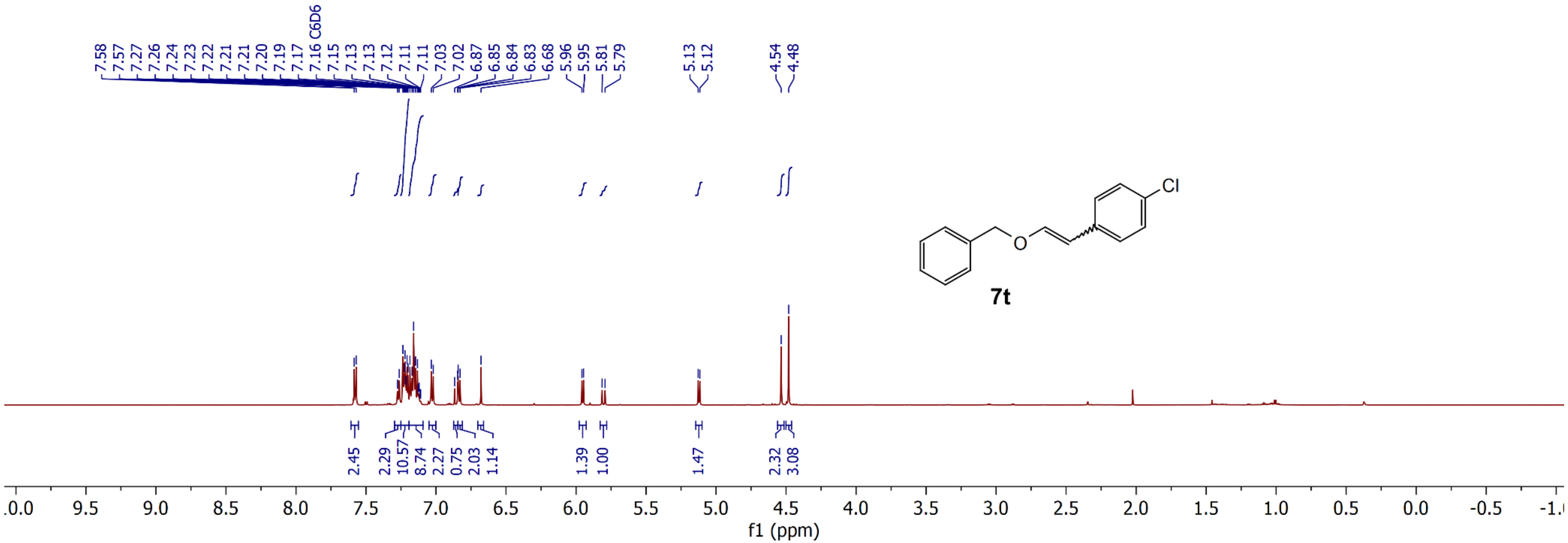

^{13}C{^{1}H}-NMR (151 MHz, C_6D_6) spectrum of compound **7t** (procedure GP2)

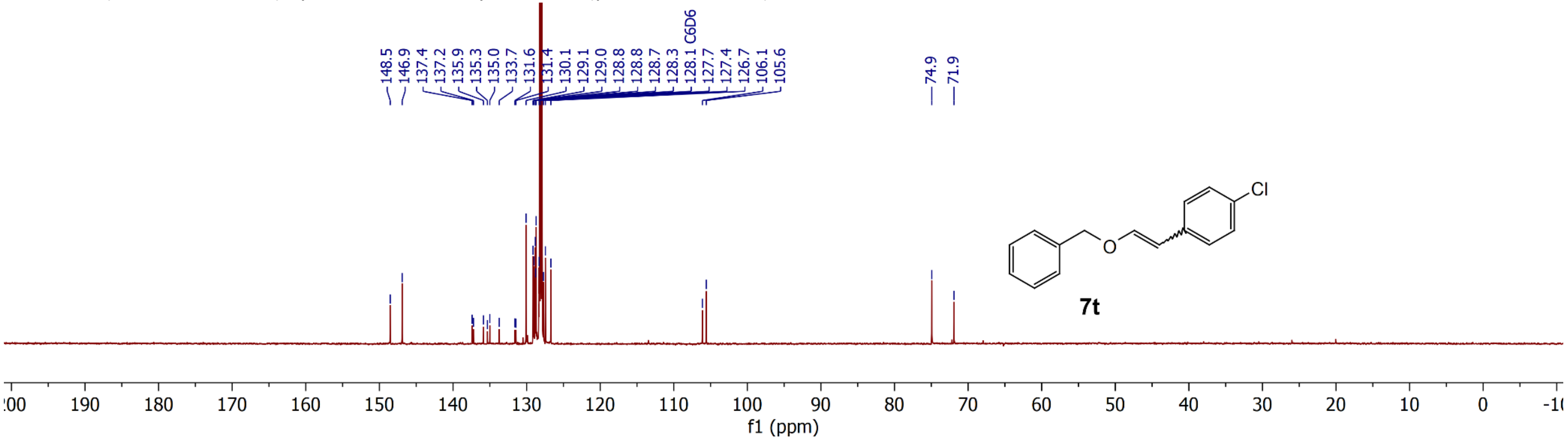

^{1}H-NMR (600 MHz, C_6D_6) spectrum of regioisomers mixture **6u/7u** (procedure GP2)

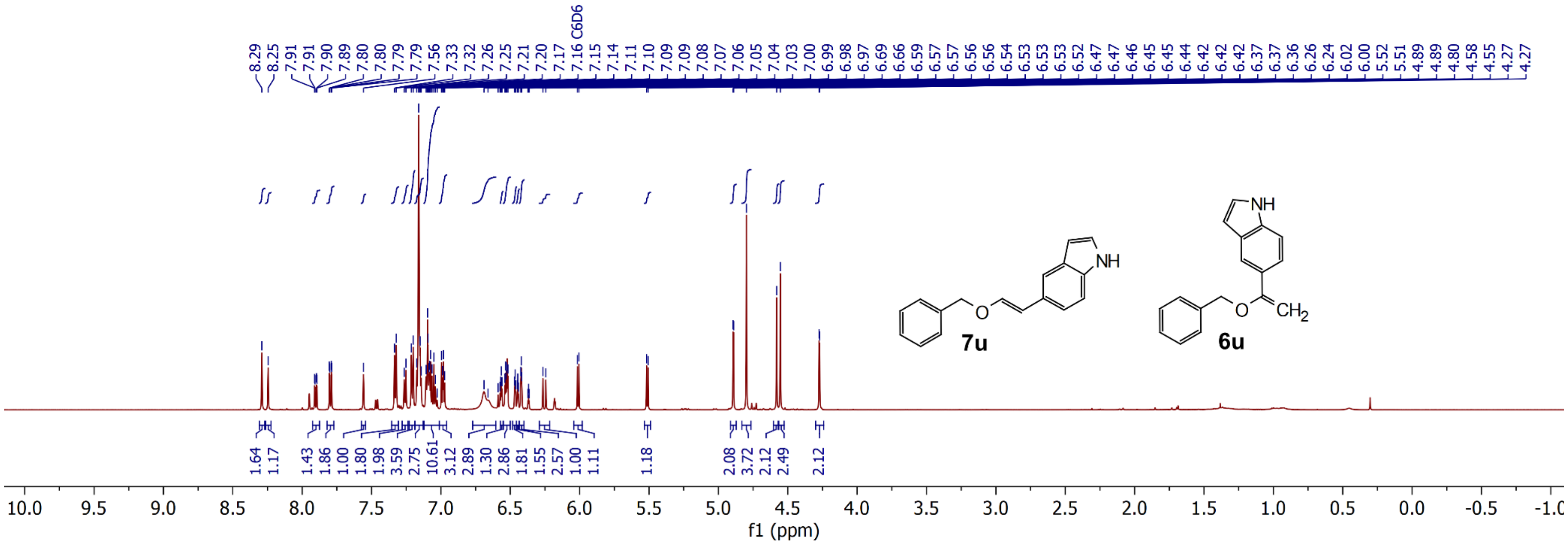

$^{13}C\{^{1}H\}$-NMR (151 MHz, C_6D_6) spectrum of regioisomers mixture **6u/7u** (procedure GP2)

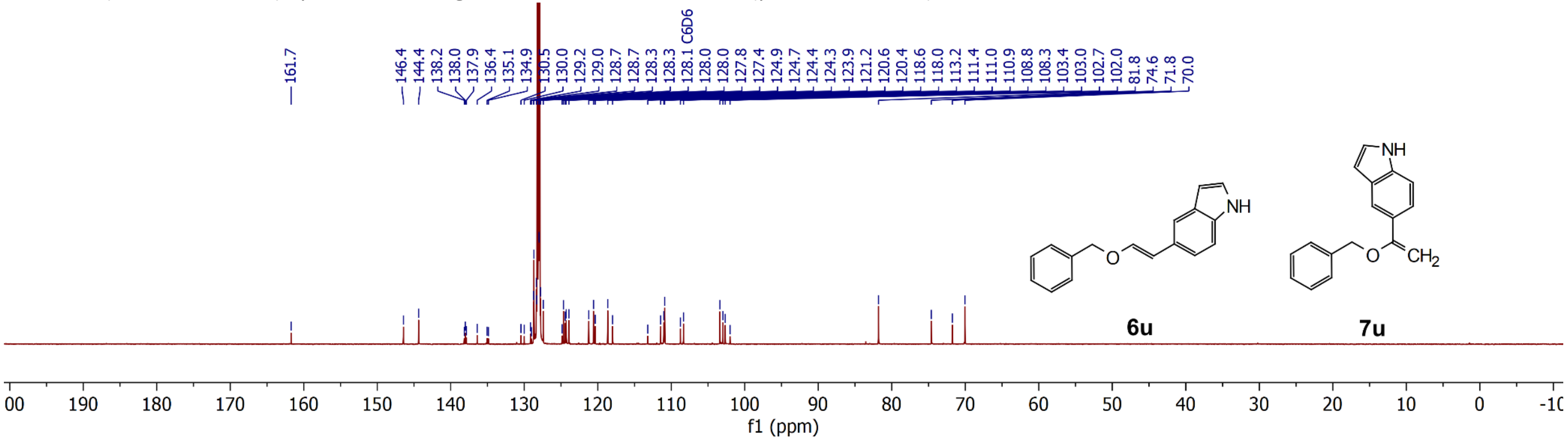

^{1}H-NMR (600 MHz, C_6D_6) spectrum of regioisomers mixture **6a/7a** (mixture used in the **7a** preparation, obtained during optimization process)

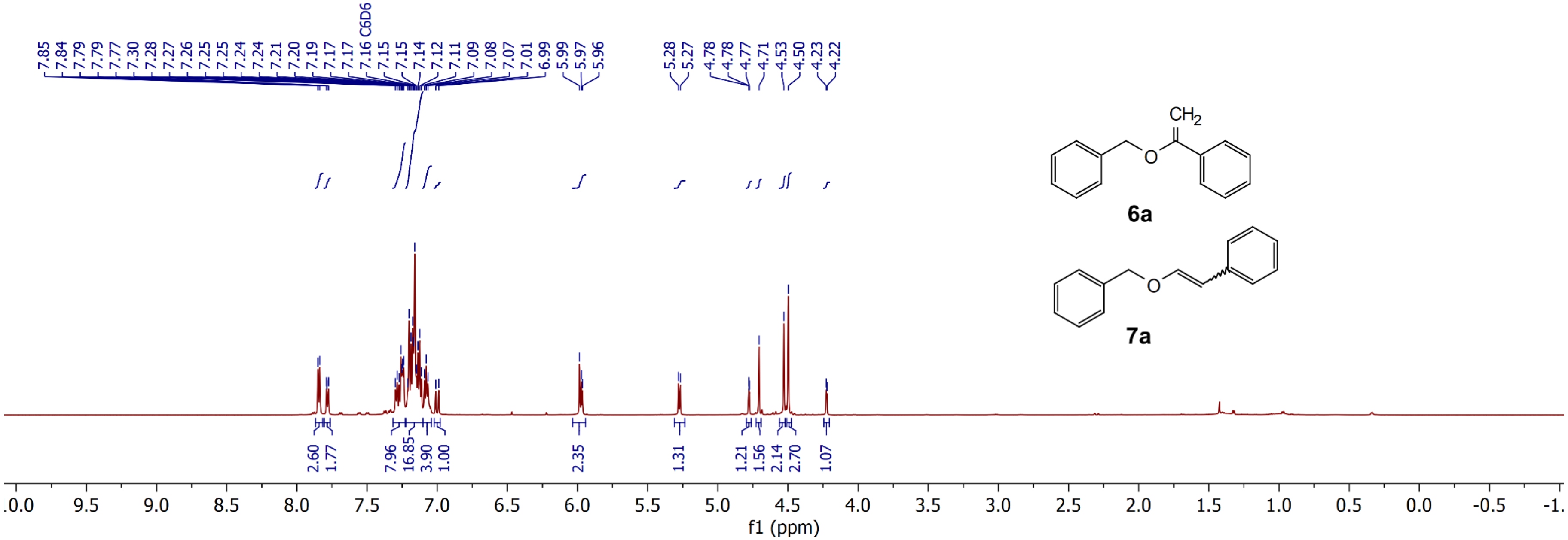

^{13}C{^{1}H}-NMR (151 MHz, C_6D_6) spectrum of regioisomers mixture **6a/7a** (mixture used in the **7a** preparation, obtained during optimization process)

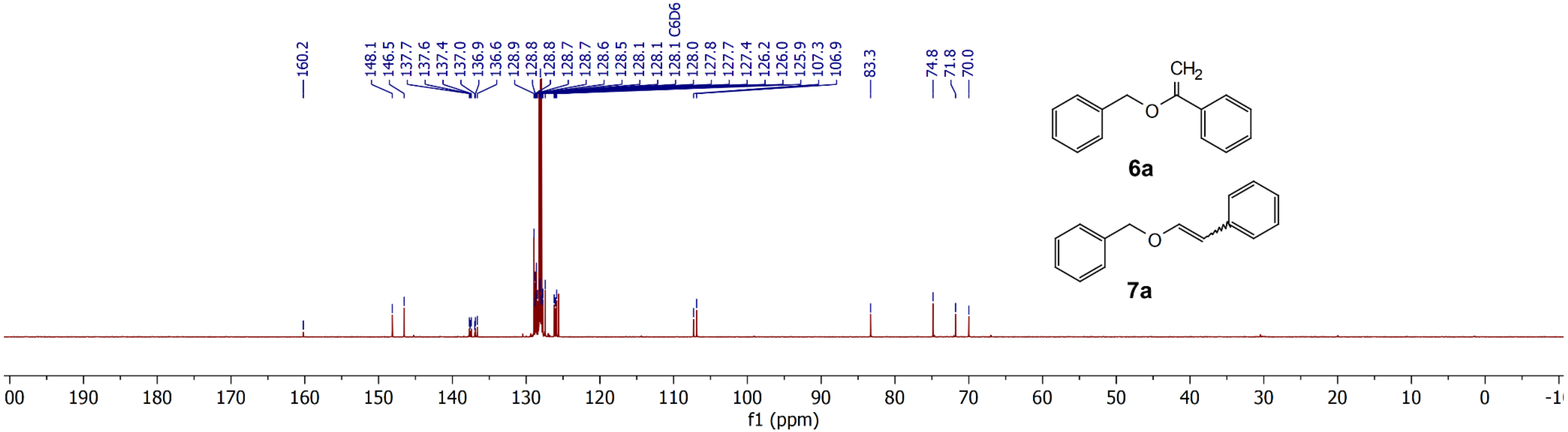

^{1}H-NMR (600 MHz, C_6D_6) spectrum of compound **7a** (procedure GP3)

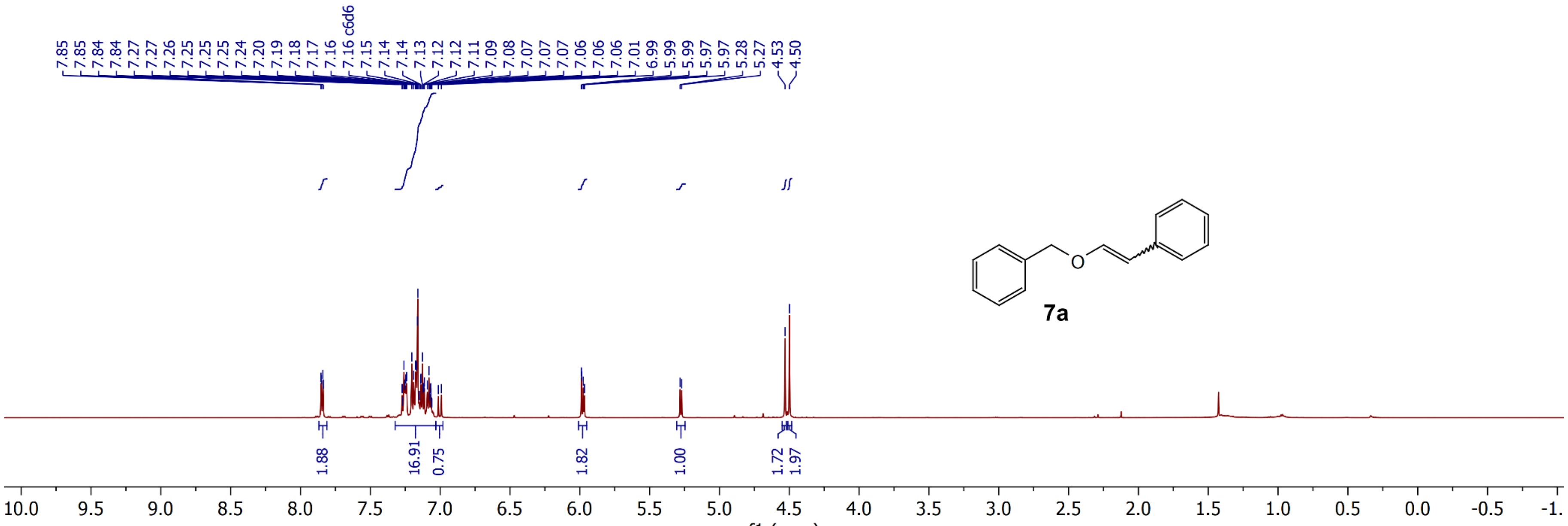

^{13}C{^{1}H}-NMR (151 MHz, C_6D_6) spectrum of compound **7a** (procedure GP3)

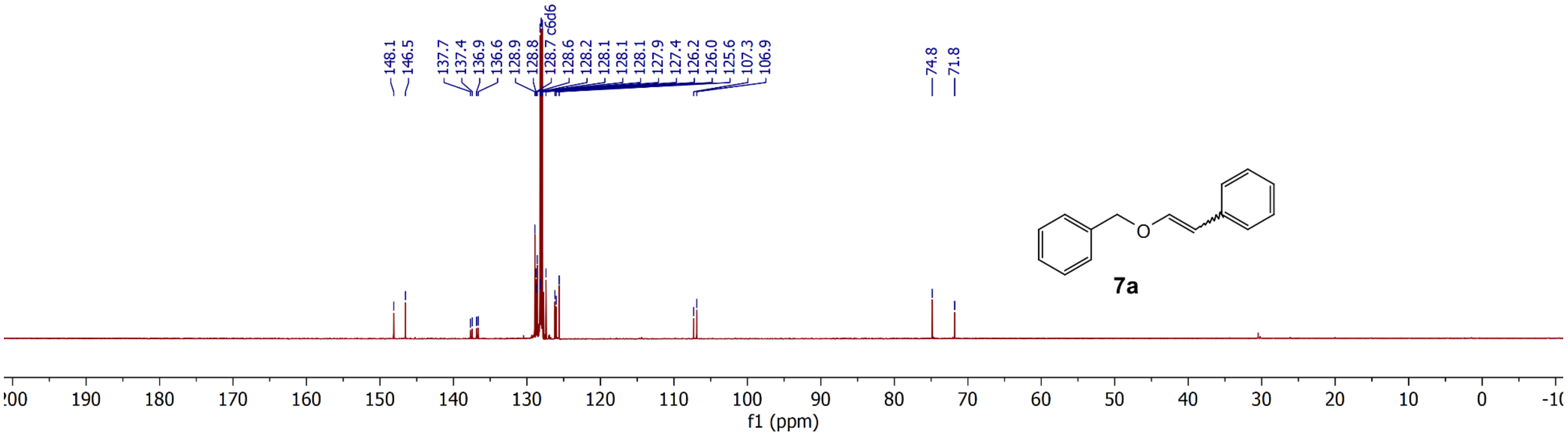

^{1}H-NMR (600 MHz, C_6D_6) spectrum of regioisomers mixture **6b/7b** (mixture used in the **7b** preparation, obtained during optimization process)

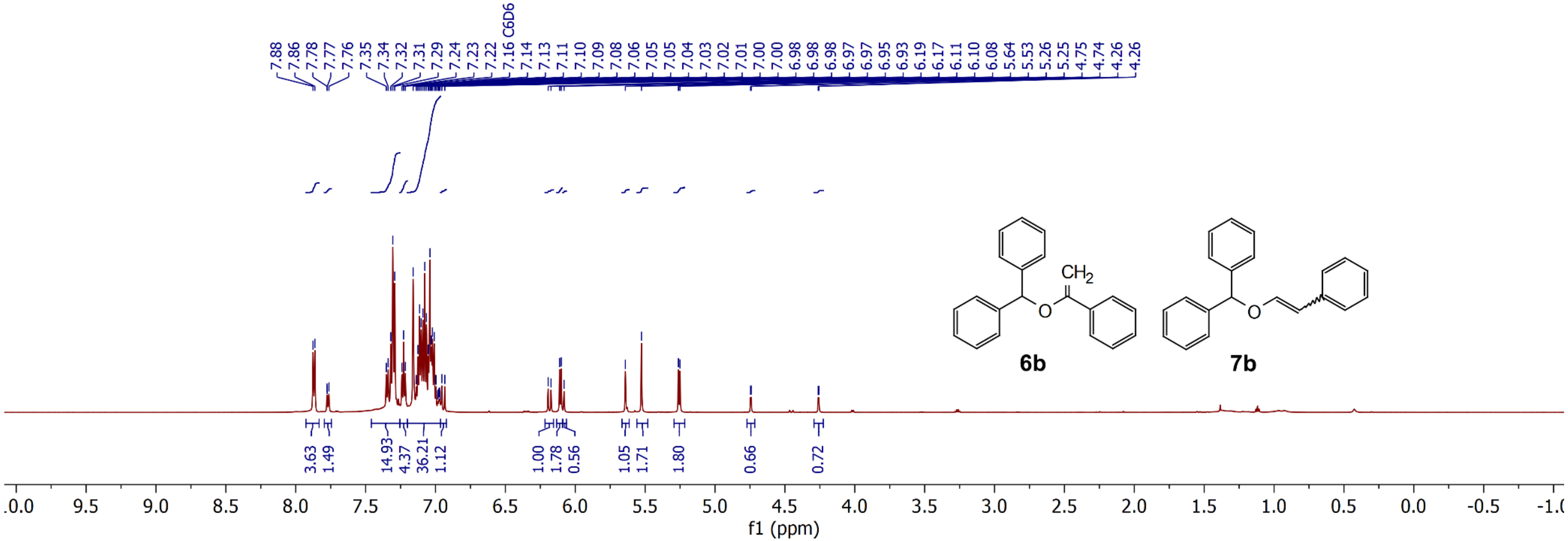

^{13}C{^{1}H}-NMR (151 MHz, C_6D_6) spectrum of regioisomers mixture **6b/7b** (mixture used in the **7b** preparation, obtained during optimization process)

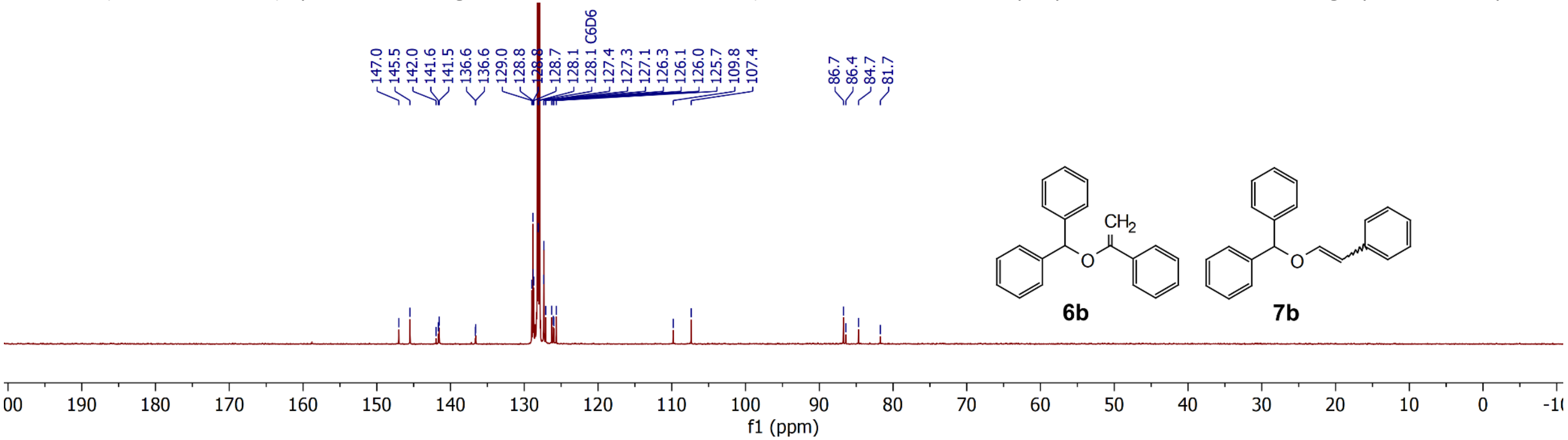

^{1}H-NMR (600 MHz, C_6D_6) spectrum of compound **7b** (procedure GP3)

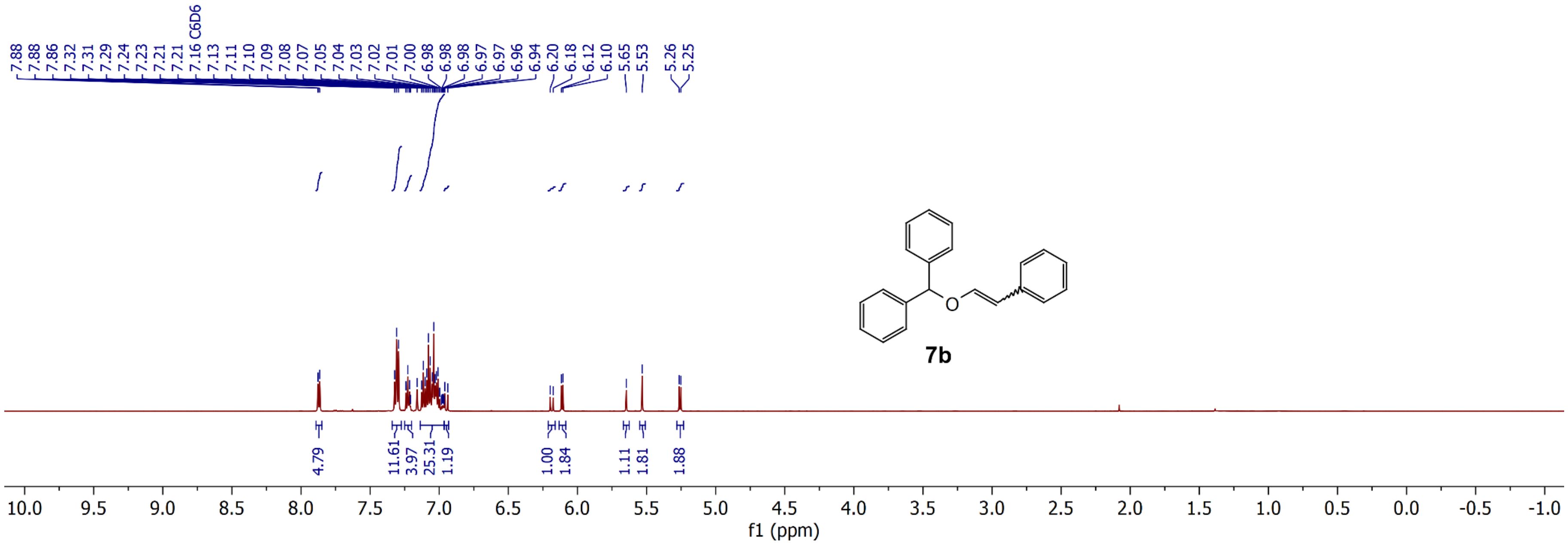

^{13}C{^{1}H}-NMR (151 MHz, C_6D_6) spectrum of compound **7b** (procedure GP3)

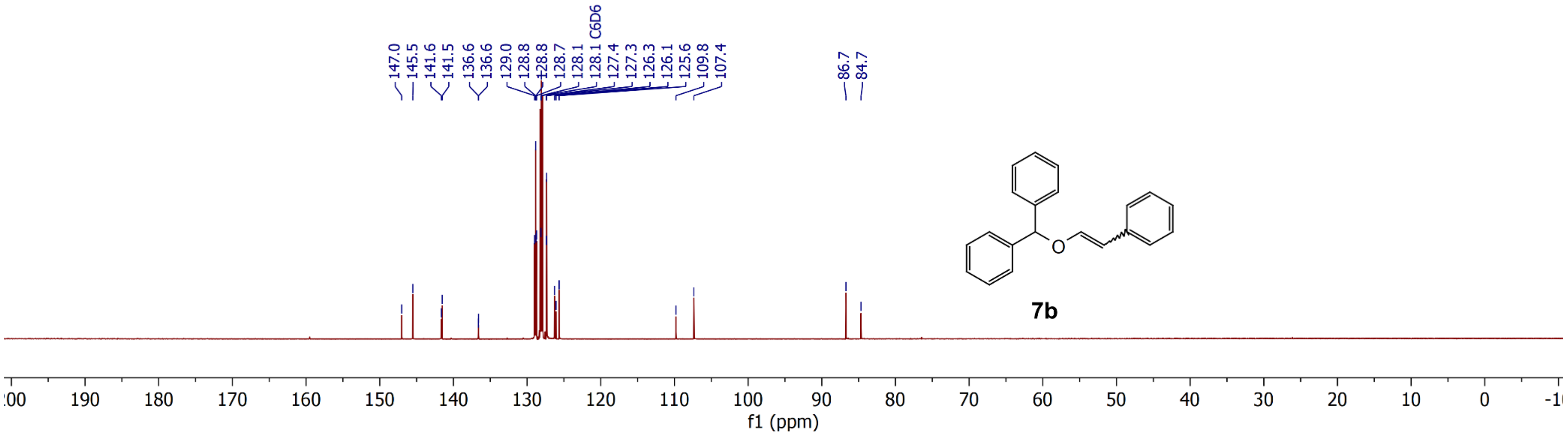

^{1}H-NMR (400 MHz, C_6D_6) spectrum of regioisomers mixture **6c/7c** (mixture used in the **7c** preparation, obtained during optimization process)

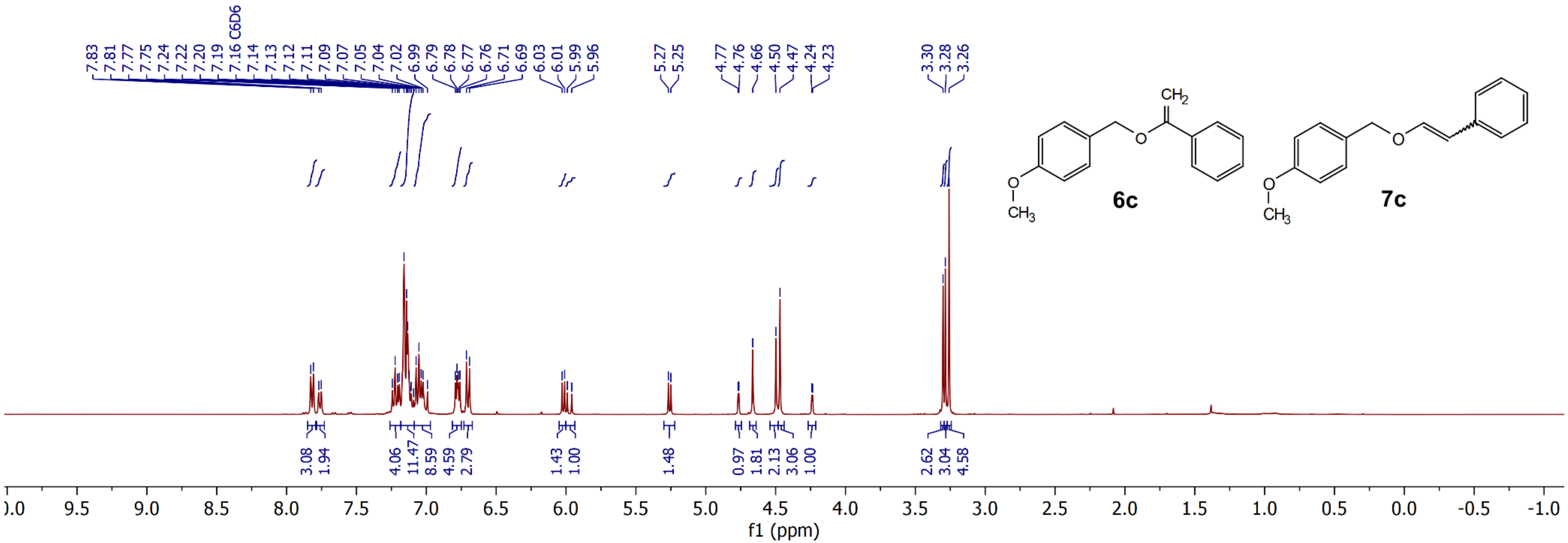

^{1}H-NMR (400 MHz, C_6D_6) spectrum of compound **7c** (procedure GP3)

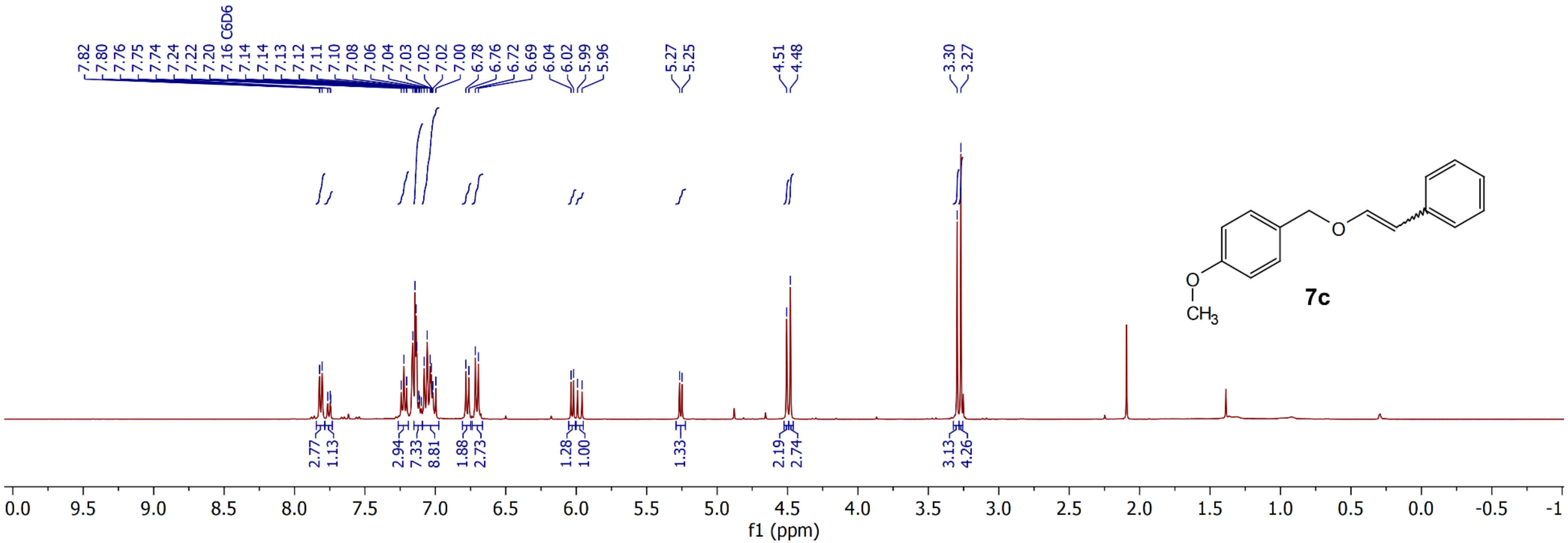

^{13}C{^{1}H}-NMR (101 MHz, C_6D_6) spectrum of compound **7c** (procedure GP3)

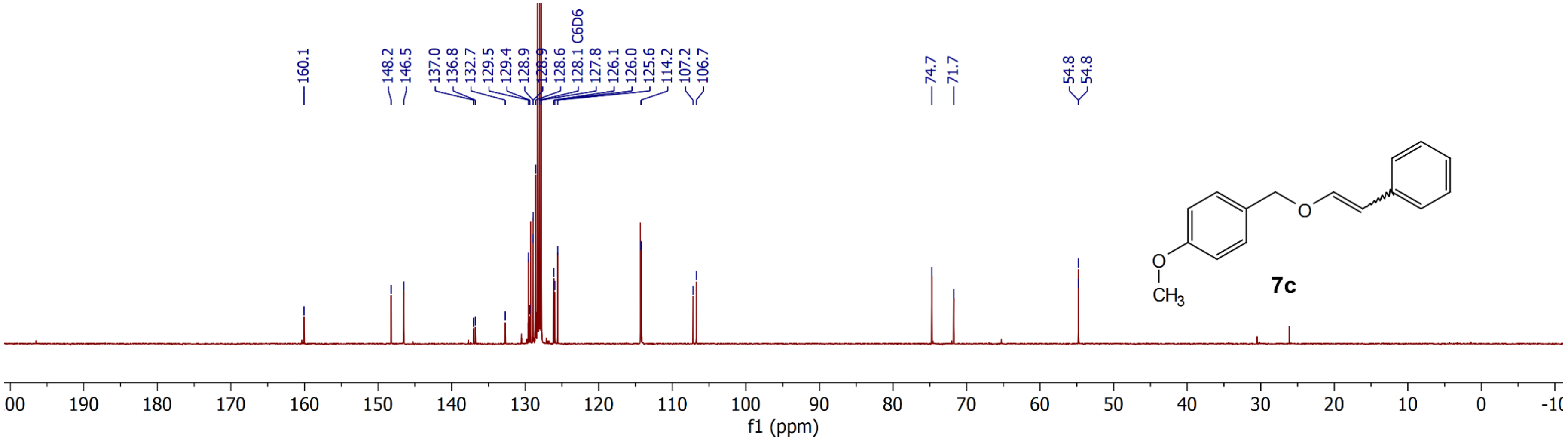

^{1}H-NMR (600 MHz, C_6D_6) spectrum of compound **7m** (procedure GP3)

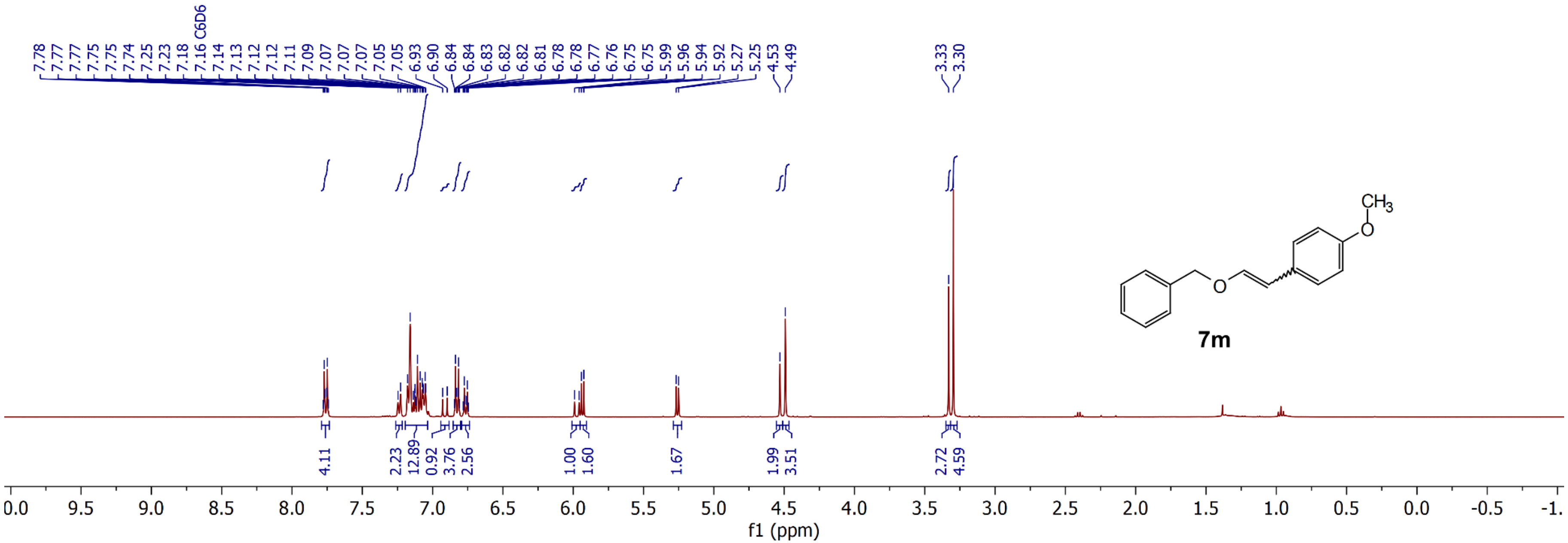

^{13}C{^{1}H}-NMR (151 MHz, C_6D_6) spectrum of compound **7m** (procedure GP3)

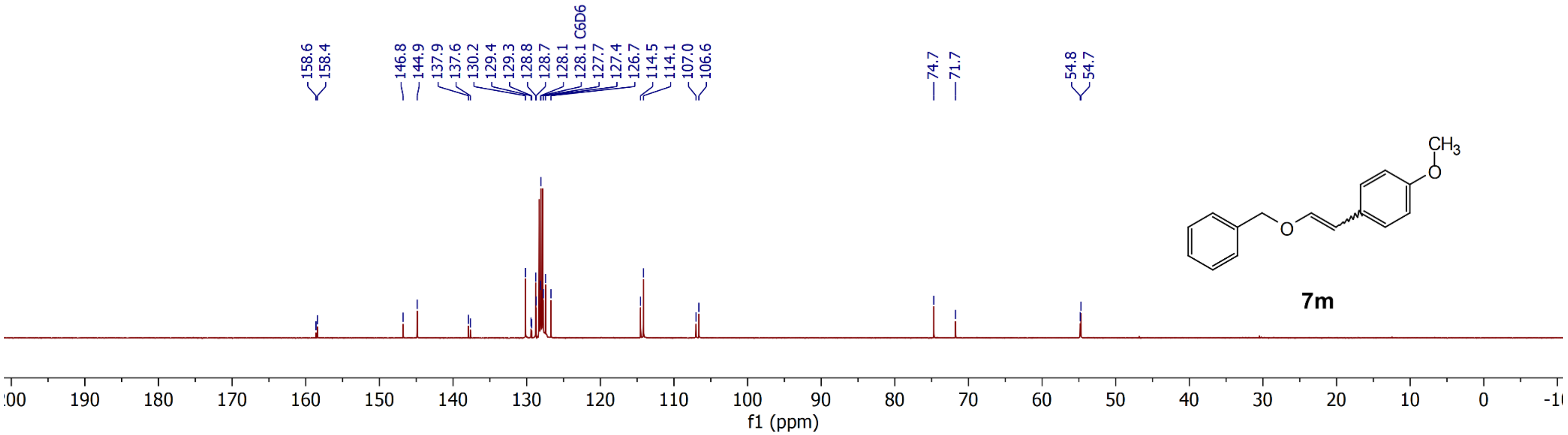

^{1}H-NMR (400 MHz, C_6D_6) spectrum of compound **7n** (procedure GP3)

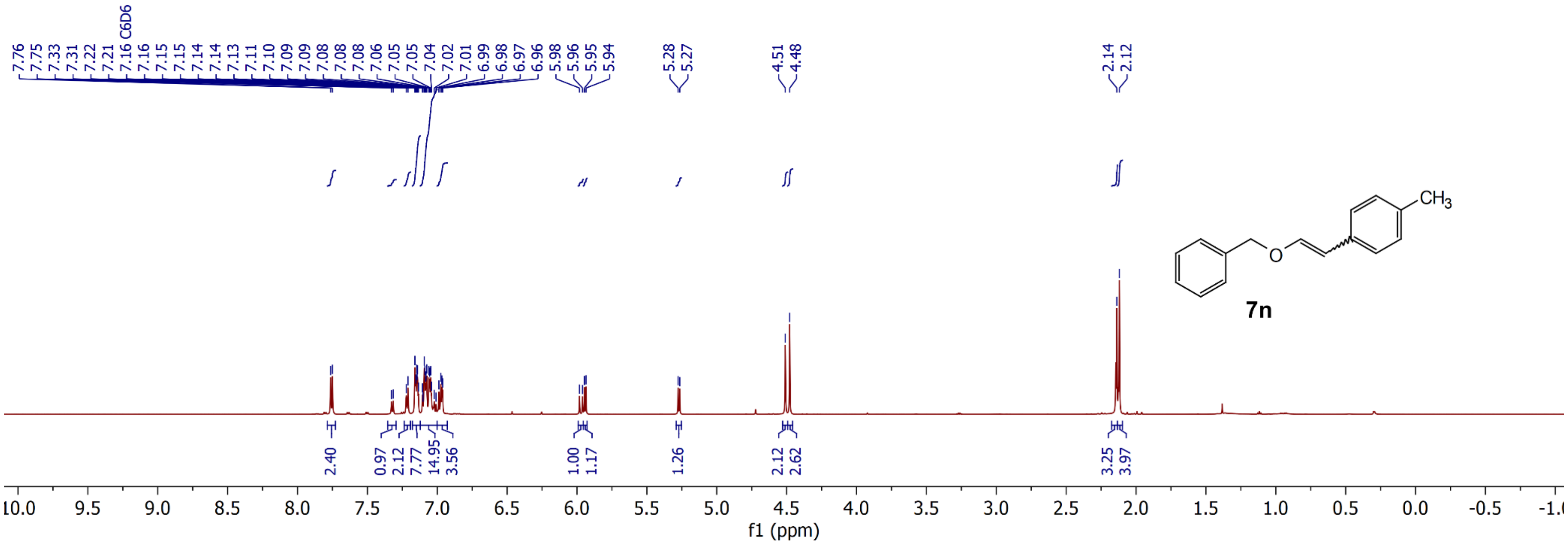

^{13}C{^{1}H}-NMR (101 MHz, C_6D_6) spectrum of compound **7n** (procedure GP3)

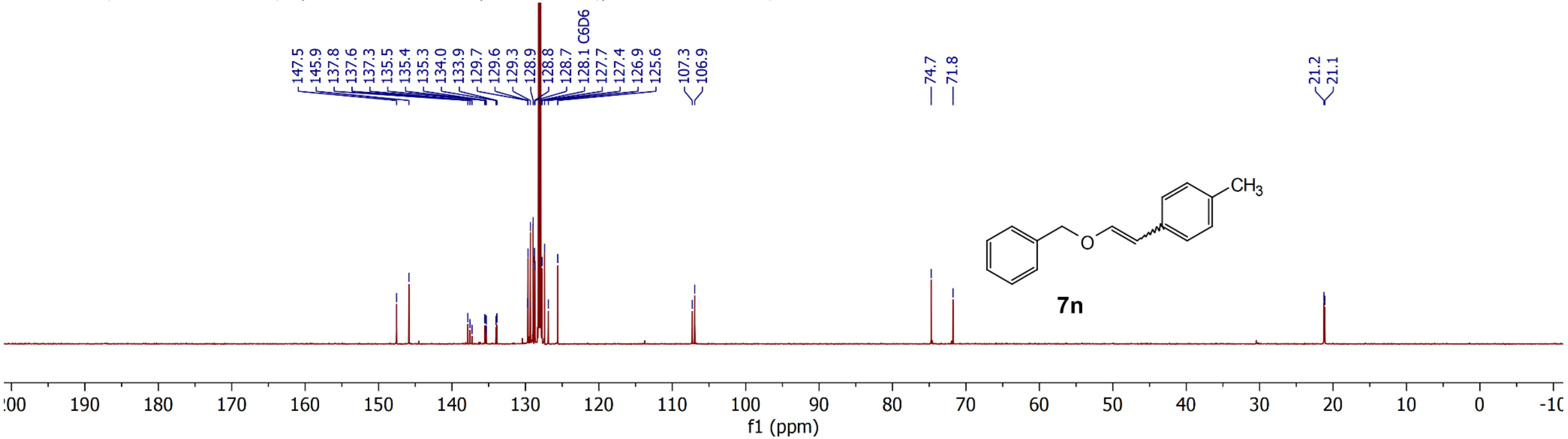

^{1}H-NMR (400 MHz, $CDCl_3$) spectrum of compound **9** (procedure GP2)

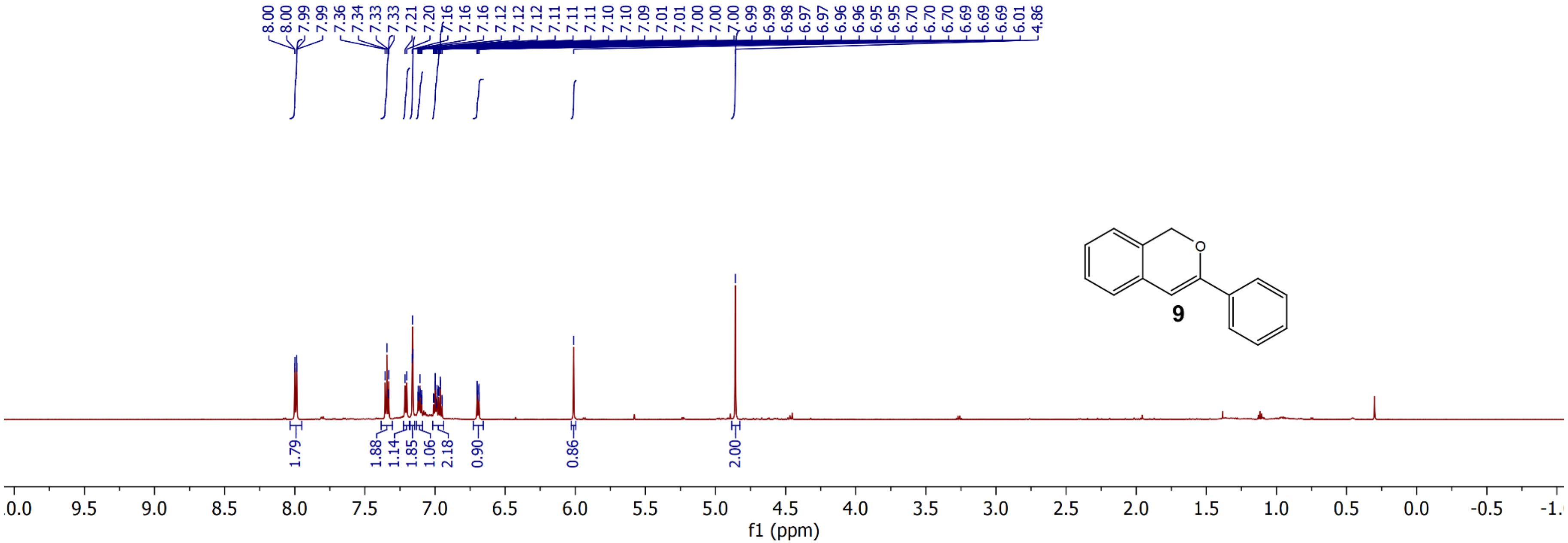

^{13}C{^{1}H}-NMR (101 MHz, $CDCl_3$) spectrum of compound **9** (procedure GP2)

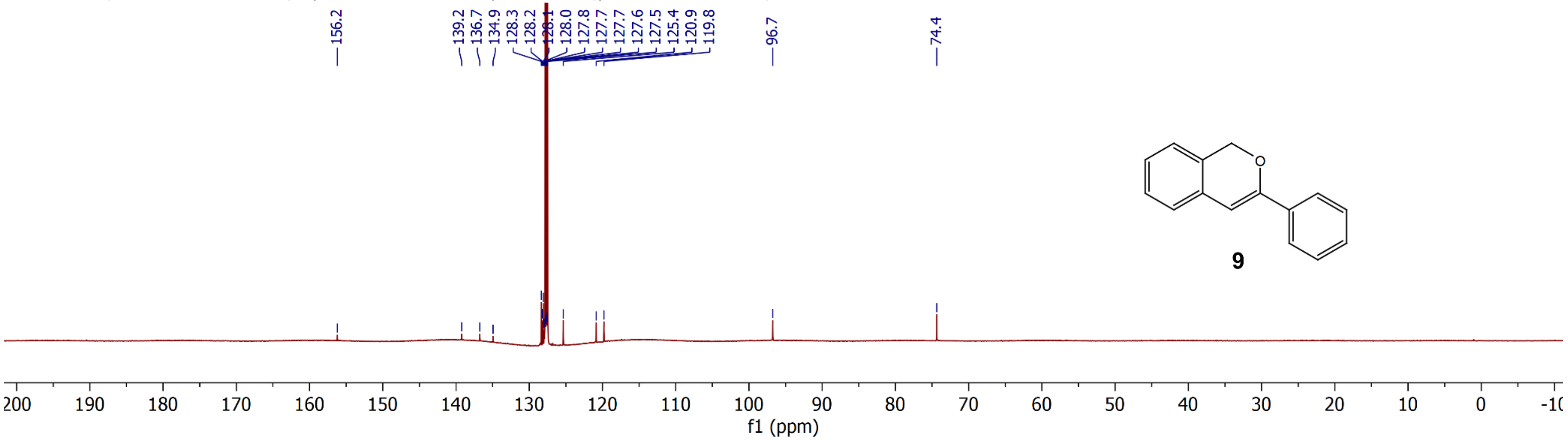

^{1}H-NMR (600 MHz, $CDCl_3$) spectrum of compound **11** (procedure GP2)

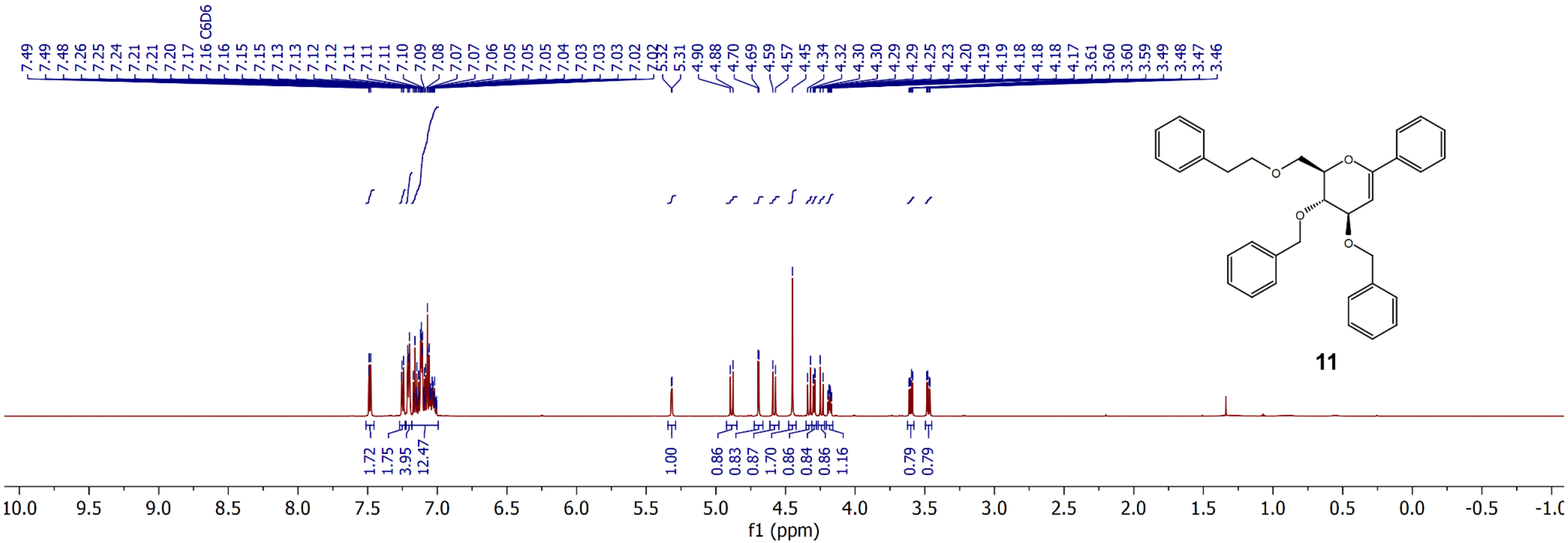

^{13}C{^{1}H}-NMR (151 MHz, $CDCl_3$) spectrum of compound **11** (procedure GP2)

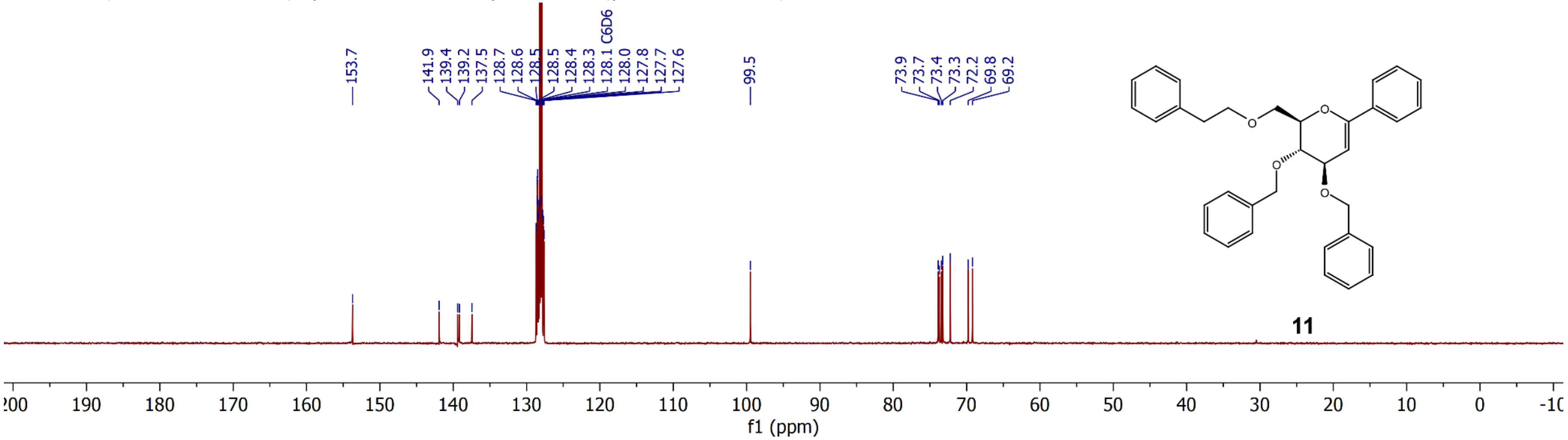

^{1}H-NMR (400 MHz, $CDCl_3$) spectrum of **1-((prop-1-enyloxy)methyl)benzene** (literature procedure[S16])

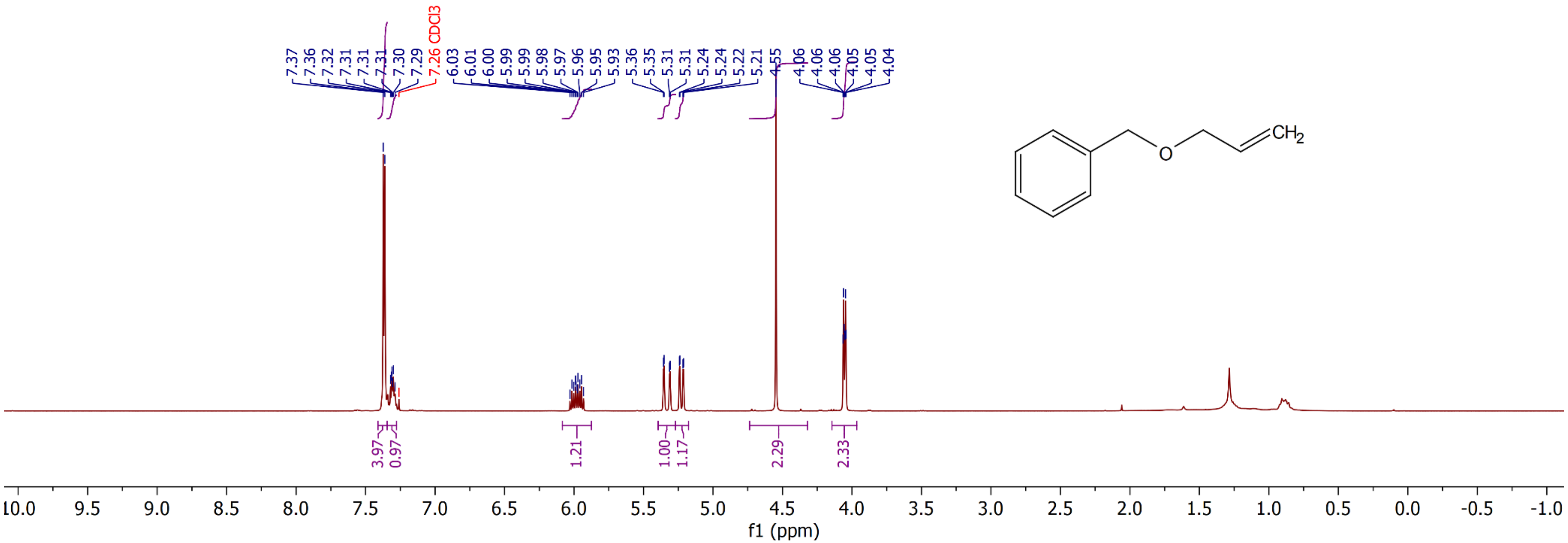

^{13}C{^{1}H}-NMR (101 MHz, $CDCl_3$) spectrum of **1-((prop-1-enyloxy)methyl)benzene** (literature procedure[S16])

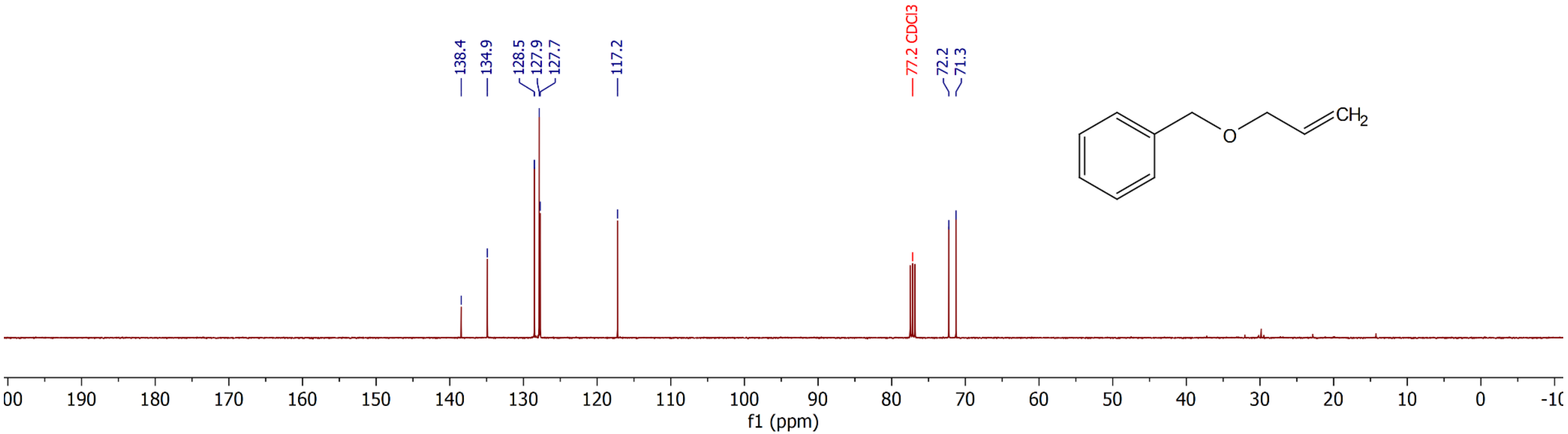

^{1}H-NMR (400 MHz, $CDCl_3$) spectrum of compound **14** (procedure GP1)

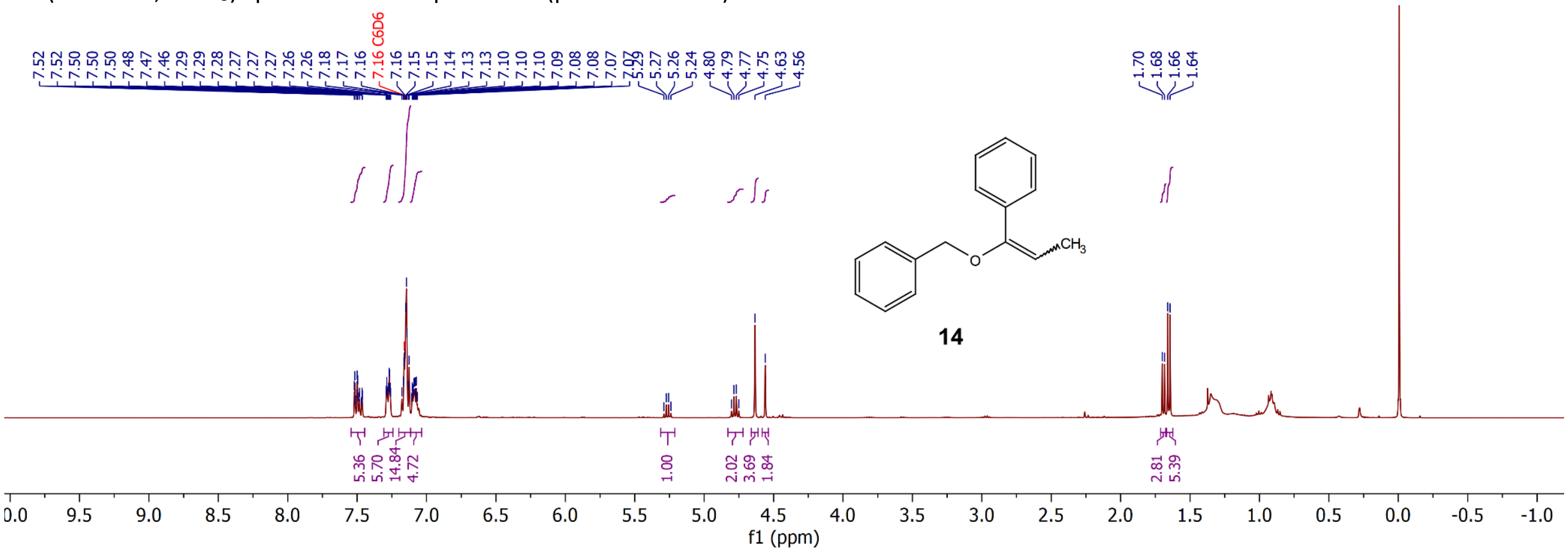

^{13}C{^{1}H}-NMR (101 MHz, $CDCl_3$) spectrum of compound **14** (procedure GP1)

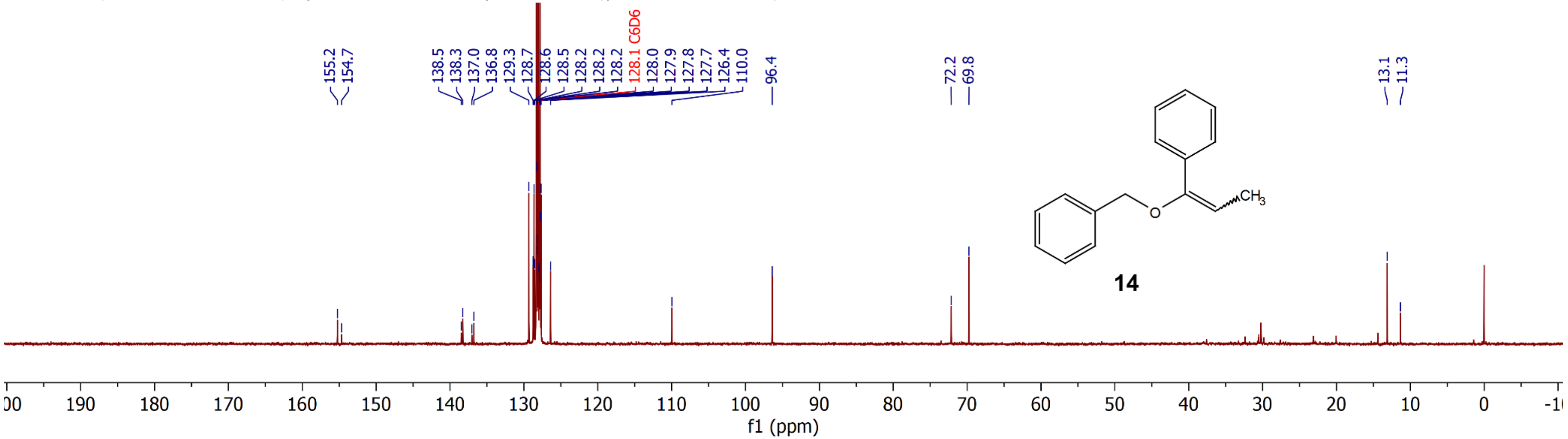

^{1}H-NMR (600 MHz, $CDCl_3$) spectrum of the mixture of compounds **14** and **15** (procedure GP2)

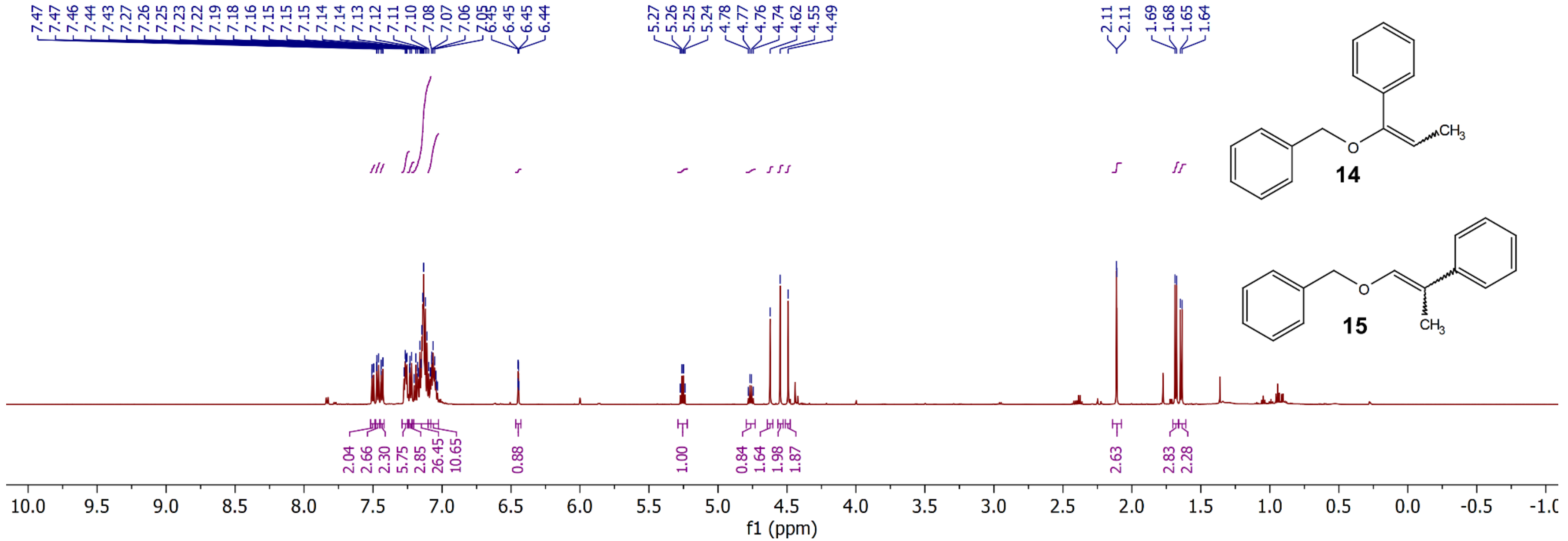

^{13}C{^{1}H}-NMR (151 MHz, $CDCl_3$) spectrum of the mixture of compounds **14** and **15** (procedure GP2)

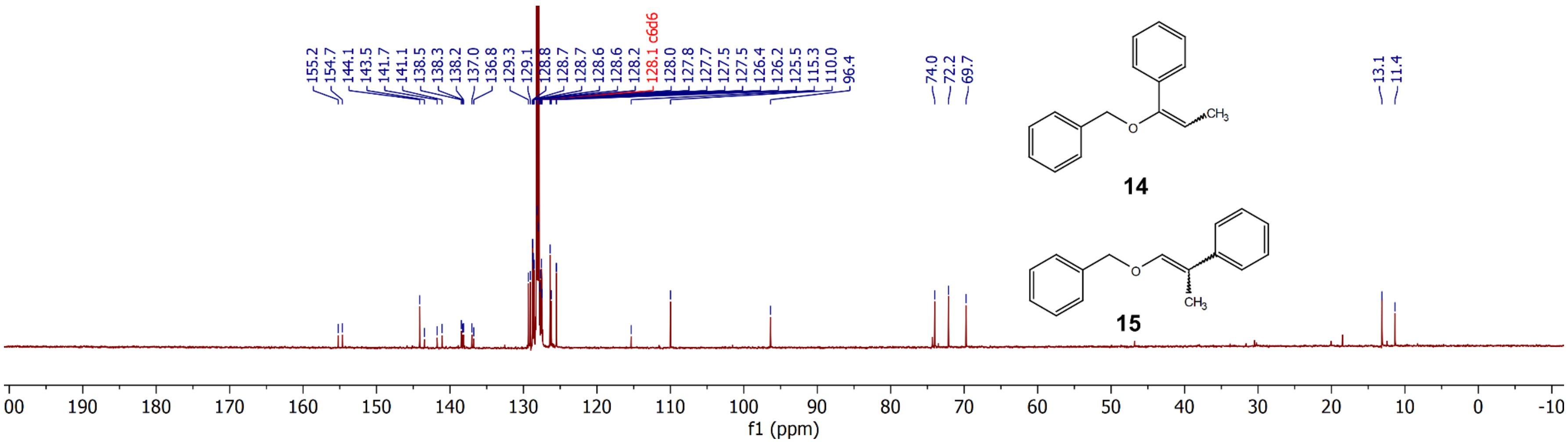

Supplement: Supplementary file 1 — Supporting Information [file ANIE-61-0-s001.pdf]
